# 2,5-disubstituted bicyclo[2.1.1]hexanes as rigidified cyclopentane variants†

**DOI:** 10.1039/d3sc02695g

**Published:** 2023-07-12

**Authors:** Shashwati Paul, Daniel Adelfinsky, Christophe Salome, Thomas Fessard, M. Kevin Brown

**Affiliations:** a Department of Chemistry, Indiana University 800 E. Kirkwood Ave Bloomington IN 47405 USA brownmkb@indiana.edu; b SpiroChem AG Rosental Area, WRO-1047-3, Mattenstrasse 22 4058 Basel Switzerland

## Abstract

Identification of rigid counterparts for common flexible scaffolds is crucial to the advancement of medicinal chemistry. Here we showcase a new class of building blocks, 2,5-disubstituted bicyclo[2.1.1]hexanes that can act as rigidified *cis*-, or *trans*-1,3-disubstituted cyclopentanes, common motifs in drugs. The scalable synthesis of these structures was enabled through the use of C–H functionalization logic and cycloaddition reactions.

## Abstract 2

Identification of rigid counterparts for common flexible scaffolds is crucial to the advancement of medicinal chemistry. Herein, synthesis of new bicyclo[2.1.1]hexanes to act as rigidified cyclopentanes is described.

## Graphical abstract

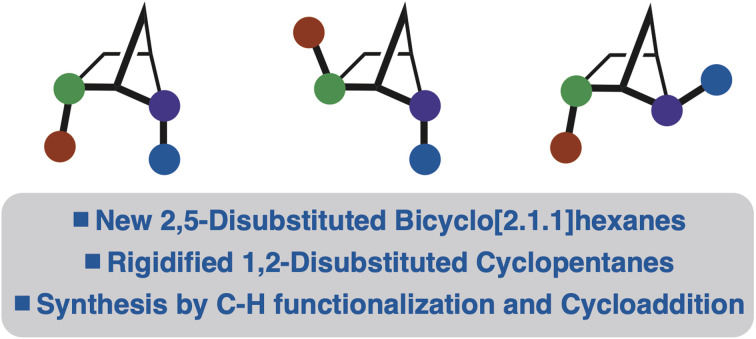

## Introduction

Conformational rigidification is an established strategy in medicinal chemistry to improve affinity, selectivity, and metabolic stability of a parent drug molecule by presenting defined exit vectors.^1^ As such, many bicyclic and polycyclic scaffolds have been designed that fulfill these features. Small rings such as, cyclobutane and cyclopropane moieties have also been extensively employed in medicinal chemistry due to their rigid structure.^2^

A recent analysis of the most common rings found in drugs ranked cyclopentane as the 18th most common.^3^ However, cyclopentane is flexible due to facile interconversion between the half-chair and envelope conformations. Despite considerable progress in the conformational restriction strategy,^1^ rigidification of cyclopentane rings has not seen significant development.

**Fig. 1:**
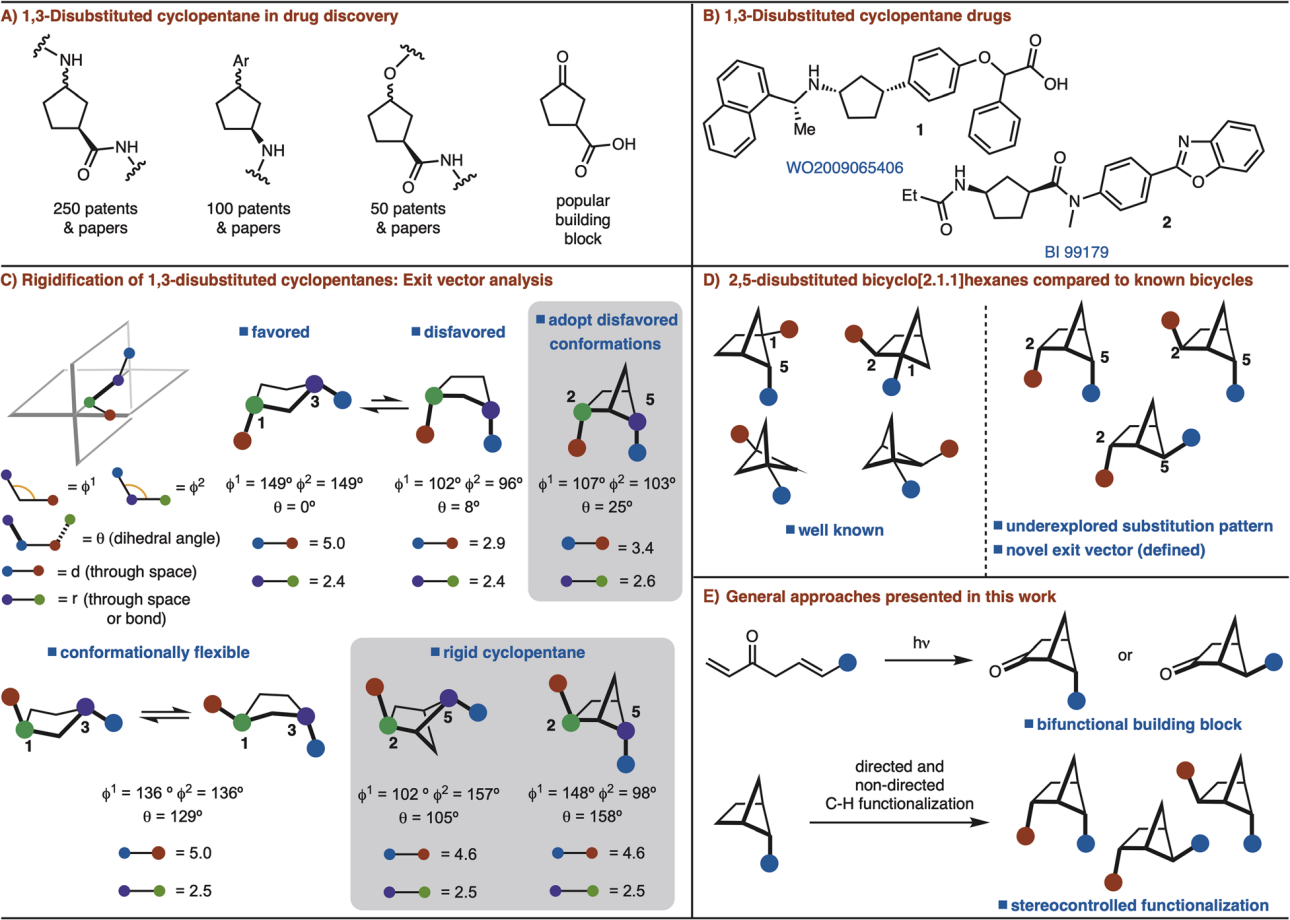
Bicyclo[2.1.1]hexanes.

Among substituted cyclopentane rings, 1,3-disubstitution is particularly common. These structures have appeared in numerous patents and journals (Fig. 1A). Furthermore, 3-oxocyclopentanecarboxylic acid is a popular building block for the synthesis of various 1,3-disubstituted cyclopentanes (Fig. 1A and B).^4^ Cognizant of the commonality of these structures, we sought to develop a rigidified 1,3-disubstituted cyclopentane variant in the form of a 2,5-disubtituted-bicyclo[2.1.1]hexane (Fig. 1C). In the case of *syn*-1,3-disubstituted cyclopentanes, rigidification can allow for disfavored conformations to be adopted (Fig. 1C). For *anti*-1,3-disubstituted cyclopentanes, which are conformationally flexible, the bicyclo[2.1.1]hexane analog can lock the conformation (Fig. 1C). In each case the exit vectors between the cyclopentane and bicyclo[2.1.1]hexane are similar (Fig. 1C).

Recent studies involving bicyclo[2.1.1]hexanes have focused on 1,2/1,5-disubstituted variants, which are not appropriate for rigidification of 1,3-disubstituted cyclopentanes.^5,6^ The required 2,5-substitution pattern has only been prepared in low yields (Fig. 1D).^7^ In addition, synthesis of this substitution pattern is challenging as four stereoisomers is possible. In this manuscript, we address these challenges though development of efficient routes for 2,5-disubstituted bicyclo[2.1.1]hexanes synthesis and diastereoselective further functionalization (Fig. 1D).

The most common method for synthesizing bicyclo[2.1.1]hexanes is a crossed [2 + 2]-cycloaddition reaction.^5*a*^ However, the synthesis of bicyclo[2.1.1]hexanes *via* crossed [2 + 2] cycloaddition is limited to preparing structures with a bridgehead substituents.^7^ Our interest is to establish a route that can incorporate two functional handles so that the target can be easily manipulated for rapid incorporation into drug molecules. In an orthogonal approach, we also envisioned that C–H functionalization logic could be applied to substitute the bridging positions of a mono substituted bicyclo[2.1.1]hexane.^8^ Thus, two distinct strategies are presented to allow for the synthesis of diverse *syn*- and *anti*-2,5-disubstituted bicyclo[2.1.1]hexanes by either [2 + 2] cycloaddition or C–H functionalization (Fig. 1E).

## Results and discussion

**Scheme 1:**
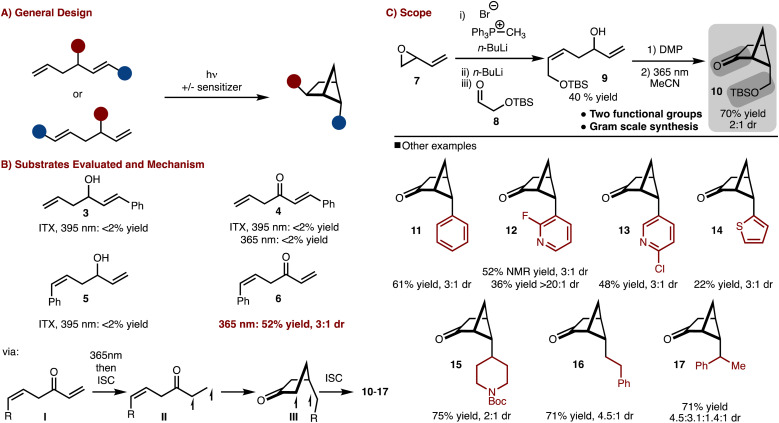
Crossed [2 + 2]-cycloaddition.

To gain access to a wide array of bicyclo[2.1.1]hexane molecular diversity, a crossed [2 + 2]-cycloaddition strategy was pursued (Scheme 1A). Several substrate classes (3–6) were examined under both direct excitation as well as sensitized with ITX (*i*-prthioxanthone) as shown in Scheme 1B. It was found that judicious positioning of the substituents and oxidation state was necessary as only substrate 6 under direct irradiation underwent cycloaddition (see the SI† for more details).^9^ The reaction likely proceeds *via* excitation of the ketone (I) to the S_1_ (*n*–π*) followed by rapid intersystem crossing (ISC) to the *T*_1_ (π–π *) (II). 1,5-radical addition to the alkene results in the formation of III, which upon ISC and radical recombination results in the formation of the products. The observed diasteroselectivity is likely the result of positioning of the R-group away from the bridging methylene hydrogen that projects over the four-membered ring.

With this knowledge in hand the synthesis of bifunctional bicyclo[2.1.1]hexane building block was pursued (Scheme 1C). Allylic/homoallylic alcohol 9 could be readily prepared from butadiene oxide *via* a three-step one pot procedure.^10^ Subsequent oxidation with DMP and exposure to 365 nm LEDs allowed for a crossed [2 + 2]-cycloaddition to occur to generate 10 in 70% yield and 2 : 1 dr. Thus in only two steps, bicyclo[2.1.1]hexane 9, with two functional group handles, could be easily prepared on multi gram scale.^11^

Furthermore, the scope could be expanded to allow for diverse product formation (products 11–17, Scheme 1C). The products were generally formed in ∼3 : 1 dr. The approach was tolerant of both aryl (product 11), heteroaryl (products 12–14) and alkyl substitution (products 15–17). However, in some cases, the heteroaryl starting materials underwent polymerization under the photochemical conditions (*e.g.*, thiophene), which resulted in lower yield.

**Scheme 2:**
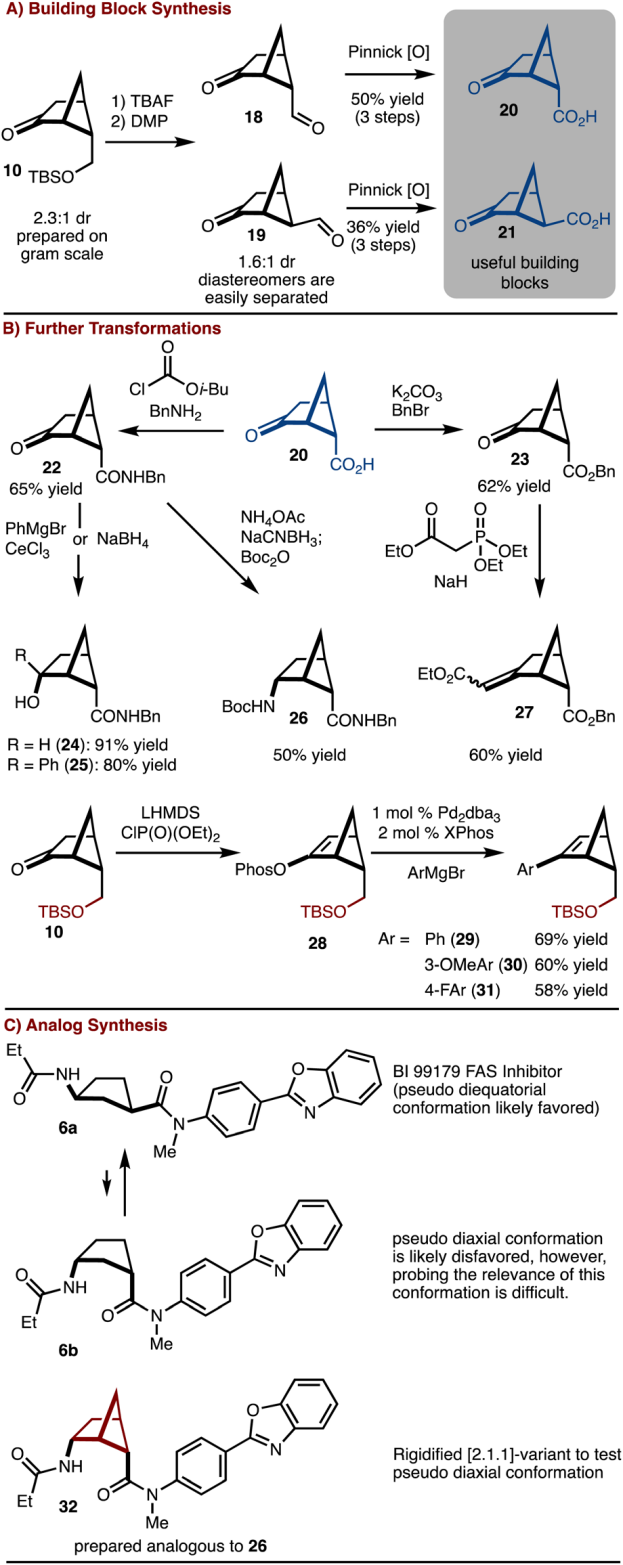
Building block synthesis and reactions.

The silyl ether product 10 was found to be particularly useful as deprotection oxidation led to the formation of readily separable aldehydes 18 and 19 (Scheme 2). Oxidation to the corresponding acids allowed for synthesis of useful building blocks 20 and 21, which are rigidified variants of the common building block 4. These keto/acid building blocks could be easily elaborated to various compounds. For example, esterification and olefination results in the formation of 27. Alternatively, amide bond formation followed by addition of NaBH_4_ or PhMgBr results in 24 and 25, respectively. In addition, reductive amination can be carried out to provide 26. This intermediate can be useful in the construction of rigid peptidomimetics.^12^ In all cases involving the ketone, the products were generated as single observable diastereomers by nucleophilic attack from the convex face of the bicyclo[2.1.1]hexane. In addition, bicyclo[2.1.1] hexenes 29–31 could be prepared by cross coupling of a generated enol phosphate 28. It should be emphasized that synthesis of bicyclo[2.1.1]-hexenes is challenging and finds little precedent.^13^

To further underscore the significance of the rigidified building blocks, a case study was explored (Scheme 2C). FAS inhibitor BI 99179 (6a) is a *syn*-1,3-substituted cyclopentane.^14^ The likely preferred conformation is that in which the two substituents are diequatorial. However, an important question to ask is if the diaxial conformation 6b is biologically relevant either in an induced fit scenario,^15^ or results in off target complications. Probing this hypothesis would be challenging with access to only the parent compound. Here, the bicyclo[2.1.1]hexane variant 32 can be prepared and used to address this question. Future studies will focus on comparative biological studies.

**Scheme 3:**
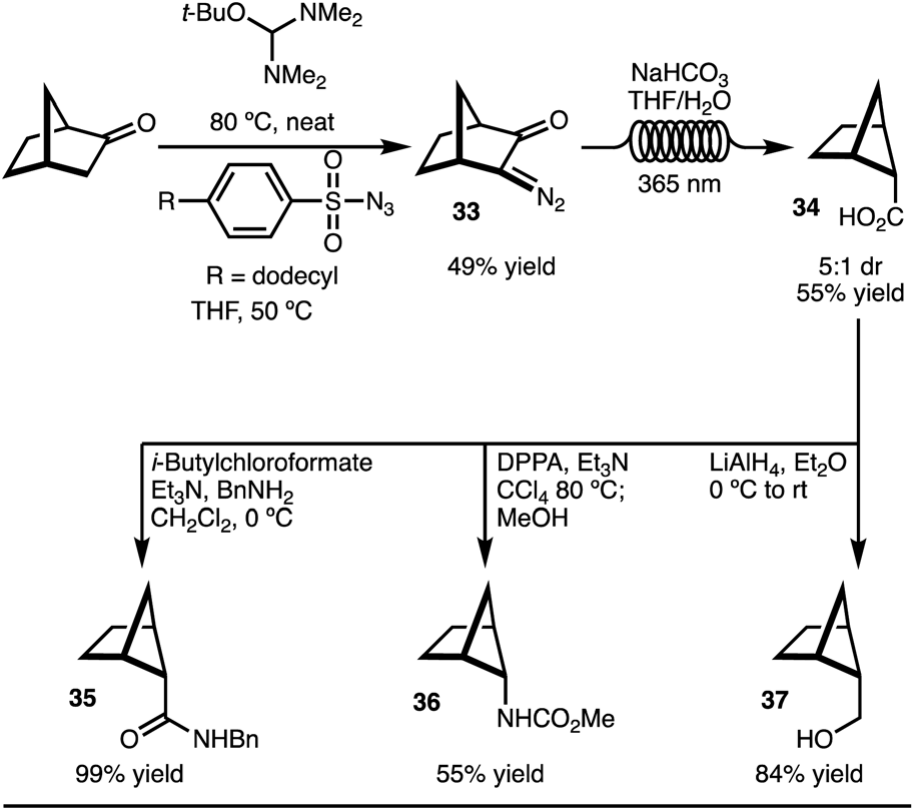
Synthesis of bicyclo[2.1.1]hexane carboxylic acid.

To explore the C–H functionalization approach towards 2,5-disubstituted bicyclo[2.1.1]-hexanes, a suitable precursor was needed (Scheme 3). It was envisioned that carboxylic acid 34 could serve as an appropriate starting material. This intermediate was easily prepared on gram scale in 5 : 1 dr from norbornanone by application of a photochemical Wolff rearrangement of diazoketone 33 (Scheme 3).^16^ Two key factors were crucial to realize the synthesis of gram quantities of product and make this approach a viable synthetic strategy accessible to most chemists: (1) use of commercially available, safe, and inexpensive dodecylbenzenesulfonylazide, and (2) the application of the photochemical Wolff rearrangement in flow. Finally, to demonstrate that the carboxylic acid 34 is a useful building block, the conversion to amide (35), carbamate (36), and alcohol (37) functional groups were easily accomplished. All of these substituents are of high relevance to medicinal chemists.

**Scheme 4:**
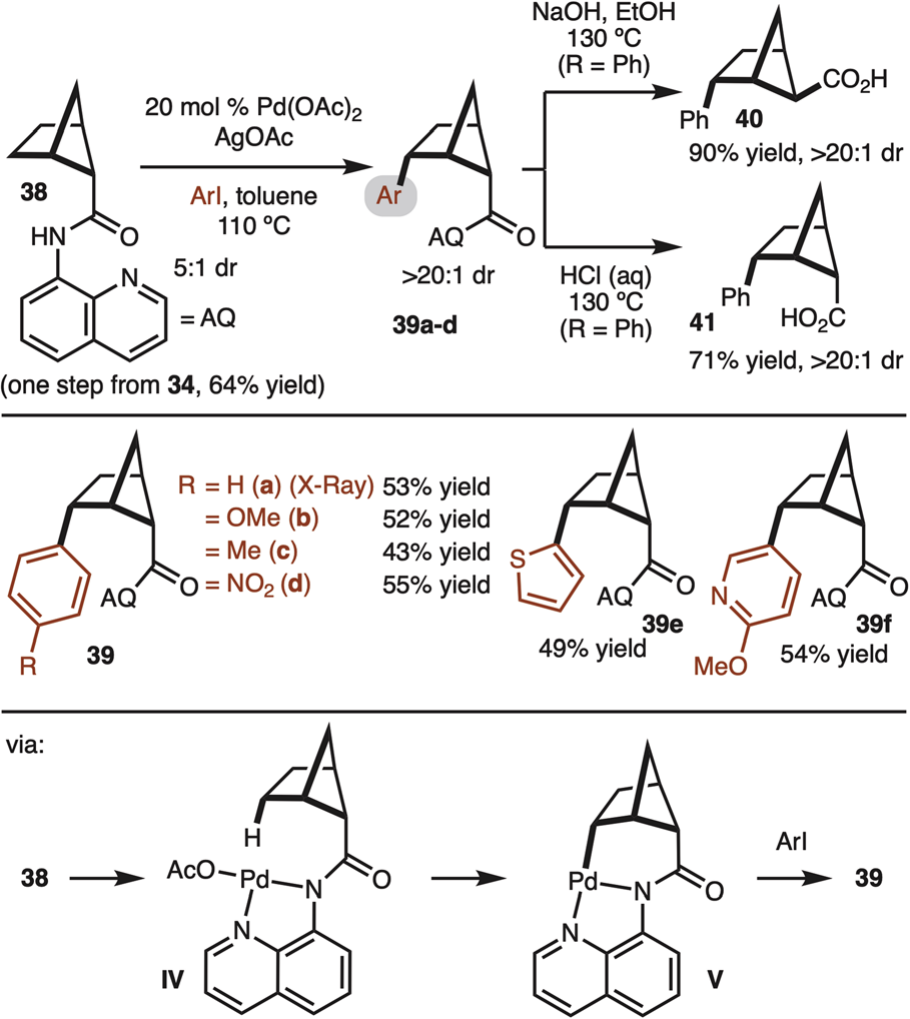
Directed C–H arylation.

With access to gram quantities of 34, C–H functionalization was explored. The first successful strategy is illustrated in Scheme 4. Pioneering work from Daugulis and Yu have demonstrated the utility of the 8-aminoisoquinoline (AQ) for directed Pd-catalyzed C–H functionalization.^17^ This system could be used to convert 38 to aryl bicyclo[2.1.1]hexanes with good control of diastereoselectivity (structure 39 confirmed by X-ray). The γ-selectivity of the reaction is likely the result positioning of the Pd-complex in IV close the C–H bond. Reaction at the β-site does not occur since the C–H bond is tertiary and orthogonal to the amide. A brief survey of substrates demonstrated tolerance to electron-rich (product 39b), electron-poor (product 39d), and heterocyclic aryl iodides (products 39e,f). Notably, while the starting material was a 5 : 1 mixture of diastereomers, the products were formed in >20 : 1 dr. At this stage it is not clear if the minor diastereomer decomposes or undergoes epimerization under the rection conditions. The relative stereochemistry was determined by X-ray crystallography analysis (Scheme 4). Next, the removal of 8-aminoquinoline was attempted. Under basic hydrolysis conditions, the carboxylic acid undergoes epimerization to deliver thermodynamically favored product 40. Whereas when the hydrolysis was conducted under acidic conditions, the relative configuration of the acid moiety was retained 41.^18^

**Scheme 5:**
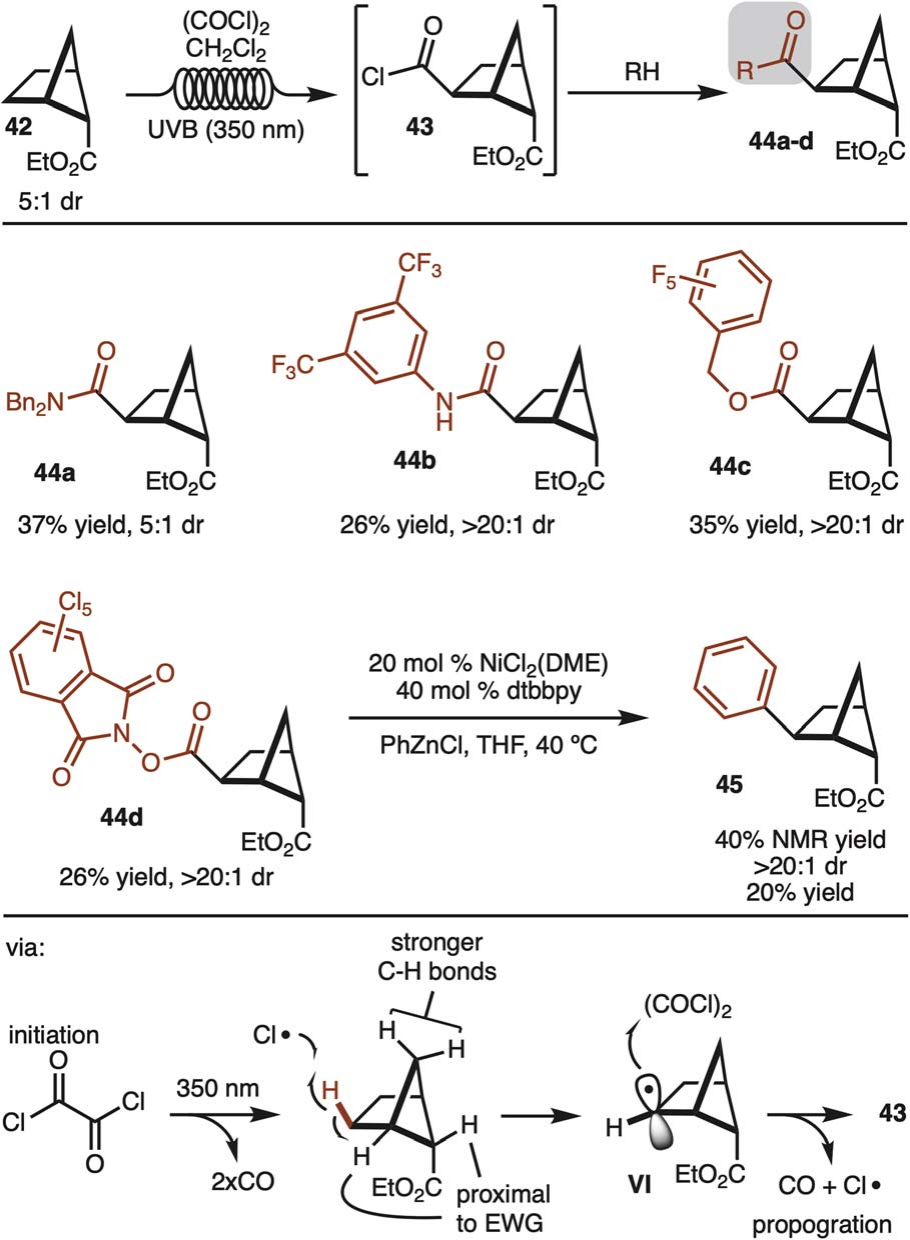
C–H carboxylation.

Alternative strategies that do not use directing groups were also explored. It was discovered that irradiation of ester 42 (5 : 1 dr) with UVB (350 nm) in the presence of oxalyl chloride led to formation of acid chloride 43 with complete stereocontrol for the newly formed C–C bond (Scheme 5).^19^ The generated acid chloride (43) could be intercepted with various alcohols or amines to provide products 44a–d. While the starting ester was a 5 : 1 mixture of diastereomers, the products resulting from the minor diastereomer could generally be separated by simple column chromatography. In addition, redox active ester 44d could also be generated and subjected to decarboxylative cross coupling to provide access to 45 as a single observable diastereomer.^20^ Notably, this route offers a strategy to synthesize the other diastereomer than the one shown in Scheme 4.

The reaction likely proceeds by initial generation of chlorine radical by irradiation of oxalyl chloride by UV light.^19^ Chlorine radical then abstracts the indicated hydrogen to generate VI. The selectivity in the reaction can be rationalized in that chlorine radical reacts with the weakest C–H bond. The bonds proximal to the ester are deactivated, whereas the methylene CH_2_ are stronger by virtue of a strain induced rehybridization.^21^ Finally, capture of the secondary radical by oxalyl chloride generates the product and chlorine radical to propagate the chain.

## Conclusions

In summary, an approach towards the rigidification of medicinally relevant 1,3-disubstituted cyclopentanes with 2,5-disubstituted bicyclo[2.1.1]hexanes is presented. To prepare the desired structures, two distinct strategies were devised. One involving [2 + 2]-cycloaddition that allowed for the synthesis of keto-acid building blocks. Whereas a C–H functionalization strategy allowed for incorporation of aryl and carboxyl groups with control of stereochemistry. Overall, a diverse range of molecular architectures can be prepared that constitute an enrichment of the toolbox of drug designers and medicinal chemists.

## Data availability

The ESI† contains method description, product characterization data, and NMR spectra.

## Author contributions

S. P., C. S., T. F., and M. K. B. designed the project. Experiments were carried out by S. P. and D. A. S. P. and M. K. B. co-wrote the manuscript and all authors provided comments.

## Conflicts of interest

There are no conflicts to declare.

## Supplementary Material

SC-014-D3SC02695G-s001

SC-014-D3SC02695G-s002
