# Supplementary material for: 2,5-disubstituted bicyclo[2.1.1]hexanes as rigidified cyclopentane variants
Source: Chem Sci. 2023 Jul 12;14(30):8070–5. doi: 10.1039/d3sc02695g (PMC10395266; doi:10.1039/d3sc02695g)
Supplement: SC-014-D3SC02695G-s001 [file SC-014-D3SC02695G-s001.pdf]

Electronic Supplementary Material (ESI) for Chemical Science.
This journal is © The Royal Society of Chemistry 2023

2,5-Disubstituted Bicyclo[2.1.1]hexanes as Rigidified Cyclopentane Variants for Medicinal Chemistry

Shashwati Paul[1], Daniel Adelfinsky[1], Christophe Salome[2], Thomas Fessard[2], and M. Kevin Brown*[1]

[1]Department of Chemistry, Indiana University, 800 E. Kirkwood Ave. Bloomington, IN, 47401.

[2]SpiroChem AG, Rosental area, WRO-1047-3, Mattenstrasse 22, 4058 Basel, Switzerland

Supplementary Information – Table of Contents

1. General information……………………………………………………………………..2
2. Reagents………………………………………………………………………………………3
3. [2+2]-cycloaddition reaction…………………………………………………………………6
4. Building block synthesis and further functionalization…………………………………….21
5. Synthesis and further functionalization of bicyclo[2.1.1]hexane-5-carboxylic acid……….39
6. Directed C-H functionalization……………………………………………………………….45
7. Photochemical C-H activation……………………………………………………………..54
8. Characterization data & X-ray data…………………………………………………………60
9. Reference………………………………………………………………………………………128

1. General information.

NMR: 1H NMR were recorded at room temperature (if mention otherwise) on a Varian I500, Bruker 500 or a Varian I600 sectrometer. Chemical shifs are reported in ppm from TMS with the residual solvent resonance as the internal standard (CDCl3; 7.26 ppm). Data are reported as follows: chemical shifts, multiplicity (s= singlet, d= doublet, t= triplet, q = quartet, br= broad, m = multiplate), coupling constants (Hz), and integration. 13C NMR spectra were recorded on a Bruker 500 (126 MHz) spectrometer with complete proton decoupling. Chemical shifts are reported in ppm from TMS with the residual solvent resonance as the internal standard (CDCl3; 77.16 ppm).

IR: Infared spectra were recorded on a Bruker Tensor ii FT-IR spectrometer, vmax in cm-1. Bnads are characterize as broad (br), strong (s), medium (m), and weak (w).

HRMS: High Resolution Mass Spectrometry analysis was obtained using Electrospray Ionization (EI) and reported as m/z (relative intensity). ESI was acquired using a Wayters/micromss LCT classics.

Solvents: Dichloromethane (CH_2Cl_2), Diethyl ether (Et_2O), Dimethyl formamide (DMF), Tetrahydrofuran (THF) were purified under a positive pressure of dry argon by passing through two columns of activated alumina. Toluene (PhMe) was purified under a positive pressure of dry argon by passing through activated alumina and Q5 (Grubbs apparatus).

Reaction: All reactions were carried out distilled and de-gassed solvents under an atmosphere of nitrogen in flame-dried glassware with standard Schlenk line technique. All work-up and purification were carried out using regent grade solvent under air.

Purification: Medium-pressure liquid chromatography (MPLC) technique was used for purification using a Teledyne ISCO CombiFlash Rf 150 instrument. Standard flash column chromatography (FCC) techniques using ZErO prep 60/40-63 μm silica gel were used for purification.

Flow-reactor: A Vapourtec easy photochem flow reactor with 10 ml reactor was used for the flow reaction.

2. Reagent and catalysts.

(Pentafluorophenyl)methanol was purchased from Oakwood Chemicals and filtered through a pad of silica before using.

1-iodo-4-methoxybenzene was purchased from Sigma Aldrich and filtered through a pad of silica before using.

1-iodo-4-methylbenzene was purchased from Sigma Aldrich and filtered through a pad of silica before using.

1-iodo-4-nitrobenzene was purchased from Sigma Aldrich and used as received.

1-Piperidinecarboxylic acid, 4-formyl-, 1,1-dimethylethyl ester was purchased from Combiblocks and filtered through a pad of silica before using.

2-((tert-butyldimethylsilyl)oxy)ethan-1-ol was purchased from Combi-blocks and used as received.

2-(Methylthio)aniline was purchased from Oakwood and filtered through a pad of silica before using.

2-Fluoropyridine-3-carboxyaldehyde was purchased from Combi-blocks and used as received.

2-iodothiophene was purchased from Matrix Scientific and filtered through a pad of silica before using.

2-phenylpropanal was purchased from Alfa Aesar and used as received.

3-phenylpropanal was purchased from Combi-blocks and used as received.

3,5-Bis-trifluoromethylaniline was purchased from Oakwood Chemicals and filtered through a pad of silica before using.

3-Methoxyphenylmagnesium bromide solution (1.0 M in THF) was purchased from Sigma -Aldrich and used as received.

4,4¡ä-di-tert-butyl-2,2¡äbipyridyl was purchased from Strem Chemicals and used as received.

4-Fluorophenylmagnesium bromide solution (2.0 M in diethyl ether) was purchased from Sigma -Aldrich and used as received.

6-chloronicotinaldehyde was purchased from Ark Pharm and used as received.

8-amino-quinoline was purchased from Oakwood and used as received.

Ammonium acetate was purchased from Mallinckrodt and used as received.

Benzaldehyde was purchased from Sigma Aldrich and used as received.

Benzyl bromide (BnBr) was purchased from Oakwood and filtered through a pad of silica before using.

Benzylamine (BnNH$_2$) was purchased from Fluka and filtered through a pad of silica before using.

Bicyclo[2.2.1]heptane-2-one was purchased from Ambeed and used as received.
Butadiene monoxide was purchased from Combi-blocks and used as received.
Cerium (III) chloride ($CeCl_3$) was purchased from Combi-blocks and oven dried before use.
delta-Isoamylene was purchased from Oakwood and used as received.
Dess-Martin-Periodinane (DMP) was purchased from Synthonix and used as received.
di-tert-butyl dicarbonate (Boc_2O) was purchased from Oakwood and used as received.
Dibenzylamine was purchased from Sigma Aldrich and filtered through a pad of silica before using.
Diethyl chlorophosphite was purchased from Oakwood and filtered through a pad of silica before using.
Diphenylphosphoryl azide (DPPA) was purchased from Oakwood and used as received.
Dodecylbenzensulfonyl azide (soft type, mixture) was purchased from Combi-blocks and used as received.
Ethyl 2-(diethoxyphosphoryl)acetate was purchased from Oakwood and used as received.
Hydroxytetrachlorophthalimide was purchased from Sigma Aldrich and used as received.
Iodobenzene was purchased from Sigma Aldrich and filtered through a pad of silica before using.
Isobutylchloroformate was purchased from Alfa Aesar and used as received.
Lithium aluminium hydride ($LiAlH_4$) was purchased from Sigma Aldrich and used as received.
Lithium bis(trimethylsilyl)amide (LHMDS) was purchased from Sigma Aldrich and used as received.
Methyltriphenylphosphonium bromide was purchased from Ambeed and used as received.
n-BuLi (2.1 M in Hexanes) was purchased from Sigma Aldrich and used as received.
$NiCl_2$.DME was purchased from Strem and used as received.
Oxalyl chloride was purchased from Oakwood and used as received.
Phenyl magnesium bromide was purchased from Sigma Aldrich and used as received.
Potassium carbonate (K_2CO_3) was purchased from EMD and used as received.
4-iodo-2-methoxy Pyridine was purchased from Sigma Aldrich and filtered through a pad of silica before using.
Sodium borohydride was purchased from Sigma Aldrich and used as received.
Sodium chlorite was purchased from Alfa Aesar and used as received.
Sodium cyanoborohydride was purchased from Oakwood and used as received.
Sodium dihydrogen phosphate was purchased from Macron Chemicals and used as received.
Sodium hydride (NaH) was purchased from Sigma Aldrich and used as received.
Sodium hydrogen carbonate was purchased from Fisher Chemical and used as received.
Tert-BuOH was purchased from Oakwood and used as received.
Tetrabutylammonium fluoride (TBAF) was purchased from Sigma Aldrich and used as received.
Thiophene-2-carbaldehyde was purchased from Sigma Aldrich and used as received.

Triethylamine was purchased from Sigma Aldrich and distilled over CaH2 before use.
Tris(dibenzylideneacetone)dipalladium(0) (Pd_2dba_3) was purchased from Strem and used as received.
XPhos was purchased from Sigma Aldrich and used as received.
Zinc chloride was purchased from Alfa Aesar and used as received.

3. [2+2]-cycloaddition reaction

3.1. General procedure A: Starting material synthesis.

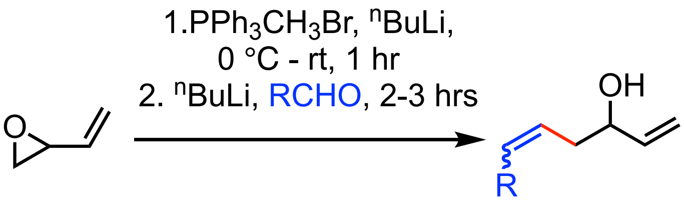

To a flame dried 100 ml round bottom flask equipped with a stir bar was added the Methyltriphenylphosphonium bromide (1.0 equiv.) and the flask was evacuated and backfilled with N_2(*3). Next, it was dissolved in THF (0.5 M) and cooled to 0 °C. To this the $^{n-}$BuLi (2.1 M in Hexanes, 1.2 equiv.) was added dropwise over 10 mins and the reaction was stirred for 20 minutes at 0 °C. Following this stirring period, butadiene monoxide (1.4 equiv.) was added dropwise and the reaction was bought to room temperature and stirred for 1 hour. Next, the reaction was cooled to -35 °C and to this $^{n-}$BuLi (2.1 M in Hexanes, 1.0 equiv.) was added dropwise. The reaction mixture became dark red in colour. Lastly, to this dark red solution, the aldehyde (1.0 equiv.) was added dropwise , the solution was slowly warmed to room temperature and allowed to stir for 2-3 hours. The disappearance of the aldehyde was confirmed by thin layer chromatography, and the reaction was quenched with water. Next, the aqueous layer was extracted with 20 ml CH_2Cl_2 (*3) and the combined organic layer was dried over anh. Na_2SO_4, organic layer was filtered and concentrated in *vacuo*. Next, the crude material was purified by MPLC to afford the alcohol as E/Z mixture.

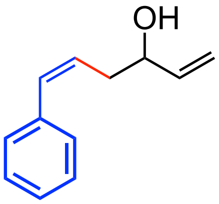

SI-1

6-phenylhexa-1,5-dien-3-ol (SI-1): This compound was prepared according to the general procedure A using benzaldehyde and purified by MPLC (gradient 4-5% EtOAc in Hex) to isolate **SI-1** as a colourless oil. Spectral data matched with literature report.[i]

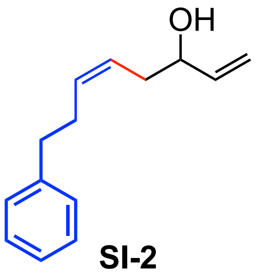

SI-2

8-phenylocta-1,5-dien-3-ol (SI-2): This compound was prepared according to the general procedure A using using 3-phenylpropanal and purified by MPLC (gradient 4-5% EtOAc in Hex) to isolate **SI-2** as a colourless oil. Spectral data matched with literature report. [I]

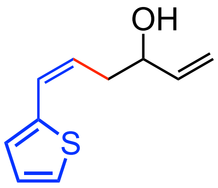

SI-3

6-(thiophen-2-yl)hexa-1,5-dien-3-ol (SI-3): This compound was prepared according to the general procedure A using thiophene-2-carbaldehyde (314 mg, 259 µL, 2.8 mmol) and purified by MPLC (gradient 4-5% EtOAc in Hex) to isolate **SI-3** as a yellow oil (290 mg, 1.61 mmol, 58 %) **R$_f$**: 0.4 in 10% EA/Hex.

^{1}H NMR (400 MHz, CDCl$_3$) δ 7.12 (d, *J* = 5.1 Hz, 1H), 6.99 – 6.89 (m, 2H), 6.68 – 6.54 (m, 1H), 6.05 (dt, *J* = 15.6, 7.4 Hz, 1H), 5.93 (ddd, *J* = 17.3, 10.5, 5.8 Hz, 1H), 5.29 (dt, *J* = 17.2, 1.5 Hz, 1H), 5.16 (dt, *J* = 10.5, 1.4 Hz, 1H), 4.29 – 4.18 (m, 1H), 2.52 – 2.37 (m, 2H).

^{13}C NMR (126 MHz, CDCl$_3$) δ 142.5, 140.3, 127.4, 126.5, 125.5, 125.1, 123.8, 115.2, 72.3, 40.8.

IR (neat): 3365 (br), 2982 (w), 1423 (w).

HRMS (EI, *m/z*): Calculated for C_{10} H_{12} O S; $[M]^+$ 180.0609; found 180.0605.

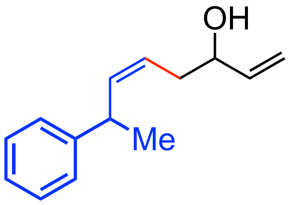

SI-4

7-phenylocta-1,5-dien-3-ol (SI-4): This compound was prepared according to the general procedure A using 2-phenylpropanal and purified by MPLC (gradient 4-5% EtOAc in Hex) to isolate **SI-4** a colourless oil (480 mg, 2.37 mmol, 42 %).

R_f: 0.5 in 10% EA/Hex.

^{1}H NMR (500 MHz, $CDCl_3$) δ 7.30 (dd, *J* = 8.2, 7.0 Hz, 2H), 7.23 – 7.17 (m, 3H), 5.88 (ddd, *J* = 17.3, 10.5, 5.8 Hz, 1H), 5.75 (ddt, *J* = 15.4, 6.8, 1.4 Hz, 1H), 5.54 – 5.42 (m, 1H), 5.25 (dq, *J* = 17.2, 1.7 Hz, 1H), 5.12 (dq, *J* = 10.5, 1.6 Hz, 1H), 4.14 (ddt, *J* = 7.1, 3.2, 1.4 Hz, 1H), 3.47 (t, *J* = 7.0 Hz, 1H), 2.37 – 2.20 (m, 2H), 1.36 (d, *J* = 7.0 Hz, 3H).

^{13}C NMR (126 MHz, $CDCl_3$) δ 146.1, 140.5, 139.6, 139.5, 128.6, 127.3, 127.0, 126.2, 124.1, 114.9, 72.3, 42.5, 40.6, 21.5.

IR (neat): 3363 (br), 2965 (w), 2871 (w), 1451 (m).

HRMS (ESI): Calculated for $C_{14}H_{18}ONa$ $[M+Na]^+$ 225.1250; found 225.1250.

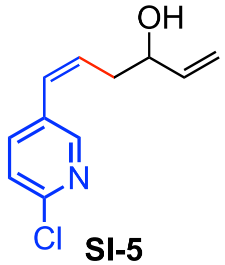

SI-5

6-(6-chloropyridin-3-yl)hexa-1,5-dien-3-ol (SI-5): This compound was prepared according to the general procedure A using 6-chloronicotinaldehyde and purified MPLC (gradient 40-50% EtOAc in Hex) to isolate **SI-5** (as a mixture of diastereomers) as a colourless oil (1.26 g, 6.01 mmol, 60 %).

R_f: 0.5 in 60% EA/Hex.

^{1}H NMR (500 MHz, $CDCl_3$) δ 8.31 (d, *J* = 2.6 Hz, 1H), 7.64 (dd, *J* = 8.3, 2.5 Hz, 1H), 7.27 – 7.23 (m, 1H), 6.43 (d, *J* = 16.0 Hz, 1H), 6.30 (dt, *J* = 15.9, 7.1 Hz, 1H), 5.97 – 5.89 (m, 1H), 5.29 (dt, *J* = 17.2, 1.5 Hz, 1H), 5.17 (dt, *J* = 10.5, 1.3 Hz, 1H), 4.28 (s, 1H), 2.58 – 2.42 (m, 2H).

^{13}**C NMR (126 MHz, CDCl$_3$)** δ 149.9 (minor), 149.8 (minor), 147.9, 140.3, 140.2 (minor), 138.7 (minor), 135.5, 132.1, 131.9 (minor), 131.1 (minor), 129.4, 128.2, 126.7 (minor), 124.2, 123.9 (minor), 115.7 (minor), 115.5, 72.6 (minor), 72.4, 40.9, 36.1 (minor).

IR (neat): 3366 (br), 2901 (w), 1582 (w), 1461 (s).

HRMS (ESI): Calculated for C_{11} H_{13} O N Cl [M+H]+ 210.0680; found 210.0681.

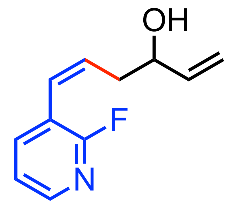

SI-6

6-(2-fluoropyridin-3-yl)hexa-1,5-dien-3-ol (SI-6): This compound was prepared according to the general procedure A using 2-Fluoropyridine-3-carboxyaldehyde and purified by MPLC (gradient 40-50% EtOAc in Hex) to afford **SI-6** (as a mixture of diastereomers) as a yellowish white oil (400 mg, 2.07 mmol, 74 %).

R_f: 0.5 in 70% EA/Hex.

^{1}**H NMR (400 MHz, CDCl$_3$)** δ 8.15 – 7.99 (m, 1H), 7.80 (tdd, *J* = 10.0, 7.3, 1.9 Hz, 1H), 7.14 (dddd, *J* = 9.5, 6.9, 4.9, 1.8 Hz, 1H), 6.58 – 6.44 (m, 1H), 6.39 (dt, *J* = 16.2, 7.1 Hz, 1H), 6.01 – 5.82 (m, 1H), 5.34 – 5.23 (m, 1H), 5.16 (ddq, *J* = 9.6, 5.5, 1.3 Hz, 1H), 4.28 (t, *J* = 6.6 Hz, 1H), 2.59 – 2.42 (m, 2H).

^{13}**C NMR (126 MHz, CDCl$_3$)** δ 161.8 (*J* = 240 Hz) (minor), 159.9, (*J* = 242 Hz), 146.1 (*J* = 14 Hz) (minor), 145.8 (*J* = 16 Hz), 140.7 (*J* = 4 Hz) (minor), 140.7, 140.3, 140.2 (minor), 137.3 (*J* = 5 Hz), 131.9 (minor), 131.2, 124.3, 122.8 (minor), 121.7 (*J* = 4 Hz), 121.2 (*J* = 5 Hz) (minor), 120.4 (J = 28 Hz), 119.9 (*J* = 29 Hz) (minor), 115.6 (minor), 115.4, 72.5 (minor), 72.4, 41.2, 36.3 (minor).

IR (neat): 3384 (br), 2909 (w), 1737 (m), 1432 (m), 1241 (m).

HRMS (ESI): Calculated for C_{11} H_{13} ONF [M+H]+ 194.0976; found 194.0976.

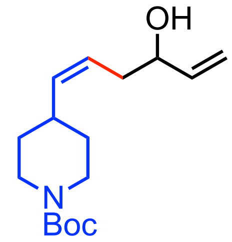

SI-7

***tert*-butyl-4-(4-hydroxyhexa-1,5-dien-1-yl)piperidine-1-carboxylate (SI-7):** This compound was prepared according to the general procedure A using 1-Piperidinecarboxylic acid, 4-formyl-, 1,1-dimethylethyl ester and purified by MPLC (gradient 20-25% EtOAc in Hex) to afford **SI-7** as a colourless oil (1.26 g, 4.48 mmol, 80 %) Note: Mixture with 10% starting aldehyde.

R_f: 0.5 in 30% EA/Hex.

^{1}H NMR (500 MHz, $CDCl_3$) δ 5.86 (ddd, *J* = 16.7, 10.4, 5.7 Hz, 1H), 5.50 (dd, *J* = 15.5, 6.4 Hz, 1H), 5.46 – 5.31 (m, 1H), 5.31 – 5.17 (m, 1H), 5.12 (tt, *J* = 10.1, 1.4 Hz, 1H), 4.21 – 3.92 (m, 3H), 2.72 (s, 2H), 2.55 – 2.02 (m, 3H), 1.73 – 1.59 (m, 2H), 1.45 (s, 11H), 1.34 – 1.20 (m, 2H).

^{13}C NMR (126 MHz, $CDCl_3$) δ 155.0, 140.5, 138.7, 124.01 114.8, 79.4, 72.5, 72.2, 40.6, 39.1, 35.4, 34.6, 32.0, 28.6.

IR (neat): 3439 (br), 2976 (m), 1693 (s), 1672 (s), 1426 (m).

HRMS (ESI): Calculated for C_{16} H_{27} O_3 N Na 304.1883; Found 304.1886.

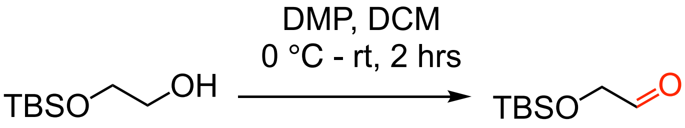

8

2-((tert-butyldimethylsilyl)oxy)acetaldehyde (8): To a flame dried 1000 ml round bottom flask equipped with a stir bar was added the DMP (69.7 g, 1.01 equiv., 164 mmol) and the flask was evacuated and backfilled with N_2(*3). Next, DMP was dissolved in CH_2Cl_2 (0.1 M, 500 ml) and cooled to 0 °C. To this, 2-((tert-butyldimethylsilyl)oxy)ethan-1-ol (28.7 g, 493 mL, 0.33 molar, 1 equiv., 163 mmol) was added dropwise and the reaction was bought to room temperature and stirred. The disappearance of the starting material was confirmed by thin layer Chromatography,

and the reaction was quenched with sat $Na_2S_2O_3$ solution. Next, the aqueous layer was extracted with CH_2Cl_2 (*3) and the combined organic layers were dried over Na_2SO_4, filtered and evaporated in *vacuo*. The crude mixture was passed through a pad of silica to remove by-product from DMP. The filtrate was evaporated in *vacuo* to afford **8** (26.90 g, 154 mmol, 95 %) which was directly used in the next step. Spectral data matched with literature report.[ii]

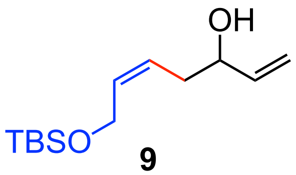

9

7-((*tert*-butyldimethylsilyl)oxy)hepta-1,5-dien-3-ol (9): This compound was prepared according to the general procedure A using -((tert-butyldimethylsilyl)oxy)acetaldehyde (5.85 g, 33.57 mmol)and purified by MPLC (gradient 4-5% EtOAc in Hex) to afford **9** (as a mixture of diastereomers) as a colourless oil (5.00 g, 0.02 mol, 40 %).

R_f: 0.5 in 10% EA/Hex.

^{1}H NMR (500 MHz, CDCl$_3$) δ 5.89 (td, *J* = 10.8, 5.3 Hz, 1H), 5.78-5.51 (m,2H), 5.30 – 5.22 (m, 1H), 5.20 – 5.09 (m, 1H), 4.24 – 4.13 (m, 3H), 2.39 – 2.22 (m, 2H), 0.95 – 0.86 (m, 9H), 0.10 – 0.06 (m, 6H).

^{13}C NMR (126 MHz, CDCl$_3$) δ 140.6 (minor), 140.5, 133.3, 132.4 (minor), 126.8 (minor), 126.1, 114.9, 114.9 (minor), 72.2, 72.0 (minor), 63.8, 59.2 (minor), 40.2, 35.5 (minor), 26.1, 25.8 (minor), 18.5, 18.5 (minor), -3.5 (minor), -5.0.

IR (neat): 3400 (br), 2955 (m), 2857 (w), 1254 (w).

HRMS (ESI): Calculated for $C_{13}H_{24}O_2NaSi$ $[M-H_2+Na]^+$ 263.1438; found 263.1439.

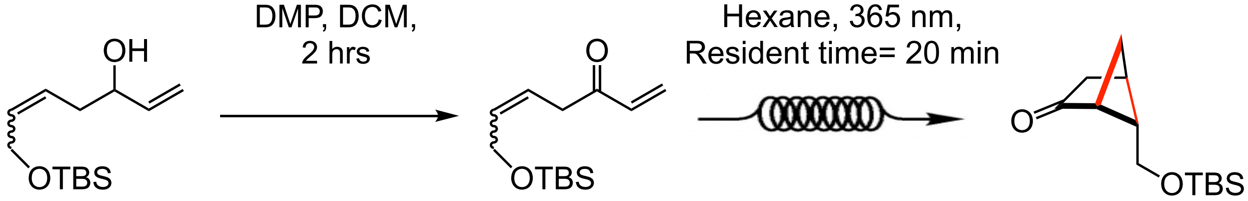

9 **SI-8** **10**

5-(((*tert*-butyldimethylsilyl)oxy)methyl)bicyclo[2.1.1]hexan-2-one (10): To a flame-dried 250 ml round bottom flask equipped with a stir bar was added DMP (12.86 g, 1.5 equiv., 30.32 mmol) and the flask was evacuated and backfilled with N_2(*3). Next, DMP was dissolved in CH_2Cl_2 (100 ml) and cooled to 0 °C. To this, 7-((tert-butyldimethylsilyl)oxy)hepta-1,5-dien-3-ol (4.9 g, 1.0 equiv., 20.21 mmol) was added dropwise and the reaction was bought to room temperature and stirred for 2 hrs. The disappearance of the starting material was confirmed by a thin layer of chromatography, and the reaction was quenched with water. The aqueous layer was extracted with 30 ml CH_2Cl_2 (*3) and the combined organic layer was dried over anh. Na_2SO_4. Next, the organic layer was filtered and concentrated in *vacuo*. The crude material was passed through a silica plug to remove the solid by product from the DMP.

Next, to a flame dried 250 ml round bottom flask was added the crude product **SI-8** and the flask was evacuated and backfilled with N_2(*3). It was dissolved in Hexane (300 ml, 0.1 M) and pumped through Vapourtec easy Photochem flow reactor with 20 min resident time (flow rate 0.5 ml/min). After that, 20 ml of hexane was pumped through the flow reactor to pump through the material. The solvent was removed in *vacuo* and the crude material was purified via MPLC (gradient 4-5% EtOAc in Hex) to afford **10** as a colourless oil (3.30 g, 14.14 mmol, 70 %, dr 2:1).

R_f: 0.5 in 10% EA/Hex.

^{1}H NMR (400 MHz, $CDCl_3$) δ 3.99 (d, *J* = 7.7 Hz, 0.8H), 3.43 (qd, *J* = 11.0, 7.0 Hz, 2H), 2.83 (q, *J* = 2.8 Hz, 2H), 2.78 – 2.64 (m, 3H) (minor), 2.53 (ddt, *J* = 11.2, 6.0, 2.9 Hz, 1H), 2.29 – 2.12 (m, 3 minor+1 major H), 2.07 – 1.99 (m, 2H), 1.65 (dd, *J* = 8.1, 6.6 Hz, 0.4H) (minor), 1.54 (d, *J* = 7.2 Hz, 1H), 0.90 (s, 4H) (minor), 0.87 (s, 9H), 0.07 (s, 3H) (minor), 0.02 (d, *J* = 1.3 Hz, 6H).

^{13}C NMR (126 MHz, $CDCl_3$) δ 213.9, 212.9, 60.5, 60.4, 57.4, 57.1, 55.2, 52.1, 43.6, 38.6, 37.6, 37.2, 36.9, 36.2, 25.9, 25.9, 18.4, 18.3, -5.3 (d).

IR (neat): 2955 (w), 2857 (w), 1760 (s), 1255 (m).

HRMS (ESI): Calculated for C_{13} H_{24} O_2 Na Si [M+Na] 263.1438; found 263.1439.

3.2. General procedure B: Photochemical [2+2]-cycloaddition.

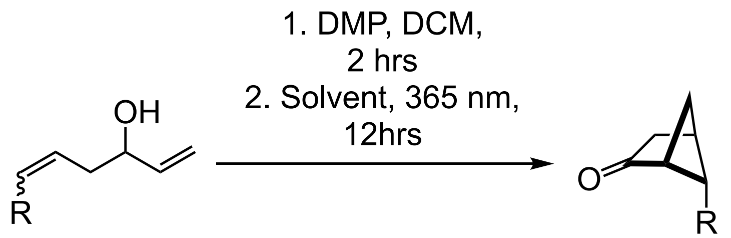

To a flame dried 100 ml round bottom flask equipped with a stir bar was added the DMP (1.2 equiv.,) and the flask was evacuated and backfilled with N_2(*3). Next, DMP was dissolved in CH_2Cl_2 (0.1 M) and cooled to 0 °C. To this, alcohol in CH_2Cl_2 (0.3M) was added dropwise and the reaction was bought to room temperature and stirred. The disappearance of the starting material was confirmed by thin layer Chromatography, and the reaction was quenched with water. The aqueous layer was extracted with 10 ml CH_2Cl_2 (*3) and the combined organic layer was dried over anh. Na_2SO_4. Next, the organic layer was filtered and concentrated in *vacuo*. The crude material was passed through a pad of silica to remove the solid byproduct from the DMP and directly used in the next step.

Next, to a flame dried 6-dram vial equipped with a stir bar was added the ketone (1 equiv..) and the flask was backfilled with N_2(*3). It was dissolved in MeCN (0.1 M) and irradiated with 365 nm LED light for 16 hrs. Next, the solvent was removed in *vacuo* and the crude material was purified by MPLC.

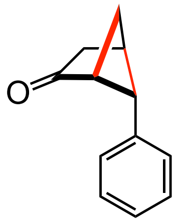

11

5-phenylbicyclo[2.1.1]hexan-2-one (11): This compound was synthesized according to general procedure B using (Z)-6-phenylhexa-1,5-dien-3-one (100 mg, 0.58 mmol) and purified using MPLC (gradient 4-5% EtOAc in Hex) to isolate **11** as a colourless oil (61 mg, 0.35 mmol, 61 %, 3:1 dr)

R_f: 0.5 in 10% EA/Hex.

^{1}H NMR (500 MHz, CDCl$_3$) δ 7.38 (t, *J* = 7.6 Hz, 0.7H) (minor), 7.33 – 7.26 (m, 2H), 7.23 – 7.15 (m, 1H), 7.03 (dt, *J* = 8.1, 1.1 Hz, 2H), 3.70 (q, *J* = 3.2 Hz, 1H), 3.34 (d, *J* = 7.4 Hz, 0.3H) (minor), 3.29 (dt, *J* = 6.8, 2.6 Hz, 1H), 3.16 (dtd, *J* = 7.0, 3.6, 1.7 Hz, 1H), 3.13 – 3.07 (m, 2H) (minor), 2.62 (dp, *J* = 8.8, 3.0 Hz, 0.3H) (minor), 2.45 – 2.40 (m, 2H) (minor), 2.27 – 2.21 (m, 1H), 1.99 (ddt, *J* = 16.1, 2.7, 1.2 Hz, 1H), 1.86 (ddd, *J* = 16.2, 4.9, 1.3 Hz, 1H), 1.71 (t, *J* = 7.6 Hz, 0.3H) (minor), 1.68 (d, *J* = 7.3 Hz, 1H).

^{13}C NMR (126 MHz, CDCl$_3$) δ 214.0, 213.4 (minor), 138.6, 137.8 (minor), 128.7 (minor), 128.6, 128.3 (minor), 127.0, 126.9 (minor), 126.7, 60.2 (minor), 58.6, 56.1 (minor), 53.6, 43.3 (minor), 39.2, 39.0 (minor), 38.5 (minor), 37.9, 37.2.

IR (neat): 2989 (w), 1755 (s), 1497 (w), 1168 (w).

HRMS (ESI): Calculated for $C_{12}H_{13}O$ $[M+H]^+$ 173.0961; found 173.0961.

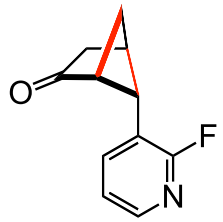

12

5-(2-fluoropyridin-3-yl)bicyclo[2.1.1]hexan-2-one (12): This compound was synthesized according to general procedure B using (Z)-6-(2-fluoropyridin-3-yl)hexa-1,5-dien-3-one (150 mg, 0.78 mmol) and purified using MPLC (gradient 40-50% EtOAc in Hex) to isolate **12** as a yellow solid as single diastereomer (54 mg, 0.28 mmol, 36 %, NMR yield 52%).

R$_f$: 0.5 in 80% EA/Hex.

^{1}H NMR (500 MHz, CDCl$_3$) δ 8.06 (d, *J* = 4.9 Hz, 1H), 7.45 – 7.35 (m, 1H), 7.12 – 7.03 (m, 1H), 3.60 (d, *J* = 4.0 Hz, 1H), 3.28 (m, 2H), 2.29 (td, *J* = 4.9, 2.3 Hz, 1H), 2.11 – 2.01 (m, 1H), 1.77 (dd, *J* = 16.5, 5.0 Hz, 1H), 1.69 (d, *J* = 7.4 Hz, 1H).

^{13}C NMR (126 MHz, CDCl$_3$) δ 213.1, 162.6 (d, J = 50 Hz), 146.4 (d, J = 55 Hz), 134.0 (d, J = 20 Hz), 121.5 (d, J= 12 Hz) 120.6 (d, J= 125 Hz), 57.9, 48.5, 48.5, 39.4, 39.4, 38.4, 37.2.

IR (neat): 2998 (w), 1752 (s), 1604 (w), 1573 (w), 1430 (s), 1243 (w).

HRMS (ESI): Calculated for C_{11} H_{11} ONF $[M+H]^+$ 192.0819; found 192.0820.

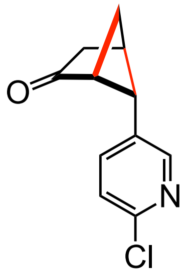

13

5-(6-chloropyridin-3-yl)bicyclo[2.1.1]hexan-2-one (13): This compound was synthesized according to general procedure B using (Z)-6-(6-chloropyridin-3-yl)hexa-1,5-dien-3-one (100 mg, 0.48 mmol) and purified using MPLC (gradient 40-50% EtOAc in Hex) to isolate **13** as a yellow solid (48 mg, 0.23 mmol, 48 %, 3:1 dr).

R_f: 0.5 in 80% EA/Hex.

^{1}H NMR (500 MHz, $CDCl_3$) δ 8.35 (dd, J = 2.5, 1.0 Hz, 0.3H) (minor), 8.15 – 7.97 (m, 1H), 7.58 (ddd, J = 8.2, 2.6, 0.9 Hz, 0.3H) (minor), 7.34 (dd, J = 8.2, 0.7 Hz, 0.3H) (minor), 7.30 (ddd, J = 8.2, 2.6, 0.9 Hz, 1H), 7.22 (dd, J = 8.2, 0.8 Hz, 1H), 3.64 (q, J = 3.0 Hz, 1H), 3.34 – 3.24 (m, 2H), 3.20 (dtd, J = 7.0, 3.6, 1.8 Hz, 1H), 3.13 – 3.04 (m, 0.7H) (minor), 2.56 – 2.49 (m, 0.3H) (minor), 2.47 – 2.40 (m, 0.7H) (minor), 2.29 (ddtd, J = 6.0, 4.7, 2.6, 1.3 Hz, 1H), 2.07 (ddt, J = 16.5, 2.7, 1.2 Hz, 1H), 1.82 (ddd, J = 16.4, 4.9, 1.2 Hz, 1H), 1.75 (dd, J = 8.2, 7.3 Hz, 1H), 1.72 (d, J = 7.4 Hz, 1H).

^{13}C NMR (126 MHz, $CDCl_3$) δ 212.4, 211.6 (minor), 150.1 (minor), 150.1, 149.5, 148.6, 138.8 (minor), 137.6, 133.0, 132.4 (minor), 124.3 (minor), 124.2, 59.5 (minor), 58.4, 53.1 (minor), 50.41, 42.9 (minor), 39.2, 38.8 (minor), 38.2, 38.2 (minor), 37.0.

IR (neat): 2994 (w), 2897 (w), 1759 (w), 1560 (w), 1462 (m), 1140 (m).

HRMS (EI, *m/z*): Calculated for C_{11} H_{10} Cl N O $[M]^+$ 207.0451; found 207.0449.

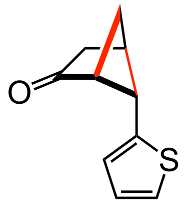

14

5-(thiophen-2-yl)bicyclo[2.1.1]hexan-2-one (14): This compound was synthesized according to general procedure B using (Z)-6-(thiophen-2-yl)hexa-1,5-dien-3-one (62 mg, 0.35 mmol) and purified using MPLC (gradient 4-5% EtOAc in Hex) to isolate **14** as a colourless oil (13.5 mg, 0.076 mmol, 22 %, 3:1 dr)

R_f: 0.5 in 10% EA/Hex.

^{1}H NMR (600 MHz, $CDCl_3$) δ 7.25 (d, *J* = 5.4 Hz, 0.6H) (minor), 7.16 (d, *J* = 5.1 Hz, 1H), 7.01 (dd, *J* = 5.1, 3.5 Hz, 0.3 H) (minor), 6.96 (dd, *J* = 3.1, 1.6 Hz, 0.3H) (minor), 6.87 (dd, *J* = 5.1, 3.5 Hz, 1H), 6.68 (dd, *J* = 3.1, 1.5 Hz, 1H), 3.75 (d, *J* = 3.8 Hz, 1H), 3.41 (dd, *J* = 7.2, 1.3 Hz, 0.3H) (minor), 3.22 (dt, *J* = 6.8, 2.6 Hz, 1H), 3.09 – 3.05 (m, 1H), 3.05 – 3.00 (m, 0.3H) (minor), 2.82 (dt, *J* = 8.2, 3.0 Hz, 0.3H), 2.39 (dd, *J* = 2.9, 1.4 Hz, 0.7H) (minor), 2.25 (ddt, *J* = 5.6, 3.7, 1.8 Hz, 1H), 2.06 (dd, *J* = 4.0, 1.6 Hz, 2H), 1.75 (t, *J* = 7.6 Hz, 0.3H) (minor), 1.66 (d, *J* = 7.4 Hz, 1H).

^{13}C NMR (126 MHz, $CDCl_3$) δ 213.5, 212.3 (minor), 141.6 (minor), 141.3, 127.0, 125.7 (minor), 125.7(minor), 124.7, 61.4 (minor), 59.7, 51.9 (minor), 49.4, 43.0 (minor), 40.8 (minor), 40.5, 39.0 (minor), 38.5, 37.0.

IR (neat): 2970 (w), 1758(s), 1365 (w), 1231 (w).

HRMS (EI, *m/z*): Calculated for $C_{10}H_{10}OS$ $[M]^+$ 178.0452; found 178.0451.

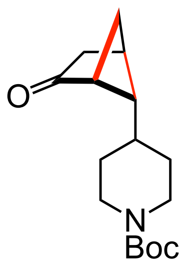

15

tert-butyl 4-(2-oxobicyclo[2.1.1]hexan-5-yl)piperidine-1-carboxylate (15): This compound was synthesized according to general procedure B using tert-butyl (Z)-4-(4-oxohexa-1,5-dien-1-yl)piperidine-1-carboxylate (100 mg, 0.36 mmol) and purified using MPLC (gradient 20-30% EtOAc in Hex) to as isolate **15** a brown solid (75 mg, 0.27 mmol, 75 %, 2:1 dr).

R_f: 0.5 in 70% EA/Hex.

^{1}H NMR (600 MHz, $CDCl_3$) δ 4.06 (s, 3H), 2.85 (dt, *J* = 7.0, 2.5 Hz, 1H), 2.80 – 2.75 (m, 2H), 2.70 – 2.64 (m, 1H), 2.60 (s, 2H), 2.36 – 2.23 (m, 0.5H) (minor), 2.23 – 2.15 (m, 0.5 H) (minor), 2.10 – 2.03 (m, 3H), 2.03 – 1.96 (m, 2H), 1.96 – 1.90 (m, 1H), 1.78 (d, *J* = 13.2 Hz, 0.5 H) (minor), 1.69 – 1.64 (m, 2H), 1.59 – 1.53 (m, 3H), 1.46 (s, 5H) (minor), 1.45 (s, 9H), 1.21 – 1.13 (m, 1H), 1.13 – 1.04 (m, 2H), 0.99 (qd, *J* = 13.0, 5.2 Hz, 1H).

^{13}C NMR (126 MHz, $CDCl_3$) δ 214.6, 213.4 (minor), 155.0 (minor), 154.9, 79.6 (minor), 79.5, 59.6 (minor), 57.8 (minor), 57.6, 56.5, 44.1 (minor), 38.0, 37.9, 37.0, 36.6, 36.6, 34.3, 33.3 (minor), 29.6, 29.5, 28.6.

IR (neat): 2976 (w), 2861 (w), 1755 (s), 1693 (s), 1450 (m), 1217 (m), 1162 (m).

HRMS (ESI): Calculated for $C_{16}H_{25}O_3NNa$ $[M+Na]^+$ 302.1727; found 302.1729.

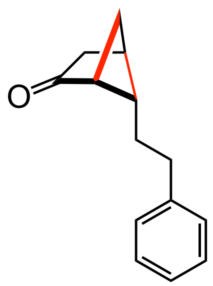

16

5-phenethylbicyclo[2.1.1]hexan-2-one (16): This compound was synthesized according to general procedure B using (Z)-8-phenylocta-1,5-dien-3-one (100 mg, 0.50 mmol) and purified using MPLC (gradient 4-5% EtOAc in Hex) to isolate **16** as a colourless oil (71 mg, 0.36 mmol, 71 %, 4.5:1 dr). Note: dr was obtained from the GC analysis of the isolated product.

R_f: 0.5 in 10% EA/Hex.

^{1}H NMR (500 MHz, $CDCl_3$) δ 7.30 – 7.22 (m, 3H), 7.18 – 7.13 (m, 2H), 7.13 – 7.08 (m, 2H), 2.74 (dt, *J* = 6.8, 2.6 Hz, 1H), 2.73 – 2.67 (m, 0.6H) (minor), 2.64 (tdd, *J* = 8.3, 5.5, 3.2 Hz, 2H), 2.57 – 2.45 (m, 3H), 2.32 (tq, *J* = 7.8, 2.9 Hz, 1H), 2.23 (dt, *J* = 16.1, 1.3 Hz, 0.6H) (minor), 2.15 (ddd, *J* = 15.9, 4.7, 1.5 Hz, 0.6H), 2.10 – 1.95 (m, 4H), 1.94 – 1.89 (m, 1H), 1.61 (dd, *J* = 8.1, 6.4 Hz, 0.6H) (minor), 1.54 – 1.47 (m, 3H).

^{13}C NMR (126 MHz, $CDCl_3$) δ 214.9, 213.7 (minor), 141.7 (minor), 141.4, 128.6 (minor), 128.5, 128.4, 126.1 (minor), 126.1, 58.9 (minor), 58.8, 53.6 (minor), 50.8, 43.8 (minor), 38.5, 37.9 (minor), 37.7, 37.6 (minor), 36.9, 34.8 (minor), 33.8, 29.2, 28.8 (minor).

IR (neat): 2994 (w), 2897 (w), 1754 (s), 1603 (w), 1496 (w).

HRMS (EI, *m/z*): Calculated for $C_{14} H_{16} O$ $[M]^+$ 200.1206; found 200.1201.

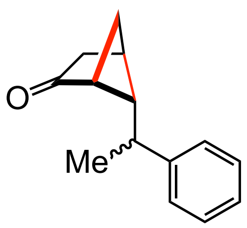

17

5-(1-phenylethyl)bicyclo[2.1.1]hexan-2-one (17): This compound was synthesized according to general procedure B using (Z)-7-phenylocta-1,5-dien-3-one (100 mg, 0.49 mmol) and purified using MPLC (gradient 4-5% EtOAc in Hex) to isolate **17** as a colourless oil (71 mg, 0.35 mmol, 71 %, 4.5:3.1:1.4:1 dr). Note: dr was obtained from the GC analysis of the isolated product.

R_f: 0.5 in 10% EA/Hex.

^{1}H NMR (500 MHz, $CDCl_3$) δ 7.36 – 7.26 (m, 5H), 7.25 – 7.09 (m, 7H), 3.28 (tt, *J* = 12.9, 6.7 Hz, 0.6H), 2.96 (dt, *J* = 6.8, 2.6 Hz, 1H), 2.89 – 2.81 (m, 1.8H), 2.75 (ddd, *J* = 7.1, 3.4, 1.7 Hz, 0.3H), 2.54 (dt, *J* = 6.8, 2.6 Hz, 1H), 2.52 – 2.48 (m, 1.6H), 2.48 – 2.40 (m, 2H), 2.31 (td, *J* = 4.6, 2.4 Hz, 1H), 2.30 – 2.27 (m, 0.5H), 2.23 (ddd, *J* = 10.3, 5.8, 1.4 Hz, 1H), 2.21 – 2.18 (m, 1H), 2.15 – 2.06 (m, 2H), 2.01 (ddt, *J* = 16.2, 2.6, 1.2 Hz, 1.9H), 1.96 – 1.90 (m, 1H), 1.87 (ddddd, *J* = 7.3, 4.7, 3.5, 2.6, 1.1 Hz, 0.89H), 1.67 (ddd, *J* = 8.3, 6.5, 2.0 Hz, 0.67H), 1.57 – 1.52 (m, 2.3H), 1.35 (d, *J* = 6.9 Hz, 0.8H), 1.29 (d, *J* = 6.9 Hz, 1H), 1.22 (d, *J* = 6.9 Hz, 2.6H), 1.16 (d, *J* = 6.7 Hz, 3H).

^{13}C NMR (126 MHz, $CDCl_3$) δ 214.8, 214.7, 213.4, 213.3, 145.5, 145.3, 144.5, 143.9, 128.7, 128.7, 128.7, 128.6, 127.1, 127.0, 127.0, 126.8, 126.6, 126.6, 126.5, 126.5, 61.3, 61.0, 58.8, 58.5, 58.5, 58.3, 58.2, 58.1, 44.3, 44.1, 37.9, 37.7, 37.7, 37.6, 37.5, 37.5, 37.4, 37.3, 37.2, 37.0, 37.0, 36.8, 21.3, 20.7, 19.7, 18.3.

IR (neat): 2961 (w), 1757 (s), 1426 (w).

HRMS (EI, *m/z*): Calculated for $C_{14}H_{16}O$; $[M]^+$ 200.1201; found 200.1209.

3.3. Substrate evaluation and mechanism for cross [2+2]-cycloaddition reaction.

A) Substrates Evaluated and Mechanism

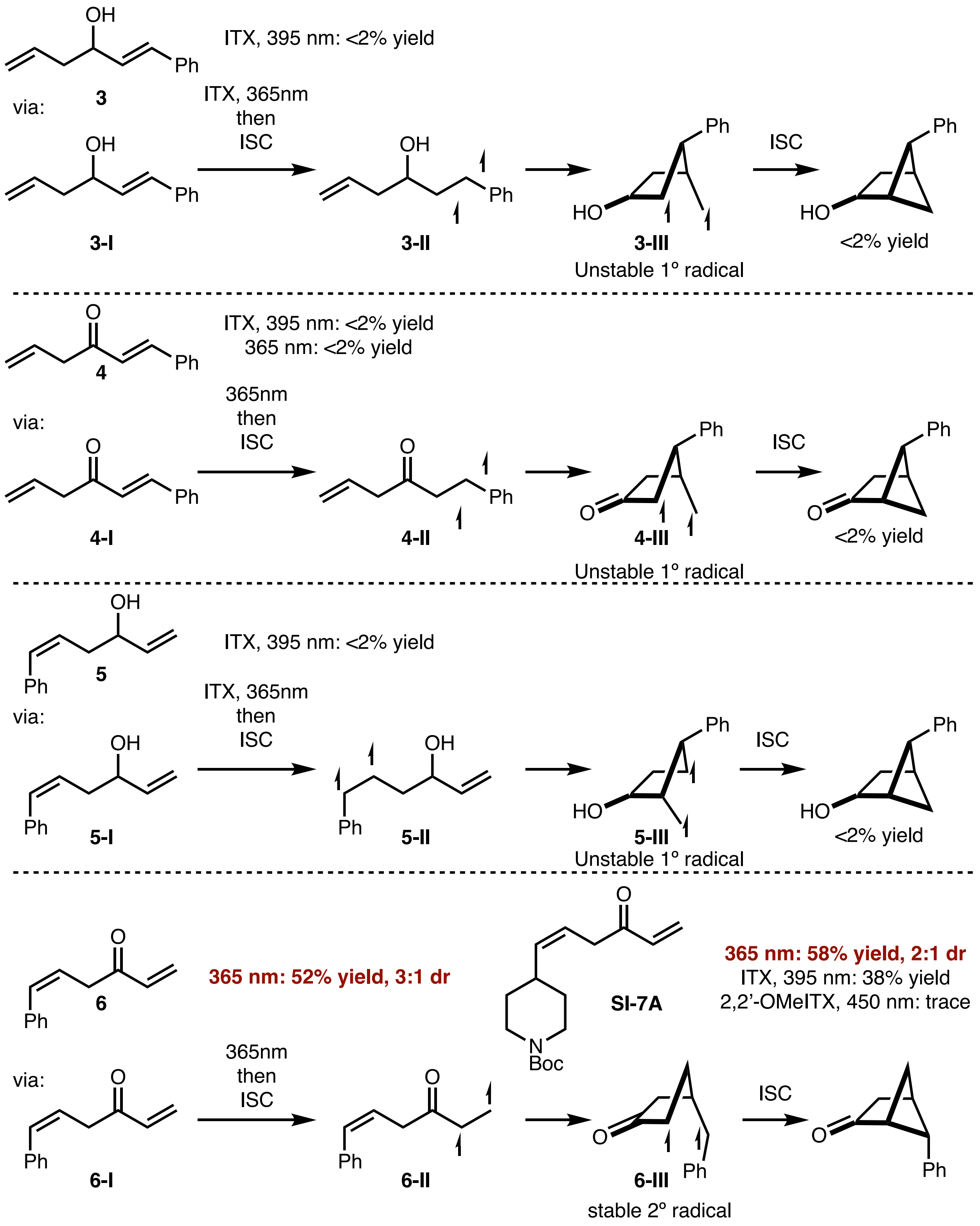

Different substitution pattern on 1,5-hexadiene system were evaluated both under direct excitation and photo-sensitization conditions. Based on recent literature ITX(I-Prthioxanthone) was chosen as suitable photosensitizer to sensitize alkenyl arenes due to exergonic in triplet energy.[iii] However, after sensitization or direct excitation of substrate **3**, **4** and **5**, the initial cyclization generates bi-radical intermediate **3-5III**, which contains at least one primary radical. Therefore, these cyclization are less favorable. Only in case of substrate **6**, which after direct excitation and initial cyclization can give rise to stable bi-radical intermediate III. Subsequent ISC and radical recombination afforded the desired product in 52% yield and 3:1 dr. Substrate **SI-7A** was irradiated with different light sources with appropriate photosensitizers, but direct excitation with 365 nm light afforded the desired product with best yield.

Building block synthesis and further functionalization

3.4. Building block synthesis

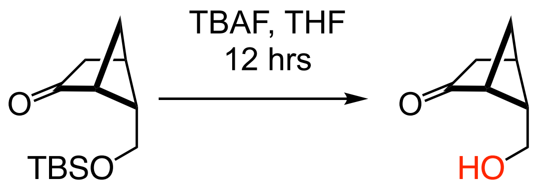

10 **SI-9**

5-(hydroxymethyl)bicyclo[2.1.1]hexan-2-one (SI-9): To a flame dried 250 ml round bottom flask equipped with a stir bar was added 5-(((tert-butyldimethylsilyl)oxy)methyl)bicyclo[2.1.1]hexan-2-one (3.3 g, 1.0 equiv., 13.73 mmol) and dissolved in THF (140 mL). To this tetrabutylammonium fluoride (20.59 mL, 1 molar, 1.5 equiv., 20.59 mmol) was added dropwise for 5 min at room temperature, which resulted in a dark yellow solution. The reaction mixture was stirred for 12 hrs at room temperature, and then water was added to quench the reaction. The resulting mixture was extracted with EtOAc, dried over anh. Na_2SO_4 and concentrated in *vacuo*. The purification of the crude product by MPLC (gradient 30-35% EtOAc in Hex) to afford **SI-9** as a colourless oil (1.41 g, 11.12 mmol, 81 %, 2:1 dr).

R_f: 0.3 in 70% Ea/Hex.

^{1}H NMR (600 MHz, $CDCl_3$) δ 4.07 – 4.02 (m, 0.8H) (minor), 3.54 – 3.44 (m, 2H), 2.88 (dt, *J* = 3.8, 2.2 Hz, 2H), 2.82 – 2.76 (m, 0.4H) (minor), 2.75 – 2.70 (m, 0.8H) (minor), 2.59 (ddd, *J* = 8.1, 6.5, 3.1 Hz, 1H), 2.34 – 2.23 (m, 1H), 2.18 (ddd, *J* = 16.3, 4.8, 1.0 Hz, 1H), 2.10 (dd, *J* = 2.8, 1.4 Hz,

0.6H) (minor), 2.06 (ddtd, J = 8.9, 4.7, 3.1, 1.4 Hz, 1H), 1.74 – 1.64 (m, 0.4H) (minor), 1.58 (d, J = 7.2 Hz, 1H).

^{13}C NMR (126 MHz, $CDCl_3$) δ 215.1, 213.5 (minor), 59.8, 59.8 (minor), 57.5, 57.0 (minor), 55.2 (minor), 52.0, 43.6 (minor), 38.7, 37.7, 37.0 (minor), 36.9, 36.2 (minor).

IR (near): 3392 (br), 2950 (w), 1744 (s), 1029 (m).

HRMS (EI, *m/z*): Calculated for $C_7H_{10}O_2$; $[M]^+$ 126.0681; found 126.0684.

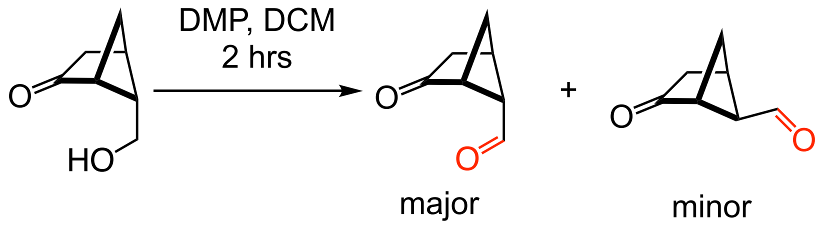

SI-9 **18** **19**

2-oxobicyclo[2.1.1]hexane-5-carbaldehyde (18 & 19): To a flame-dried 250 ml round bottom flask equipped with a stir bar was added 5-(hydroxymethyl)bicyclo[2.1.1]hexan-2-one (1.4 g, 1.0 equiv., 11 mmol) and dissolved in CH_2Cl_2 (140 mL). To this DMP (7.1 g, 1.5 equiv., 17 mmol) was added at 0 °C. The reaction mixture was moved to room temperature and stirred for 2 hrs. Upon consumption of the starting material monitored by Thin layer chromatography, the reaction was quenched with sat. $Na_2S_2O_3$ solution and followed by addition of sat. $NaHCO_3$. The aqueous part was extracted with CH_2Cl_2, and washed with Sat. $NaHCO_3$. The combined organic layer was dried over Na_2SO_4, filtered and concentrated in *vacuo*. The purification of the crude product by flash column chromatography on silica gel to afford two diastereomers of the aldehyde which was subjected for subsequent Pinnick oxidations. Note: both diastereomers of the aldehyde are very volatile.

Major diastereomer (18):

R_f: 0.6 in 90% Ethyl ether/hex

^{1}H NMR (500 MHz, $CDCl_3$) δ 9.59 (s, 1H), 3.27 (s, 1H), 3.21 (s, 1H), 3.05 (dt, J = 6.7, 2.1 Hz, 1H), 2.22 – 2.13 (m, 2H), 2.07 (d, J = 16.8 Hz, 1H), 1.50 (d, J = 6.8 Hz, 1H).

^{13}C NMR (126 MHz, CDCl$_3$) δ 210.3, 199.5, 58.8, 56.5, 40.1, 37.8, 37.0.

Minor diastereomer (19):

R$_f$: 0.5 in 90% Ethyl ether/hex

^{1}H NMR (500 MHz, CDCl$_3$) δ 10.00 (s, 1H), 3.22 (qd, *J* = 6.6, 3.9 Hz, 2H), 2.82 (dd, *J* = 7.4, 2.1 Hz, 1H), 2.77 (dt, *J* = 7.2, 3.8 Hz, 1H), 2.41 – 2.31 (m, 2H), 1.73 (t, *J* = 8.0 Hz, 1H).

^{13}C NMR (126 MHz, CDCl$_3$) δ 209.8, 199.1, 62.7, 57.2, 42.7, 38.5, 38.1.

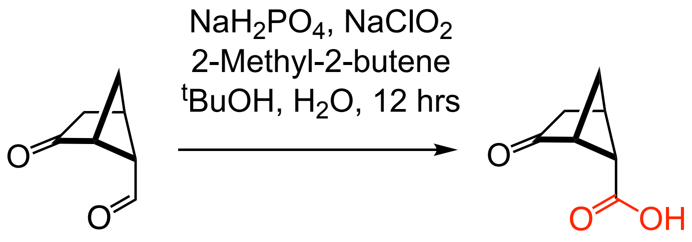

18 **20**

2-oxobicyclo[2.1.1]hexane-5-carboxylic acid (20): To a 100 ml round bottom flask equipped with a stir bar was added 2-oxobicyclo[2.1.1]hexane-5-carbaldehyde (1g, 1 equiv., 8.10 mmol) and dissolved in tert-BuOH (80 ml). Followed by delta-Isoamylene (1.695 g, 2.50 mL, 3 equiv., 24.2 mmol) was added to the reaction mixture. Next, a solution sodium chlorite (2.550 g, 3.5 equiv., 28.2 mmol) and sodium dihydrogen phosphate (3.866 g, 4 equiv., 32.2 mmol) in water (50 ml) was added to the reaction mixture dropwise and the reaction was stirred 12 hrs. Next, the reaction mixture was acidified with 1 N HCl to pH 1 followed by extraction with Ethyl acetate (*5). Next, the combined organic layer was dried over Na_2SO_4, filtered and concentrated in *vacuo.* The purification of the crude product by flash column chromatography on silica gel to afford **20** (700 mg, 5.00 mmol, 62 % over 2 steps) as a white semi-solid.

R$_f$: 0.8 in 80% EA/Hex.

^{1}H NMR (500 MHz, CDCl$_3$) δ 3.20 (ddt, *J* = 5.2, 3.5, 1.6 Hz, 1H), 3.15 (dd, *J* = 4.1, 2.5 Hz, 1H), 3.09 (dt, *J* = 6.5, 2.5 Hz, 1H), 2.33 (ddd, *J* = 16.4, 5.1, 1.3 Hz, 1H), 2.18 – 2.09 (m, 2H), 1.51 (d, *J* = 7.4 Hz, 1H).

^{13}C NMR (126 MHz, CDCl$_3$) δ 210.7, 175.5, 57.4, 51.5, 40.2, 37.6, 37.2.

IR (near): 3400 (br), 2975 (w), 1753 (s), 1709 (s), 1230 (m).

HRMS (ESI): Calculated for $C_7 H_8 O_3$ Na; $[M+Na]^+$ 163.0366; found 163.0365.

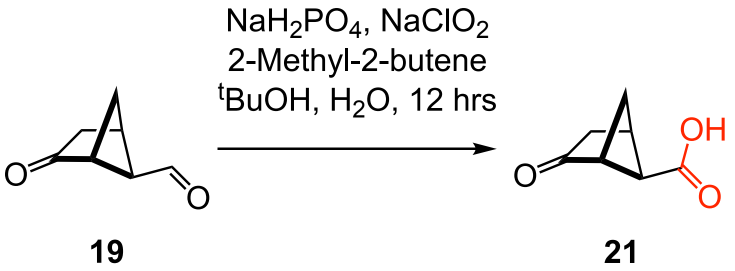

2-oxobicyclo[2.1.1]hexane-5-carboxylic acid (21): To a 100 ml round bottom flask equipped with a stir bar was added 2-oxobicyclo[2.1.1]hexane-5-carbaldehyde (300 mg, 1.0 equiv. 2.42 mmol) and dissolved in tert-BuOH (25 ml). Followed by delta-Isoamylene (508 mg, 751 µL, 3.0 equiv., 7.25 mmol) was added to the reaction mixture. Next, a solution of sodium chlorite (765 mg, 3.5 equiv., 8.46 mmol) and sodium dihydrogen phosphate (1.16 g, 4.0 equiv., 9.67 mmol) in water (16 ml) was added to the reaction mixture dropwise and the reaction was stirred 12 hrs. Next, the reaction mixture was acidified with 1 N HCl to pH 1 followed by extraction with Ethyl acetate. Next, the combined organic layers were dried over anh. Na_2SO_4, filtered and concentrated in *vacuo*. The purification of the crude product by flash column chromatography on silica gel to afford **21** (150 mg, 2.42 mmol, 44 % over 2 steps) as a white solid.

R_f: 0.7 in 80% EA/Hex.

^{1}H NMR (500 MHz, $CDCl_3$) δ 10.79 (s, 1H), 3.21 – 3.09 (m, 2H), 2.87 (dq, *J* = 5.4, 2.8 Hz, 1H), 2.83 (d, *J* = 7.5 Hz, 1H), 2.35 (d, *J* = 2.1 Hz, 2H), 1.72 (t, *J* = 7.9 Hz, 1H).

^{13}C NMR (126 MHz, $CDCl_3$) δ 210.4, 176.9, 58.7, 54.4, 42.8, 39.3, 39.2.

IR (near): 3479 (br), 2932 (w), 1736 (s), 1706 (s), 1231 (m).

HRMS (EI, *m/z*): Calculated for $C_7 H_8 O_3$; $[M]^+$ 140.0473; found 140.0473.

3.5. Further functionalization

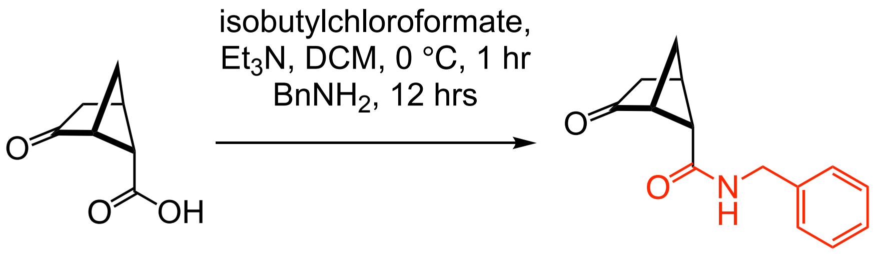

20 **22**

5-((benzyl-λ²-azaneyl)carbonyl)bicyclo[2.1.1]hexan-2-one (22): To a flame dried 2-dram vial equipped with a stir bar and capped with a septum was added the 2-oxobicyclo[2.1.1]hexane-5-carboxylic acid (100 mg, 1.0 equiv., 0.71 mmol) dissolved in CH_2Cl_2 (2 ml). This acid solution was cooled down to -10 °C followed by triethylamine (108 mg, 149 µL, 1.5 equiv., 1.07 mmol) and Isobutylchloroformate (136 mg, 130 µL, 1.4 equiv., 999 µmol) was added dropwise to the reaction mixture. Next, the reaction mixture was stirred at -10 °C for 1 hr and then benzylamine (115 mg, 117 µL, 1.5 equiv., 1.07 mmol) was added to the reaction mixture. The septum was replaced with a screw cap and the reaction was stirred 12 hrs while warming to room temperature. Next day, the reaction was quenched with sat NH_4Cl and the aqueous layer was extracted with CH_2Cl_2 (*3 times). The combined organic layer was dried over Na_2SO_4 and filtered. The filtrate was concentrated in *vacuo* and purified via MPLC (gradient 70-80% EtOAc in Hex) to afford **22** a white powder (100 mg, 0.44 mmol, 61 %).

R_f: 0.5 in 10% MeOH in CH_2Cl_2.

^{1}H NMR (500 MHz, $CDCl_3$) δ 7.36 – 7.21 (m, 5H), 5.72 (s, 1H), 4.45 – 4.34 (m, 2H), 3.13 (ddt, *J* = 7.1, 3.7, 2.0 Hz, 1H), 3.03 (ddt, *J* = 10.9, 6.0, 2.7 Hz, 2H), 2.32 (ddd, *J* = 16.2, 5.0, 1.3 Hz, 1H), 2.11 – 2.05 (m, 2H), 1.50 (d, *J* = 7.3 Hz, 1H).

^{13}C NMR (126 MHz, $CDCl_3$) δ 211.4, 169.3, 137.9, 128.9, 128.0, 127.8, 57.2, 53.5, 43.7, 39.8, 37.3, 37.0.

IR (neat): 3306 (br), 2998 (w), 1753 (s), 1654 (s), 1545 (m), 1259 (m).

HRMS (ESI): Calculated for $C_{14}H_{15}O_2NNa$; $[M+Na]^+$ 252.0995; found 252.0997.

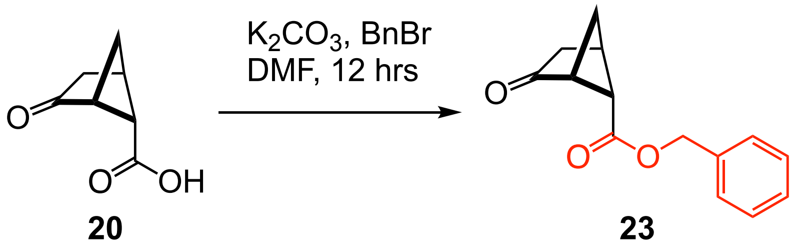

20 **23**

benzyl-2-oxobicyclo[2.1.1]hexane-5-carboxylate (23): To a flame dried 2-dram vial equipped with a stir bar and capped with a septum was added 2-oxobicyclo[2.1.1]hexane-5-carboxylic acid (500 mg, 1.0 equiv., 3.57 mmol)and it was dissolved in DMF (12 ml). Next, the reaction was cooled to 0 °C and K_2CO_3 (986 mg, 2.0 equiv., 7.14 mmol) was added to it. Followed by benzyl bromide (732 mg, 509 μL, 1.2 equiv., 4.28 mmol) was added and the reaction was brought to room temperature. The reaction was stirred at room temperature for 12 hrs. Next, it was quenched with saturated NH_4Cl solution. The aqueous layer was extracted with 10 ml ethyl ether (*3). The combined organic layer was dried over Na_2SO_4 and filtered. The filtrate was concentrated in *vacuo* and purified via MPLC (gradient 15-20% EtOAc in Hex) to afford **23** a yellowish white oil (500 mg, 2.17 mmol, 61 %).

R_f: 0.5 in 30% EA/Hex

^{1}H NMR (500 MHz, $CDCl_3$) δ 7.44 – 7.27 (m, 5H), 5.09 (s, 2H), 3.21 – 3.13 (m, 2H), 3.08 (dt, *J* = 5.6, 2.7 Hz, 1H), 2.21 (ddd, *J* = 15.9, 4.9, 1.1 Hz, 1H), 2.14 – 2.04 (m, 2H), 1.48 (d, *J* = 7.5 Hz, 1H).

^{13}C NMR (126 MHz, $CDCl_3$) δ 210.6, 169.8, 135.5, 128.7, 128.6, 128.5, 66.8, 57.5, 51.8, 40.1, 37.5, 37.3.

IR (neat): 2959 (w), 1762 (s), 1731 (s), 1291 (m).

HRMS (ESI): Calculated for $C_{14} H_{14} O_3$ Na; $[M+Na]^+$ 253.0835; found 253.0835.

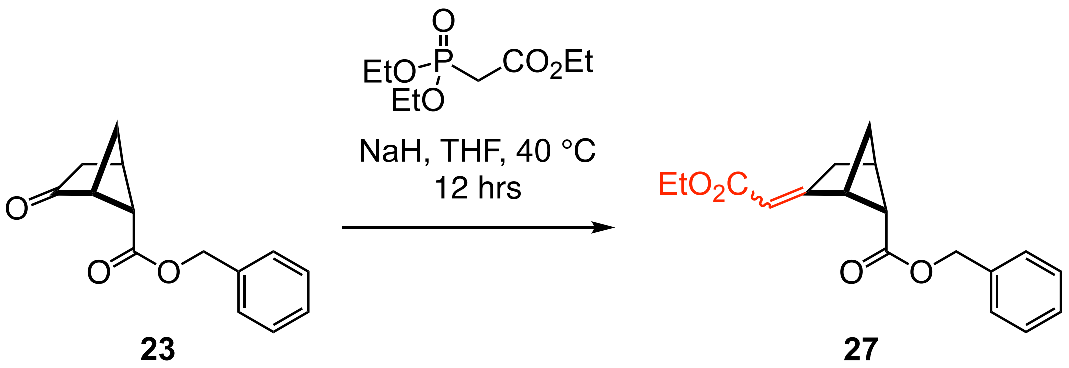

benzyl-2-(2-ethoxy-2-oxoethylidene)bicyclo[2.1.1]hexane-5-carboxylate (27): To a flame dried 2-dram vial equipped with a stir bar and capped with a septum was added the sodium hydride (7.3 mg, 60% Wt, 1.4 equiv., 0.18 mmol) followed by THF (0.2 ml). The NaH suspension was cooled to 0-5 °C and followed by ethyl 2-(diethoxyphosphoryl)acetate (44 mg, 39 µL, 1.5 equiv., 0.20 mmol) was added and the mixture was stirred at 0-5 °C for 30 minutes. Next, a solution of benzyl-2-oxobicyclo[2.1.1]hexane-5-carboxylate (30 mg, 1.0 equiv., 0.13 mmol) in THF (0.2 mL) was added, and the resulting mixture was allowed to warm to 40 °C temperature. After 48 h, the mixture was treated with EtOAc and water and the aqueous layer was extracted with EtOAc (*3 times). The organic layer was dried over Na_2SO_4 and filtered and concentrated. The residue was purified by MPLC (gradient 8-10% EtOAc in Hex) to give **27** as a colourless oil (24 mg, 0.08 mmol, 61 %, dr 1:1.1).

R_f: 0.6 in 20% EA/Hex.

^{1}H NMR (500 MHz, $CDCl_3$) δ 7.35 – 7.17 (m, 10H), 5.90 (t, *J* = 1.6 Hz, 1H), 5.67 (s, 0.8H) (minor), 5.03 (dd, *J* = 12.2, 2.3 Hz, 2H), 4.92 (dd, *J* = 16.6, 12.3 Hz, 2H), 4.46 (dt, *J* = 5.8, 2.7 Hz, 0.8H) (minor), 4.15 – 4.03 (m, 4H), 3.25 (dt, *J* = 6.5, 2.6 Hz, 1H), 2.91 – 2.80 (m, 4H), 2.74 (ddt, *J* = 16.9, 3.7, 1.6 Hz, 1H), 2.59 (dq, *J* = 17.0, 2.0 Hz, 1H), 2.47 (ddt, *J* = 15.5, 3.5, 1.5 Hz, 1H), 2.23 (dq, *J* = 15.5, 1.8 Hz, 0.9H) (minor), 1.79 (dd, *J* = 6.8, 3.4 Hz, 0.9H) (minor), 1.77 – 1.70 (m, 1H), 1.22 (dt, *J* = 13.9, 7.1 Hz, 6H), 1.12 (d, *J* = 6.8 Hz, 1H), 1.08 (d, *J* = 6.9 Hz, 0.9H) (minor).

^{13}C NMR (126 MHz, $CDCl_3$) δ 170.2, 170.2, 166.9, 166.6, 164.4, 163.1, 136.0, 135.9, 128.6, 128.6, 128.4, 128.3, 128.2, 128.2, 112.5, 110.8, 66.3, 66.1, 59.8, 53.6, 52.6, 51.8, 50.6, 40.1, 39.5, 39.2, 38.5, 34.1, 34.1, 14.5, 14.5.

IR (neat): 2986 (w), 1709 (s), 1673 (m), 1455 (w) 1219 (m).

HRMS (ESI): Calculated for $C_{18}H_{20}O_4Na$; $[M+Na]^+$ 323.1254; found 323.1257.

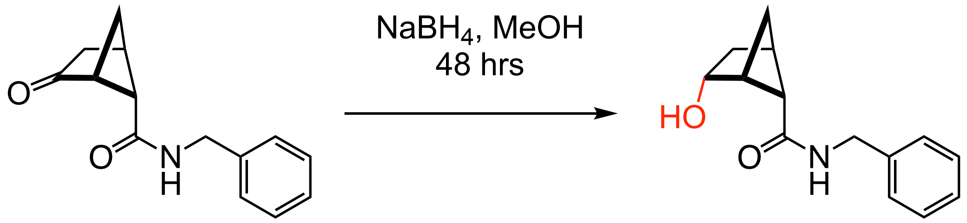

22 **24**

(benzyl-λ²-azaneyl)(-2-hydroxybicyclo[2.1.1]hexan-5-yl)methanone (24): To a flame-dried 2-dram vial equipped with a stir bar and capped with a septum was added N-benzyl-2-oxobicyclo[2.1.1]hexane-5-carboxamide (23 mg, 1.0 equiv., 0.10 mmol) and dissolved in MeOH (0.2 ml, 0.5 M) to this was added sodium borohydride (11 mg, 3.0 equiv., 0.30 mmol) in one portion. The vial was capped and stirred at room temperature for 48 hrs. After that, the reaction mixture was acidified with 1 N HCl and concentrated. The crude mixture was dissolved in water and the aqueous layer was extracted with CH_2Cl_2 (*3 times). The combined organic layer were dried over Na_2SO_4, filtered, concentrated in *vacuo*, and purified by MPLC (gradient 70-80% EtOAc in Hex) to afford **24** a white solid (21 mg, 0.91 mmol, 91 %).

R_f: 0.5 in 90% EA/Hex.

^{1}H NMR (500 MHz, $CDCl_3$) δ 7.37 – 7.26 (m, 5H), 5.93 (s, 1H), 5.64 (d, *J* = 11.0 Hz, 1H), 4.41 (d, *J* = 5.7 Hz, 2H), 4.25 (ddd, *J* = 10.8, 7.4, 2.8 Hz, 1H), 2.88 (dtd, *J* = 6.6, 2.6, 1.4 Hz, 1H), 2.77 (ddt, *J* = 6.4, 3.0, 1.4 Hz, 1H), 2.56 (q, *J* = 1.7 Hz, 1H), 2.18 (ddt, *J* = 12.1, 7.6, 1.5 Hz, 1H), 1.64 – 1.59 (m, 1H), 1.42 (dq, *J* = 6.6, 3.2 Hz, 1H), 0.91 (dd, *J* = 7.1, 0.9 Hz, 1H).

^{13}C NMR (126 MHz, $CDCl_3$) δ 173.0, 137.9, 129.0, 128.0, 127.9, 72.3, 51.0, 49.2, 44.4, 43.9, 37.2, 35.4.

IR (neat): 3278 (br), 2965 (m), 1638 (s), 1553 (m), 1495 (m), 1268 (s).

HRMS (ESI): Calculated for $C_{14}H_{17}O_2NNa$; $[M+Na]^+$ 254.1152; found 254.1155.

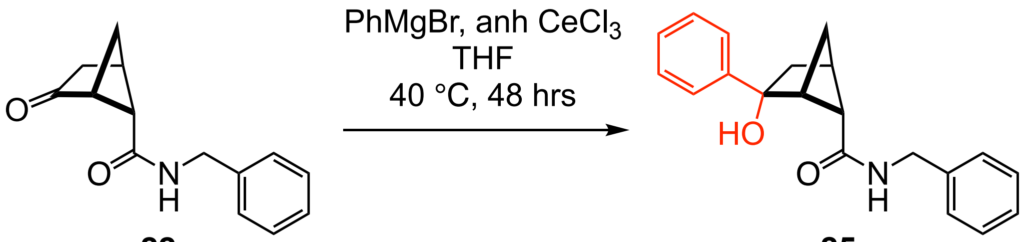

***N*-benzyl-2-hydroxy-2-phenylbicyclo[2.1.1]hexane-5-carboxamide (25):** To a flame-dried 2-dram vial equipped with a stir bar and capped with a septum was added N-benzyl-2-oxobicyclo[2.1.1]hexane-5-carboxamide (23 mg, 1.0 equiv., 0.10 mmol) and dissolved THF (0.33 ml, 0.3 M). To this was added cerium (III) chloride (49 mg, 2.0 equiv., 0.20 mmol) followed by Phenyl magnesium bromide (73 mg, 0.40 mL, 1 molar, 4.0 equiv., 0.40 mmol) dropwise. The vial was capped and stirred at 40 °C for 48 hrs. After that, the reaction mixture was quenched with sat. NH_4Cl and the aqueous layer was extracted with CH_2Cl_2 (*3 times). The combined organic layers were dried over Na_2SO_4, filtered, concentrated in *vacuo*, and purified by MPLC (gradient 15-20% EtOAc in Hex) to afford **25** a white powder (25 mg, 0.081 mmol, 81 %)·

R_f: 0.5 in 30% EA/Hex.

^{1}H NMR (500 MHz, $CDCl_3$) δ 7.53 – 7.27 (m, 9H), 7.25 – 7.20 (m, 1H), 5.96 (s, 1H), 5.73 – 5.63 (m, 1H), 4.52 – 4.41 (m, 2H), 3.24 (dt, *J* = 6.9, 2.6 Hz, 1H), 2.81 (ddt, *J* = 6.8, 3.1, 1.7 Hz, 1H), 2.69 (s, 1H), 2.58 (dt, *J* = 12.1, 1.6 Hz, 1H), 2.37 – 2.30 (m, 1H), 1.47 (dq, *J* = 6.3, 3.0 Hz, 1H), 1.06 (d, *J* = 7.3 Hz, 1H).

^{13}C NMR (126 MHz, $CDCl_3$) δ 173.1, 146.2, 138.0, 129.0, 128.4, 128.1, 127.9, 127.1, 126.9, 80.3, 53.3, 52.4, 43.9, 42.6, 40.6, 38.2.

IR (neat): 3271 (br), 2979 (m), 1635 (s), 1557 (s), 1454 (s), 1268 (m).

HRMS (ESI): Calculated for C_{20} H_{21} O_2 N Na; $[M+Na]^+$ 330.1465; found 330.1468.

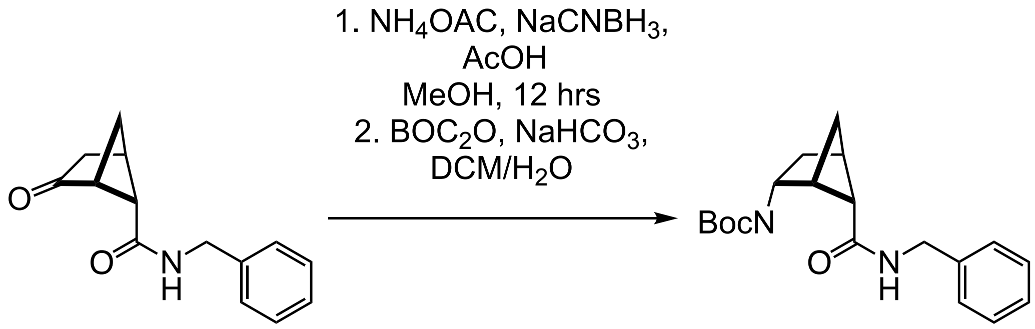

22 **26**

***tert*-butyl-5-(benzylcarbamoyl)bicyclo[2.1.1]hexan-2-yl)-λ²-azanecarboxylate (26):** To a flame-dried 2-dram vial equipped with a stir bar and capped with a septum was added N-benzyl-2-oxobicyclo[2.1.1]hexane-5-carboxamide (13 mg, 1.0 equiv., 0.057 mmol) and ammonium acetate (0.22 g, 50.0 equiv., 2.8 mmol). To this was added MeOH (0.1 M, 0.57 ml) and few drops of AcOH, followed by the reaction mixture was stirred for 30 mins. Next, Sodium cyanoborohydride (18 mg, 17 µL, 5 equiv..0, 0.28 mmol) was added and the vial was capped and stirred at room temperature for 12 hrs. After that, the solvent was evaporated in vacuo and the aqueous layer was extracted with EtOAc(*3 times). The combined organic layers were dried over Na_2SO_4, filtered, concentrated in *vacuo*, and directly used for the next step.

The crude material was dissolved in 1:1 CH_2Cl_2/water (0.6 ml). To this sodium hydrogen carbonate (9.6 mg, 2.0 equiv., 0.11 mmol) was added, followed by di-tert-butyl dicarbonate (15 mg, 1.2 equiv., 68 µmol), and the reaction was stirred for 12 hrs. After that, the aqueous layer was extracted with EtOAc (*3 times) and the combined organic layers were dried over Na_2SO_4, filtered, and concentrated in *vacuo*. The crude material was purified via MPLC (gradient 20-25% EtOAc in Hex) to afford **26** an off-white solid (9.4 mg, 0.028 mmol, 50 % over 2 steps).

R_f: 0.5 in 50% EA/Hex.

^{1}H NMR (500 MHz, $CDCl_3$) δ 7.31 – 7.09 (m, 5H), 6.22 (d, *J* = 8.3 Hz, 1H), 5.64 (s, 1H), 4.52 – 4.23 (m, 2H), 4.15 – 3.98 (m, 1H), 2.78 (tt, *J* = 3.9, 2.3 Hz, 1H), 2.73 – 2.53 (m, 1H), 2.44 (d, *J* = 3.1 Hz, 1H), 2.24 – 2.02 (m, 1H), 1.38 (s, 9H), 0.87 (d, *J* = 7.1 Hz, 1H).

^{13}C NMR (126 MHz, $CDCl_3$) δ 172.3, 156.3, 138.1, 129.0, 128.0, 127.8, 78.9, 50.5, 50.1, 47.5, 43.6, 42.3, 37.4, 33.5, 28.7.

IR (near): 3306 (br), 2979 (w), 1686 (s), 1663 (s), 1508 (s), 1363 (m).

HRMS (ESI): Calculated for C_{19} H_{26} O_3 N_2 Na ; $[M+Na]^+$ 353.1836; found 353.1839.

3.6. Synthesis of 28:

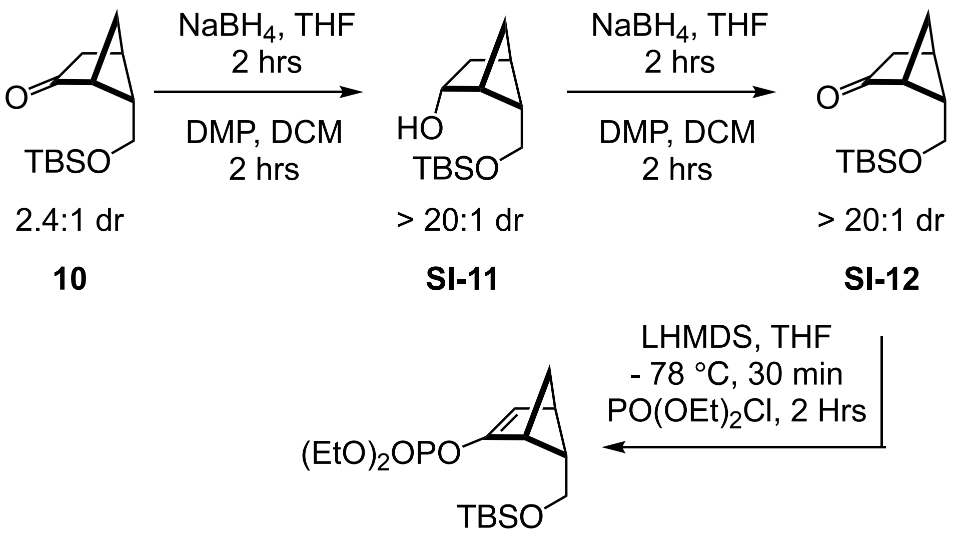

28

5-(((tert-butyldimethylsilyl)oxy)methyl)bicyclo[2.1.1]hex-2-en-2-yl diethyl phosphate (28): To a flame-dried 2-dram vial equipped with a stir bar and capped with a septum was added **10** (100 mg, 832 µL, 0.5 molar, 1 equiv., 416 µmol) and dissolved in MeOH (0.8 ml, 0.5 M) to this was added sodium borohydride (47.2 mg, 3 equiv., 1.25 mmol) in one portion. The vial was capped and stirred at room temperature for 2 hrs. The completion of the reaction was monitore by Thin layer chromatography. After that, the reaction mixture was acidified with saturated Nh4Cl and concentrated. The crude mixture was dissolved in water and the aqueous layer was extracted with EtOAc (*3 times). The combined organic layer was dried over Na_2SO_4, filtered, concentrated in *vacuo*, and purified by MPLC (gradient 70-80% EtOAc in Hex) to afford **SI-11** as a white solid (48 mg, 0.20 mmol, 48 %, > 20:1 dr)

R_f: 0.5 in 20% EA/Hex.

^{1}H NMR (500 MHz, $CDCl_3$) δ 4.24 (t, *J* = 7.2 Hz, 1H), 3.74 (dd, *J* = 11.4, 7.0 Hz, 1H), 3.60 (dd, *J* = 11.4, 6.4 Hz, 1H), 3.38 (d, *J* = 6.9 Hz, 1H), 2.65 – 2.53 (m, 1H), 2.43 (ddt, *J* = 6.6, 3.0, 1.5 Hz, 1H), 2.09 – 1.95 (m, 2H), 1.57 (dddd, *J* = 11.9, 3.7, 2.4, 1.2 Hz, 1H), 1.35 (dq, *J* = 6.7, 3.2 Hz, 1H), 0.89 (s, 9H), 0.85 (d, *J* = 6.9 Hz, 1H), 0.06 (d, *J* = 2.8 Hz, 6H).

^{13}C NMR (126 MHz, $CDCl_3$) δ 72.3, 61.5, 48.5, 48.2, 41.1, 37.6, 35.1, 26.0, 18.4, -5.1 (d).

Next, to a flame-dried 2 Dram vial equipped with a stir bar was added **SI-11** (76.00 mg, 1.567 mL, 0.2 molar, 1 equiv., 313.5 μmol) and dissolved in CH_2Cl_2 (1.5 ml, 0.2 molar). To this DMP (172.9 mg, 1.3 equiv., 407.5 μmol) was added at 0 °C. The reaction mixture was moved to room temperature and stirred for 2 hrs. Upon consumption of the starting material monitored by Thin layer chromatography, the reaction was quenched with sat. $Na_2S_2O_3$ solution and followed by addition of sat. $NaHCO_3$. The aqueous part was extracted with CH_2Cl_2, and washed with Sat. $NaHCO_3$. The combined organic layer was dried over Na_2SO_4, filtered and concentrated in *vacuo*. The purification of the crude product by flash column chromatography on silica gel to afford **SI-12** as a colourless oil (66 mg, 0.27 mmol, 88 %).

^{1}H NMR (500 MHz, $CDCl_3$) δ 3.49 – 3.36 (m, 2H), 2.89 – 2.74 (m, 2H), 2.53 (ddd, *J* = 8.0, 6.0, 3.1 Hz, 1H), 2.16 (ddd, *J* = 16.3, 4.9, 1.2 Hz, 1H), 2.08 – 1.98 (m, 2H), 1.53 (d, *J* = 7.2 Hz, 1H), 0.87 (s, 9H), 0.02 (d, *J* = 1.8 Hz, 6H).

^{13}C NMR (126 MHz, $CDCl_3$) δ 214.1, 60.6, 57.5, 52.2, 38.7, 37.2, 37.0, 26.0, 18.4, -5.2, -5.3.

After that a flame-dried 2 Dram vial equipped with a stir bar was added 5-(((tert-butyldimethylsilyl)oxy)methyl)bicyclo[2.1.1]hexan-2-one (300 mg, 2.50 mL, 0.5 molar, 1 equiv., 1.25 mmol) and dissolved in THF (2.5 ml, 0.5 M). To this LiHMDS (418 mg, 2.50 mL, 1 molar, 2 equiv., 2.50 mmol) dropwise was added at -78 °C and stirred for 30 mins. Next, 1-[chloro(ethoxy)phosphoryl]oxyethane (431 mg, 361 μL, 2 equiv., 2.50 mmol) was added to the reaction mixture. Next, the reaction mixture was moved to room temperature and stirred for 2 hrs. Upon consumption of the starting material monitored by Thin layer chromatography, the reaction was quenched with saturated NH4Cl solution and followed by addition of water. The aqueous part was extracted with ether (3 times). The combined organic layer was dried over Na_2SO_4, filtered and concentrated in *vacuo*. The purification of the crude product by flash column chromatography on silica gel to afford **28** as a colourless oil (360 mg, 0.96 mmol, 76.6 %).

R_f: 0.5 in 30% EA/Hex.

^{1}H NMR (500 MHz, CDCl$_3$) δ 5.72 (dd, *J* = 2.4, 1.5 Hz, 1H), 4.18 (dqd, *J* = 8.1, 7.1, 3.8 Hz, 4H), 3.71 (d, *J* = 6.9 Hz, 2H), 3.29 – 3.23 (m, 1H), 2.63 – 2.56 (m, 1H), 2.51 – 2.39 (m, 3H), 1.36 (td, *J* = 7.1, 1.1 Hz, 6H), 0.86 (s, 9H), 0.02 (s, 6H).

^{13}C NMR (126 MHz, CDCl$_3$) δ 163.8, 163.7 (d, J = 35 Hz), 110.1, 110.1 (d, J = 25 Hz), 78.2, 64.6 (q, J = 15 Hz), 64.6, 64.6, 64.5, 64.5, 61.6, 47.3, 47.2 (d, J = 20 Hz), 41.5, 26.1, 18.5, 16.3, 16.2 (d, J = 30 Hz), -5.1, -5.1 (d, J = 5 Hz).

IR (near): 2981 (w), 2857 (w), 1616 (w), 1296 (m), 1508 (m).

HRMS (ESI): Calculated for C_{17} H_{33} O_5 P Si Na ; [M+Na]$^+$ 399.1738; found 399.1727.

3.7. General procedure C: Kumada coupling.

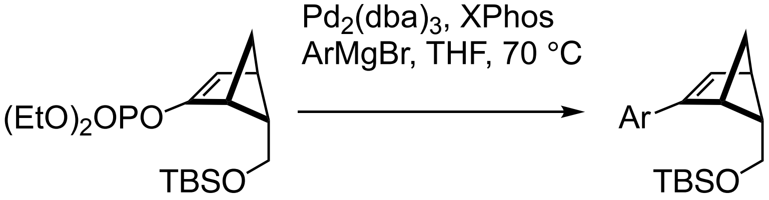

In an N2-filled glovebox, to a flamed dried screw-capped vial, equipped with a stir bar was added Pd(dba)2 (2 mol %) and X-Phos (4 mol %). The vial was sealed with a septum and then removed from the glove box. Enol-phosphate **28** (1.0 equiv.) and THF (2 mL) were then added. To the resultant red mixture was added the Grignard reagent (2.5 equiv.) dropwise over 5 min. The vial was sealed with a Teflon-lined screw cap and the reaction was allowed to stir for the indicated time (see text) at 70 C. The reaction was quenched upon addition of saturated NH_4Cl (1 mL) and water (1 mL) and the mixture was extracted with Et_2O (3 x 3 mL). The combined organic layers were dried over Na_2SO_4, filtered, and concentrated. The residue was purified by silica gel column chromatography to obtain the desired product.

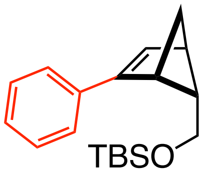

29

***tert*-butyldimethyl((2-phenylbicyclo[2.1.1]hex-2-en-5-yl)methoxy)silane**: This compound was synthesized according to general procedure C using 5-(((tert-butyldimethylsilyl)oxy)methyl)bicyclo[2.1.1]hex-2-en-2-yl diethyl phosphate (40 mg, 0.11 mmol) and purified using MPLC (gradient 4-5% Ether in Hex) to isolate **29** as a colourless oil (22 mg, 0.07 mmol, 69 %).

R_f: 0.8 in 5% Ether/Hex.

^{1}H NMR (500 MHz, $CDCl_3$) δ 7.44 – 7.39 (m, 2H), 7.30 (dd, *J* = 8.5, 7.0 Hz, 2H), 7.23 – 7.16 (m, 1H), 6.88 (t, *J* = 2.2 Hz, 1H), 3.56 (d, *J* = 6.9 Hz, 2H), 3.29 (ddt, *J* = 7.0, 4.6, 2.4 Hz, 1H), 3.07 (dq, *J* = 8.6, 2.2 Hz, 1H), 2.68 (dq, *J* = 8.6, 2.3 Hz, 1H), 2.45 (dt, *J* = 4.9, 2.3 Hz, 1H), 2.38 (d, *J* = 5.1 Hz, 1H), 0.84 (s, 9H), -0.06 (d, *J* = 7.7 Hz, 6H).

^{13}C NMR (126 MHz, $CDCl_3$) δ 152.4, 136.0, 131.7, 128.5, 126.9, 124.8, 76.6, 62.6, 61.7, 45.6, 44.9, 26.1, 18.5, -5.2(d).

IR (near): 2954 (m), 2884 (w), 1682 (w), 1471 (w), 1253 (m).

HRMS (ESI): Calculated for C_{19} H_{27} O Si Na ; $[M-H]^+$ 299.1826; found 299.1825.

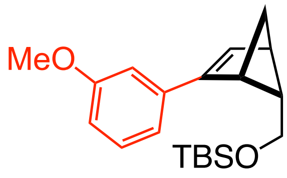

30

tert-butyl(((1R,4R,5S)-2-(3-methoxyphenyl)bicyclo[2.1.1]hex-2-en-5-yl)methoxy)dimethylsilane: This compound was synthesized according to general procedure C using 5-(((tert-butyldimethylsilyl)oxy)methyl)bicyclo[2.1.1]hex-2-en-2-yl diethyl phosphate (40 mg, 0.11 mmol) and purified using MPLC (gradient 4-5% Ether in Hex) to isolate **30** as a colourless oil (21 mg, 0.064 mmol, 60 %). Note: Product is very unstable.

R_f: 0.8 in 10% Ether/Hex.

^{1}H NMR (500 MHz, CDCl$_3$) δ 7.22 (t, *J* = 7.9 Hz, 1H), 7.00 (dt, *J* = 7.7, 1.3 Hz, 1H), 6.94 (dd, *J* = 2.7, 1.6 Hz, 1H), 6.88 (t, *J* = 2.2 Hz, 1H), 6.76 (ddd, *J* = 8.2, 2.6, 0.9 Hz, 1H), 3.82 (s, 3H), 3.54 (d, *J* = 6.9 Hz, 2H), 3.28 (ddt, *J* = 6.9, 4.6, 2.3 Hz, 1H), 3.03 (dq, *J* = 8.6, 2.2 Hz, 1H), 2.67 (dq, *J* = 8.6, 2.3 Hz, 1H), 2.44 (dt, *J* = 4.8, 2.3 Hz, 1H), 2.38 (d, *J* = 5.1 Hz, 1H), 0.83 (s, 9H), -0.06 (d, *J* = 6.0 Hz, 6H).

^{13}C NMR (126 MHz, CDCl$_3$) δ 159.8, 152.4, 137.4, 132.2, 129.5, 117.5, 112.5, 110.3, 62.7, 61.69, 55.4, 45.8, 44.9, 26.1, 18.5, -5.1(d).

IR (near): 2850 (m), 28 (m), 1630 (w), 1460 (w), 1201 (m).

HRMS (ESI): Calculated for $C_{20}H_{31}O_2Si$; $[M+H]^+$ 331.2088; found 331.2087.

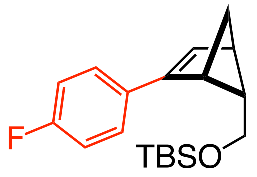

31

tert-butyl(((1R,4R,5S)-2-(4-fluorophenyl)bicyclo[2.1.1]hex-2-en-5-yl)methoxy)dimethylsilane: This compound was synthesized according to general procedure C and purified using 5-(((tert-butyldimethylsilyl)oxy)methyl)bicyclo[2.1.1]hex-2-en-2-yl diethyl phosphate (40 mg, 0.11 mmol) MPLC (gradient 4-5% Ether in Hex) to isolate **31** as a colourless oil (13 mg, 0.04 mmol, 38 %).

R_f: 0.8 in 5% Ether/Hex.

^{1}H NMR (500 MHz, CDCl$_3$) δ 7.35 (dd, *J* = 8.7, 5.5 Hz, 2H), 6.99 (t, *J* = 8.8 Hz, 2H), 6.80 (t, *J* = 2.2 Hz, 1H), 3.53 (d, *J* = 6.9 Hz, 2H), 3.32 – 3.22 (m, 1H), 3.02 (dd, *J* = 8.6, 2.3 Hz, 1H), 2.65 (dd, *J* = 8.5, 2.3 Hz, 1H), 2.43 (dt, *J* = 4.9, 2.3 Hz, 1H), 2.37 (d, *J* = 5.2 Hz, 1H), 0.83 (s, 9H), -0.07 (d, *J* = 9.8 Hz, 6H).

^{13}C NMR (126 MHz, CDCl$_3$) δ 163.0, 161.0 (d, J = 975 Hz), 151.4, 132.3, 132.3 (d, J = 15 Hz), 131.2, 131.2 (d, J = 15 Hz), 126.3, 126.3 (d, J = 30 Hz), 115.5, 115.3 (d, J=90 Hz), 76.6, 62.6, 61.6, 45.8, 44.9, 26.1, 18.5, -5.2.

IR (near): 2850 (m), 28 (m), 1630 (w), 1460 (w), 1201 (m).

HRMS (ESI): Calculated for $C_{19} H_{28} O F Si$; $[M+H]^+$ 319.1888; found 319.1887.

3.8. Matched pair synthesis

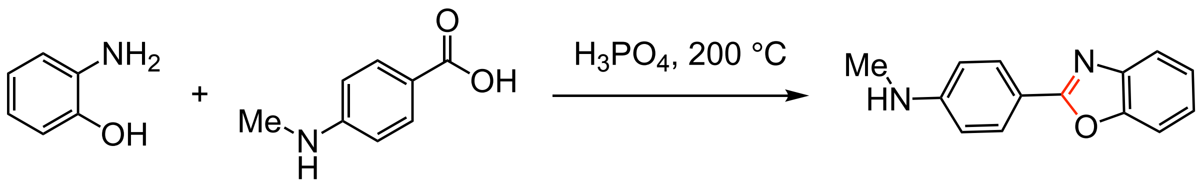

SI-13

4-(benzo[d]oxazol-2-yl)-N-methylaniline (SI-13): To a 25 ml round bottom flask was taken 4-(methylamino)benzoic acid (1000 mg, 1 equiv., 6.616 mmol) and 2-aminophenol (722.0 mg, 1 equiv., 6.616 mmol) and dissolved in polyphosphoric acid (0.1 M, 7 ml). The reaction mixture was heated to 200 °C and stirred for 4 hrs. After that, the reaction was cooled to 80 °C and poured in 100 ml of water. the aqueous layer was extracted with EtOAc (*3 times) and the combined organic layers were dried over Na_2SO_4, filtered, and concentrated in *vacuo*. The crude material was purified via column chromatography to afford **SI-13** (700 mg, 3.12 mmol, 47.2 %) as a pink solid. Spectral data matched with literature report.[iv]

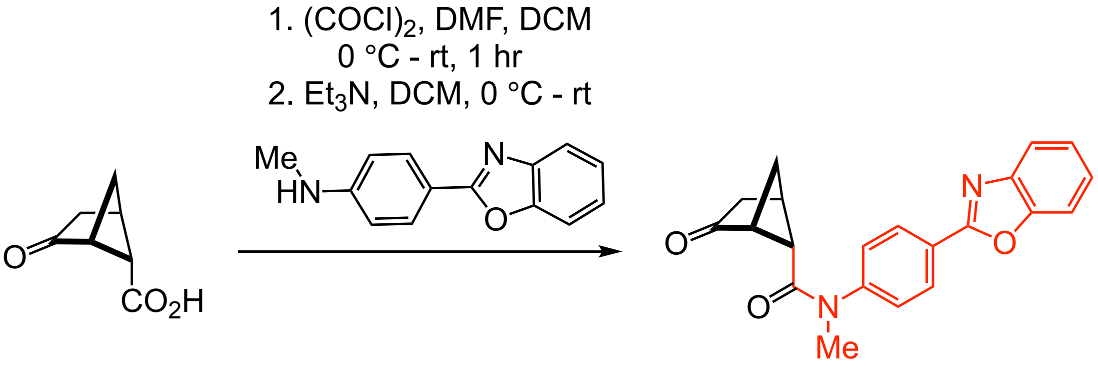

20 **SI-14**

(4-(benzo[d]oxazol-2-yl)phenyl)-N-methyl-2-oxobicyclo[2.1.1]hexane-5-carboxamide (SI-14): To a flame-dried 2-dram vial equipped with a stir bar and capped with a septum was added 2-oxobicyclo[2.1.1]hexane-5-carboxylic acid (23 mg, 1.6 mL, 0.1 molar, 1 equiv., 0.16 mmol) and dissolved DCM (1.6 ML, 0.1 M), followed by few drops of DMF was added. Next, oxalyl chloride

(42 mg, 29 µL, 2 equiv., 0.33 mmol) was added dropwise at 0 C followed by the reaction was warmed to room temperature and stirred for 1 hr. Next, the To this was added cerium (III) chloride (49 mg, 2.0 equiv., 0.20 mmol) followed by Phenyl magnesium bromide (73 mg, 0.40 mL, 1 molar, 4.0 equiv., 0.40 solvent was evaporated in vacuo and the crude material was directly used in the next reaction.

The crude material was dissolved in DCM (1.6 ml, 0.1 M) and followed by 4-(benzo[d]oxazol-2-yl)-N-methylaniline (74 mg, 2 equiv., 0.33 mmol) was added. Next, ET3N (83 mg, 0.11 mL, 5 equiv., 0.82 mmol) was added dropwise and the reaction was warmed to room temperature and stirred for 12 hrs. Next, the reaction mixture was quenched with sat. NH_4Cl and the aqueous layer was extracted with CH_2Cl_2 (*3 times). The combined organic layers were dried over Na_2SO_4, filtered, concentrated in *vacuo*, and purified by MPLC (gradient 40-50% EtOAc in Hex) to afford **SI-14** as a white powder (45 mg, 0.13 mmol, 79 %).

R_f: 0.5 in 80% EA/Hex.

^{1}H NMR (500 MHz, $CDCl_3$) δ 8.35 (d, *J* = 8.1 Hz, 2H), 7.84 – 7.74 (m, 1H), 7.67 – 7.57 (m, 1H), 7.44 – 7.32 (m, 4H), 3.28 (s, 3H), 3.00 (s, 1H), 2.75 – 2.59 (m, 2H), 2.33 (ddd, *J* = 15.8, 5.0, 1.3 Hz, 1H), 2.07 – 1.92 (m, 1H), 1.86 – 1.74 (m, 1H), 1.30 (d, *J* = 7.5 Hz, 1H).

^{13}C NMR (126 MHz, $CDCl_3$) δ 210.6, 169.2, 161.2, 151.0, 145.7, 142.1, 129.2, 127.9, 127.2, 125.7, 125.0, 120.4, 110.9, 57.8, 53.5, 40.7, 37.4, 37.2, 36.8.

IR (neat): 2992 (w), 1760 (s), 1653 (s), 1603 (m), 1490 (m), 1243 (m).

HRMS (ESI): Calculated for $C_{21}H_{18}O_3N_2Na$ $[M+Na]^+$ 369.1210; found 369.1214.

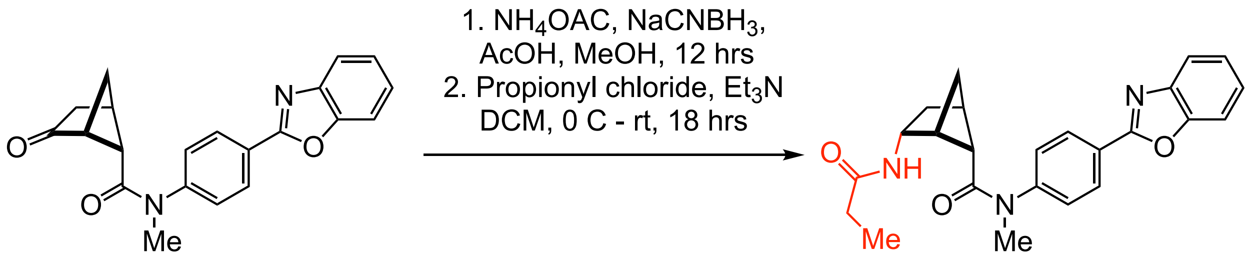

SI-14 **32**

N-(4-(benzo[d]oxazol-2-yl)phenyl)-N-methyl-2-propionamidobicyclo[2.1.1]hexane-5-carboxamide (32): To a flame-dried 2-dram vial equipped with a stir bar and capped with a septum was added N-(4-(benzo[d]oxazol-2-yl)benzyl)-N-methyl-2-oxobicyclo[2.1.1]hexane-5-carboxamide (32 mg, 89 μmol) and Sodium cyanoborohydride (28 mg, 5 equiv., 0.44 mmol). To this was added MeOH (0.1 M, 0.9 ml) and a few drops of AcOH, followed by the reaction mixture was stirred for 30 mins. Next, Sodium cyanoborohydride (18 mg, 5 equiv., 0.28 mmol) was added and the vial was capped and stirred at room temperature for 12 hrs. After that, the solvent was evaporated in vacuo and the aqueous layer was extracted with EtOAc(*3 times). The combined organic layers were dried over Na_2SO_4, filtered, concentrated in *vacuo*, and directly used for the next step.

The crude material was dissolved in CH_2Cl_2 (0.1 M, 0.9 ml) followed by Propionyl chloride (25 mg, 23 μL, 3 equiv., 0.27 mmol) was added dropwise at 0 C. Next, freshly distilled triethylamine (45 mg, 62 μL, 5 equiv., 0.44 mmol) was added dropwise and the reaction mixture was warmed to room temperature. The reaction was stirred at room temperature for 18 hrs. After that, the aqueous layer was extracted with EtOAc (*3 times) and the combined organic layers were dried over Na_2SO_4, filtered, and concentrated in *vacuo*. The crude material was purified via MPLC (gradient 80-90% EtOAc in Hex) to afford **32** an brown solid (19 mg, 0.047 mmol, 53 %).

R_f: 0.5 in 10% MeOH IN CH_2Cl_2.

^{1}H NMR (500 MHz, $CDCl_3$) δ 8.32 (d, *J* = 8.1 Hz, 2H), 7.84 – 7.75 (m, 1H), 7.75 – 7.65 (m, 1H), 7.63 – 7.59 (m, 1H), 7.41 (s, 5H), 4.32 (q, *J* = 6.9 Hz, 1H), 3.28 (s, 3H), 2.71 (t, *J* = 5.6 Hz, 1H), 2.56 (s, 1H), 2.25 (q, *J* = 7.7 Hz, 2H), 2.20 – 2.09 (m, 1H), 1.88 (s, 1H), 1.49 – 1.42 (m, 1H), 1.18 (dt, *J* = 11.7, 6.3 Hz, 4H), 0.78 (d, *J* = 7.1 Hz, 1H).

^{13}C NMR (126 MHz, $CDCl_3$) δ 173.9, 172.5, 161.9, 151.0, 145.9, 142.1, 129.2, 127.8, 127.2, 125.7, 125.0, 120.4, 110.8, 50.3, 49.1, 47.5, 44.0, 37.8, 37.5, 33.8, 30.3, 10.1.

IR (neat): 3328 (br), 2974 (w), 1642 (s), 1615 (m), 1498 (s), 1382 (m), 1242 (m).

HRMS (ESI): Calculated for $C_{24}H_{25}O_3N_3Na$ $[M+Na]^+$ 426.1788; found 426.1794.

4. Synthesis and further functionalizations of bicyclo[2.1.1]hexane-5-carboxylic acid:

4.1. Modified diazoketone synthesis:

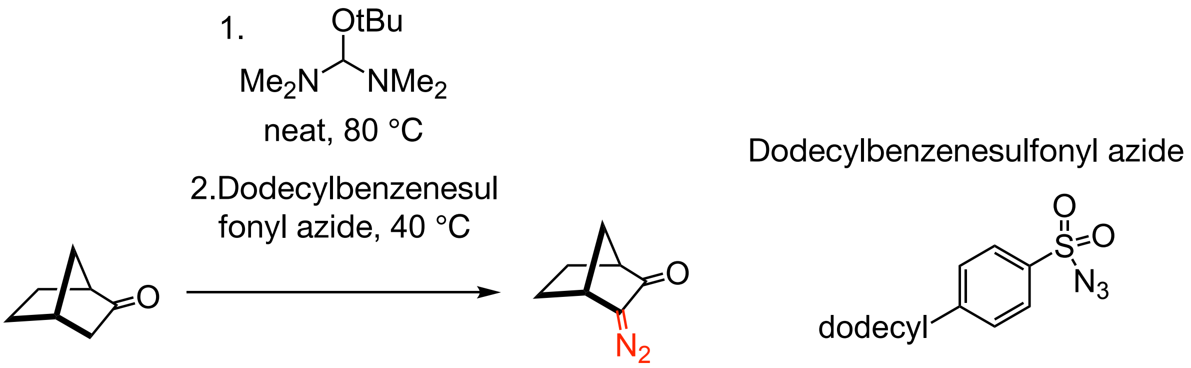

3-diazobicyclo[2.2.1]heptan-2-one (27): To a flame dried 100 ml round bottom flask equipped with a stir bar was added bicyclo[2.2.1]heptan-2-one (12 g, 1 equiv., 0.11 mol). The flask was evacuated and backfilled with N_2 (x3). Next, Bredereck's reagent (22 mL, 1 equiv., 0.11 mol) was added to the ketone dropwise. The reaction was warmed to 80 °C and stirred until the starting material was consumed (typically two days for this scale). The disappearance of the starting material was monitored by Gas chromatography. The volatile materials from the reaction mixture were evaporated in vacuo, and the crude material was passed through a small pad of alumina with 50-70% EA in hexane. The eluent was concentrated and directly used in the next step.

The crude material was dissolved in THF (550 ml, 0.15 M) and cooled to 0 °C for 5 mins before Dodecylbenzensulfonylazide (42.5 g, 40.5 mL, 1.1 equiv., 121 mmol) was added dropwise and the reaction was slowly warm to a 40 °C and stirred. Upon the disappearance of the starting material monitored by Gas chromatography (typically two days for this scale), the solvent was evaporated and the crude material was purified using flash column chromatography to afford **33** (7.3 g, 54 mmol, 49% yield) as a yellow liquid. Characterization matched that of the literature.[v] Note: Product is volatile.

4.2. Wolff rearrangement:

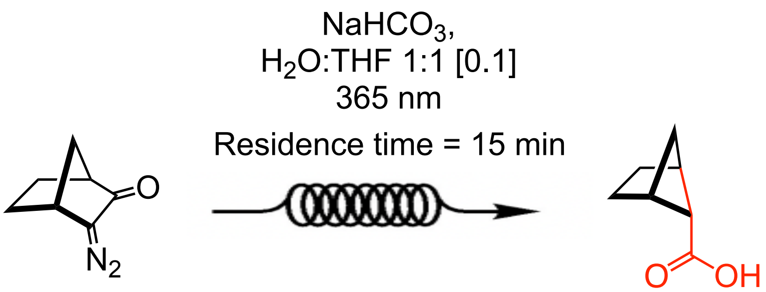

34

bicyclo[2.1.1]hexane-5-carboxylic acid (34):

In batch: 3-diazobicyclo[2.2.1]heptan-2-one **33** (77 mg, 0.57 mmol, 1 equiv.) was dissolved in 38 ml (0.015 M) THF/H_2O (1:1) in a 100 ml round bottom flask. To this solution was added sodium bicarbonate (124 mg, 1.47 mmol, 2.6 equiv.) was added and the flask was capped with a septum. Next it was sparged with N_2 for 10 mins and irradiated at 352 nm (UV-A) under N_2. The reaction mixture was irradiated for 12 hrs and disappearance of the starting material was observed by thin layer chromatography. Next, the reaction mixture was evaporated to remove the THF and washed with ethyl ether. The aqueous layer was acidified with 1 N HCl to pH 1 and extracted with 5 ml of Ethyl acetate (x5). The combined organic layers were evaporated in vacuo to afford **34** (38 mg, 0.31 mmol, 5:1 *endo:exo* 55% yield) as a yellowish viscous liquid.

In flow reactor: 3-diazobicyclo[2.2.1]heptan-2-one **33** (1.6 g, 1 equiv., 11.75 mmol) was dissolved in 115 ml (0.1 M) THF/H_2O (1:1) in a 250 ml round bottom flask. To this solution was added sodium bicarbonate (1.975 g, 2 equiv., 23.50 mmol), and the flask was capped with a septum. Next, it was sparged with N_2 for 10 mins before being pumped through the Vapourtec Flow reactor with 15 min residence time (Flow rate 0.667ml/min) and 365 nm LED light. After all the reaction solution was pumped through the flow reactor, an additional 20 ml THF/water was pumped through the reactor. The reaction mixture was evaporated to remove the THF and washed with ethyl ether and the aqueous layer was acidified with 1 N HCl to pH 1. The aqueous layer was treated with activated charcoal to remove the yellow impurity. Next, the aqueous layer was filtered to remove the activated charcoal and extracted with 30 ml of Ethyl acetate (x5) to afford bicyclo[2.1.1]hexane-5-carboxylic acid **34** (809 mg, 6.41 mmol, 55% yield, 5:1 dr) as a yellowish viscous liquid.

^{1}H NMR (500 MHz, $CDCl_3$) δ 11.00 (s, 1H), 2.79 (d, J = 2.7 Hz, 0.4H) (*minor*), 2.77 (t, J = 2.9 Hz, 2H), 2.31 (dt, J = 8.0, 2.6 Hz, 1H), 2.52 (s, 0.2H) (minor), 2.26 (d, J = 7.5 Hz, 0.2H) (*minor*), 1.83

– 1.75 (m, 2H), 1.71 (q, *J* = 8.8 Hz, 4x0.2H) (*minor*), 1.58 (q, *J* = 4.2 Hz, 2H), 1.45 (dq, *J* = 6.2, 2.7 Hz, 1H), 1.06 (t, *J* = 7.5 Hz, 0.2H) (*minor*), 0.82 (d, *J* = 6.6 Hz, 1H).

^{13}C NMR (126 MHz, $CDCl_3$) δ 180.7 (*minor*), 178.2, 54.6 (*minor*), 48.7, 43.4 (*minor*), 42.1, 37.3 (*minor*), 36.6, 27.1 (*minor*), 24.0.

IR (neat): 3133 (br), 2966 (m), 2881 (w), 1702 (s), 1284 (w).

HRMS (ESI): Calculated for $C_7H_9O_2$ $[M-H]^+$ 125.0608; Found 125.0608.

4.3. Further functionalizations of mono-substituted-[2.1.1]-bicyclohexane

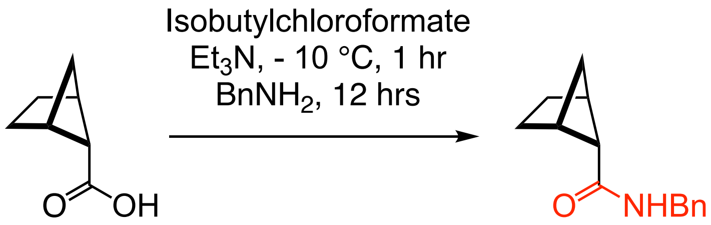

34 **35**

***N*-benzylbicyclo[2.1.1]hexane-5-carboxamide (35):** To a flame-dried 2-dram vial equipped with a stir bar and capped with a septum was added the bicyclo[2.1.1]hexane-5-carboxylic acid **34** (50 mg, 1 equiv., 0.40 mmol). The flask was evacuated and backfilled with N_2 (x3) and CH_2Cl_2 (2 ml) was added. This acid solution was cooled to -10 °C followed by addition of triethylamine (83 µL, 1.5 equiv., 0.59 mmol) and isobutyl chloroformate (72 µL 1.4 equiv., 0.55 mmol) was added dropwise. The reaction mixture was stirred at -10 °C for 1.5 hrs. and then benzylamine (65 µL, 1.5 equiv., 0.59 mmol) was added and the septum was replaced with a screw cap. The reaction was stirred 12 hrs while warming to room temperature. The reaction was quenched with sat. aq. NH_4Cl and extracted with CH_2Cl_2 (x3). The combined organic layers were dried over Na_2SO_4 and filtered. The filtrate was concentrated in vacuo and purified via flash column chromatography to afford **35** as a white solid (100 mg, 0.46 mmol, quant, 5:1 dr).

R_f: 0.5 in 70% EA/Hex.

^{1}H NMR (500 MHz, $CDCl_3$) δ 7.33 (t, *J* = 7.2 Hz, 2H), 7.30 – 7.24 (m, 4H), 5.85 (s, 0.2H) (minor), 5.45 (s, 1H), 4.49 (d, *J* = 5.8 Hz, 0.4H) (minor), 4.40 (d, *J* = 5.8 Hz, 2H), 2.73 (d, *J* = 2.8 Hz, 2H),

2.43 (s, 1H), 2.19 (m, 0.4H) (minor), 1.78 – 1.73 (m, 2H), 1.56 (q, *J* = 4.2 Hz, 2H), 1.40 (dt, *J* = 6.1, 2.7 Hz, 1H), 1.07 (t, *J* = 7.6 Hz, 0.2H) (minor), 0.82 (d, *J* = 6.6 Hz, 1H).

^{13}C NMR (126 MHz, $CDCl_3$) δ 171.7, 138.7, 128.9, 128.0, 127.6, 56.5 (minor), 50.3, 43.7 (minor), 43.3, 41.4, 37.5 (minor), 36.0, 27.2 (minor), 24.2.

IR (neat): 2961 (m), 2927(w), 2878 (m), 1644 (s), 1540 (s), 1454 (w), 1028 (w).

HRMS (EI, *m/z*): Calculated for $C_{14}H_{17}NO$ $[M]^+$ 215.1299; Found: 215.1305.

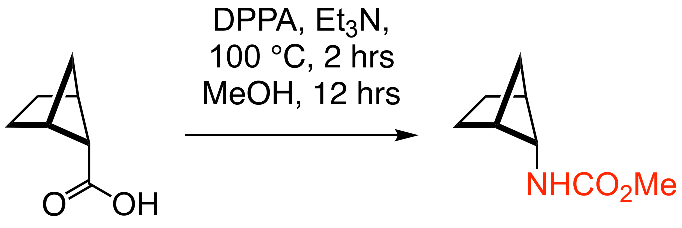

34 **36**

***N*-bicyclo[2.1.1]hexan-5-yl)acetamide (36):** To a flame-dried 2-dram vial equipped with a stir bar and capped with a septum was added bicyclo[2.1.1]hexane-5-carboxylic acid **34** (53 mg,1 equiv., 0.42 mmol). The flask was evacuated and backfilled with N_2 (x3) and CCl_4 (1.4 ml, 0.33 M) was added. Next, Et_3N (61 μL, 1.05 equiv., 0.44 mmol) was added to the vial, and the temperature was increased to give it a gentle boil. Diphenylphosphoryl azide (91 μL, 1 equiv., 0.42 mmol) was added to the reaction flask dropwise. Next, the septum was quickly replaced with a screw cap and the reaction was refluxed for 2 hrs. The reaction was brought to room temperature and methanol (20 μL, 1.2 equiv., 0.50 mmol) was added. The reaction was stirred at room temperature for 12 hrs. The reaction mixture was evaporated to dryness and dissolved in ethyl ether and quenched with sat. aq. $NaHCO_3$. The layers were separated and the aqueous layer was extracted with ethyl ether (x3). The combined organic layers were dried over an. Na_2SO_4, filtered and concentrated in vacuo. Next, the crude mixture was purified by flash column chromatography to afford **36** a yellowish semi-solid as a single diastereomer (36 mg, 0.23 mmol, 55% yield, >20:1dr).

R_f: 0.5 in 20% EA/Hex.

^{1}H NMR (500 MHz, CDCl$_3$) δ 4.60 (s, 1H), 3.63 (s, 3H), 3.59 – 3.49 (m, 1H), 2.52 (s, 2H), 1.55 (t, *J* = 5.7 Hz, 2H), 1.48 – 1.37 (m, 2H), 1.29 – 1.16 (m, 1H), 0.73 (d, *J* = 7.3 Hz, 1H).

^{13}C NMR (126 MHz, CDCl$_3$) δ 156.4, 52.2, 52.0, 41.9, 32.0, 22.4.

IR (neat): 3327 (br), 2969 (m), 2880 (w), 1699 (s), 1525 (m), 1242 (m).

HRMS (ESI): Calculated for $C_8H_{14}O_2N$ $[M+H]^+$ 156.1019; Found 156.1019.

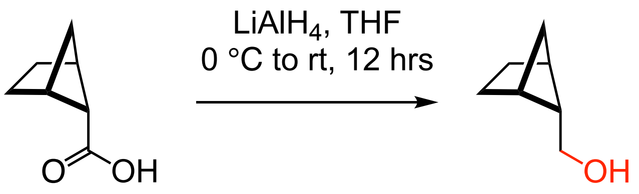

34 **37**

bicyclo[2.1.1]hexan-5-yl-methanol (37): To a flame-dried 20-dram vial equipped with a stir bar and fitted with a septum was added bicyclo[2.1.1]hexane-5-carboxylic acid **34** (200 mg, 1 equiv., 1.59 mmol). The flask was evacuated and backfilled with N_2 (x3). Next, carboxylic acid **34** was dissolved in THF (8 ml) and cooled to 0 °C. To this solution, $LiAlH_4$ (72.2 mg, 1.2 equiv., 1.90 mmol) was added in portions. Next, the reaction was slowly warmed to room temperature and stirred for 4 hrs. Upon completion, the reaction was quenched with sat. aq. NaOH solution, and the heterogeneous mixture was passed through a pad of Celite. The crude reaction mixture was evaporated in vacuo and purified by flash column chromatography to afford **37** as a colourless oil (150 mg, 1.34 mmol, 84% yield, 5:1 dr).

R$_f$: 0.5 in 30% EA/Hex

^{1}H NMR (500 MHz, CDCl$_3$) δ 3.86 (dd, *J* = 7.9, 1.1 Hz, 0.4H) (minor), 3.30 (dd, *J* = 7.2, 1.2 Hz, 2H), 2.45 (t, *J* = 2.8 Hz, 2H), 2.35 (d, *J* = 2.8 Hz, 0.4H) (minor), 2.07 (d, *J* = 7.2 Hz, 0.2H) (minor), 1.84 (td, *J* = 7.1, 3.5 Hz, 1H), 1.58 – 1.44 (m, 4H), 1.37 – 1.32 (m, 1H), 0.97 (t, *J* = 7.1 Hz, 0.2H) (minor), 0.80 (d, *J* = 6.5 Hz, 1H).

^{13}C NMR (126 MHz, CDCl$_3$) δ 62.1 (minor), 60.1, 54.7 (minor), 47.9, 40.2 (minor), 39.7, 37.2, 35.9 (minor), 27.5, 23.1 (minor).

IR (neat) 3322 (b), 2959 (m), 2878 (w), 1025 (s).

HRMS (EI, *m/z*): Calculated for $C_7H_{11}O$ $[M-H]^+$ 110.0726; Found: 110.0725.

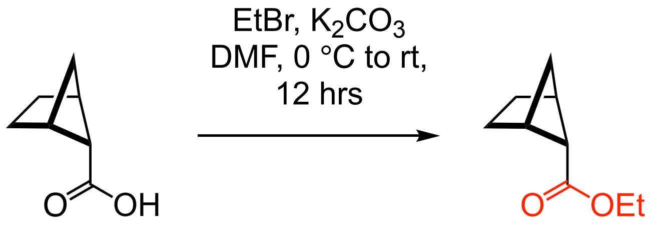

34 **44**

ethyl bicyclo[2.1.1]hexane-5-carboxylate (44): To a flame-dried 20-dram vial equipped with a stir bar and capped with a septum was added bicyclo[2.1.1]hexane-5-carboxylic acid **34** (400 mg, 1 equiv., 3.17 mmol). The flask was evacuated and backfilled with N_2 (x3) and DMF (9 ml, 0.33 molar) was added. Next, the reaction was cooled to 0 °C and K_2CO_3 (1.10 g, 2.5 equiv., 7.93 mmol) was added to the solution. Ethyl bromide (0.7 ml, 3 equiv., 9.51 mmol) was added and the reaction was brought to room temperature. The reaction was stirred for 3-4 hrs and then quenched with saturated NH_4Cl solution. The aqueous layer extracted with 10 ml ethyl ether (x3). The combined organic layers were dried over Na_2SO_4 and filtered. The filtrate was concentrated in vacuo and purified via flash column chromatography to afford **44** (400 mg, 2.59 mmol, 82% yield, 5:1 dr) as a colourless oil. Note: The product is very volatile.

R_f: 0.5 in 10% Ethyl ether/Hex.

^{1}H NMR (500 MHz, $CDCl_3$) δ 4.16 (q, *J* = 7.2 Hz, 0.4H) (minor), 4.12 – 4.02 (m, 2H), 2.76 (d, *J* = 2.6 Hz, 0.2H) (minor), 2.74 (d, *J* = 2.6 Hz, 2H), 2.45 (s, 1H), 2.26 – 2.20 (m, 0.2H) (minor), 2.19 (d, *J* = 7.6 Hz, 0.2H) (minor), 1.70 (dd, *J* = 13.2, 7.6 Hz, 2H major+minor), 1.59 – 1.51 (m, 2H), 1.42 (dq, *J* = 7.0, 2.5 Hz, 1H), 1.29 – 1.25 (m, 0.6H) (minor), 1.23 (t, *J* = 7.2 Hz, 3H), 1.03 (t, *J* = 7.5 Hz, 0.2H) (minor), 0.79 (d, *J* = 6.6 Hz, 1H).

^{13}C NMR (126 MHz, $CDCl_3$) δ 174.0 (minor), 171.8, 60.3 (minor), 59.8, 54.8 (minor), 53.5 (minor), 48.9, 43.4 (minor), 41.9, 37.3 (minor), 36.4, 27.1 (minor), 24.0, 14.4.

IR (neat) 2979 (m), 2881 (w), 1731 (s), 1244 (m), 1017 (m).

HRMS (EI, *m/z*) Calculated for C_6H_9 $[M-C_3H_5O_2]^+$ 81.0704; Found: 81.0702.

5. Directed C-H functionalization:

5.1. General procedure D:

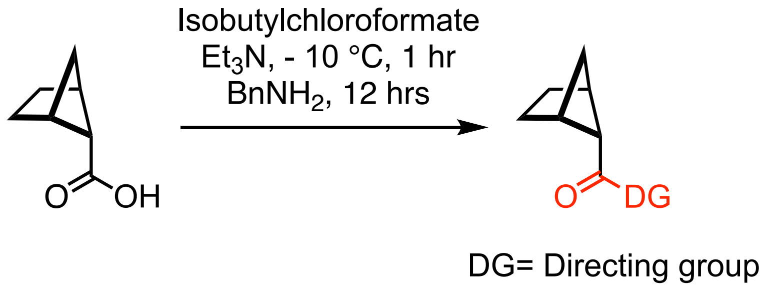

To a flame dried 2-dram vial equipped with a stir bar and capped with a septum was added the acid (1 equiv.) dissolved in CH_2Cl_2 (0.5 M). This acid solution was cooled down to –10 °C followed by Et_3N (1.3 equiv.) and isobutyl chloroformate (1.2 equiv.) was added dropwise to the reaction mixture. The reaction mixture was stirred at -10 °C for 1.5 hrs. and then amine (1.3 equiv.) was added to the reaction mixture and the septum was replaced with a screw cap. The reaction was stirred 12 hrs while warming to the room temperature. Next day, the reaction was quenched with sat NH_4Cl and the aqueous layer was extracted with CH_2Cl_2 (*3 times). The combined organic layer was dried over Na_2SO_4 and filtered. The filtrate was concentrated in vacuo and purified via flash column chromatography to afford the amide.

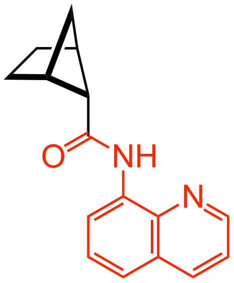

38

***N*-(quinolin-8-yl)bicyclo[2.1.1]hexane-5-carboxamide (38):** The compound **38** was prepared according to general procedure D using bicyclo[2.1.1]hexane-5-carboxylic acid (500 mg, 3.96 mmol) and purified by flash column chromatography to afford as a yellow solid (640 mg, 2.54 mmol, 64% yield, 5:1 dr)

R_f: 0.6 in 20% EA/Hex.

^{1}H NMR (500 MHz, CDCl$_3$) δ 10.00 (s, 0.2H) (minor), 9.63 (s, 1H), 8.89 – 8.69 (m, 2H), 8.15 (dd, *J* = 8.3, 1.9 Hz, 1H), 7.58 – 7.41 (m, 3H), 2.98 (t, *J* = 2.8 Hz, 2H), 2.75 (s, 1H), 2.54 (d, *J* = 7.4 Hz, 0.2H) (minor), 2.38 (dq, *J* = 7.5, 2.5 Hz, 0.2H) (minor), 1.95 – 1.88 (m, 2H), 1.87 – 1.76 (m, 0.8H) (minor), 1.73 – 1.59 (m, 2H), 1.59 – 1.46 (m, 1H), 1.16 (t, *J* = 7.6 Hz, 0.2H) (minor), 0.91 (d, *J* = 6.5 Hz, 1H).

^{13}C NMR (126 MHz, CDCl$_3$) δ 171.9 (minor), 170.8, 148.3, 148.3 (minor), 138.6, 138.5, 136.5 (minor), 136.4, 134.7, 134.5, 128.1, 128.1 (minor), 127.6 (minor), 127.5, 121.7 (minor), 121.7, 121.4 (minor), 121.3, 116.5 (minor), 116.4, 58.0 (minor), 51.6, 43.5 (minor), 41.8, 37.7 (minor), 36.0, 27.3 (minor), 24.2.

IR (neat): 3351 (w), 2966 (w), 1684 (m), 1524 (s), 1484 (m), 1324 (m).

HRMS (ESI): Calculated for $C_{16}H_{16}ON_2Na$ $[M+Na]^+$ 275.1155; Found 275.1156.

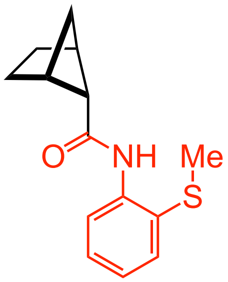

SI-15

***N*-(2-(methylthio)phenyl)bicyclo[2.1.1]hexane-5-carboxamide (SI-15):** Compound **SI-15** was prepared according to general procedure D and purified by silica gel chromatography to afford a reddish solid (100 mg, 0.4 mmol, 50% yield, 5:1 dr).

R_f: 0.4 in 30% EA/Hex.

^{1}H NMR (500 MHz, CDCl$_3$) δ 8.32 (d, *J* = 8.3 Hz, 1H), 8.20 – 8.05 (m, 1H), 7.47 (dd, *J* = 7.8, 1.5 Hz, 1H), 7.32 – 7.27 (m, 1H), 7.04 (td, *J* = 7.7, 1.4 Hz, 1H), 2.89 (d, *J* = 3.1 Hz, 2H), 2.64 (s, 1H), 2.38 (s, 4H), 1.84 (dd, *J* = 7.2, 2.5 Hz, 2H), 1.81 – 1.72 (m, 0.7H) (minor), 1.64 (t, *J* = 5.4 Hz, 2H), 1.50 (dt, *J* = 6.9, 2.7 Hz, 1H), 1.15 (t, *J* = 7.6 Hz, 0.14 H) (minor), 0.89 (d, *J* = 6.6 Hz, 1H).

^{13}C NMR (126 MHz, CDCl$_3$) ^{13}C NMR (126 MHz, CDCl$_3$) δ 170.3, 138.4, 133.1, 129.1, 124.9, 124.2, 120.4, 51.5, 43.4, 41.6, 35.9, 27.2, 24.1, 19.2.

IR (neat): 3339 (w), 2961 (w), 1689 (m), 1578 (m), 1508 (s) 1297 (m).

5.2. General procedure E: Directed C-H functionalization reaction.

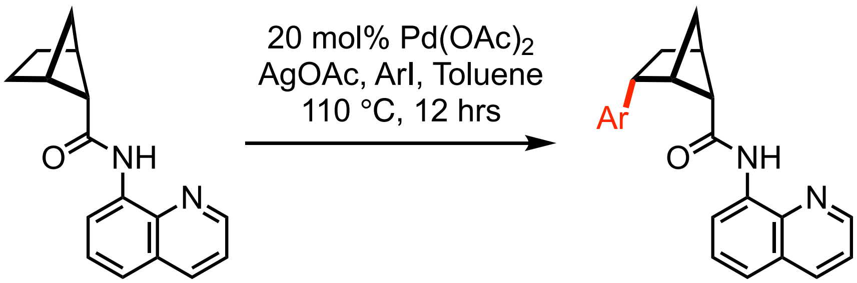

To a flame dried 2-dram vial equipped with a stir bar and capped with a septum was taken inside the glovebox. To this Pd(OAc)$_2$ (0.2 equiv.), AgOAc (3 equiv.) and *N*-(quinolin-8-yl) bicyclo [2.1.1]-hexane-5-carboxamideamidoquinoline **38** (1 equiv.) were added. The vial was taken out of the glovebox and the starting materials were dissolved in toluene (0.3 M). Next, iodoarene (3 equiv.) was added to the reaction mixture, and the septum was replaced with a screw cap. The reaction mixture was stirred at 110 °C for 12 hrs. The crude reaction mixture was passed through a pad of Celite and purified by flash column chromatography.

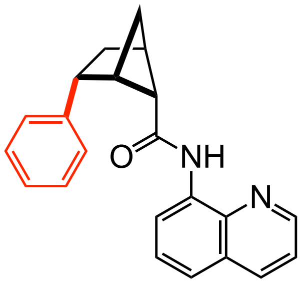

39a

2-phenyl-*N*-(quinolin-8-yl)bicyclo[2.1.1]hexane-5-carboxamide (39a): The compound was prepared according to the general procedure E using **38** (26 mg, 0.10 mmol), iodobenzene (3 equiv.) and was purified by flash column chromatography to afford **39a** as a yellowish solid (18 mg, 53% yield, >20:1 dr). Relative stereochemistry was confirmed by X-ray analysis.

R$_f$: 0.7 in 20% EA/Hex.

^{1}H NMR (500 MHz, CDCl$_3$) δ 9.18 (s, 1H), 8.72 (dd, *J* = 4.2, 1.6 Hz, 1H), 8.20 (dd, *J* = 7.2, 1.7 Hz, 1H), 8.08 (dd, *J* = 8.2, 1.7 Hz, 1H), 7.40 (dd, *J* = 8.3, 4.2 Hz, 1H), 7.38 – 7.30 (m, 2H), 7.19 (d, *J* = 7.7 Hz, 2H), 6.62 (t, *J* = 7.6 Hz, 2H), 6.39 (t, *J* = 7.3 Hz, 1H), 3.52 – 3.43 (m, 2H), 3.08 – 3.02 (m, 1H), 2.77 (d, *J* = 3.0 Hz, 1H), 2.66 (dt, *J* = 10.9, 3.5 Hz, 1H), 2.22 – 2.15 (m, 1H), 1.67 (dq, *J* = 5.6, 2.7 Hz, 1H), 1.19 (d, *J* = 6.4 Hz, 1H).

^{13}C NMR (126 MHz, CDCl$_3$) δ 170.0, 147.8, 142.4, 138.3, 136.1, 134.1, 127.7, 127.5, 127.5, 127.3, 124.8, 121.3, 120.8, 116.1, 50.6, 48.4, 43.3, 41.3, 38.0, 28.9.

IR (neat): 3351 (br), 2953 (w), 2881 (w), 1685 (m), 1523 (s), 1484 (m), 1262 (m).

HRMS (ESI): Calculated for $C_{22}H_{20}ON_2Na$ $[M+Na]^+$ 351.1468; Found: 351.1471.

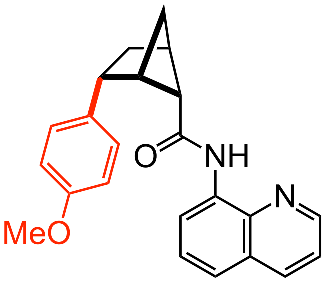

39b

2-(4-methoxyphenyl)-*N*-(quinolin-8-yl)bicyclo[2.1.1]hexane-5-carboxamide (39b): The compound was prepared according to general procedure E using **38** (26 mg, 0.10 mmol), 1-iodo-4-methylbenzene (3 equiv.) and was purified by flash column chromatography to afford **39b** as a yellowish solid (19.3 mg, 0.052 mmol, 52% yield, >20:1 dr).

R$_f$: 0.7 in 40% EA/Hex

^{1}H NMR (400 MHz, CDCl$_3$) δ 9.12 (s, 1H), 8.72 (dd, *J* = 4.2, 1.7 Hz, 1H), 8.28 (dd, *J* = 5.3, 3.7 Hz, 1H), 8.08 (dd, *J* = 8.3, 1.7 Hz, 1H), 7.40 (dd, *J* = 8.2, 4.2 Hz, 1H), 7.39 – 7.33 (m, 2H), 7.13 – 7.05 (m, 2H), 6.16 – 6.07 (m, 2H), 3.41 (td, *J* = 3.8, 1.8 Hz, 2H), 3.15 (s, 3H), 3.04 (d, *J* = 6.4 Hz, 1H), 2.75 (s, 1H), 2.59 (s, 1H), 2.13 (d, *J* = 2.0 Hz, 1H), 1.64 (dt, *J* = 5.5, 2.8 Hz, 1H), 1.17 (dd, *J* = 6.5, 0.9 Hz, 1H).

^{13}C NMR (101 MHz, CDCl$_3$) δ 170.2, 156.8, 147.8, 138.3, 136.0, 134.5, 134.3, 128.4, 127.7, 127.3, 121.4, 120.7, 116.0, 113.0, 54.7, 50.6, 48.8, 42.5, 41.3, 37.9, 28.7.

IR (neat): 3349 (br), 2951 (w), 2880 (w), 1684 (m), 1524 (s), 1484 (m), 1384 (m).

HRMS (ESI): Calculated for $C_{23}H_{23}O_2N_2$ $[M+H]^+$ 359.1754; Found: 359.1755.

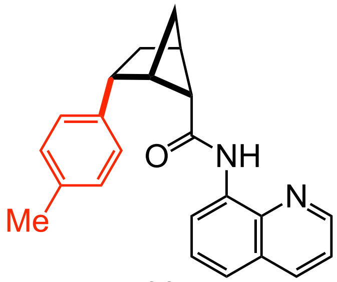

39c

***N*-(quinolin-8-yl)-2-(*p*-tolyl)bicyclo[2.1.1]hexane-5-carboxamide (39c):** The compound was prepared according to general procedure E **38** (26 mg, 0.10 mmol), and was purified by flash column chromatography to afford **39c** as a yellow solid (15.3 mg, 0.043 mmol, 43% yield, >20:1 dr).

R$_f$: 0.8 in 20% EA/Hex.

^{1}H NMR (500 MHz, CDCl$_3$) δ 9.10 (s, 1H), 8.72 (dd, *J* = 4.2, 1.7 Hz, 1H), 8.25 (dd, *J* = 6.1, 2.9 Hz, 1H), 8.08 (dd, *J* = 8.2, 1.7 Hz, 1H), 7.40 (dd, *J* = 8.2, 4.2 Hz, 1H), 7.38 – 7.30 (m, 2H), 7.05 (d, *J* = 7.7 Hz, 2H), 6.36 (d, *J* = 7.8 Hz, 2H), 3.45 – 3.38 (m, 2H), 3.04 (ddd, *J* = 5.9, 3.6, 1.9 Hz, 1H), 2.75 (d, *J* = 2.9 Hz, 1H), 2.66 – 2.59 (m, 1H), 2.12 (ddd, *J* = 11.3, 9.2, 1.5 Hz, 1H), 1.63 (tt, *J* = 6.1, 3.0 Hz, 1H), 1.56 (s, 3H), 1.17 (d, *J* = 6.4 Hz, 1H).

^{13}C NMR (126 MHz, CDCl$_3$) δ 170.2, 147.7, 139.3, 138.3, 136.0, 134.3, 134.3, 128.2, 127.7, 127.3, 121.3, 120.7, 116.0, 50.6, 48.6, 42.9, 41.3, 37.8, 28.5, 20.3.

IR (neat): 3349 (br), 2951 (w), 2880 (w), 1648 (m), 1524 (s), 1484 (m), 1325 (m).

HRMS (ESI): Calculated for $C_{23}H_{22}ON_2Na$ $[M+Na]^+$ 365.1624; Found: 365.1630.

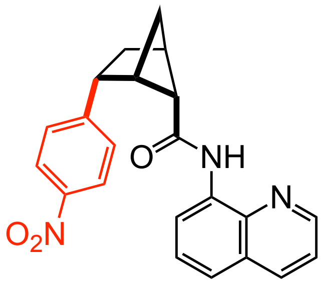

39d

2-(4-nitrophenyl)-*N*-(quinolin-8-yl)bicyclo[2.1.1]hexane-5-carboxamide (39d): The compound was prepared according to general procedure E **38** (26 mg, 0.10 mmol), 1-iodo-4-nitrobenzene (3 equiv.) and was purified by flash column chromatography to obtain **39d** as a yellow solid (21 mg, 0.055 mmol, 55% yield).

R_f: 0.5 in 40% EA/Hex

^{1}H NMR (600 MHz, $CDCl_3$) δ 9.14 (s, 1H), 8.68 (dd, *J* = 4.2, 1.7 Hz, 1H), 8.16 (dd, *J* = 7.1, 1.9 Hz, 1H), 8.06 (dd, *J* = 8.3, 1.7 Hz, 1H), 7.39 (dd, *J* = 8.2, 4.2 Hz, 1H), 7.37 – 7.22 (m, 6H), 3.46 (dt, *J* = 5.5, 3.4 Hz, 2H), 3.12 – 3.01 (m, 1H), 2.81 (d, *J* = 3.4 Hz, 1H), 2.67 (ddd, *J* = 12.9, 4.1, 2.1 Hz, 1H), 2.24 – 2.16 (m, 1H), 1.71 (dq, *J* = 5.6, 2.7 Hz, 1H), 1.21 (d, *J* = 6.5 Hz, 1H).

^{13}C NMR (126 MHz, $CDCl_3$) δ 169.6, 150.5, 148.0, 145.0, 137.9, 136.5, 133.5, 128.4, 127.8, 127.2, 125.2, 122.7, 121.6, 115.9, 50.4, 48.7, 43.7, 41.3, 37.9, 28.7.

IR (neat): 3342 (br), 2977 (w), 1682 (m), 1514(s), 1483 (m), 1343 (m), 1262 (m).

HRMS (ESI): Calculated for $C_{22}H_{19}O_3N_3Na$ $[M+Na]^+$ 396.1319; Found: 396.1319.

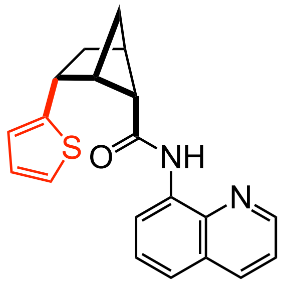

43

N-(quinolin-8-yl)-2-(thiophen-2-yl)bicyclo[2.1.1]hexane-5-carboxamide (43): The compound was prepared according to general procedure E using **38** (26 mg, 0.10 mmol), 2-Iodo thiophene (3 equiv.) and was purified by flash column chromatography to afford **43** as a brown solid (17 mg, 0.051 mmol, 49% yield, >20:1 dr).

R_f: 0.6 in 20% EA/Hex.

^{1}H NMR (600 MHz, $CDCl_3$) δ 9.24 (s, 1H), 8.74 (dd, *J* = 4.2, 1.7 Hz, 1H), 8.35 (t, *J* = 4.5 Hz, 1H), 8.08 (dd, *J* = 8.2, 1.7 Hz, 1H), 7.44 – 7.35 (m, 3H), 6.75 – 6.62 (m, 1H), 6.42 (d, *J* = 5.0 Hz, 1H), 6.15 (dd, *J* = 5.1, 3.5 Hz, 1H), 3.54 (dd, *J* = 8.9, 4.2 Hz, 1H), 3.40 – 3.33 (m, 1H), 3.06 (ddt, *J* = 6.5, 2.8, 1.4 Hz, 1H), 2.81 (d, *J* = 3.1 Hz, 1H), 2.68 – 2.59 (m, 1H), 2.26 (ddt, *J* = 11.5, 8.8, 1.5 Hz, 1H), 1.65 (dq, *J* = 5.6, 2.7 Hz, 1H), 1.16 (d, *J* = 6.6 Hz, 1H).

^{13}C NMR (126 MHz, $CDCl_3$) δ 169.9, 147.9, 147.4, 138.4, 136.1, 134.4, 127.8, 127.3, 126.1, 123.8, 122.7, 121.4, 120.4, 116.0, 50.8, 50.1, 41.1, 39.5, 37.7, 31.0.

IR (neat): 2924 (s), 1732 (m), 1526 (s), 1486 (m), 1326 (m).

HRMS (ESI): Calculated for $C_{20}H_{19}ON_2S$ $[M+H]^+$ 335.1213; Found: 335.1213.

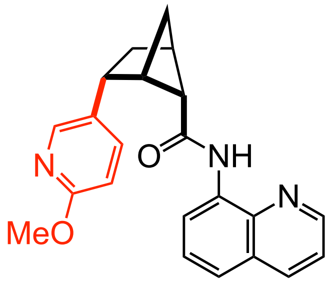

44

2-(6-methoxypyridin-3-yl)-N-(quinolin-8-yl)bicyclo[2.1.1]hexane-5-carboxamide (44): The compound was prepared according to general procedure E using **38** (26 mg, 0.10 mmol), 4-iodo-2-methoxy-pyridine (3 equiv.) and was purified by flash column chromatography to afford **44** as a brownish solid (20 mg, 0.054 mmol, 54% yield, >20:1 dr).

R_f: 0.5 in 50% EA/Hex

^{1}H NMR (500 MHz, $CDCl_3$) δ 9.19 (s, 1H), 8.72 (dd, *J* = 4.2, 1.8 Hz, 1H), 8.27 (dd, *J* = 6.2, 2.8 Hz, 1H), 8.09 (dd, *J* = 8.2, 1.8 Hz, 1H), 7.99 (d, *J* = 2.6 Hz, 1H), 7.45 – 7.31 (m, 4H), 5.96 (d, *J* = 8.5 Hz, 1H), 3.42 – 3.35 (m, 2H), 3.33 (s, 2H), 3.10 – 3.01 (m, 1H), 2.78 (s, 1H), 2.62 (dt, *J* = 11.4, 3.1 Hz, 1H), 2.15 (t, *J* = 10.2 Hz, 1H), 1.71 – 1.63 (m, 2H), 1.18 (d, *J* = 6.5 Hz, 1H).

^{13}C NMR (126 MHz, $CDCl_3$) δ 169.9, 161.8, 148.0, 145.8, 138.2, 138.1, 136.2, 133.9, 130.3, 127.8, 127.3, 121.5, 121.0, 116.1, 109.3, 53.0, 52.8, 50.5, 48.5, 41.3, 40.5, 37.7, 28.3.

IR (neat): 3346 (br), 2922 (w), 1684 (m), 1523 (s), 1484 (m), 1265 (m).

HRMS (ESI): Calculated for $C_{22}H_{21}O_2N_3Na$ $[M+Na]^+$ 382.1526; Found: 382.1528

5.3. Removal of 8-aminoquinoline directing group:

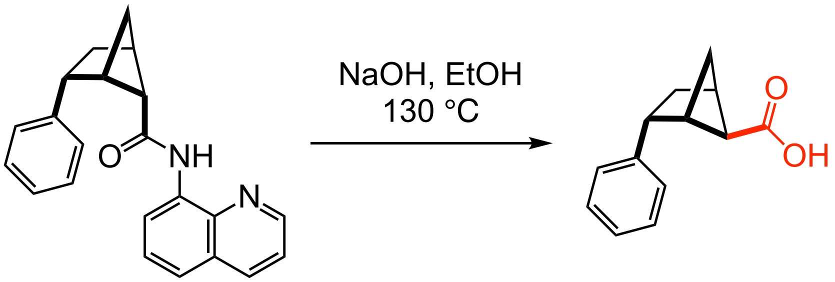

39a **40**

2-phenylbicyclo[2.1.1]hexane-5-carboxylic acid (40): A 2-dram vial was charged with 2-phenyl-*N*-(quinolin-8-yl)bicyclo[2.1.1]hexane-5-carboxamide **39a** (18 mg, 1 equiv., 55 µmol), NaOH (33 mg, 15 equiv., 0.82 mmol), and ethanol 0.23 ml (0.24 M). The flask was sealed and placed in a pre-heated oil bath (130 ºC) and stirred for 18 h. The reaction mixture was cooled to room temperature, and the solvent was removed in vacuo. The crude residue was diluted with water and the aqueous layer was washed with ethyl ether Next, the aqueous layer was acidified with 1 N HCl to pH 1 and extracted with EtOAc (3 times). The combined organic layers were dried over Na_2SO_4, filtered, and concentrated in vacuo to give **40** as a yellowish solid (10 mg, 90% yield).

^{1}H NMR (500 MHz, $CDCl_3$) δ 7.25 (t, *J* = 7.5 Hz, 2H), 7.19 (dd, *J* = 5.3, 3.2 Hz, 3H), 7.13 (t, *J* = 7.3 Hz, 1H), 3.38 (dd, *J* = 9.3, 3.9 Hz, 1H), 3.05 (ddd, *J* = 6.4, 3.1, 1.6 Hz, 1H), 2.85 – 2.78 (m, 1H), 2.50 – 2.35 (m, 2H), 2.30 – 2.21 (m, 1H), 1.89 – 1.80 (m, 1H), 1.32 – 1.27 (m, 1H).

^{13}C NMR (126 MHz, $CDCl_3$) δ 179.4, 144.2, 128.6, 127.5, 126.0, 51.4, 47.3, 43.6, 43.6, 38.9, 35.2.

IR (neat): 3105 (br), 2981 (w), 2886 (w), 1698 (s), 1495 (w), 1293 (w).

HRMS (EI, *m/z*): Calculated for $C_{13}H_{14}O_2$ $[M]^+$ 202.0994; Found 202.0998.

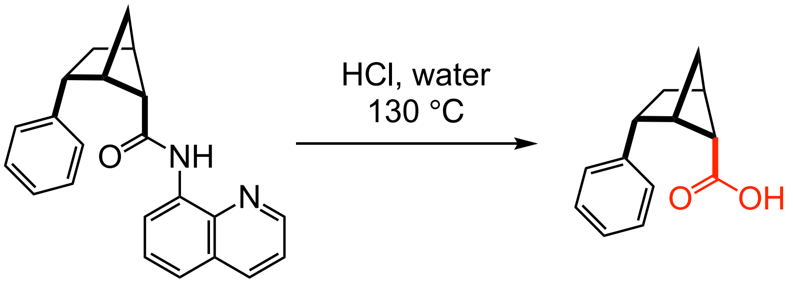

39a **41**

2-phenylbicyclo[2.1.1]hexane-5-carboxylic acid (41): A 2-dram vial was charged with 2-phenyl-N-(quinolin-8-yl)bicyclo[2.1.1]hexane-5-carboxamide (25 mg, 1 equiv., 76 µmol) and HCl (2.8 mg, 1.5 mL, 0.05 molar, 1 equiv., 76 µmol) The flask was sealed and placed in a pre-heated oil bath (130 ºC) and stirred for 18 h. The reaction mixture was cooled to room temperature, and the solvent was removed in vacuo. The crude residue was diluted with water and the aqueous layer was extracted with EtOAc (3 times). The combined organic layers were dried over Na_2SO_4, filtered, and concentrated in vacuo to give **41** as a yellowish solid (11 mg, 0.054 mmol, 71 %)

^{1}H NMR (500 MHz, $CDCl_3$) δ 7.26 – 7.23 (m, 2H), 7.20 (dd, *J* = 8.5, 6.8 Hz, 2H), 7.11 (ddq, *J* = 8.5, 6.9, 1.2 Hz, 1H), 3.42 – 3.34 (m, 2H), 2.80 (ddt, *J* = 6.4, 2.7, 1.3 Hz, 1H), 2.45 (dtt, *J* = 11.2, 2.7, 1.5 Hz, 2H), 2.19 (ddt, *J* = 10.9, 9.2, 1.5 Hz, 1H), 1.63 – 1.55 (m, 1H), 1.09 (dd, *J* = 6.6, 0.9 Hz, 1H).

^{13}C NMR (126 MHz, $CDCl_3$) δ 174.7, 142.7, 127.9, 127.7, 125.8, 47.7, 47.0, 42.8, 41.1, 38.4, 30.0.

IR (neat): 3058 (br), 2981 (w), 1703 (s), 1496 (w), 1255 (m).

HRMS(EI, *m/z*): Calculated for $C_{13}H_{14}O_2$ $[M]^+$ 202.0994; Found 202.0986.

6. Photochemical C-H activation

6.1. General procedure F:

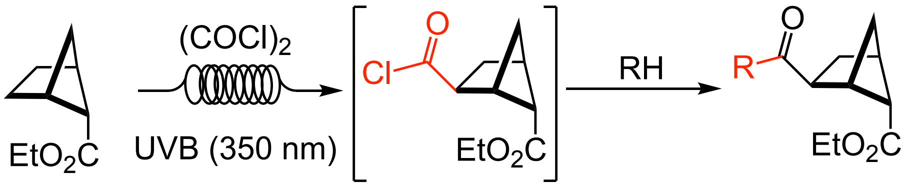

44 **45** **46a-d**

To a flame dried 20-dram vial equipped with a stir bar and capped with a septum was added the ester **44** (1 equiv.) and dissolved in CH_2Cl_2 (0.1 M). Next, oxalyl chloride (20 equiv.) was added, and the vial was capped with a screw cap. The reaction mixture was pumped through Vapourtec easy photochem flow reactor with 1ml/min (Resident time= 10 min) in a re-circulating manner for 6 hrs with UV-B irradiation. The reaction mixture was cooled by using an ice bath during the whole time. the Next, the excess oxalyl chloride was evaporated, and the crude product **45** was used immediately in the next reaction. Note: The starting material is very volatile.

To a flame dried 2-dram vial equipped with a stir bar and capped with a septum was added the crude reaction mixture (1 equiv..). It was dissolved in CH_2Cl_2 (0.3 M). This acid chloride solution was cooled down to 0 °C followed by dropwise addition of (5 equiv.). Next, the nucleophile (3 equiv.) was added to the reaction mixture. The reaction was stirred 12 hrs while warming to room temperature. The reaction was quenched with sat. aq. NH_4Cl and the aqueous layer was extracted with CH_2Cl_2 (x3). The combined organic layers were dried over Na_2SO_4 and filtered. The filtrate was evaporated in vacuo and purified via flash column chromatography.

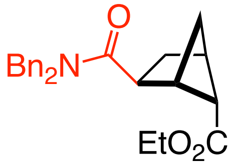

46a

ethyl-2-(dibenzylcarbamoyl)bicyclo[2.1.1]hexane-5-carboxylate (46a): The compound was prepared according to the general procedure F using benzyl amine (3 equiv.) as nucleophile and was purified by flash column chromatography (70 mg, 0.19 mmol, 37% yield, >20:1 dr).

R_f: 0.5 in 10% EA/Hex.

^{1}H NMR (500 MHz, $CDCl_3$) δ 7.38 – 7.07 (m, 10H + $CDCl_3$ peak), 4.58 – 4.46 (m, 4H), 3.92 (q, *J* = 7.2 Hz, 2H), 3.14 (t, *J* = 6.5 Hz, 1H), 2.89 (dd, *J* = 6.3, 3.0 Hz, 1H), 2.70 (d, *J* = 6.2 Hz, 1H), 2.56 (d, *J* = 3.0 Hz, 1H), 2.03 – 1.97 (m, 2H), 1.53 – 1.47 (m, 1H), 1.44 (d, *J* = 7.1 Hz, 1H), 1.02 (td, *J* = 7.0, 1.9 Hz, 3H).

^{13}C NMR (126 MHz, $CDCl_3$) δ 174.9, 171.2, 137.6, 136.7, 129.0, 128.7, 128.7, 128.2, 128.2, 127.7, 127.4, 126.8, 601, 50.5, 49.7, 47.7, 45.4, 41.0, 39.2, 34.1, 29.8, 14.2.

IR (neat) 3028 (w), 2982 (w), 1726 (s), 1644(s), 1424(m), 1207(s).

HRMS (ESI): Calculated for $C_{24}H_{27}O_3NNa$ $[M+Na]^+$ 400.1883 Found: 400.1881.

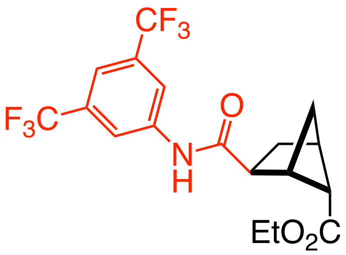

46b

ethyl-2-((3,5-bis(trifluoromethyl)phenyl)carbamoyl)bicyclo[2.1.1]hexane-5-carboxylate (46b): The compound was prepared according to the general procedure F 3,5-Bis-trifluoromethylaniline (3 equiv.) as nucleophile and was purified by flash column chromatography to afford an off-white solid (54 mg, 0.13 mmol, 26% yield, >20:1 dr).

R_f: 0.5 in 10% EA/Hex

^{1}H NMR (500 MHz, $CDCl_3$) δ 8.05 (s, 2H), 7.98 – 7.84 (m, 1H), 7.57 (s, 1H), 4.18 – 4.05 (m, 2H), 3.11 (dd, *J* = 8.9, 3.9 Hz, 1H), 2.99 (dq, *J* = 6.3, 2.4 Hz, 1H), 2.80 (ddp, *J* = 5.7, 2.8, 1.4 Hz, 1H), 2.70 (dt, *J* = 3.9, 2.2 Hz, 1H), 2.12 (dd, *J* = 11.5, 4.1 Hz, 1H), 2.04 (dddd, *J* = 12.8, 8.7, 2.7, 1.3 Hz, 1H), 1.51 (ddt, *J* = 7.6, 2.8, 1.4 Hz, 1H), 1.38 (d, *J* = 7.4 Hz, 1H), 1.25 (td, *J* = 7.2, 1.7 Hz, 3H).

^{13}C NMR (126 MHz, CDCl$_3$) δ 173.5, 171.3, 139.7, 132.8 (q, J = 34 Hz), 126.5 (q, J = 273 Hz), 119.4, 117.4, 60.5, 51.1, 45.7, 43.2, 41.4, 33.5, 28.6, 14.4, 14.4.

IR (neat) 2991 (w), 1730 (m), 1700 (m), 1559 (m), 1472 (m), 1275 (m).

HRMS (ESI) Calculated for $C_{18}H_{18}O_3NF_6$ $[M+H]^+$ 410.1185 Found: 410.1188.

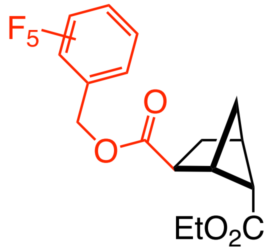

46c

Ethyl 2-(2-(perfluorophenoxy)acetyl)bicyclo[2.1.1]hexane-5-carboxylate (46c): The compound was prepared according to the general procedure F (Pentafluorophenyl)methanol (3 equiv.) and was purified by flash column chromatography (66 mg, 0.17 mmol, 35% yield, >20:1 dr).

R_f: 0.5 in 5% EA/Hex.

^{1}H NMR (500 MHz, CDCl$_3$) δ 5.20 (d, J = 1.8 Hz, 2H), 4.12 – 4.00 (m, 2H), 3.08 – 3.02 (m, 1H), 2.92 (td, J = 4.1, 2.1 Hz, 1H), 2.73 (ddt, J = 6.3, 2.9, 1.5 Hz, 1H), 2.61 (q, J = 2.5 Hz, 1H), 2.04 (dddd, J = 10.4, 9.1, 2.6, 1.4 Hz, 1H), 1.91 (ddt, J = 11.7, 3.6, 1.6 Hz, 1H), 1.46 (dp, J = 7.2, 2.5 Hz, 1H), 1.21 (t, J = 7.1 Hz, 3H), 1.08 (d, J = 7.5 Hz, 1H).

^{13}C NMR (126 MHz, CDCl$_3$) δ 174.6, 170.8, 146.9 – 144.7 (dm, J = 252 Hz), 144.9 – 140.7 (dm, J = 256 Hz) , 138.8 – 136.5 (dm, J = 270 Hz), 109.8, 60.3, 53.5, 50.5, 45.1, 41.2, 40.8, 33.9, 28.8, 14.4.

IR (neat): 2991 (w), 1730 (m), 1700 (m), 1558 (m), 1472 (m), 1380 (m), 1275 (s).

HRMS (ESI): Calculated for $C_{17}H_{15}O_4F_5Na$ $[M+Na]^+$ 401.0783; Found 401.0783.

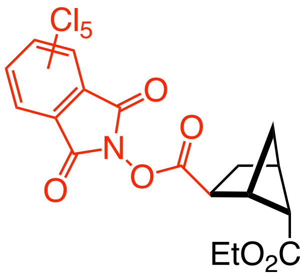

46d

5-ethyl-2-(4,5,6,7-tetrachloro-1,3-dioxoisoindolin-2-yl)bicyclo[2.1.1]hexane-2,5-dicarboxylate (46d): Starting material was synthesized as the general procedure F using N-Hydroxytetrachlorophthalimide as nucleophile and isolated as an off-white solid (250 mg, 26% yield, >20:1 dr).

R_f: 0.5 in 8% EA/Hex.

^{1}H NMR (500 MHz, $CDCl_3$) δ 4.13 (m, 2H), 3.48 (ddd, J = 9.1, 4.0, 2.0 Hz, 1H), 3.19 – 3.13 (m, 1H), 2.86 – 2.80 (m, 1H), 2.73 (t, J = 2.5 Hz, 1H), 2.24 (dd, J = 12.0, 9.2 Hz, 1H), 2.11 (ddd, J = 11.9, 3.9, 1.9 Hz, 1H), 1.63 – 1.58 (m, 1H), 1.27 (td, J = 7.2, 1.9 Hz, 3H), 1.23 (d, J = 8.0 Hz, 1H).

^{13}C NMR (126 MHz, $CDCl_3$) δ 171.3, 170.5, 157.8, 141.2, 130.6, 124.9, 60.5, 50.5, 45.3, 41.5, 38.6, 34.2, 29.4, 14.4.

6.2. Redox active cross coupling:

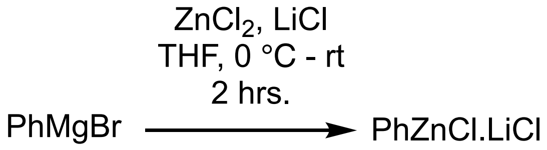

A flame-dried 2-dram vial was taken inside an argon-filled Glove-box where Zinc chloride (237 mg, 1 equiv., 1.74 mmol) and Lithium chloride (92.1 mg, 1.25 equiv., 2.17 mmol)were added and the vial was capped with a septum. Next, THF was added and the reaction mixture was cooled to 0 C. Followed by phenylmagnesium bromide (315 mg, 2.29 mL, 0.76 molar, 1 equiv., 1.74 mmol) was added dropwise and the reaction was moved to room temperature. After stirring for 2 hrs, a white precipitate was formed which was allowed to settle down. The Phenyl zinc chloride. lithium chloride was assumed to be quantitative (0.1 M) and was directly used in the next step.

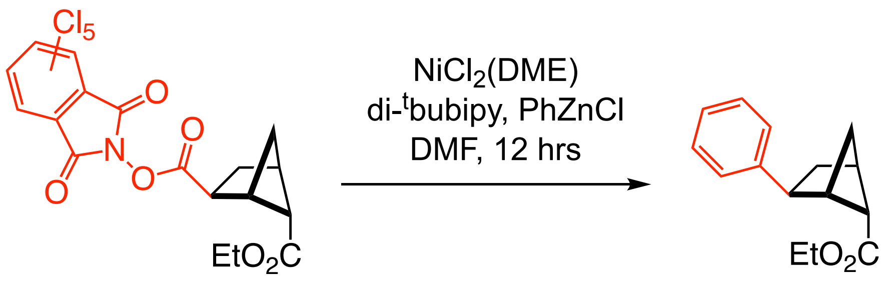

46d **47**

ethyl 2-phenylbicyclo[2.1.1]hexane-5-carboxylate (47): A flame dried 2-dram vial with a stir bar was charged with $NiCl_2$·DME (4.2 mg, 0.2 equiv., 19 μmol), 4,4'-di-tert-butyl-2,2'-bipyridyl (10 mg, 0.4 equiv., 38 μmol) and ethyl-2-(4,5,6,7-tetrachloro-1,3-dioxoisoindolin-2-yl)bicyclo[2.1.1]hexane-5-carboxylate **46d** (42 mg, 1 equiv., 96 μmol) . The vial was sealed with a screw cap, evacuated, and refilled with N_2 (x3). DMF (0.8 mL) was added via syringe, and the mixture stirred for 2 minutes at room temperature. Then, PhZnCl·LiCl (2.9 mL, 0.1 molar, 3 equiv., 0.29 mmol) was added in one portion, and the mixture was stirred for 12 hrs at r.t. The mixture was diluted with ethyl acetate and quenched with 1M HCl (aq). The organic layer was washed with water and $NaHCO_3$, dried over anh. Na_2SO_4, and concentrated under *vacuo*. The crude product was dissolved in $CDCl_3$ and an exact amount of standard mesitylene (0.028 ml, 0.2 mmol) was added to check the NMR yield. The crude material was purified via flash column chromatography to afford **47** (4 mg, 19 μmol, 20% yield, >20:1 dr) as a clear, colourless liquid.

NMR yield= 42%, Isolated yield= 20%

R_f: 0.7 in 10% Ethyl ether/Hex.

^{1}H NMR (500 MHz, $CDCl_3$) δ 7.26 – 7.07 (m, 5H), 4.07 (q, J = 7.2 Hz, 2H), 3.35 (dd, J = 9.6, 3.9 Hz, 1H), 3.00 (dd, J = 6.6, 3.0 Hz, 1H), 2.73 (d, J = 6.1 Hz, 1H), 2.60 (t, J = 2.6 Hz, 1H), 2.30 (t, J = 10.2 Hz, 1H), 1.74 – 1.68 (m, 1H), 1.19 (td, J = 7.1, 1.9 Hz, 3H), 1.02 (d, J = 7.0 Hz, 1H), 0.84 – 0.79 (m, 1H).

^{13}C NMR (126 MHz, $CDCl_3$) δ 171.6, 144.6, 128.3, 127.7, 125.8, 60.1, 50.6, 46.3, 41.7, 40.3, 33.2, 31.9, 14.5.

IR (neat) 2980 (m), 2929 (m), 1731 (s), 1601 (w), 1494 (w), 1220 (m), 699 (m).

HRMS (ESI): Calculated for $C_{15}H_{19}O_2$ $[M+H]^+$ 231.1380; Found 231.1380.

7. Characterization data

NMR of compound SI-3

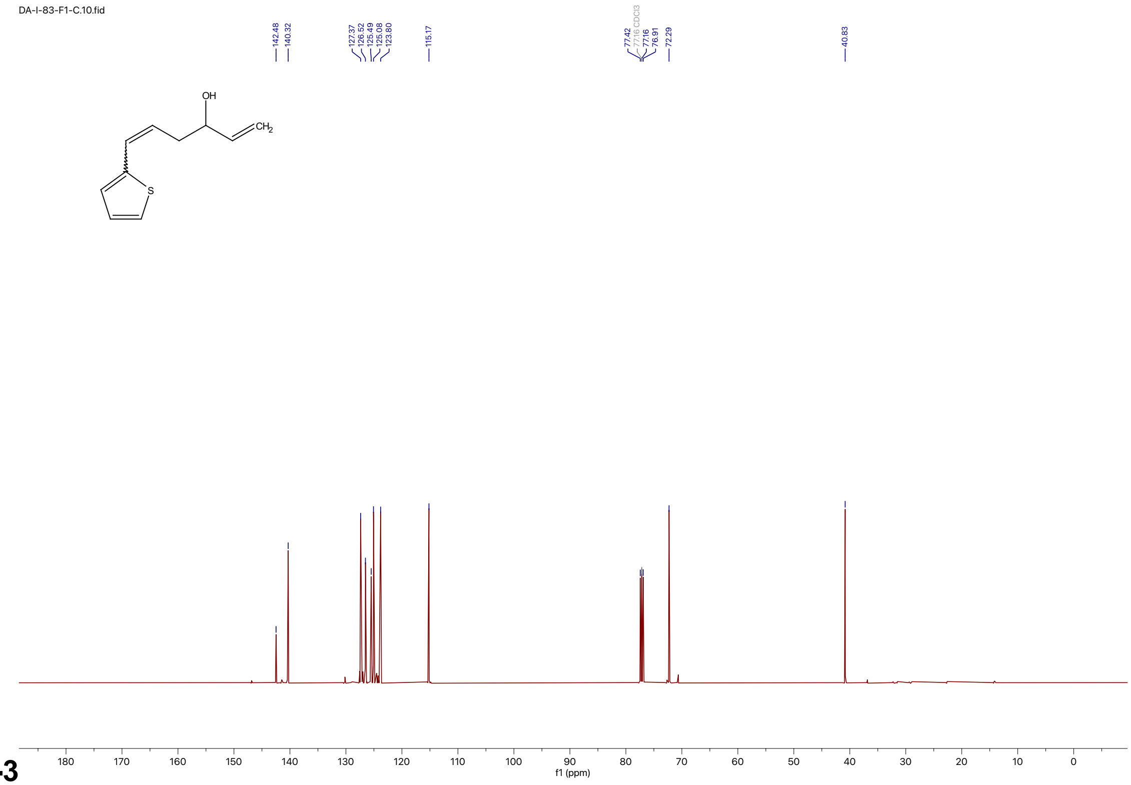

SI-3

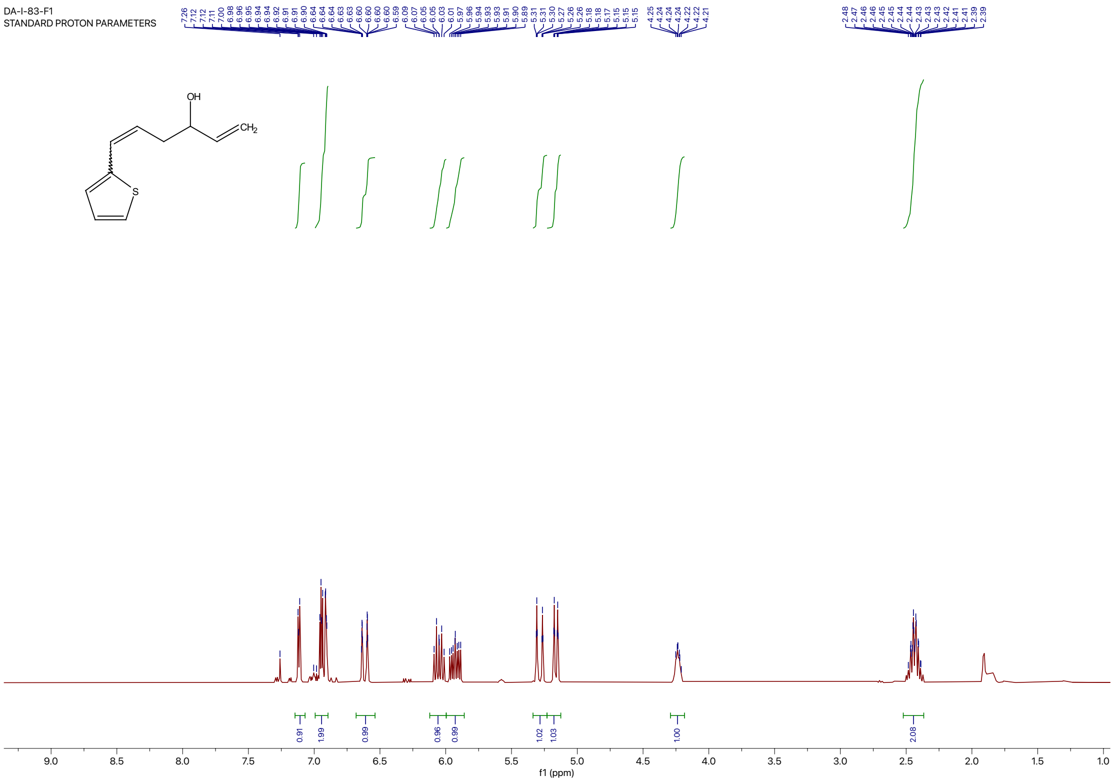

SI-4

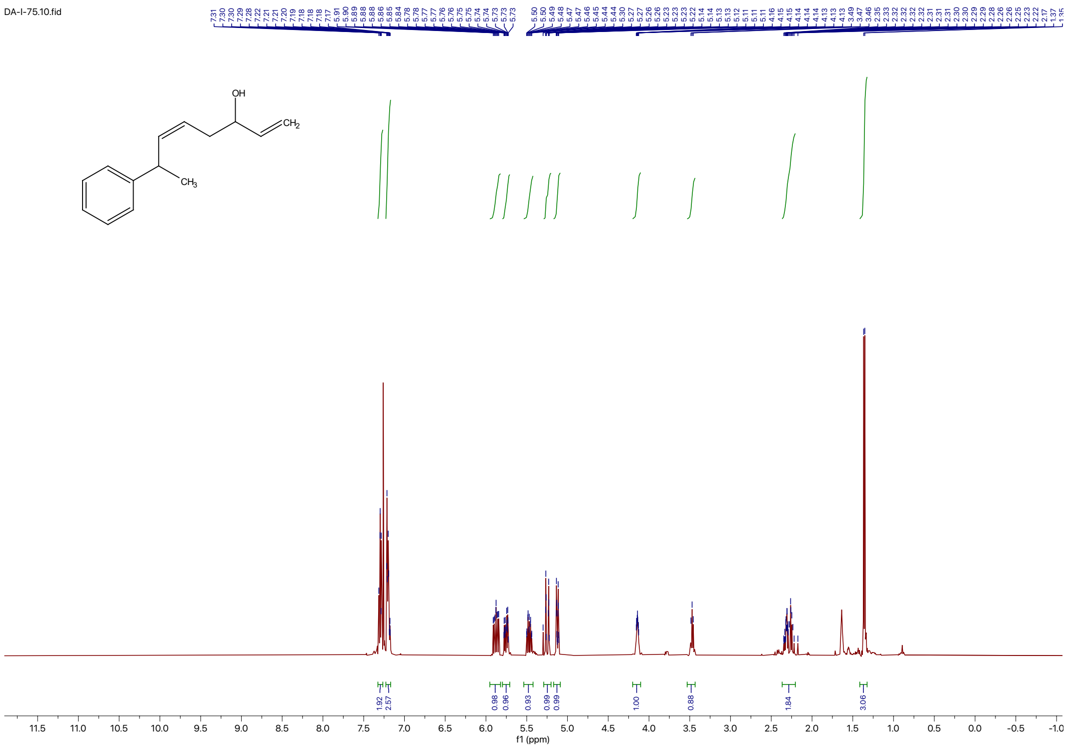
DA-I-75.10.fid
OH
CH2
CH3
1.92
2.57
0.98
0.96
0.93
0.99
0.99
1.00
0.88
1.84
3.06
f1 (ppm)

SI-4

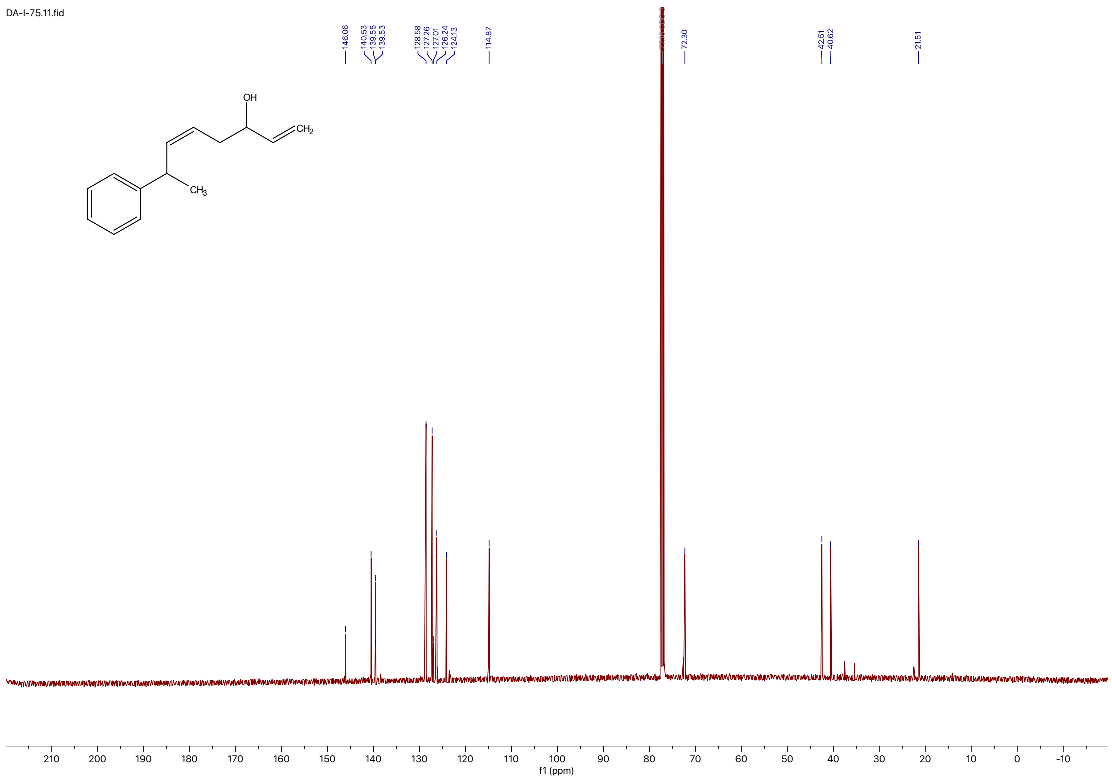
DA-I-75.11.fid
146.06
140.53
139.55
139.53
128.58
127.26
127.01
126.24
124.13
114.87
72.30
42.51
40.62
21.51
OH
CH2
CH3
f1 (ppm)

SI-5

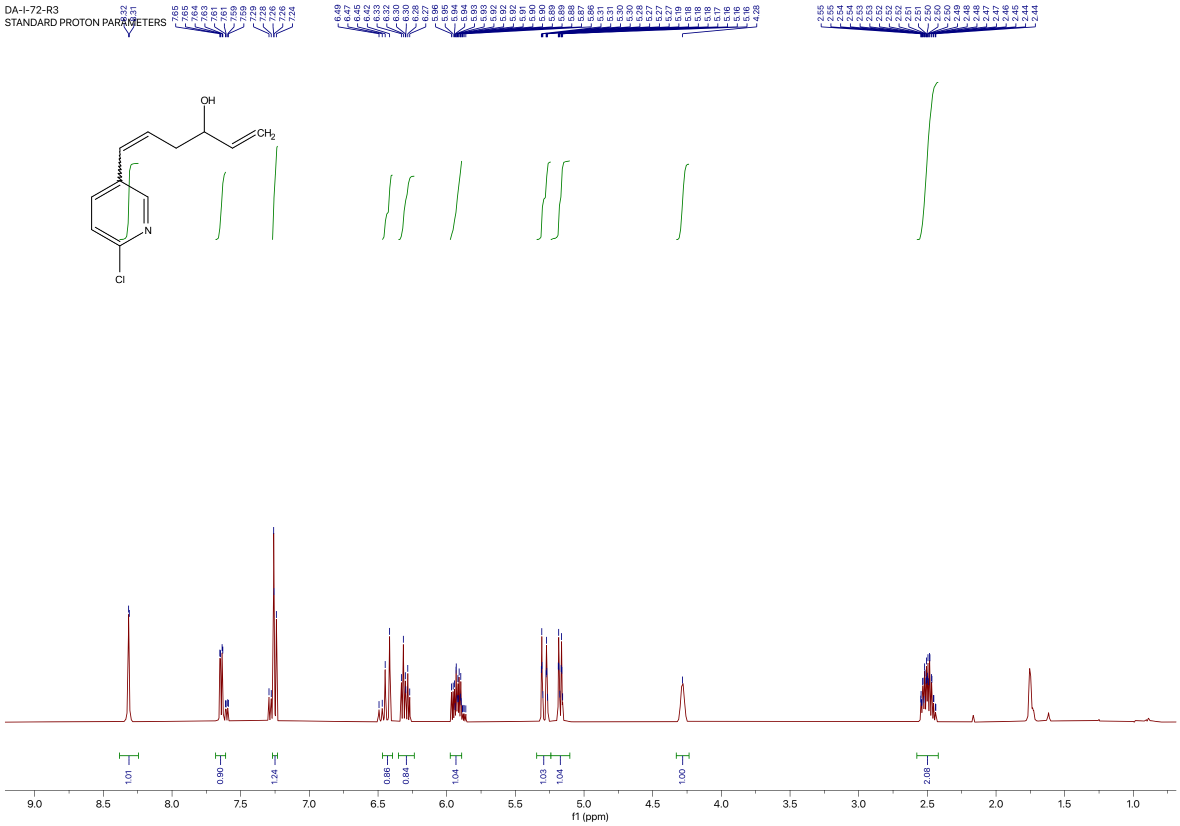
DA-I-72-R3
STANDARD PROTON PARAMETERS
OH
CH2
N
Cl
1.01
0.90
1.24
0.86
0.84
1.04
1.03
1.04
1.00
2.08
f1 (ppm)

SI-5

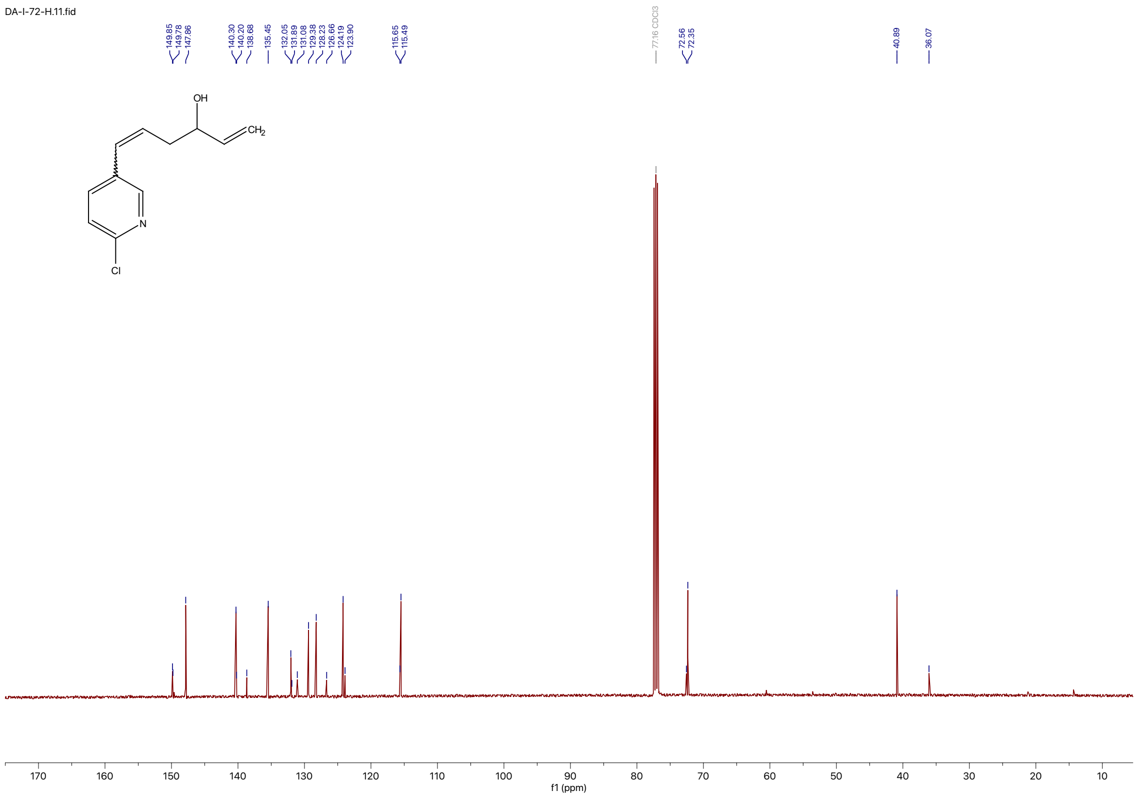
DA-I-72-H.11.fid
149.85
149.78
147.86
140.30
140.20
138.68
135.45
132.05
131.89
131.08
129.38
128.23
126.66
124.19
123.90
115.65
115.49
77.16 CDCl3
72.56
72.35
40.89
36.07
OH
CH2
N
Cl
f1 (ppm)

SI-6

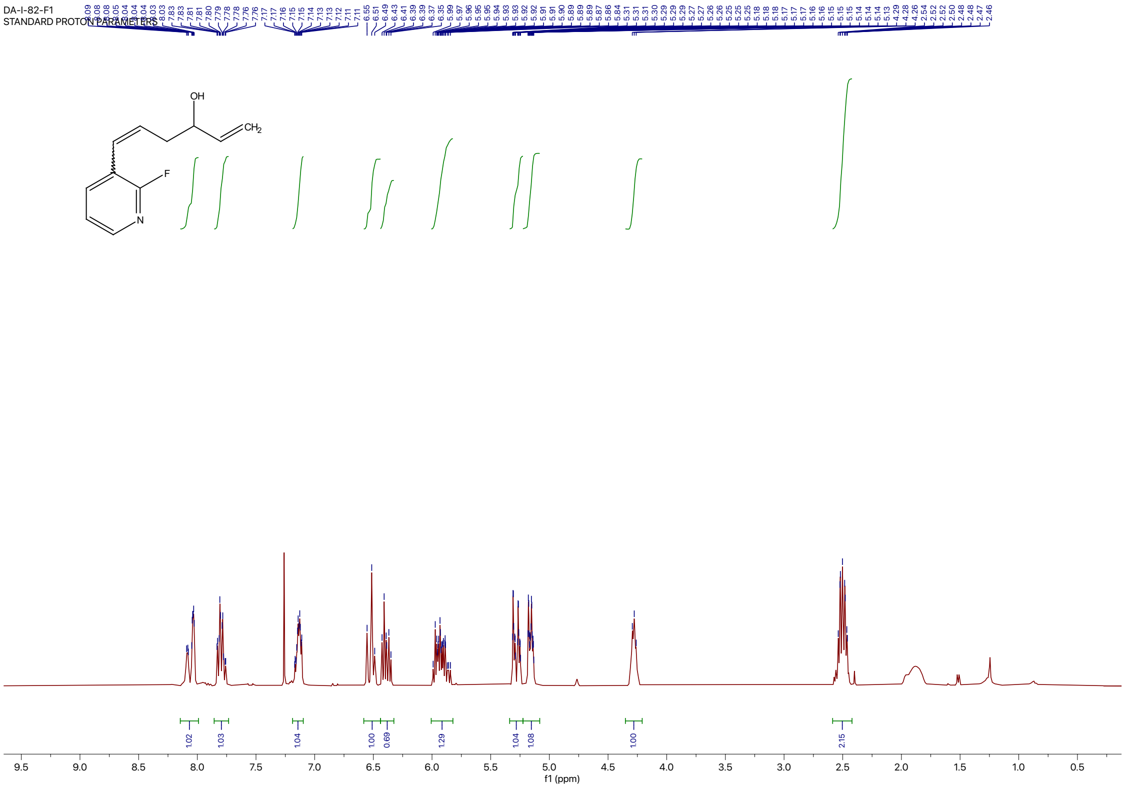
DA-I-82-F1
STANDARD PROTON PARAMETERS
OH
CH2
F
N
1.02
1.03
1.04
1.00
0.69
1.29
1.04
1.08
1.00
2.15
9.5
9.0
8.5
8.0
7.5
7.0
6.5
6.0
5.5
5.0
4.5
4.0
3.5
3.0
2.5
2.0
1.5
1.0
0.5
f1 (ppm)

SI-6

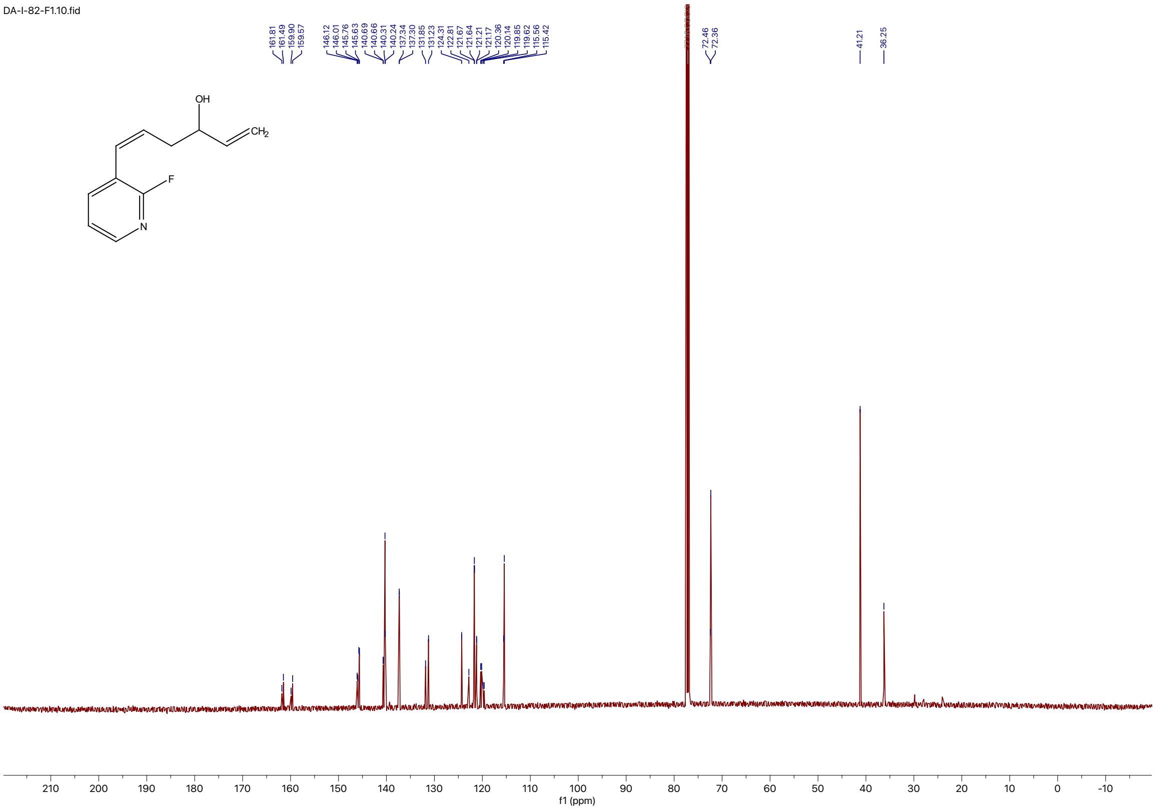
DA-I-82-F1.10.fid
161.81
161.49
159.90
159.57
146.12
146.01
145.76
145.63
140.69
140.66
140.31
140.24
137.34
137.30
131.55
131.23
124.31
122.81
121.67
121.64
121.21
121.17
120.36
120.14
119.85
119.62
115.56
115.42
72.46
72.36
41.21
36.25
OH
CH2
F
N
210
200
190
180
170
160
150
140
130
120
110
100
90
80
70
60
50
40
30
20
10
0
-10
f1 (ppm)

SI-7

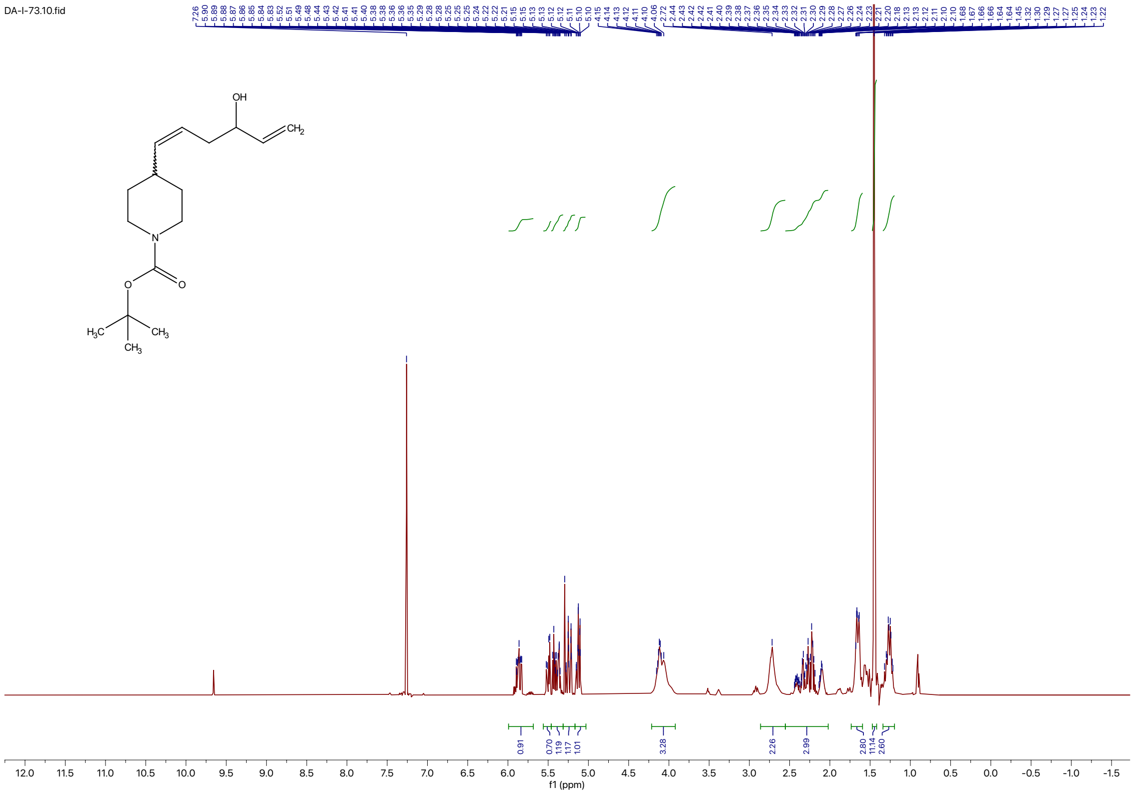

SI-7

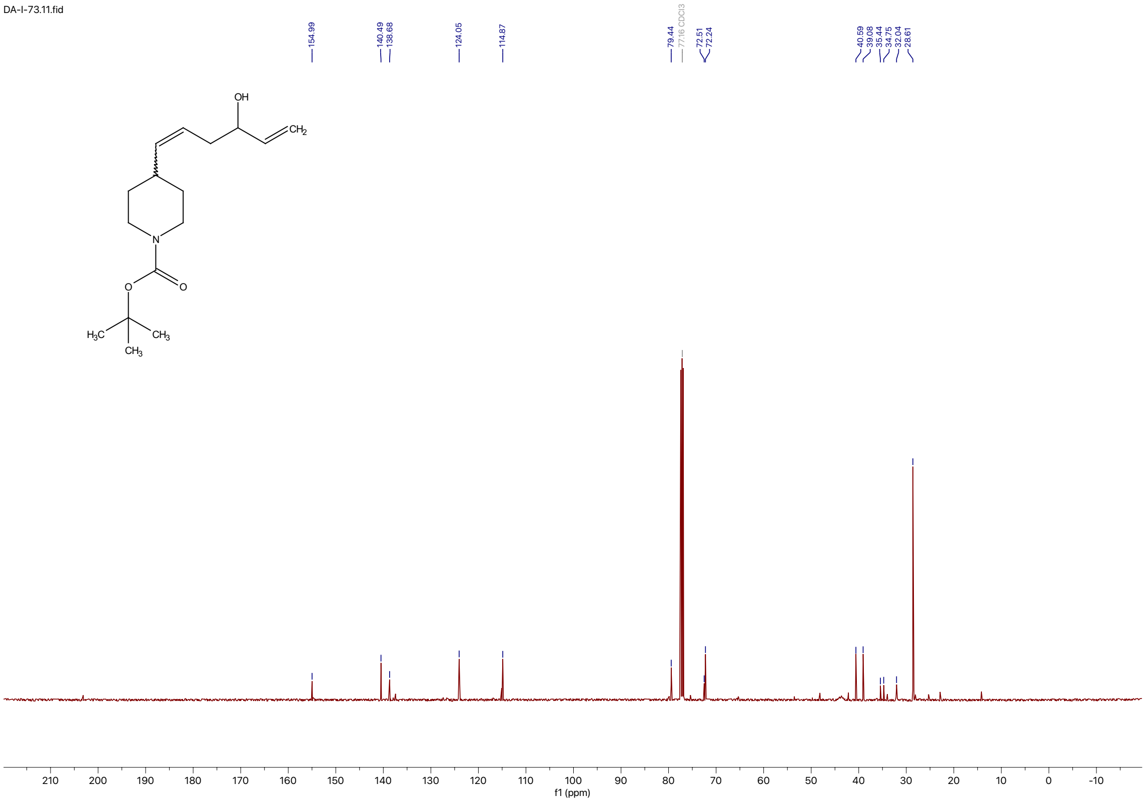

9

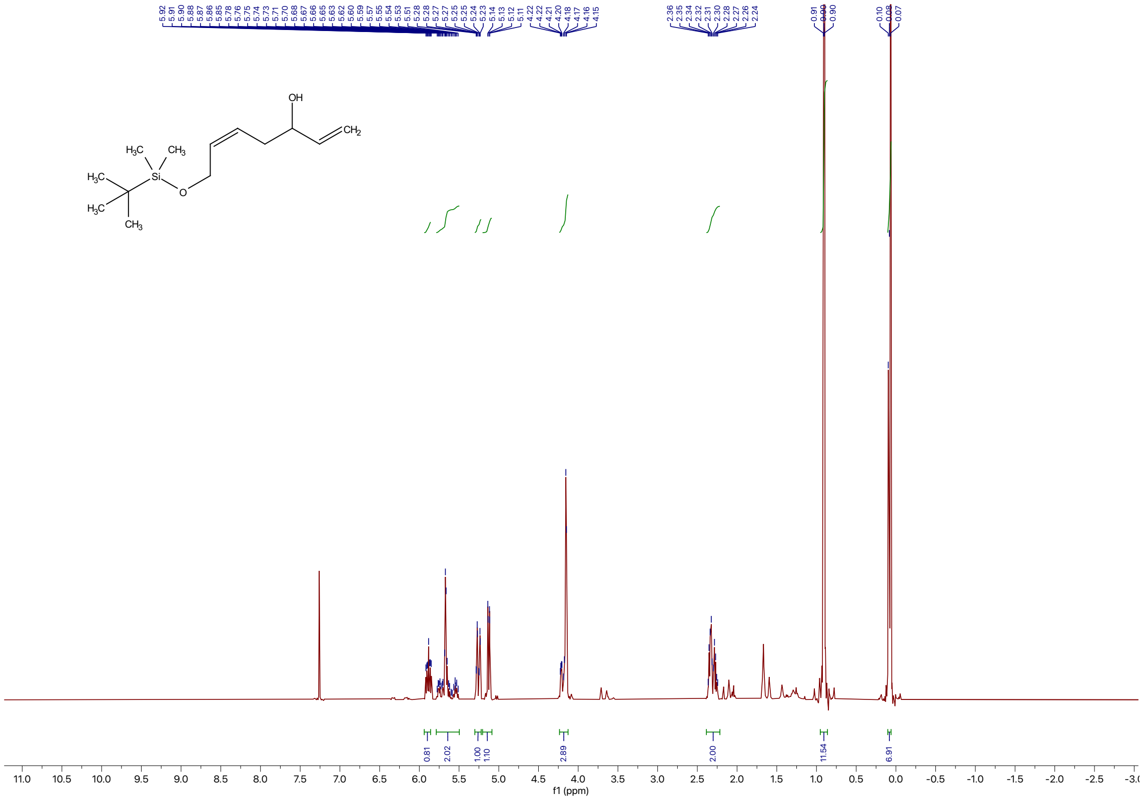

9

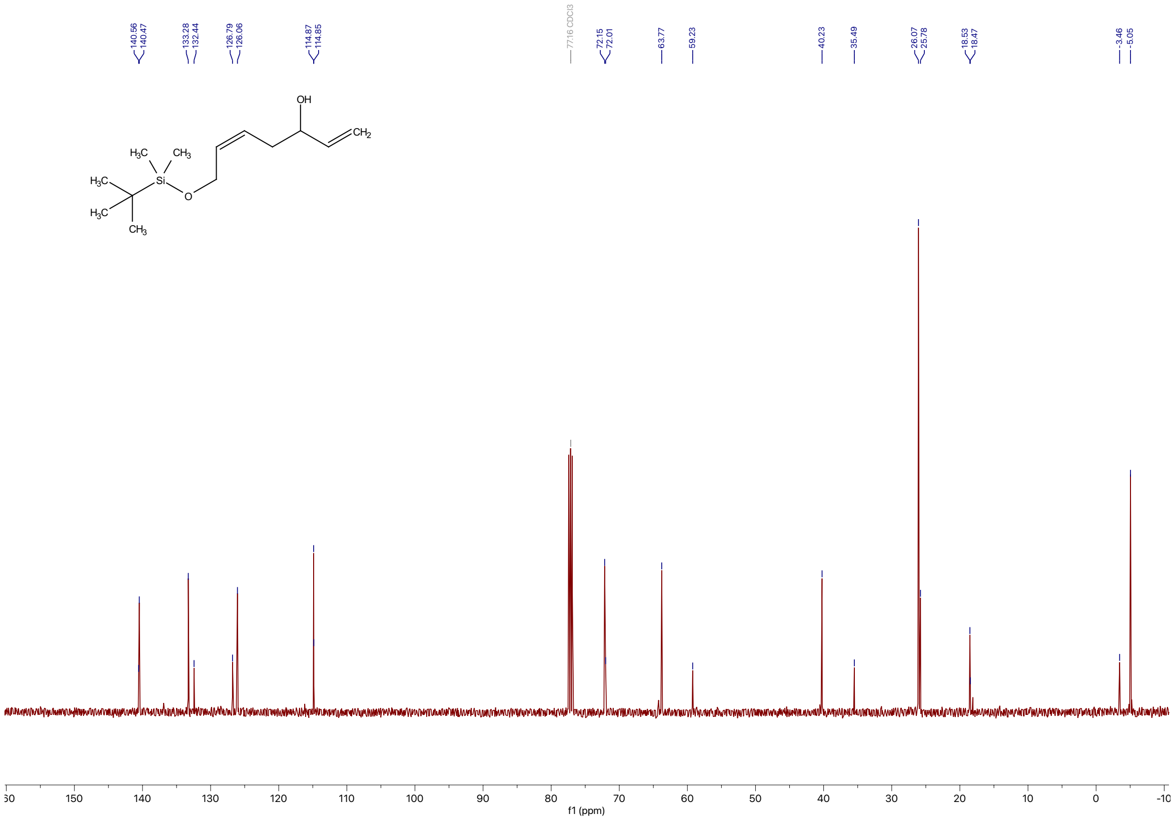

10

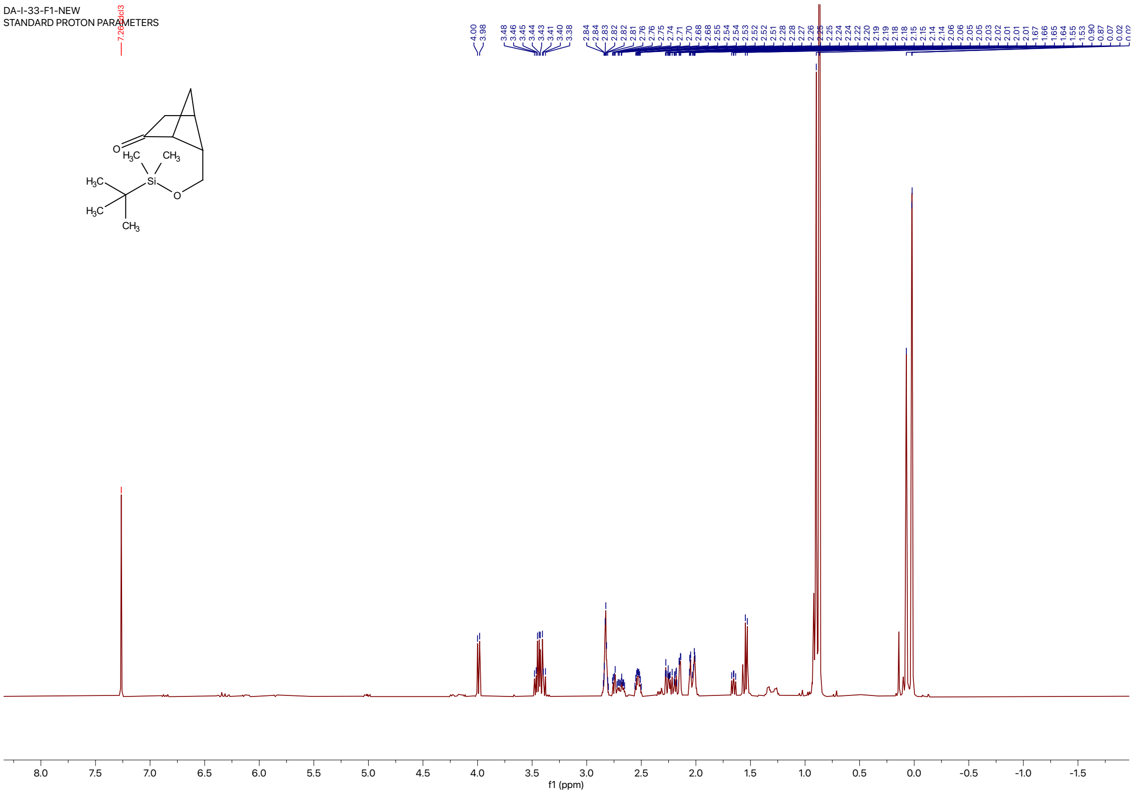
DA-I-33-F1-NEW
STANDARD PROTON PARAMETERS
7.26 cdcl3
4.00
3.98
3.48
3.46
3.45
3.44
3.43
3.41
3.40
3.38
2.84
2.84
2.83
2.82
2.82
2.81
2.76
2.76
2.75
2.74
2.71
2.70
2.68
2.68
2.55
2.54
2.54
2.53
2.52
2.52
2.51
2.28
2.28
2.27
2.26
2.25
2.25
2.24
2.24
2.22
2.20
2.19
2.19
2.18
2.18
2.15
2.15
2.14
2.14
2.06
2.06
2.05
2.05
2.03
2.02
2.01
2.01
1.67
1.66
1.65
1.64
1.55
1.53
0.90
0.87
0.07
0.02
O
H3C
CH3
Si
O
H3C
H3C
CH3
8.0
7.5
7.0
6.5
6.0
5.5
5.0
4.5
4.0
3.5
3.0
2.5
2.0
1.5
1.0
0.5
0.0
-0.5
-1.0
-1.5
f1 (ppm)

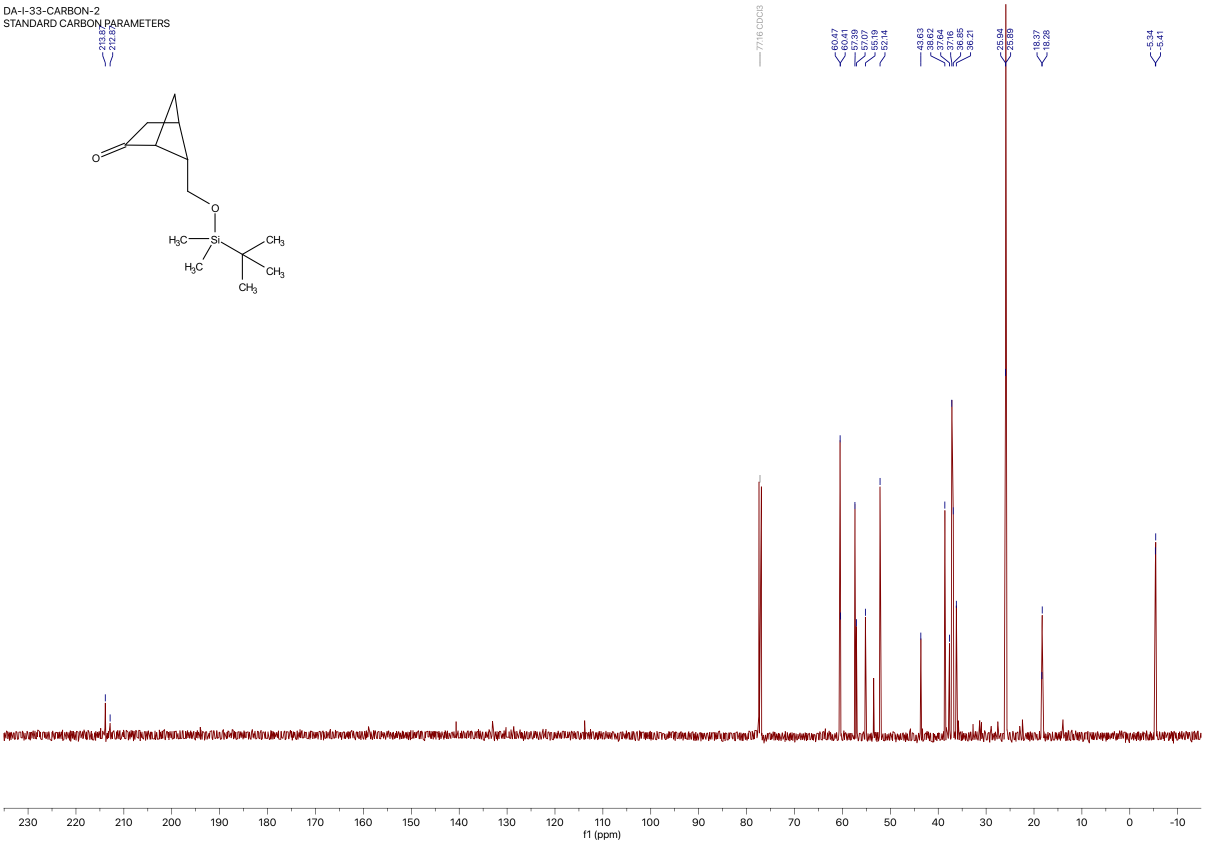
DA-I-33-CARBON-2
STANDARD CARBON PARAMETERS
213.87
212.87
77.16 CDCl3
60.47
60.41
57.39
57.07
55.19
52.14
43.63
38.62
37.64
37.16
36.85
36.21
25.94
25.89
18.37
18.28
-5.34
-5.41
O
O
H3C
Si
CH3
H3C
CH3
CH3
230
220
210
200
190
180
170
160
150
140
130
120
110
100
90
80
70
60
50
40
30
20
10
0
-10
f1 (ppm)

11

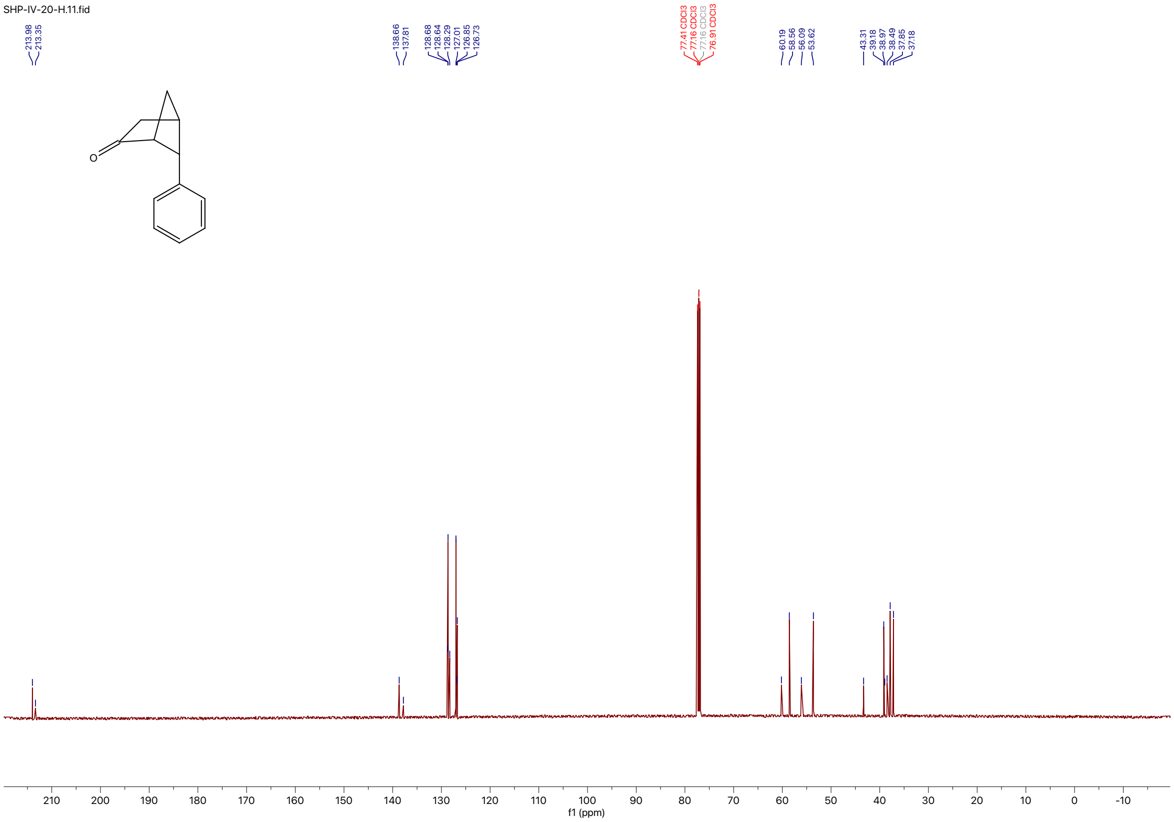
SHP-IV-20-H.11.fid
213.98
213.35
138.66
137.81
128.68
128.64
128.29
127.01
126.85
126.73
77.41 CDCl3
77.16 CDCl3
77.16 CDCl3
76.91 CDCl3
60.19
58.56
56.09
53.62
43.31
39.18
38.97
38.49
37.85
37.18
O
f1 (ppm)

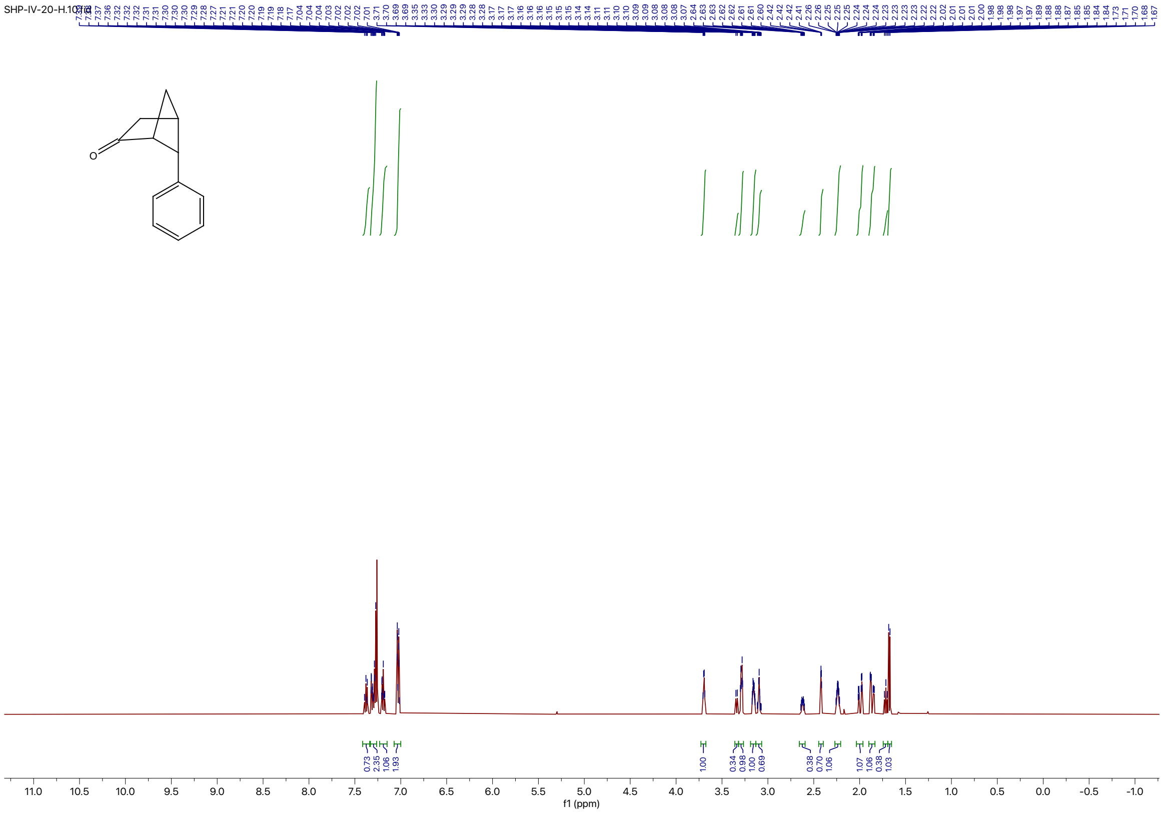
O
f1 (ppm)

12

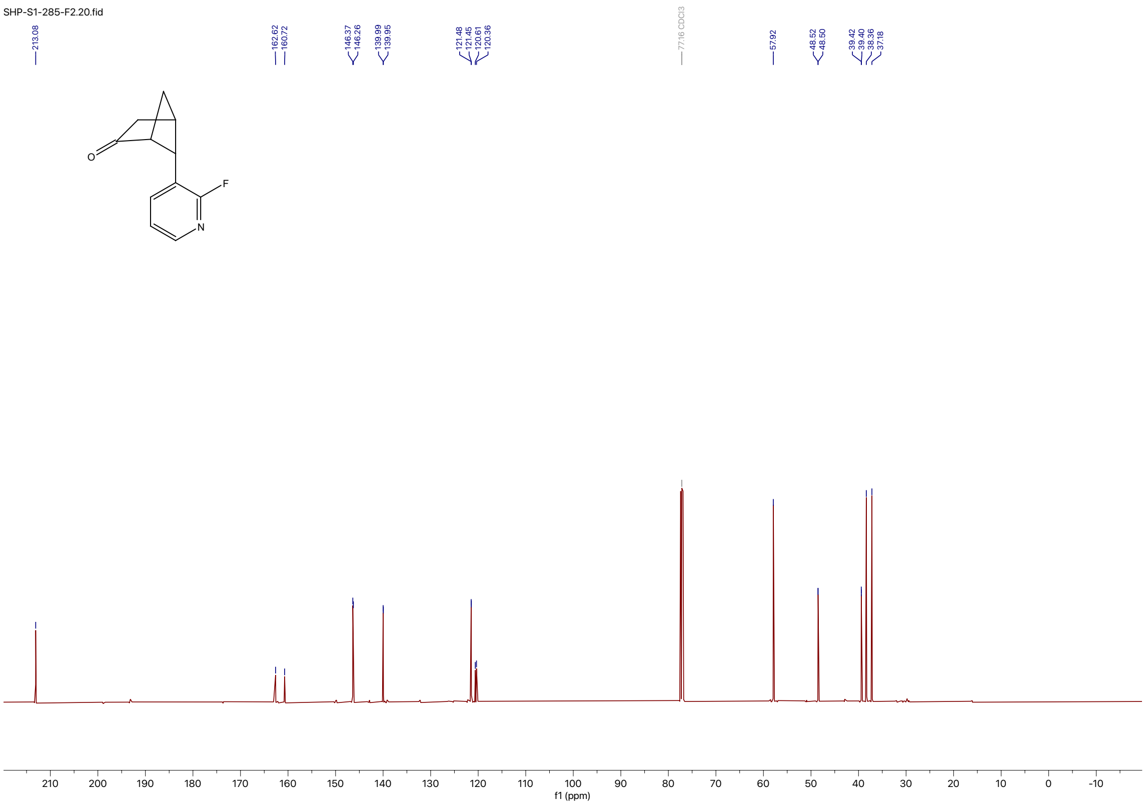
SHP-S1-285-F2.20.fid
213.08
162.62
160.72
146.37
146.26
139.99
139.95
121.48
121.45
120.61
120.36
77.16 CDCl3
57.92
48.52
48.50
39.42
39.40
38.36
37.18
O
F
N
f1 (ppm)

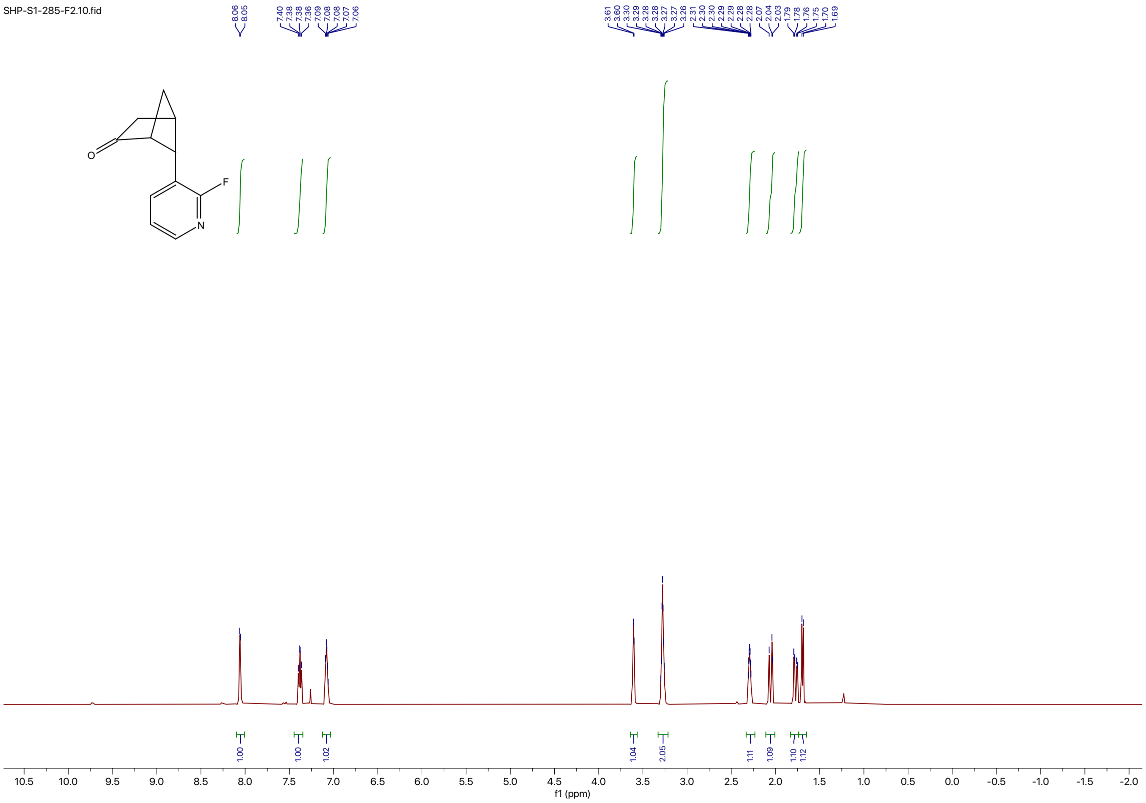
SHP-S1-285-F2.10.fid
8.06
8.05
7.40
7.38
7.36
7.09
7.08
7.08
7.07
7.06
3.61
3.60
3.30
3.29
3.28
3.28
3.27
3.27
3.26
2.31
2.30
2.30
2.29
2.29
2.28
2.28
2.07
2.04
2.03
1.79
1.78
1.76
1.75
1.70
1.69
O
F
N
1.00
1.00
1.02
1.04
2.05
1.11
1.09
1.10
1.12
f1 (ppm)

13

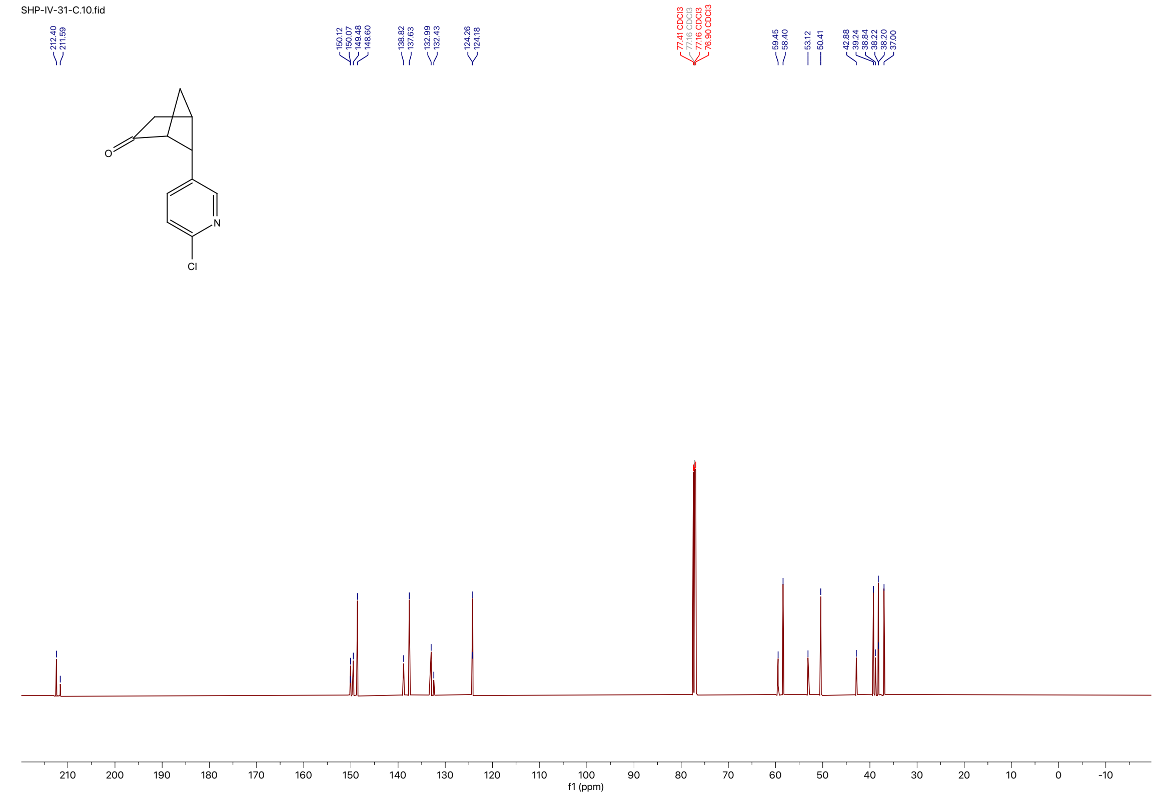
SHP-IV-31-C.10.fid
212.40
211.59
150.12
150.07
149.48
148.60
138.82
137.63
132.99
132.43
124.26
124.18
77.41 CDCl3
77.16 CDCl3
77.16 CDCl3
76.90 CDCl3
59.45
58.40
53.12
50.41
42.88
39.24
38.84
38.22
38.20
37.00
O
N
Cl
f1 (ppm)

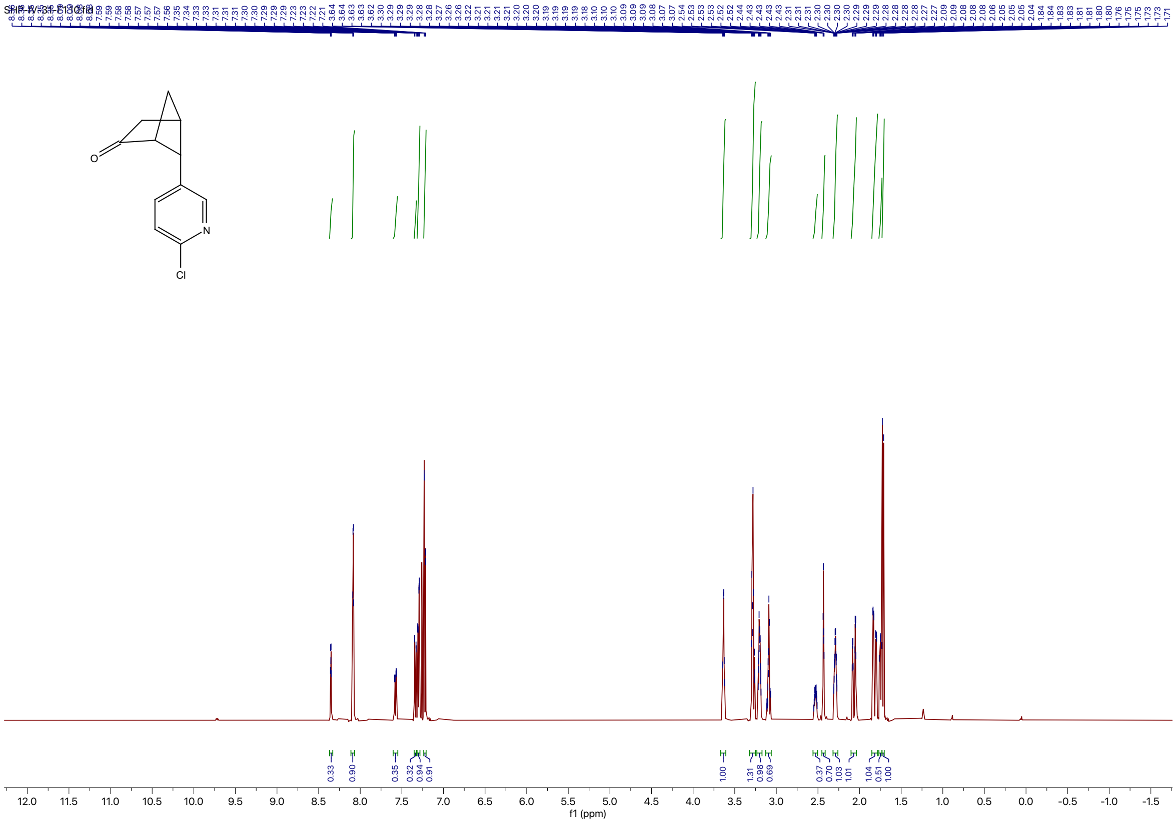
O
N
Cl
0.33
0.90
0.35
0.32
0.94
0.91
1.00
1.31
0.98
0.69
0.37
0.70
1.03
1.01
1.04
0.51
1.00
f1 (ppm)

14

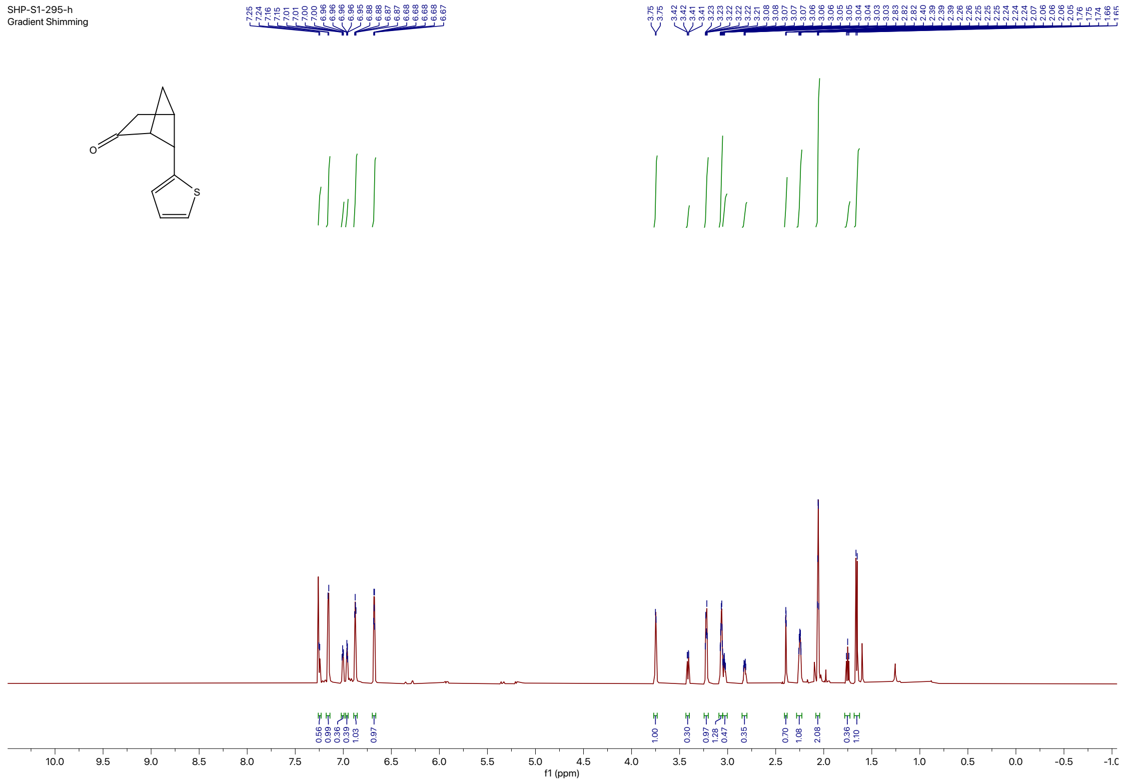
SHP-S1-295-h
Gradient Shimming
O
S
f1 (ppm)

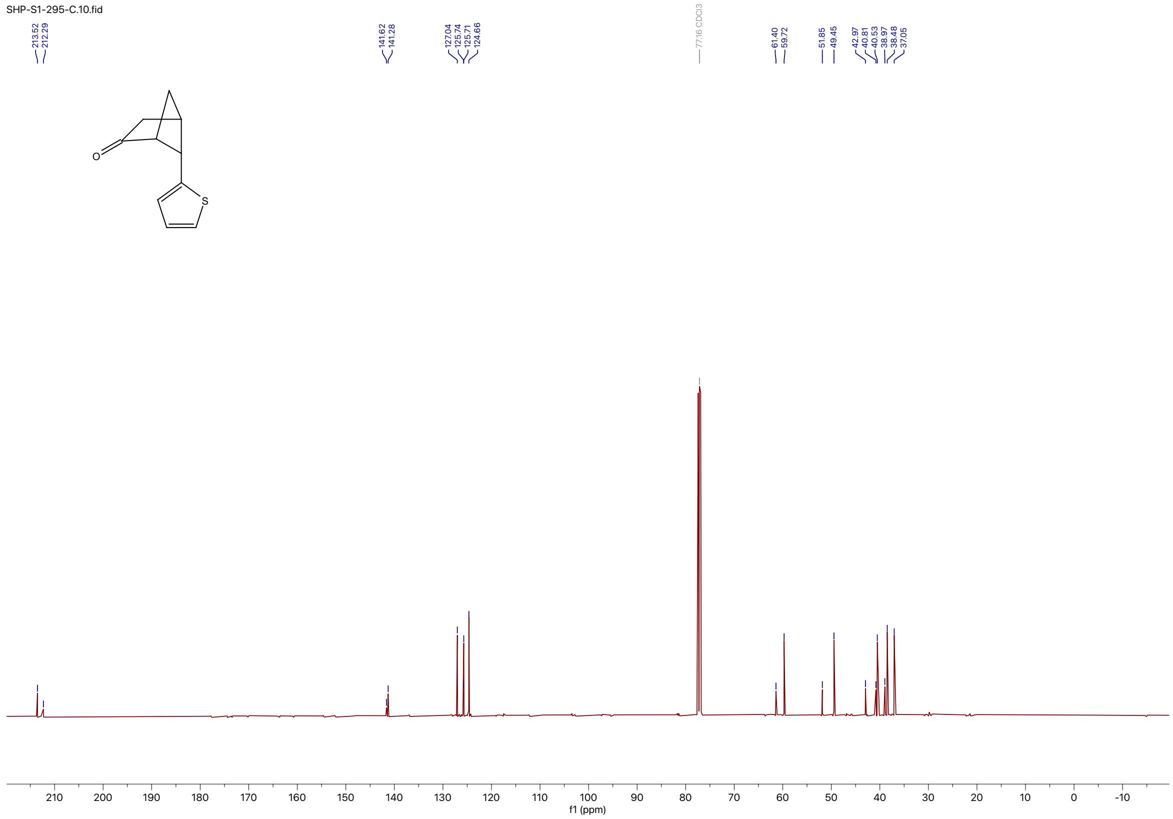
SHP-S1-295-C.10.fid
213.52
212.29
141.62
141.28
127.04
125.74
125.71
124.66
77.16 CDCl3
61.40
59.72
51.85
49.45
42.97
40.81
40.53
38.97
38.48
37.05
O
S
f1 (ppm)

15

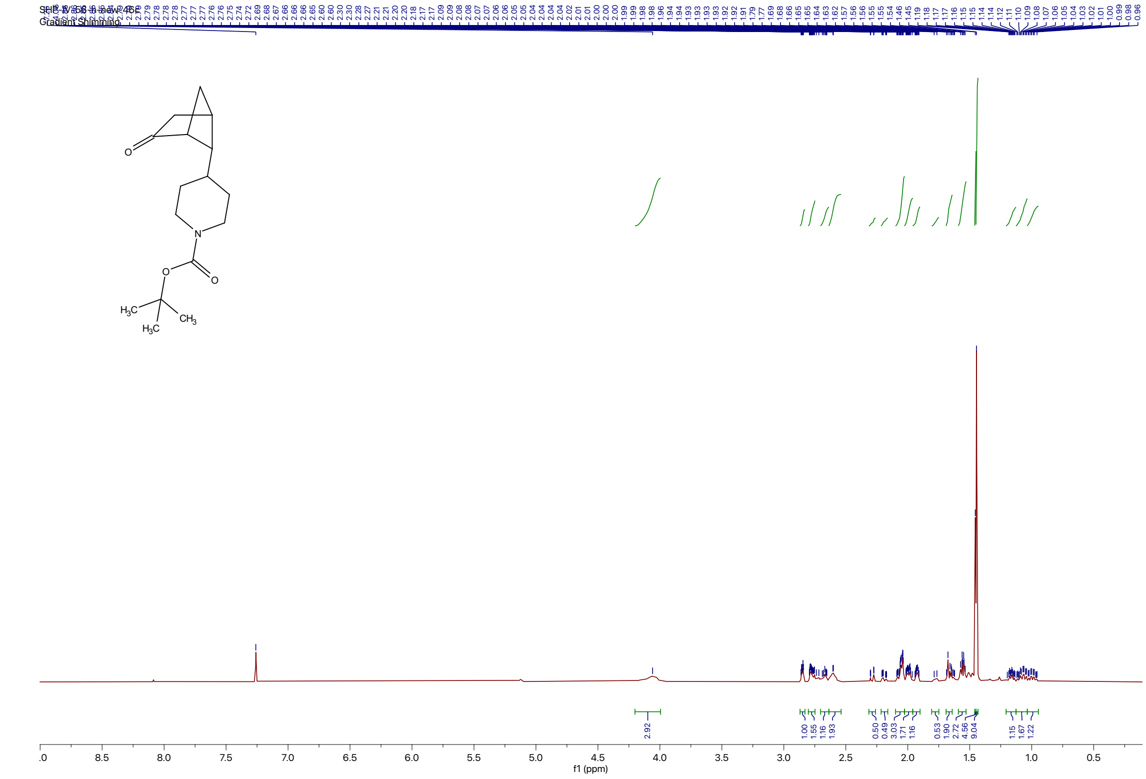

f1 (ppm)

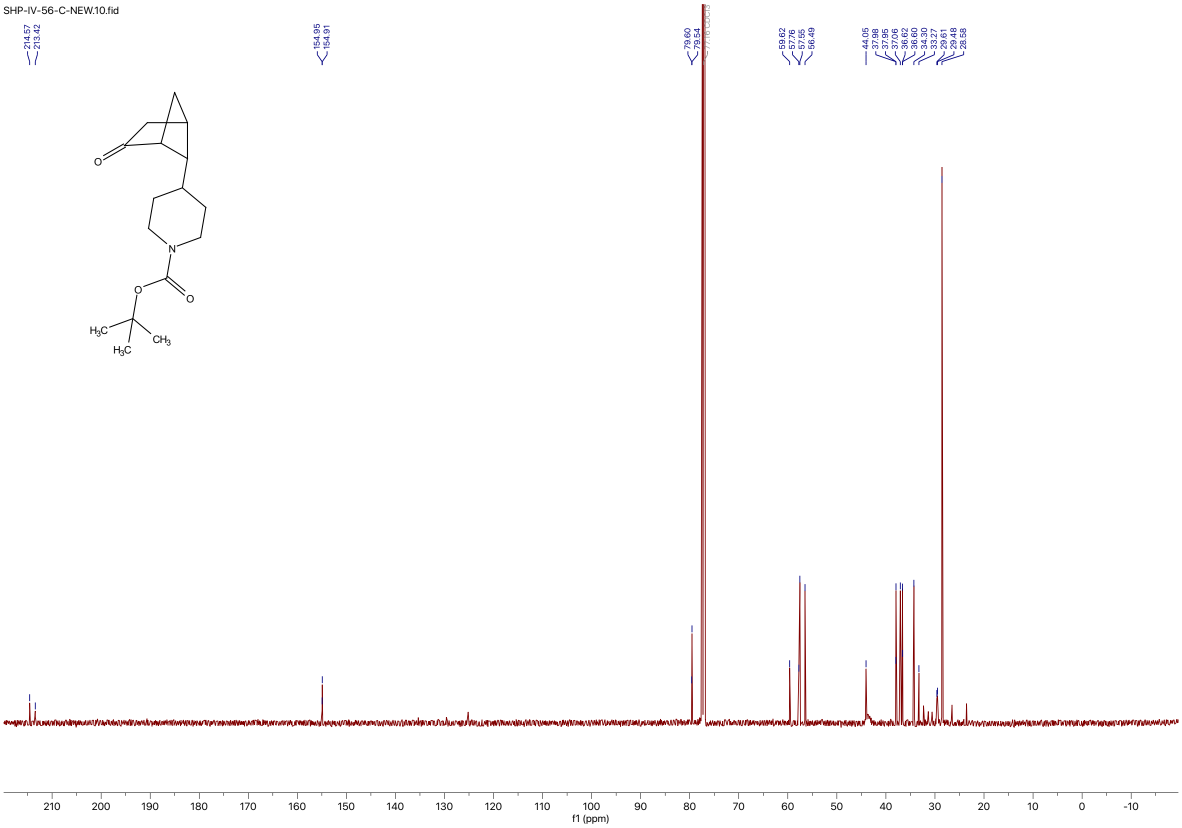

SHP-IV-56-C-NEW.10.fid
214.57
213.42
154.95
154.91
79.60
79.54
59.62
57.76
57.55
56.49
44.05
37.98
37.95
37.06
36.62
36.60
34.30
33.27
29.61
29.48
28.58
f1 (ppm)

16

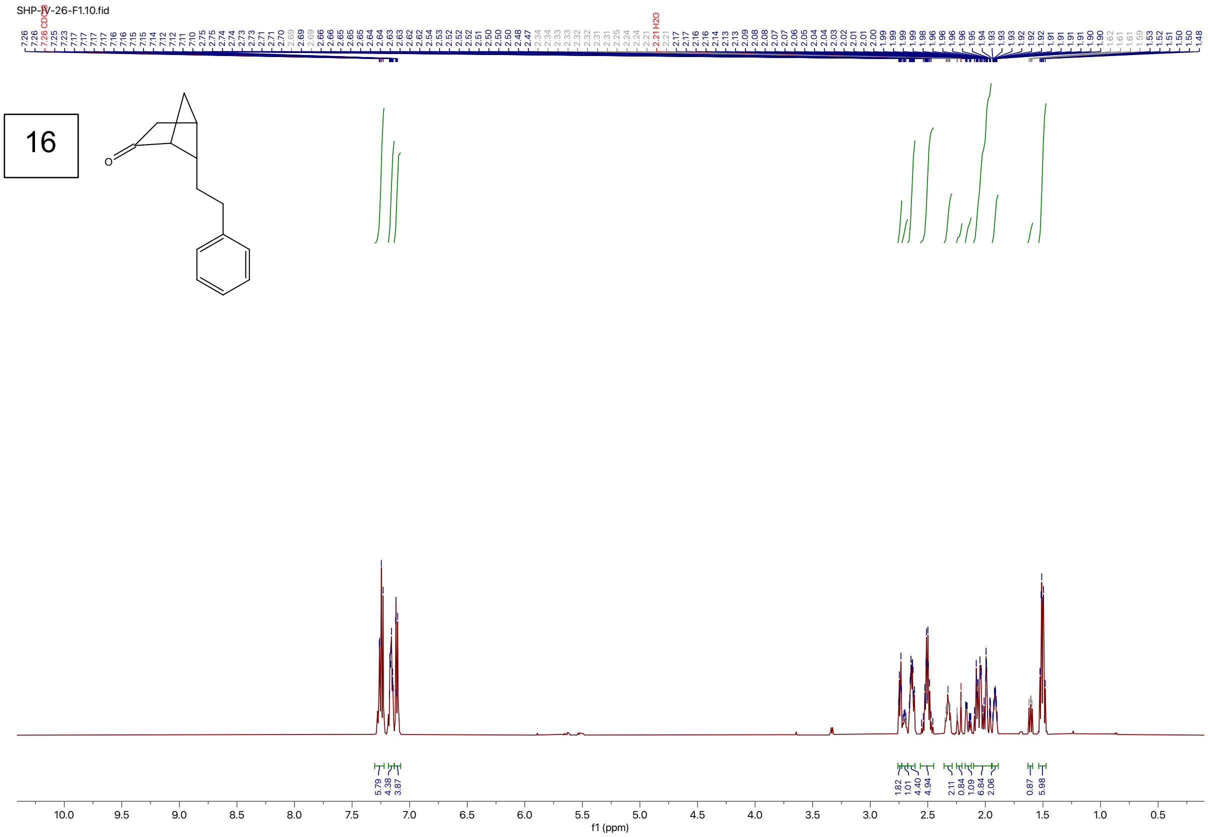
SHP-IV-26-F1.10.fid
7.26 CDCl3
2.21 H2O
16
O
f1 (ppm)

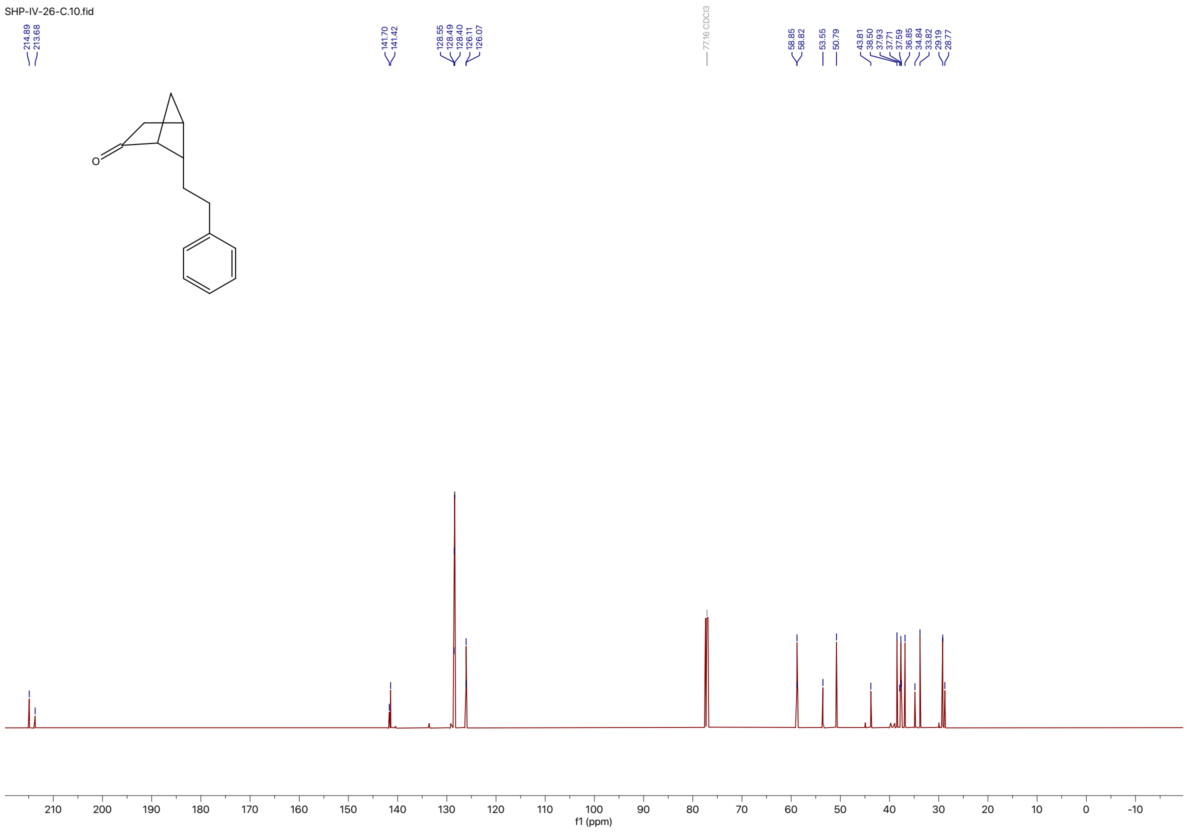
SHP-IV-26-C.10.fid
214.89
213.68
141.70
141.42
128.55
128.49
128.40
126.11
126.07
77.16 CDCl3
58.85
58.82
53.55
50.79
43.81
38.50
37.93
37.71
37.59
36.85
34.84
33.82
29.19
28.77
O
f1 (ppm)

17

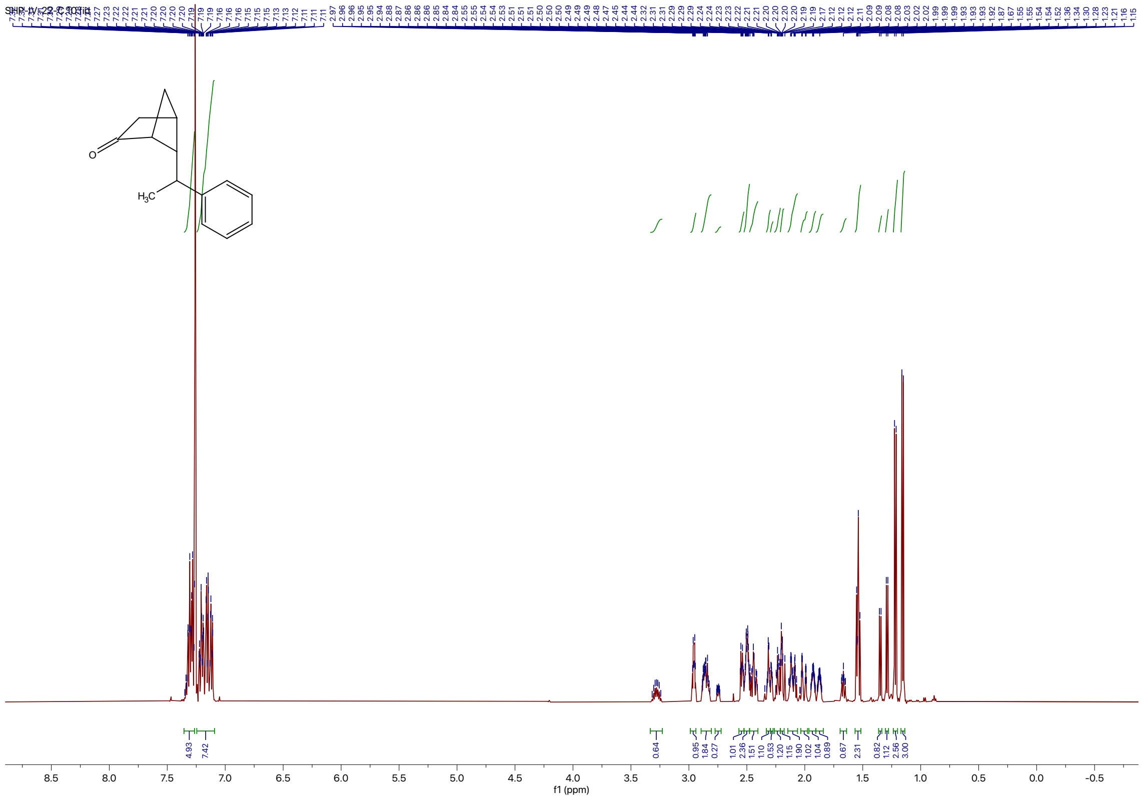

H3C
O
f1 (ppm)

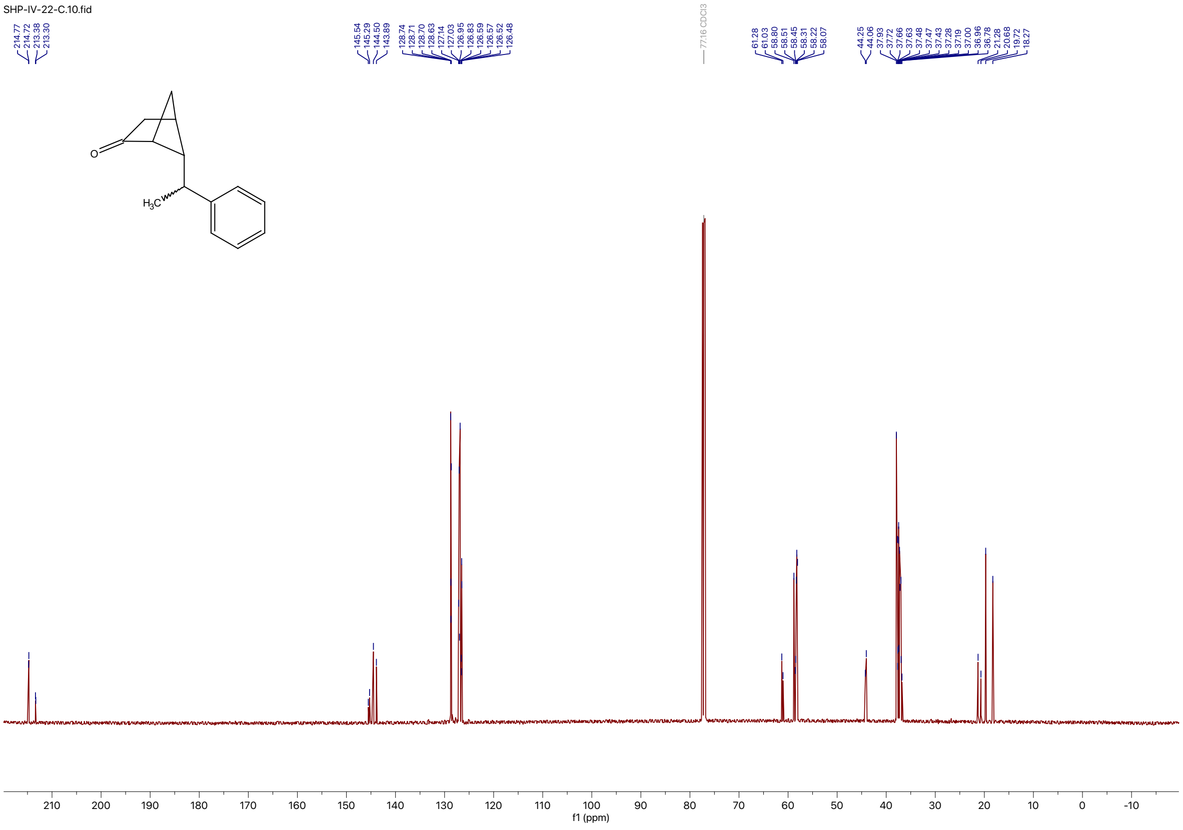

SHP-IV-22-C.10.fid
214.77 214.72 213.38 213.30
145.54 145.29 144.50 143.89
128.74 128.71 128.70 128.63 127.14 127.03 126.95 126.83 126.59 126.57 126.52 126.48
77.16 CDCl3
61.28 61.03 58.60 58.51 58.45 58.31 58.22 58.07
44.25 44.06 37.93 37.72 37.66 37.63 37.48 37.47 37.43 37.28 37.19 37.00 36.96 36.78 21.28 20.68 19.72 18.27
O
H3C
f1 (ppm)

SI-9

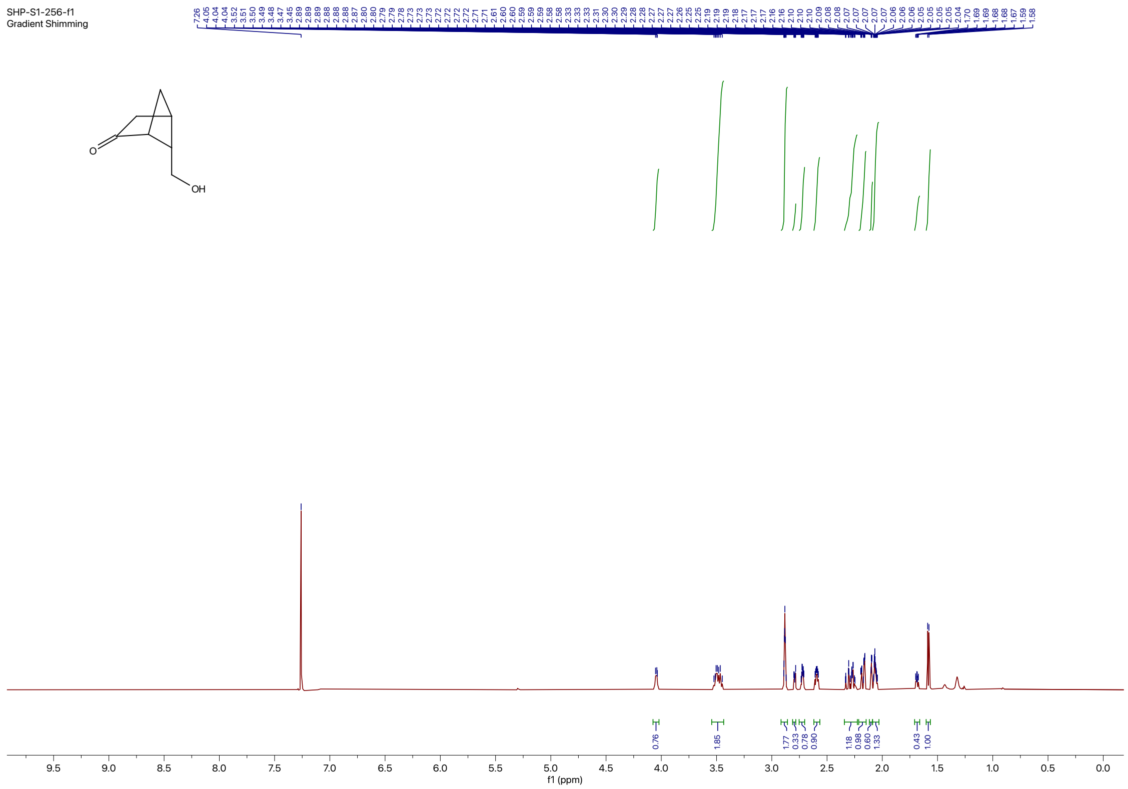

SHP-S1-256-f1
Gradient Shimming
O
OH
0.76
1.85
1.77
0.33
0.78
0.90
1.18
0.98
0.60
1.33
0.43
1.00
9.5 9.0 8.5 8.0 7.5 7.0 6.5 6.0 5.5 5.0 4.5 4.0 3.5 3.0 2.5 2.0 1.5 1.0 0.5 0.0
f1 (ppm)

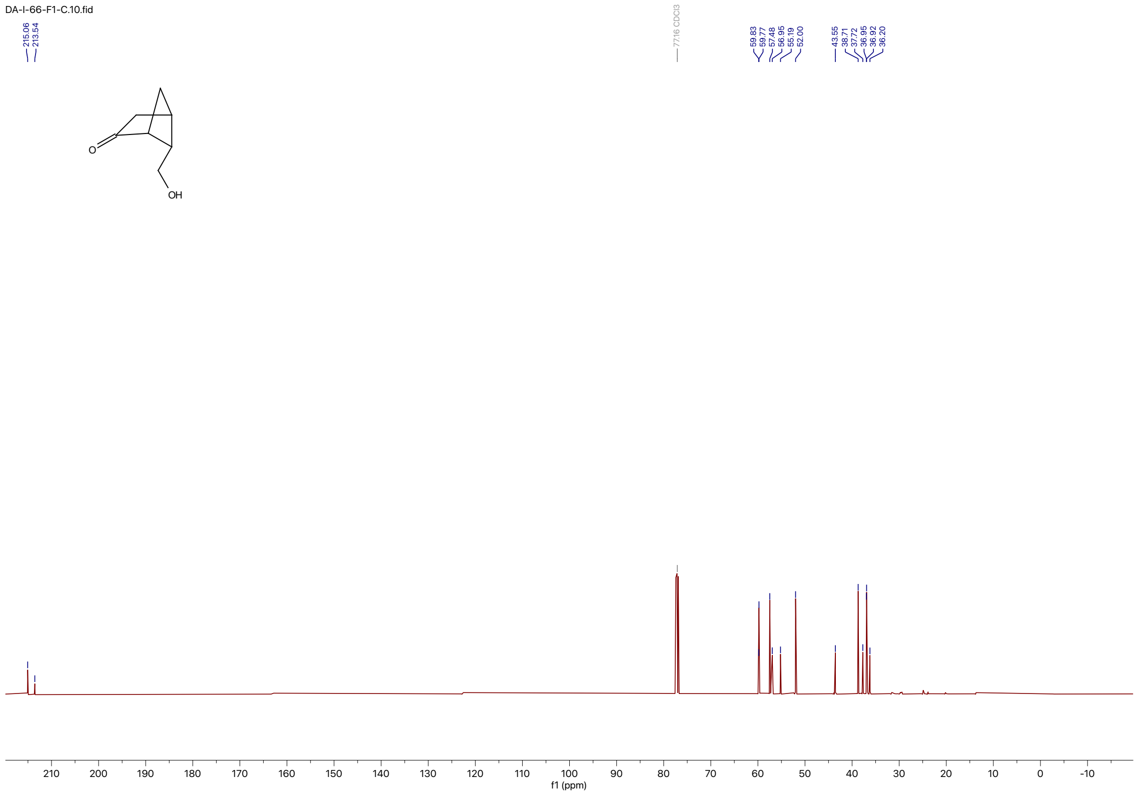

DA-I-66-F1-C.10.fid
215.06
213.54
77.16 CDCl3
59.83
59.77
57.48
56.95
55.19
52.00
43.55
38.71
37.72
36.95
36.92
36.20
O
OH
210 200 190 180 170 160 150 140 130 120 110 100 90 80 70 60 50 40 30 20 10 0 -10
f1 (ppm)

18

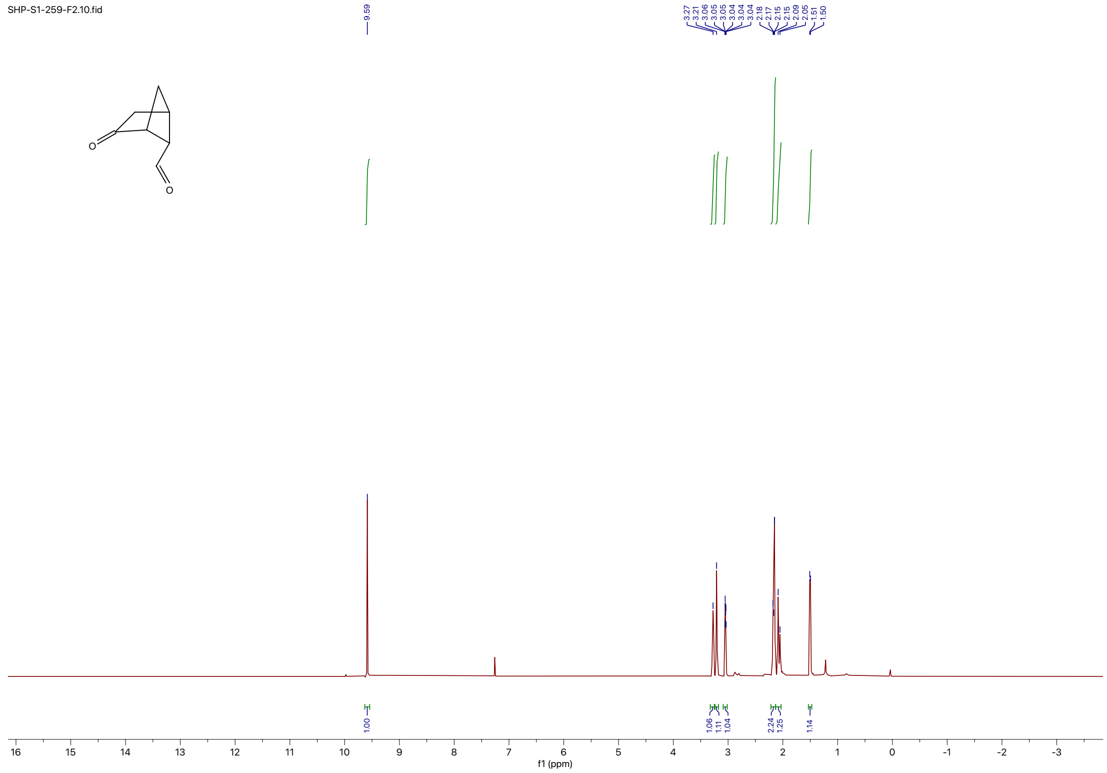
SHP-S1-259-F2.10.fid
9.59
3.27 3.21 3.06 3.05 3.05 3.04 3.04 3.04 2.18 2.17 2.15 2.15 2.09 2.06 1.51 1.50
O
O
1.00
1.06 1.11 1.04
2.24 1.25
1.14
16 15 14 13 12 11 10 9 8 7 6 5 4 3 2 1 0 -1 -2 -3
f1 (ppm)

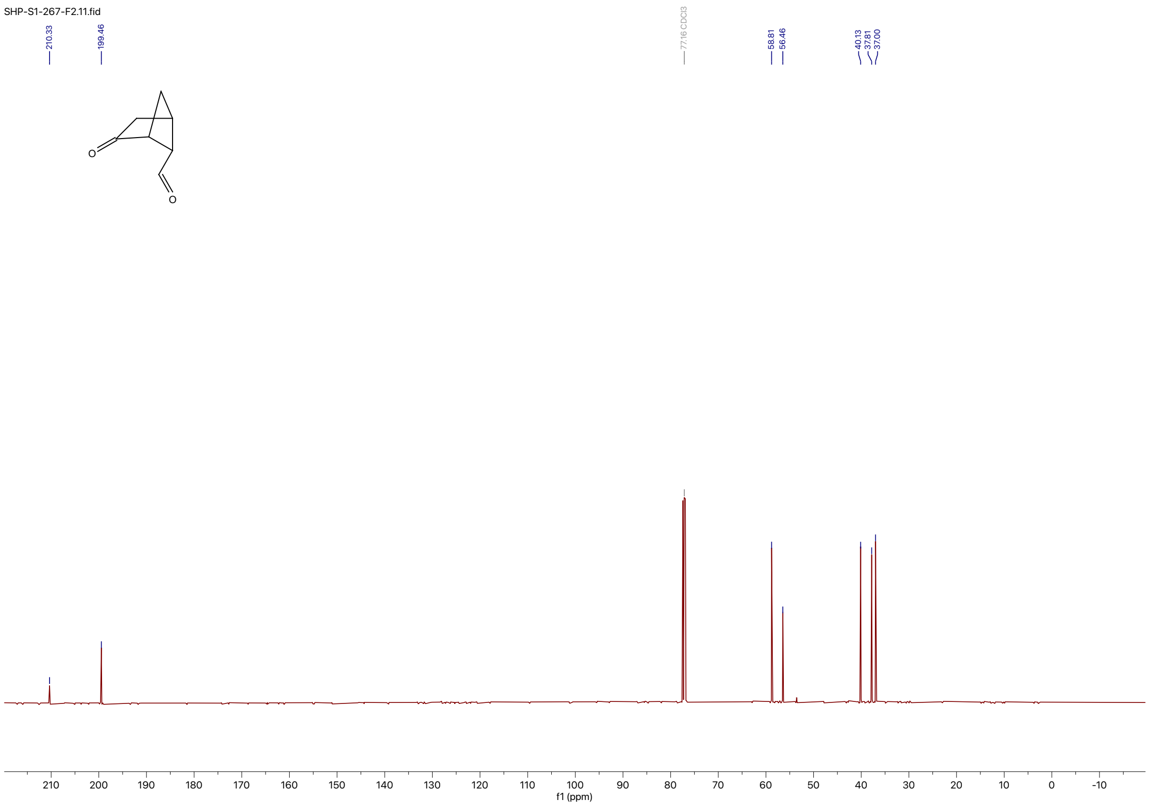
SHP-S1-267-F2.11.fid
210.33
199.46
77.16 CDCl3
58.81
56.46
40.13 37.81 37.00
O
O
210 200 190 180 170 160 150 140 130 120 110 100 90 80 70 60 50 40 30 20 10 0 -10
f1 (ppm)

19

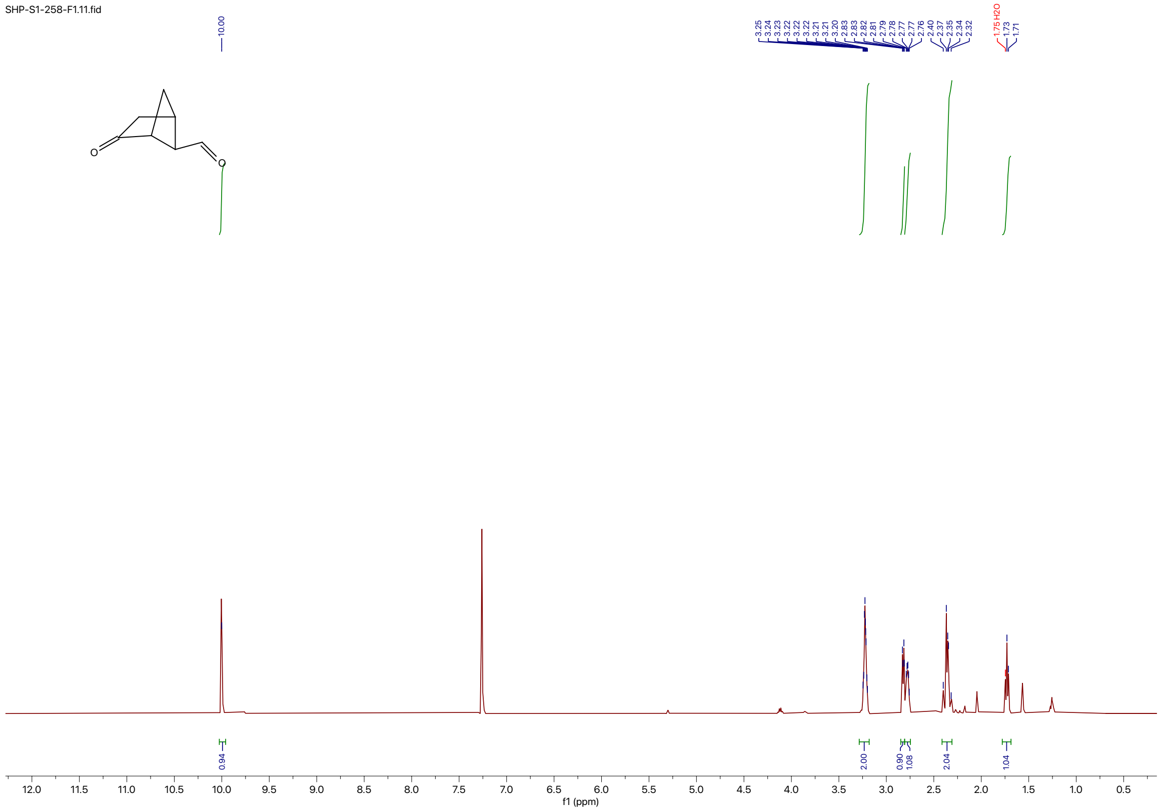
SHP-S1-258-F1.11.fid
10.00
3.25
3.24
3.23
3.22
3.22
3.22
3.21
3.21
3.20
2.83
2.83
2.82
2.81
2.79
2.78
2.77
2.77
2.76
2.40
2.37
2.35
2.34
2.32
1.75 H2O
1.73
1.71
0.94
2.00
0.90
1.08
2.04
1.04
f1 (ppm)

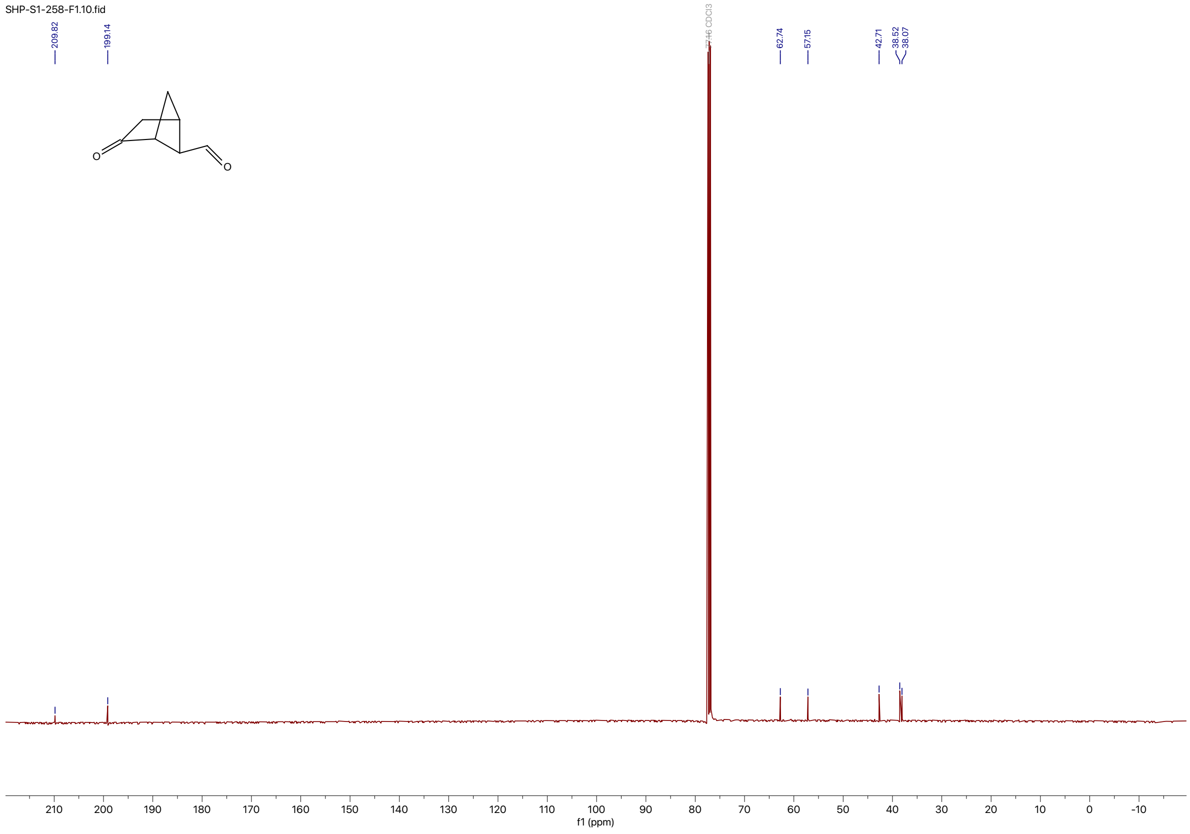
SHP-S1-258-F1.10.fid
209.82
199.14
77.16 CDCl3
62.74
57.15
42.71
38.52
38.07
f1 (ppm)

20

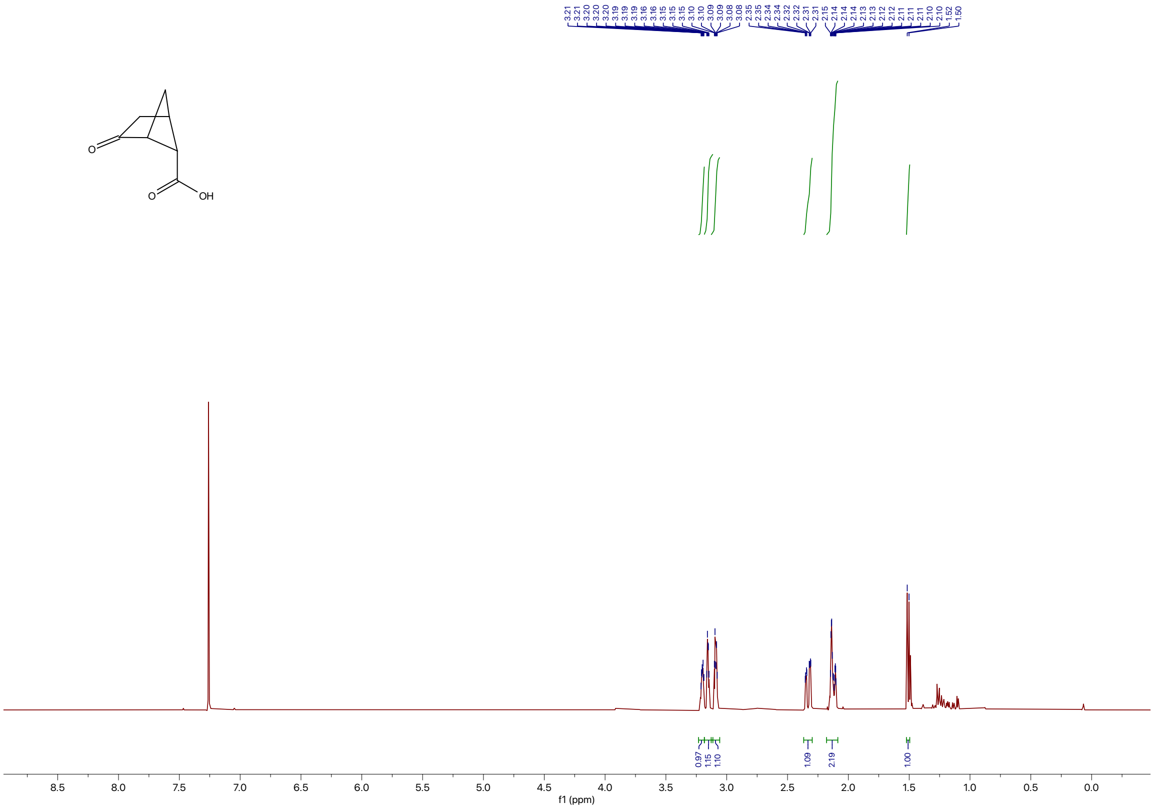

SHP-S1-274.11.fid

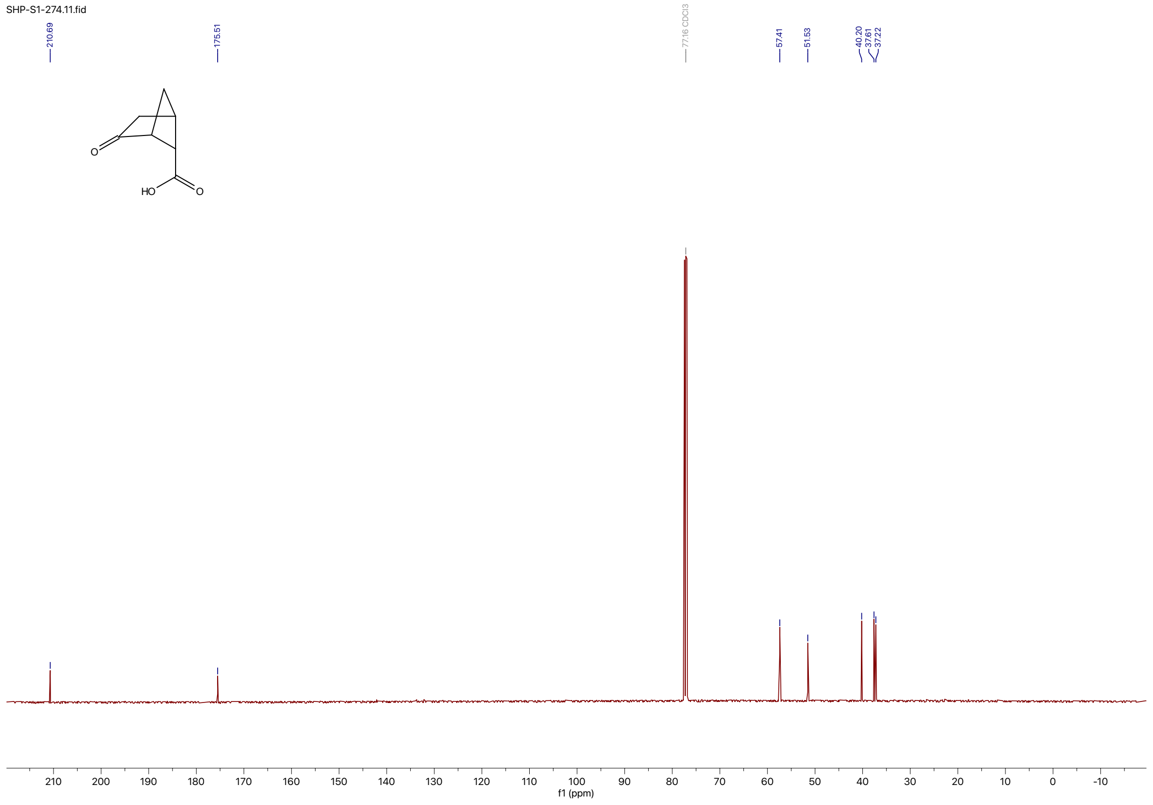

COSY analysis of 20

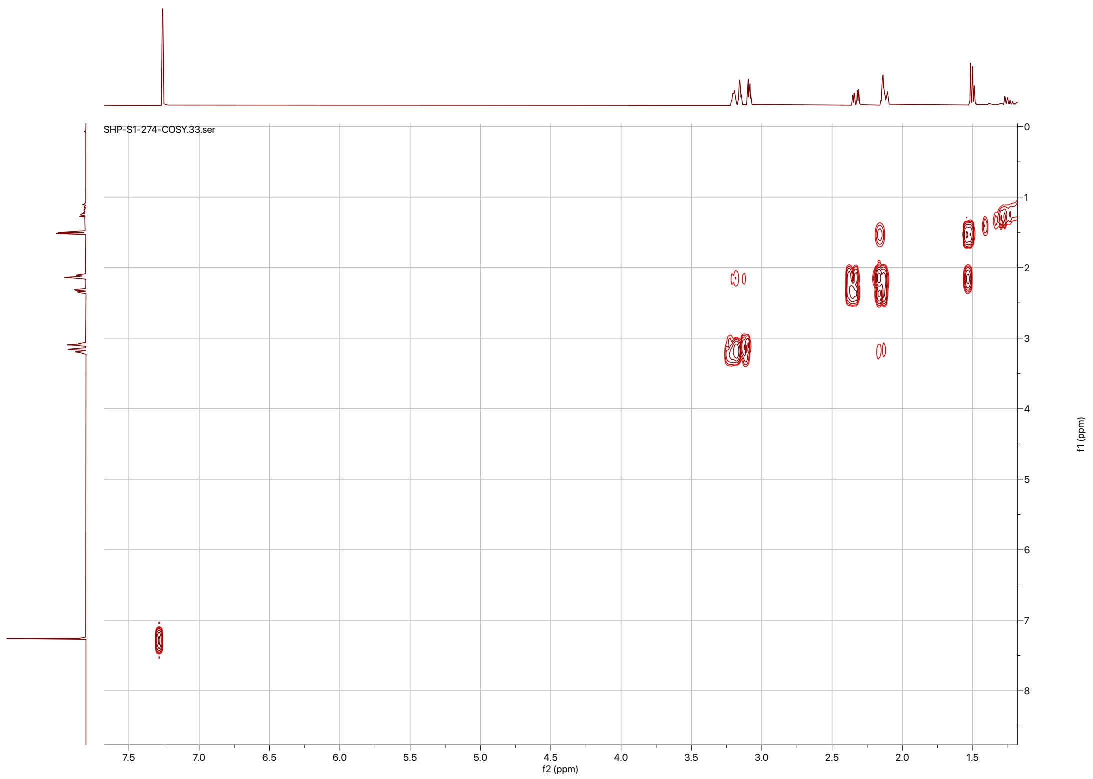

HMBC analysis of 20

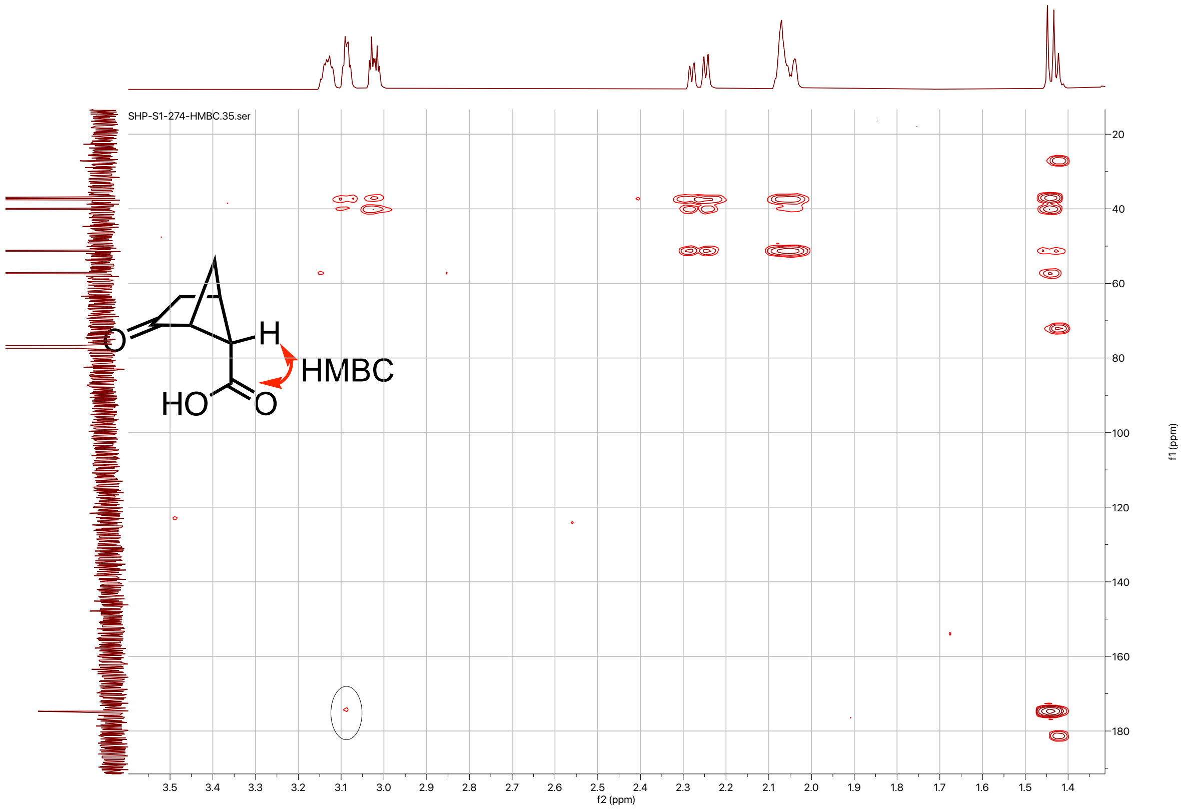

HSQC analysis of 20

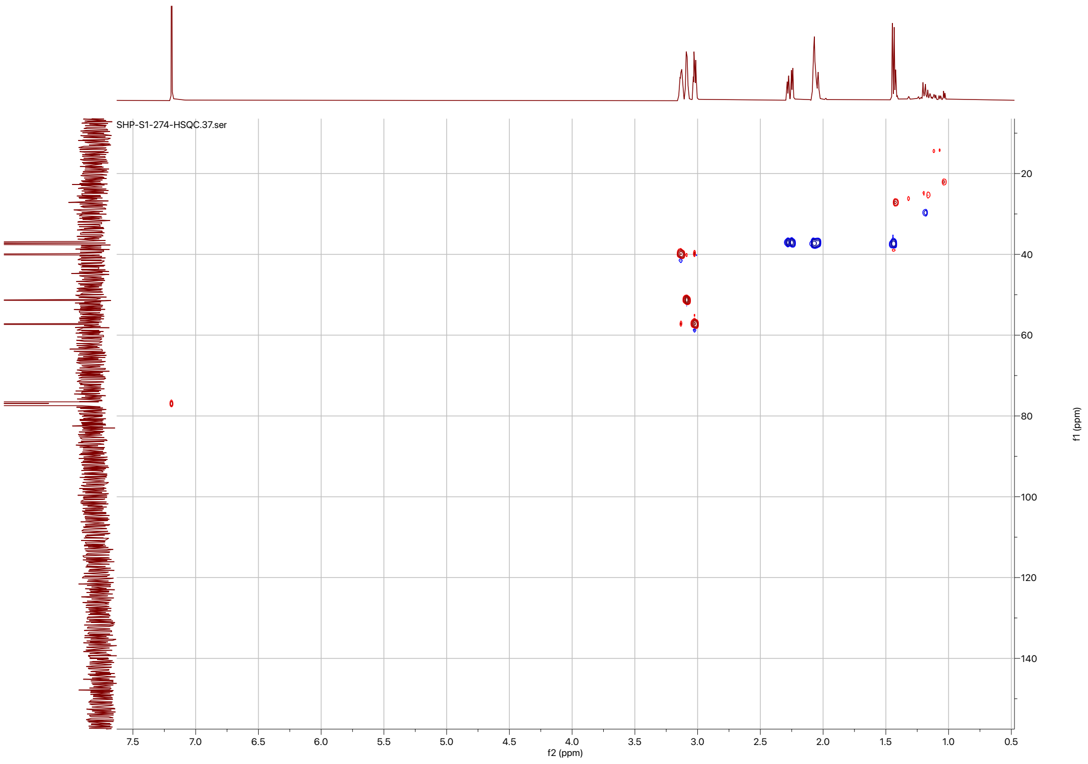

NOSEY analysis of 20

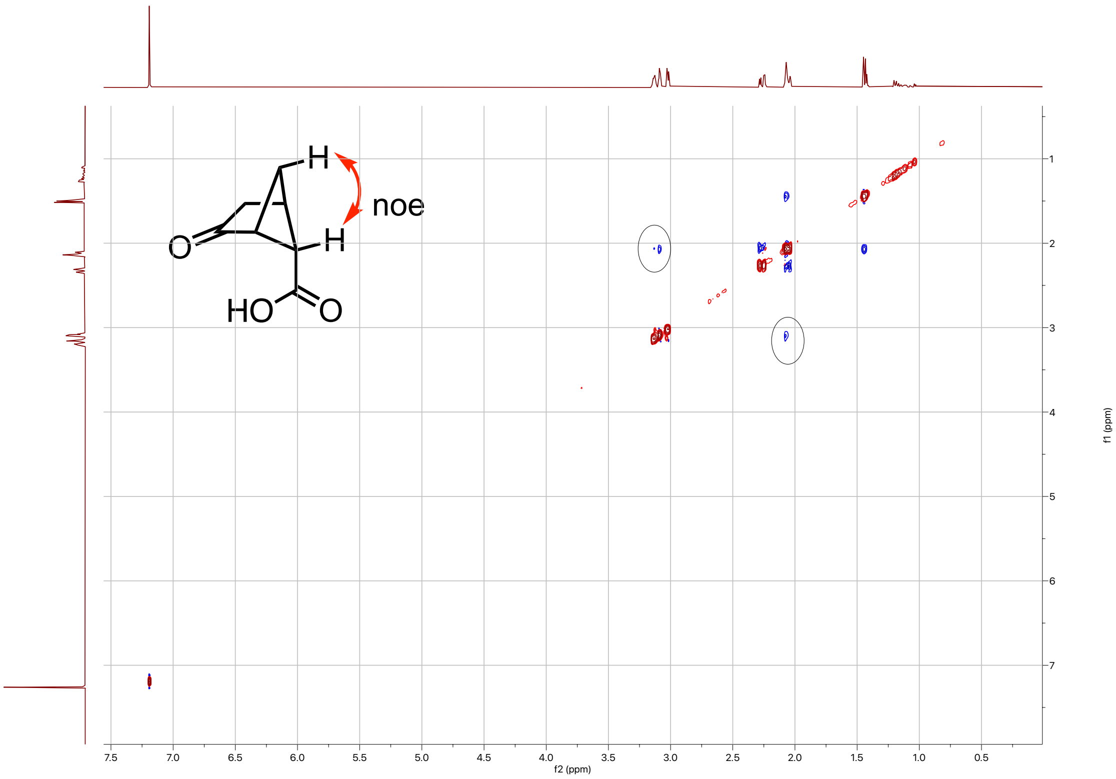

21

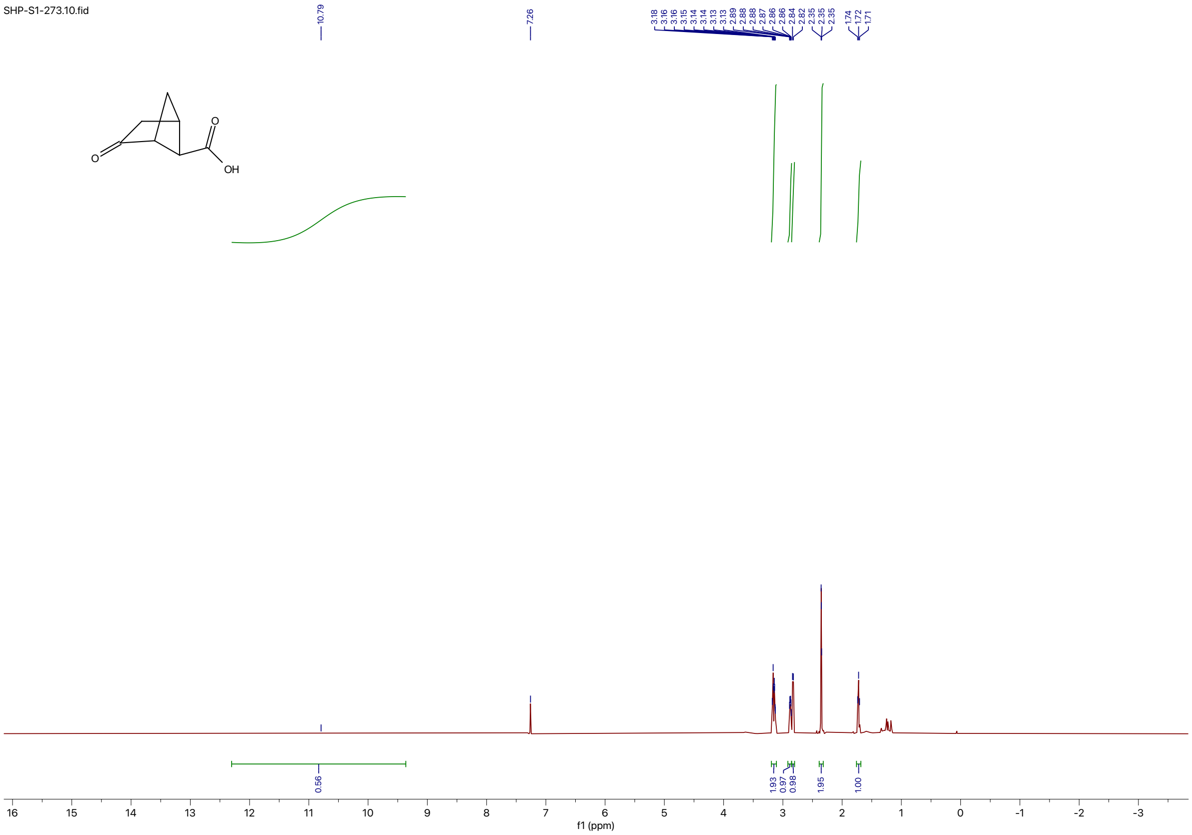
SHP-S1-273.10.fid
10.79
7.26
3.18 3.16 3.15 3.14 3.14 3.13 3.13 2.89 2.88 2.88 2.87 2.86 2.86 2.84 2.82 2.35 2.35 2.35 2.35 1.74 1.72 1.71
O
OH
0.56
1.93
0.97
0.98
1.95
1.00
f1 (ppm)

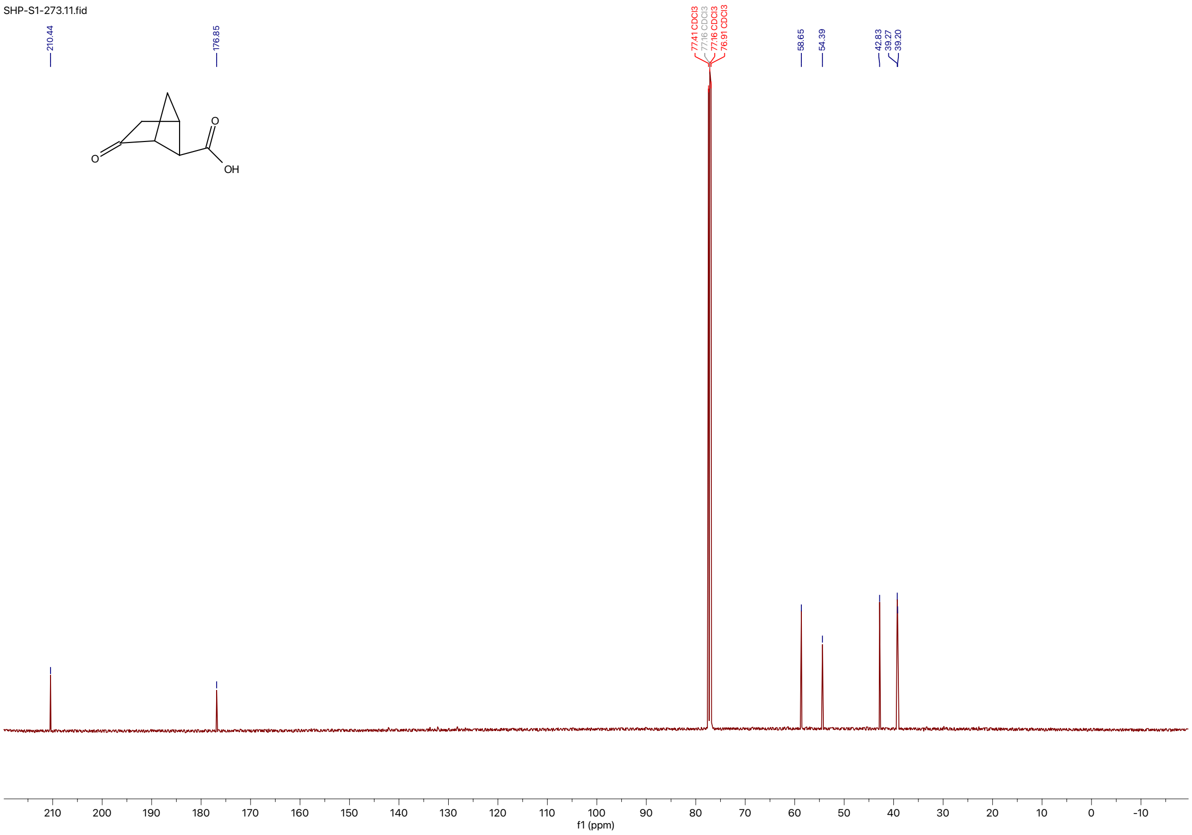
SHP-S1-273.11.fid
210.44
176.85
77.41 CDCl3
77.16 CDCl3
77.16 CDCl3
76.91 CDCl3
58.65
54.39
42.83
39.27
39.20
O
OH
f1 (ppm)

23

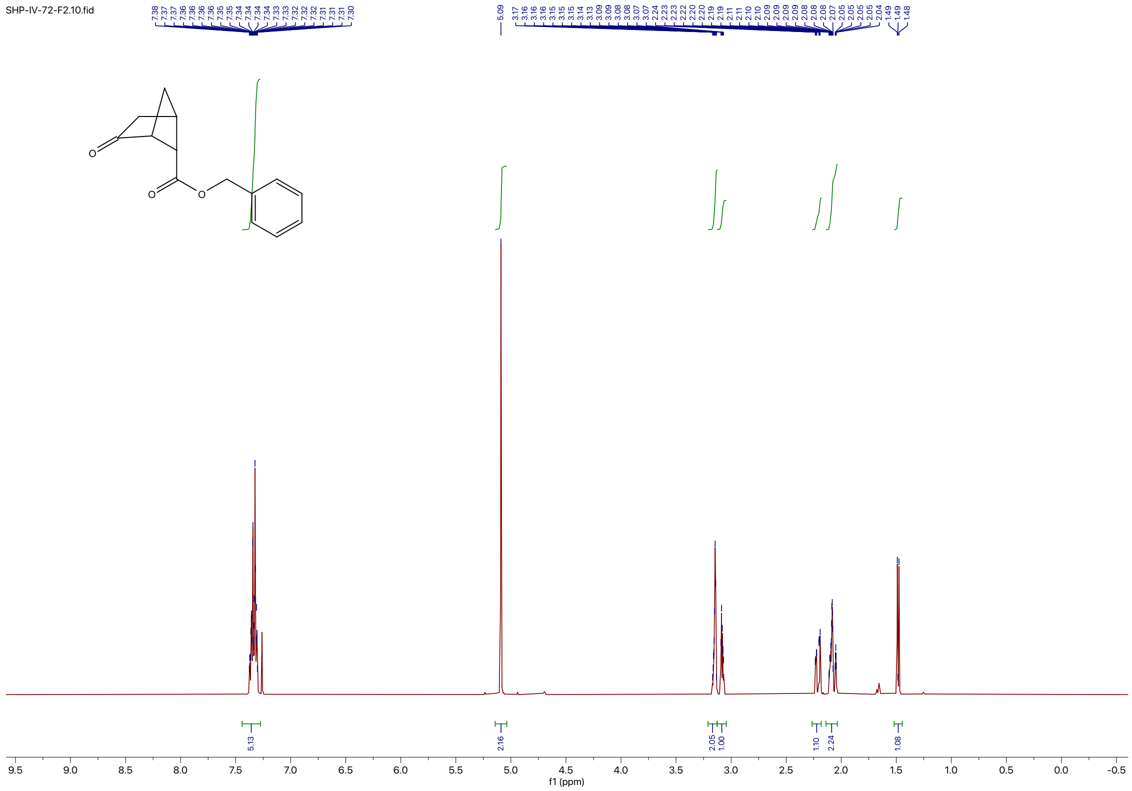
SHP-IV-72-F2.10.fid
f1 (ppm)

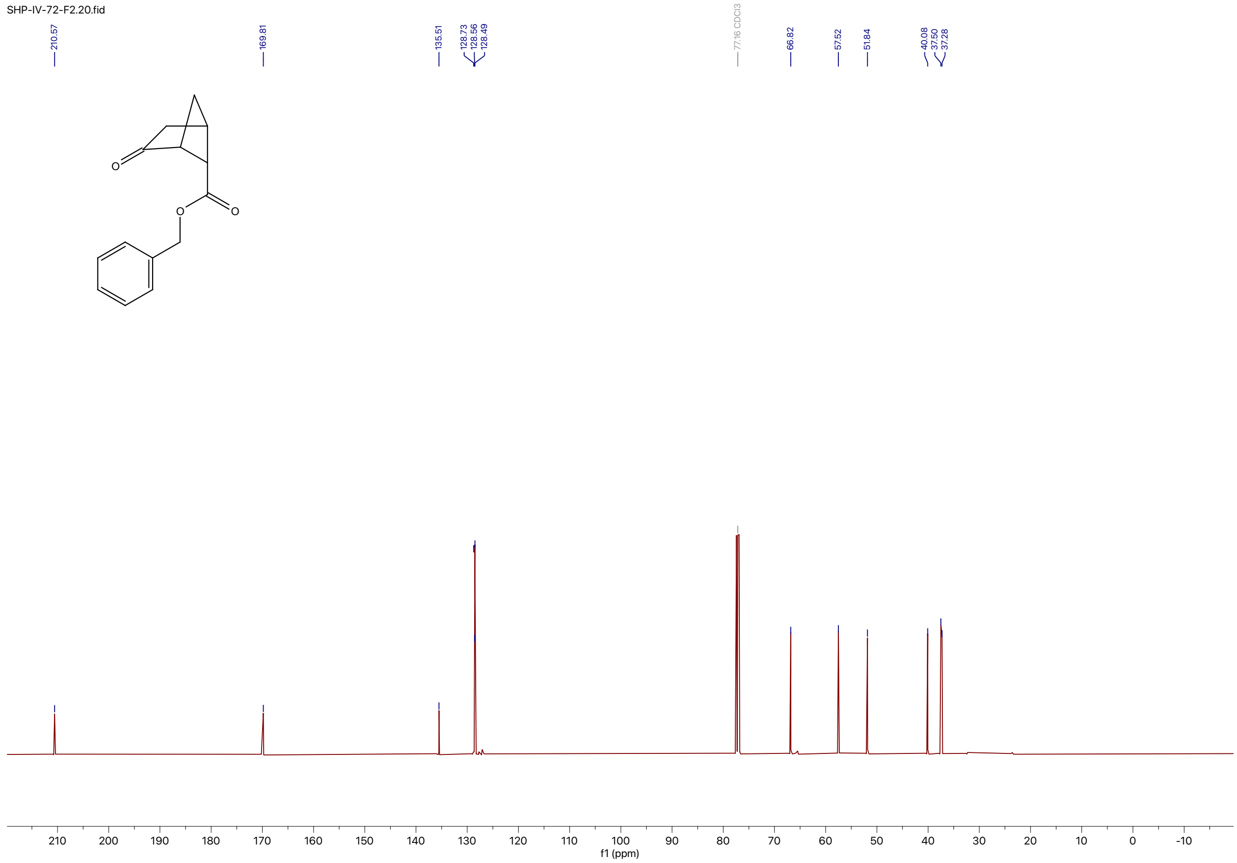
SHP-IV-72-F2.20.fid
210.57
169.81
135.51
128.73
128.56
128.49
77.16 CDCl3
66.82
57.52
51.84
40.08
37.50
37.28
f1 (ppm)

22

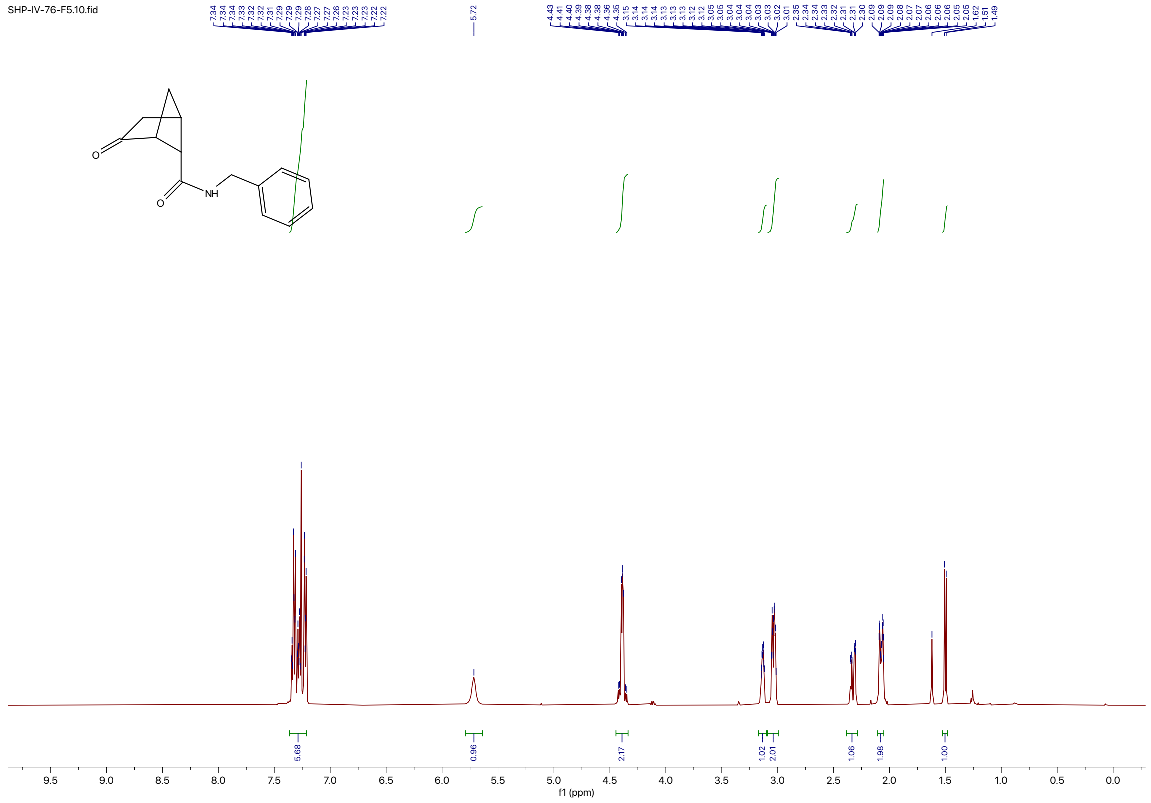
SHP-IV-76-F5.10.fid
7.34 7.34 7.33 7.32 7.32 7.31 7.29 7.29 7.28 7.27 7.27 7.26 7.23 7.23 7.22 7.22
5.72
4.43 4.41 4.40 4.39 4.38 4.38 4.36 4.35 3.15 3.14 3.14 3.13 3.13 3.13 3.12 3.12 3.05 3.05 3.04 3.04 3.04 3.03 3.03 3.02 3.01 2.35 2.34 2.34 2.33 2.32 2.31 2.31 2.30 2.09 2.09 2.09 2.08 2.07 2.07 2.06 2.06 2.06 2.05 2.05 1.62 1.51 1.49
O
O
NH
5.68
0.96
2.17
1.02
2.01
1.06
1.98
1.00
f1 (ppm)

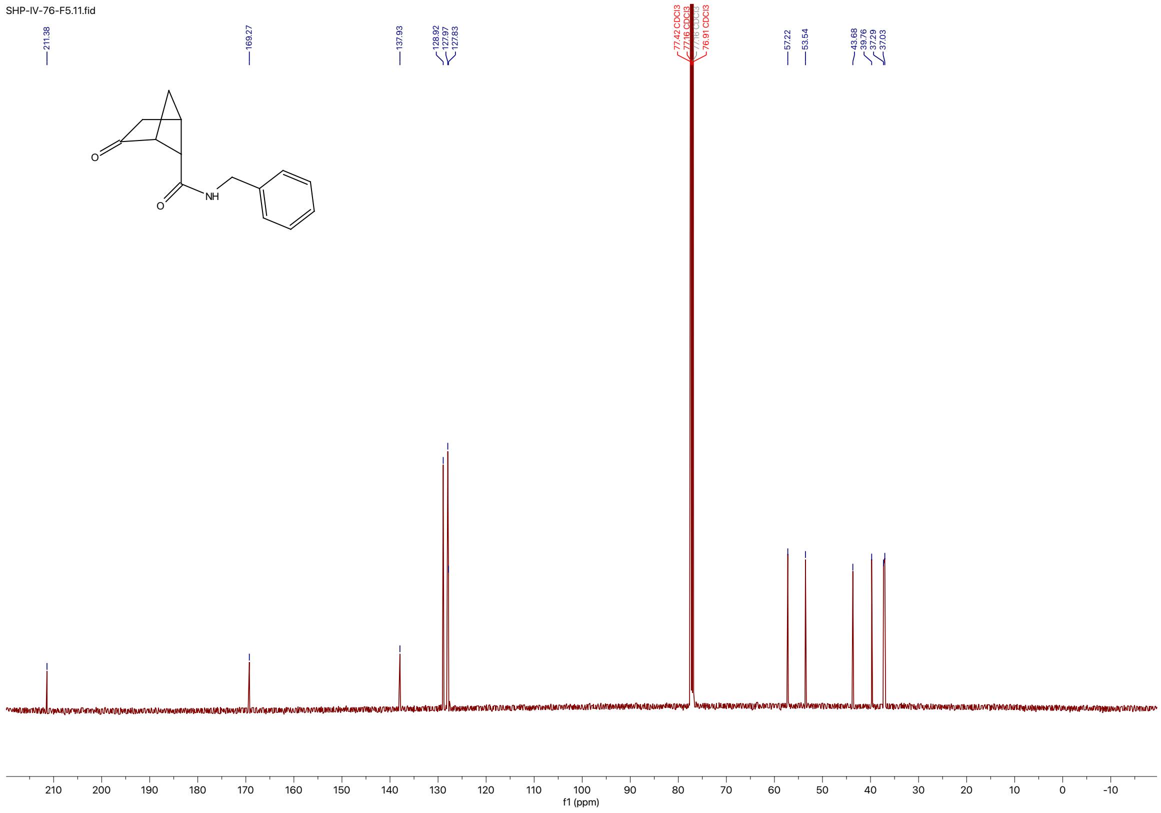
SHP-IV-76-F5.11.fid
211.38
169.27
137.93
128.92 127.97 127.83
77.42 CDCl3 77.16 CDCl3 76.91 CDCl3
57.22
53.54
43.68 39.76 37.29 37.03
O
O
NH
f1 (ppm)

27

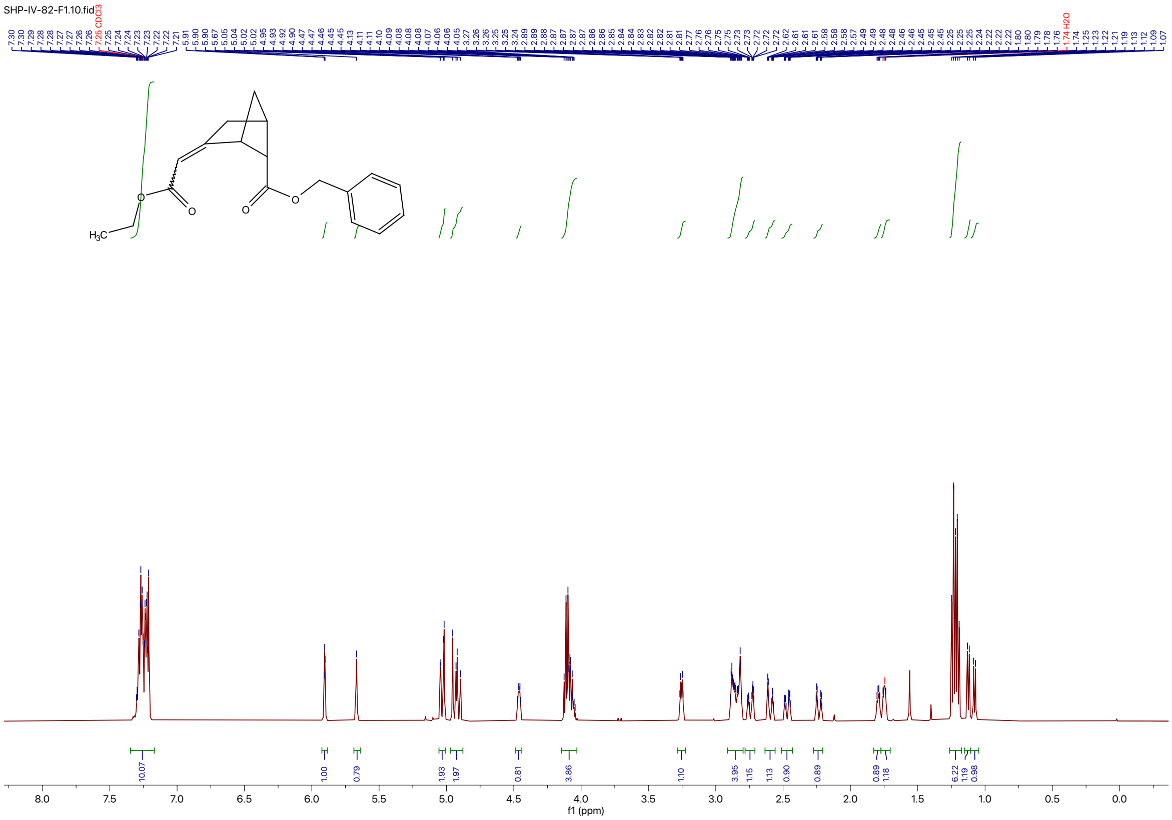

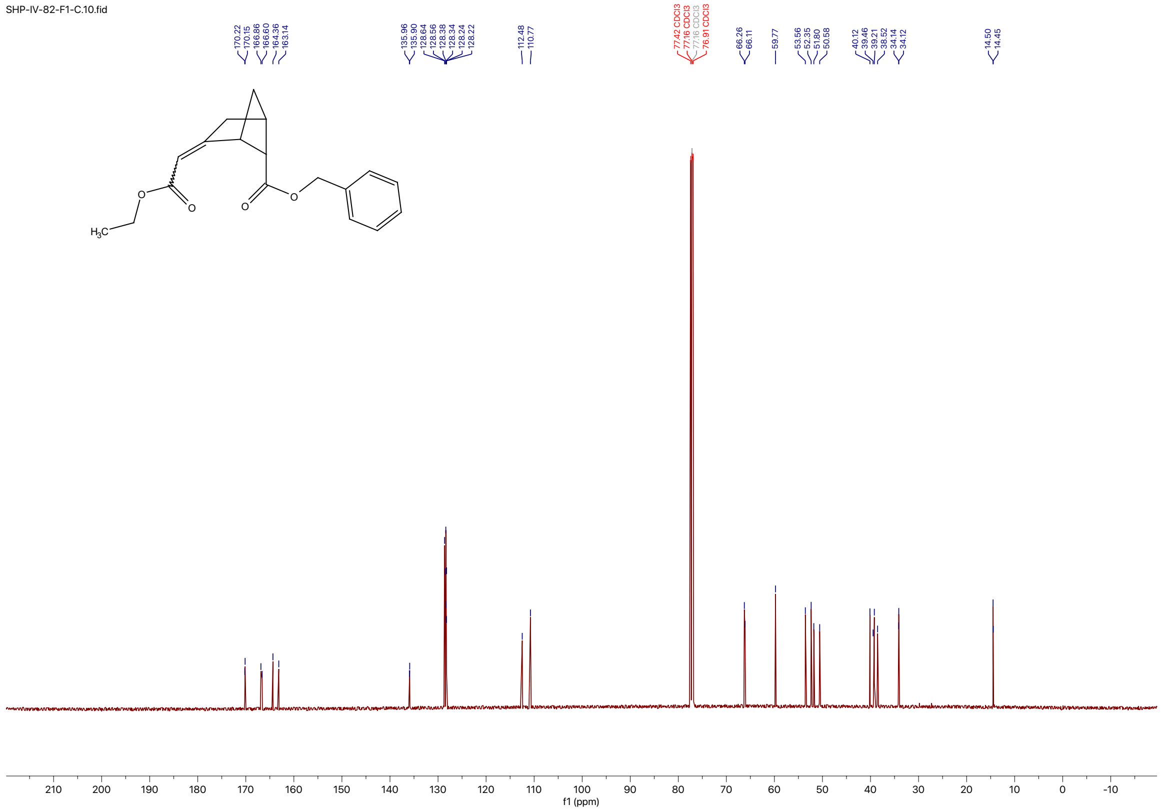

24

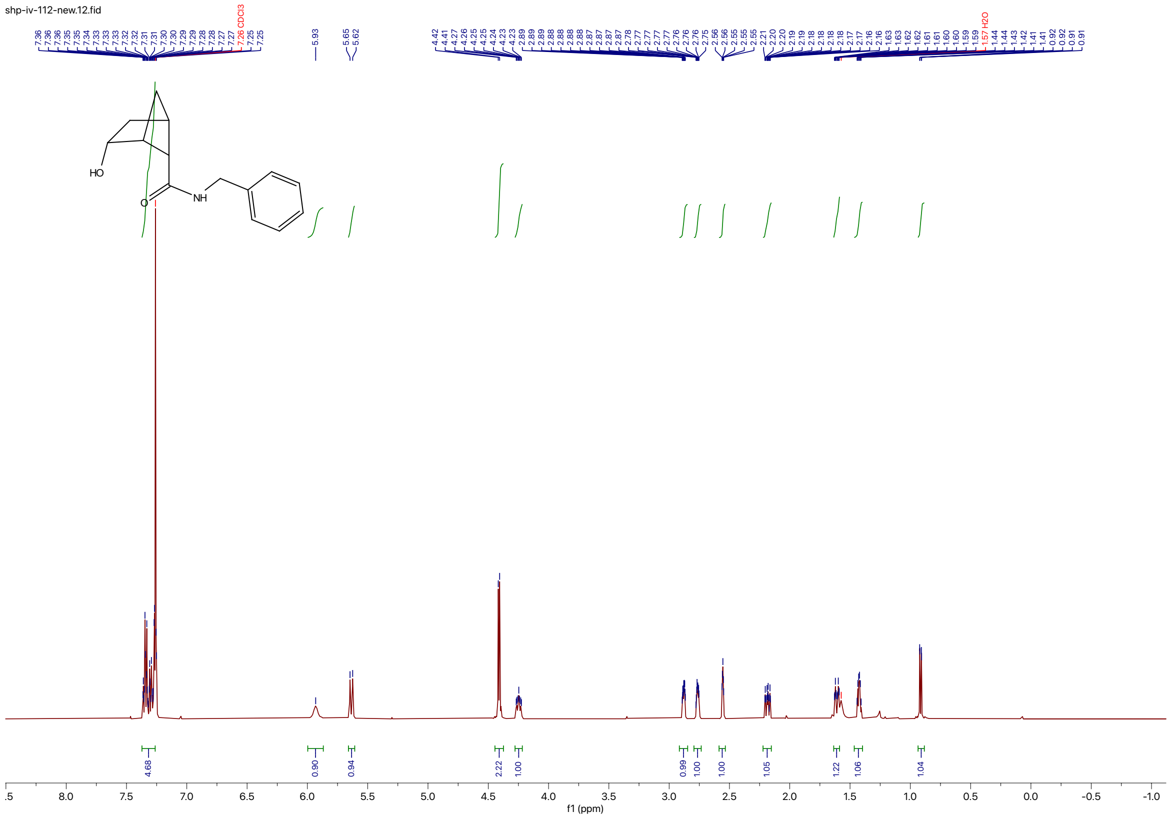
shp-iv-112-new.12.fid
HO
O
NH
f1 (ppm)

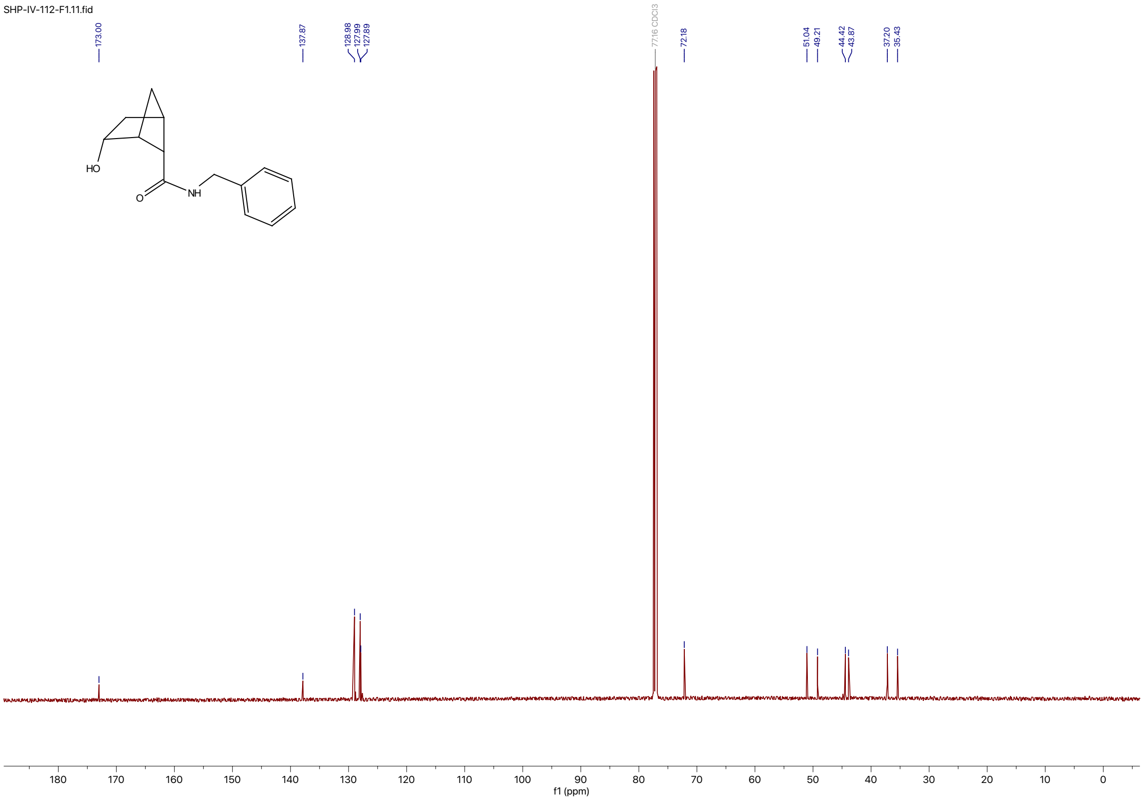
SHP-IV-112-F1.11.fid
173.00
137.87
128.98
127.99
127.89
77.16 CDCl3
72.18
51.04
49.21
44.42
43.87
37.20
35.43
HO
O
NH
f1 (ppm)

COSY analysis of 24

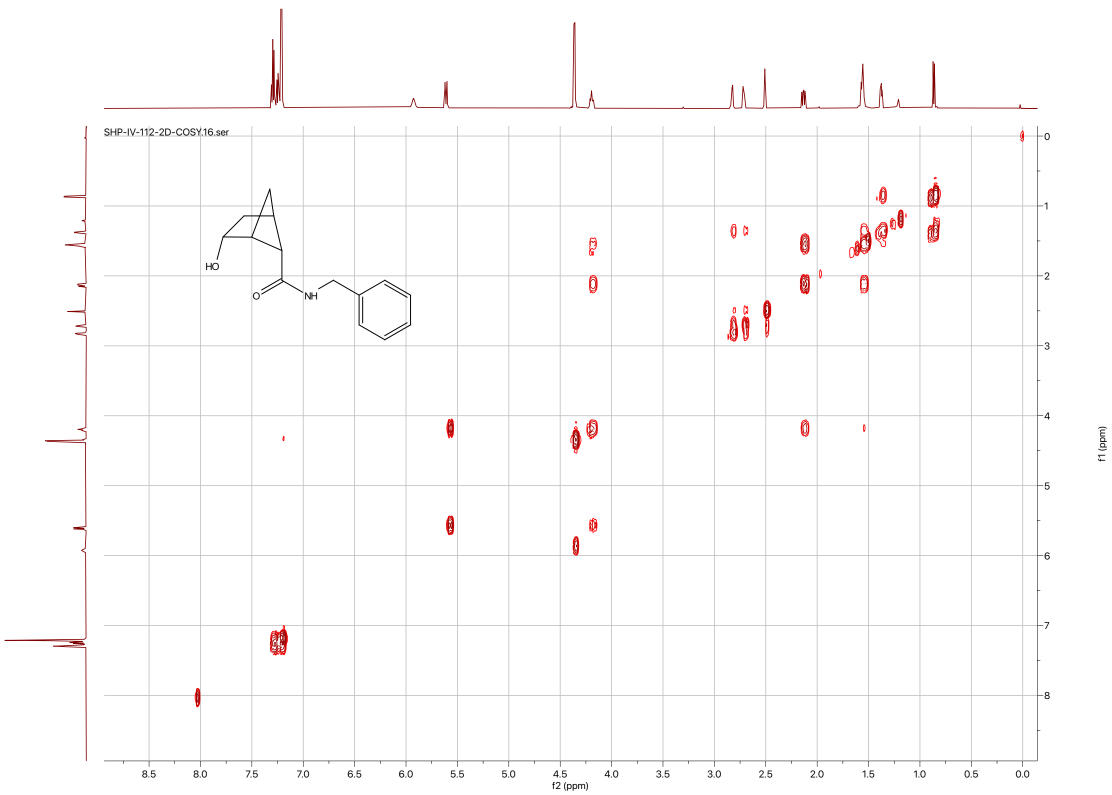

HSQC analysis of 24

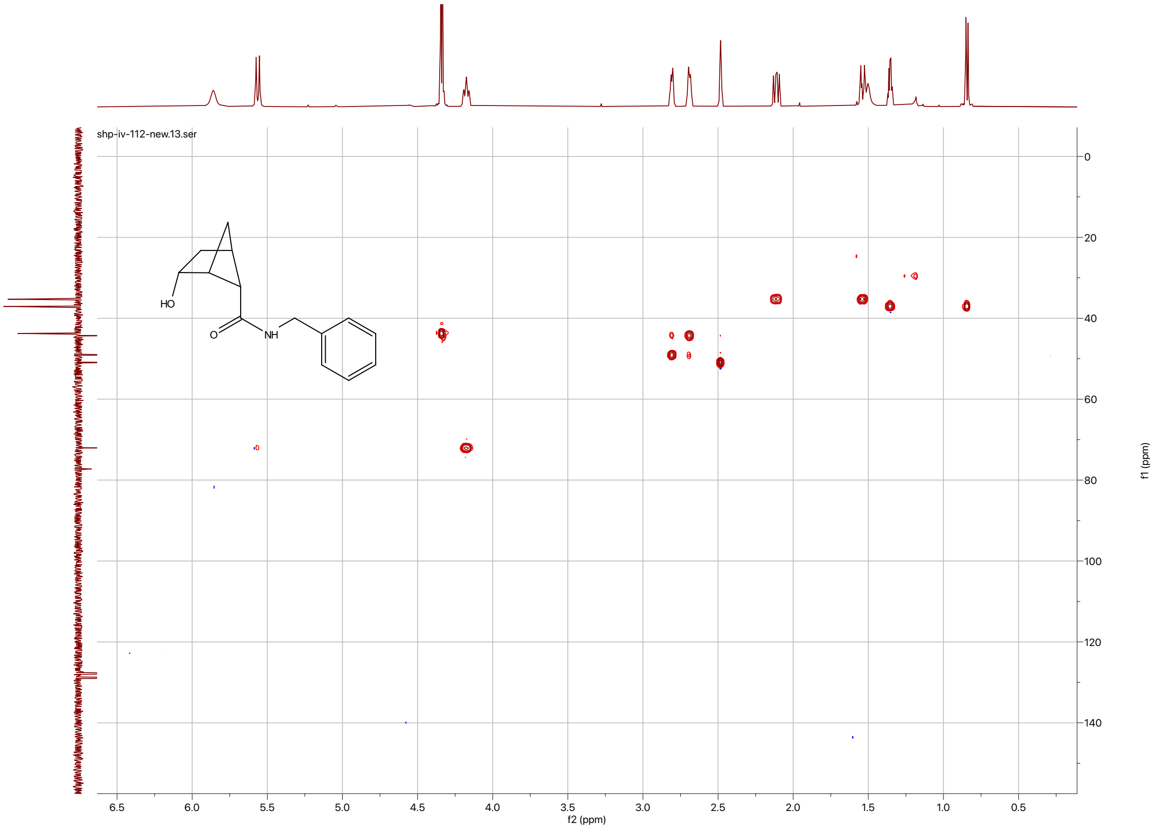

NOSEY analysis of 24

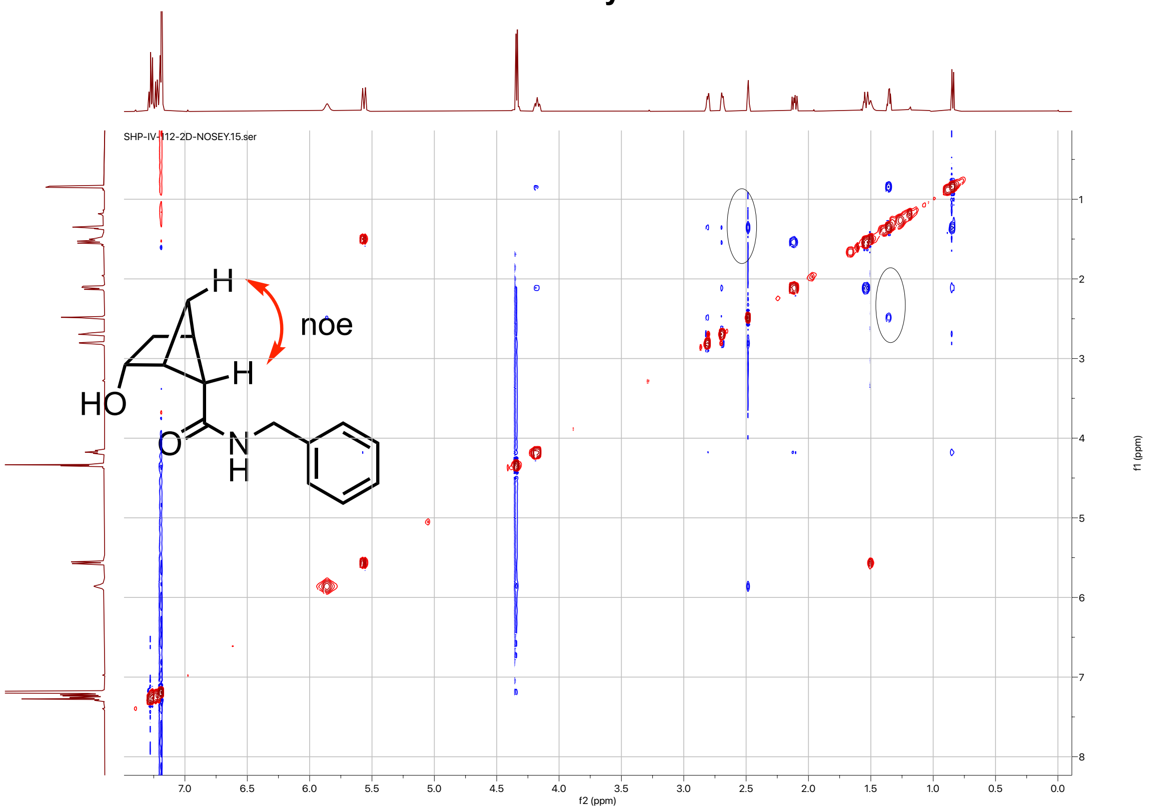

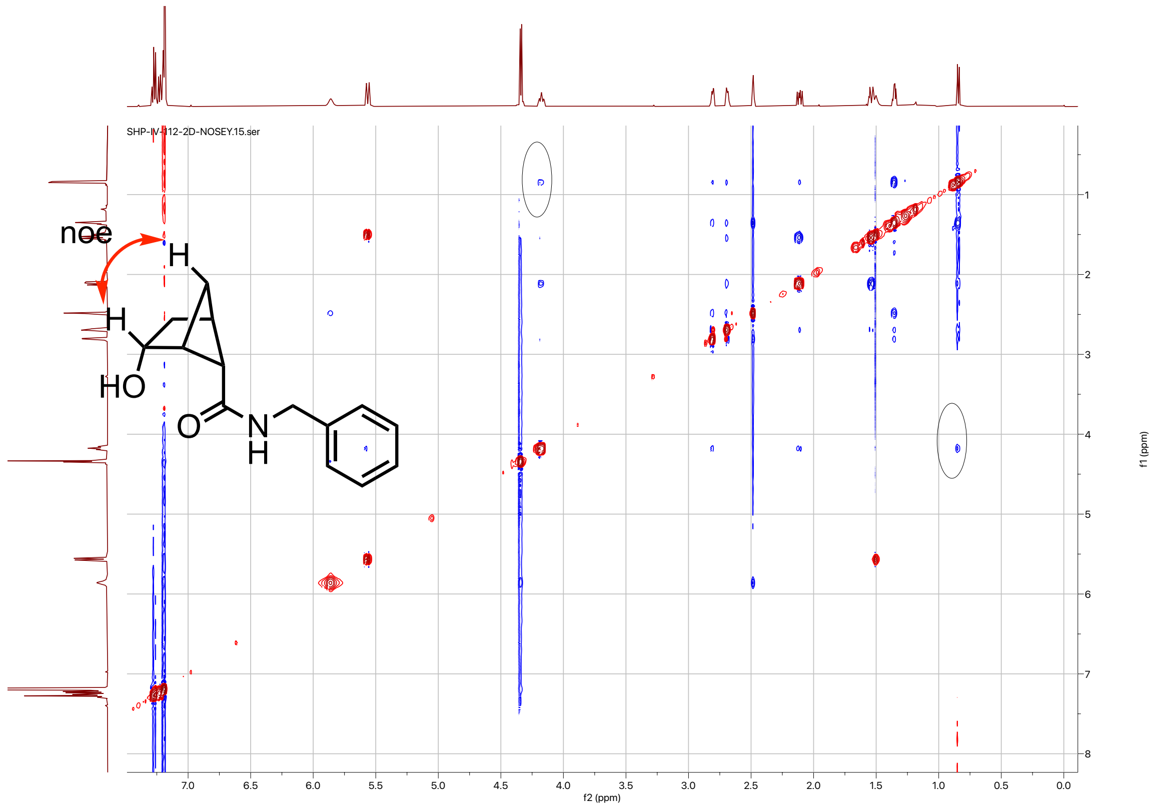

25

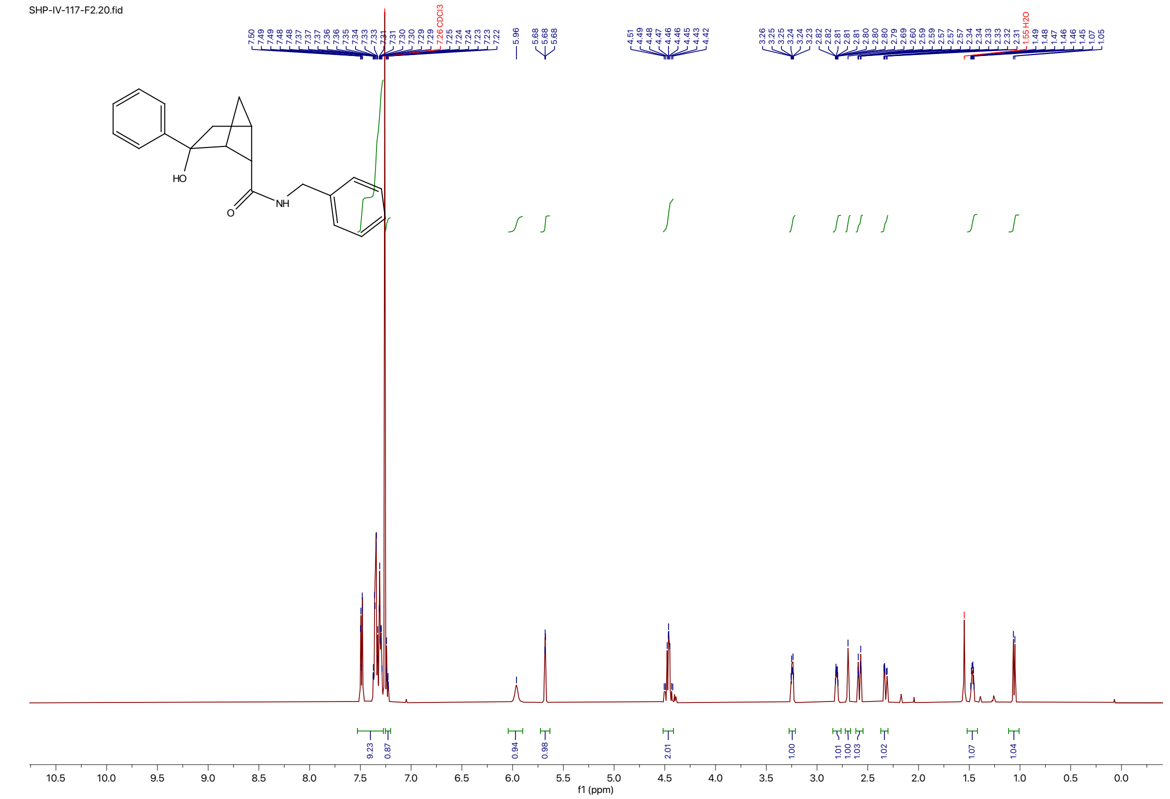
SHP-IV-117-F2.20.fid
7.50 7.49 7.49 7.48 7.37 7.37 7.37 7.36 7.36 7.35 7.34 7.33 7.33 7.31 7.31 7.30 7.30 7.29 7.29 7.26 CDCl3 7.25 7.24 7.24 7.23 7.23 7.22
5.96 5.68 5.68 5.68
4.51 4.49 4.48 4.47 4.46 4.46 4.46 4.45 4.43 4.42
3.26 3.25 3.25 3.24 3.24 3.23 2.82 2.82 2.81 2.81 2.81 2.80 2.80 2.80 2.79 2.69 2.60 2.59 2.59 2.57 2.57 2.57 2.34 2.34 2.33 2.33 2.32 2.31
1.55 H2O
1.49 1.48 1.47 1.46 1.46 1.45 1.07 1.05
HO
NH
O
9.23 0.87 0.94 0.98 2.01 1.00 1.01 1.00 1.03 1.02 1.07 1.04
f1 (ppm)

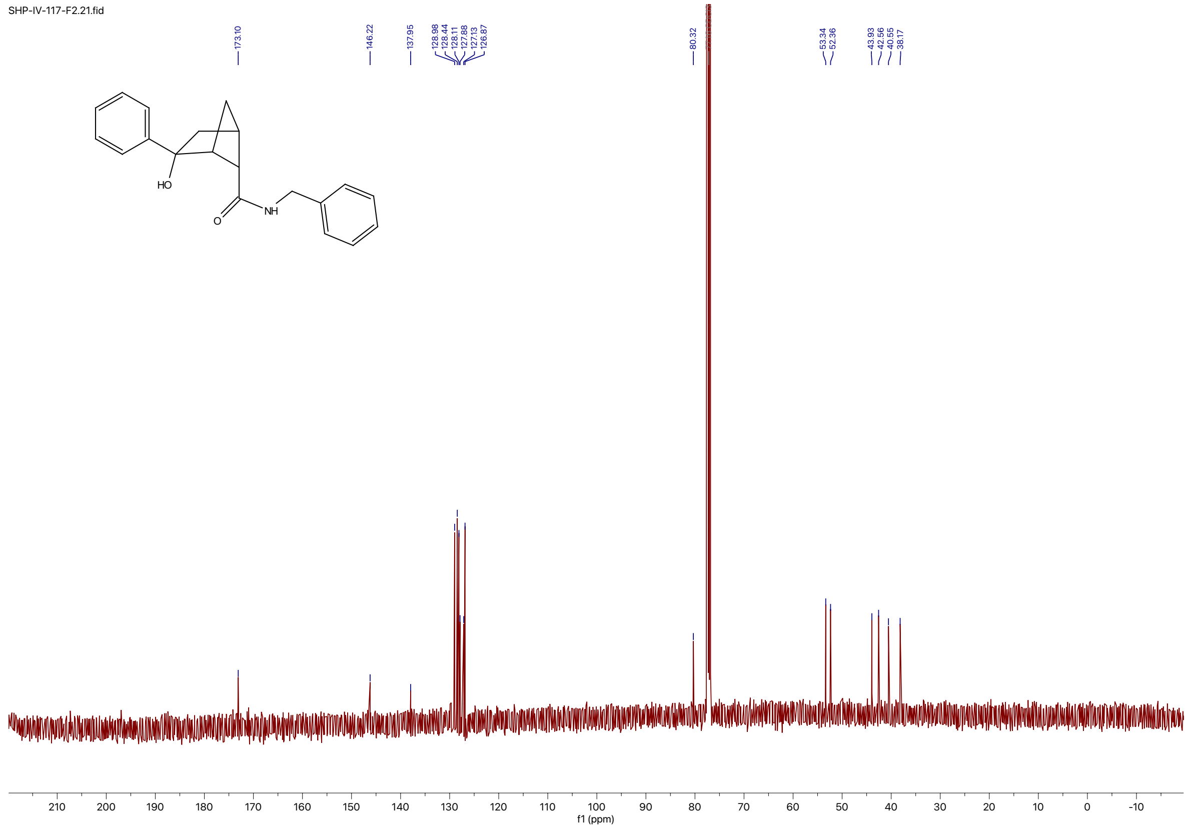
SHP-IV-117-F2.21.fid
173.10
146.22
137.95
128.98 128.44 128.11 127.88 127.13 126.87
80.32
53.34 52.36
43.93 42.56 40.55 38.17
HO
NH
O
f1 (ppm)

26

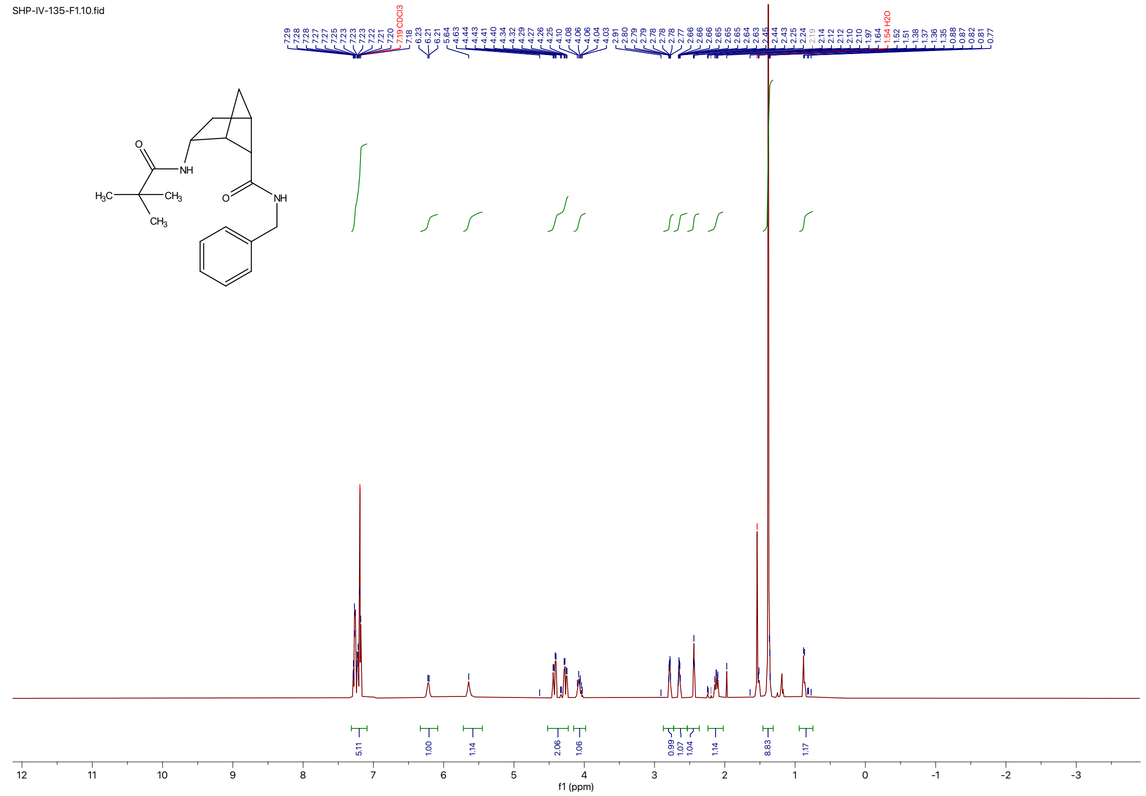
SHP-IV-135-F1.10.fid
7.29 7.28 7.28 7.27 7.27 7.25 7.23 7.23 7.23 7.22 7.21 7.20 7.19 CDCl3 7.18 6.23 6.21 6.21 5.64 4.63 4.44 4.43 4.41 4.40 4.34 4.32 4.29 4.27 4.26 4.25 4.10 4.08 4.06 4.06 4.04 4.03 2.91 2.80 2.79 2.78 2.78 2.77 2.66 2.66 2.65 2.65 2.65 2.64 2.63 2.45 2.44 2.43 2.25 2.24 2.14 2.12 2.12 2.10 2.10 1.97 1.64 1.54 H2O 1.52 1.51 1.38 1.37 1.36 1.35 0.88 0.87 0.82 0.81 0.77
H3C CH3 CH3 O NH O NH
5.11 1.00 1.14 2.06 1.06 0.99 1.07 1.04 1.14 8.83 1.17
12 11 10 9 8 7 6 5 4 3 2 1 0 -1 -2 -3
f1 (ppm)

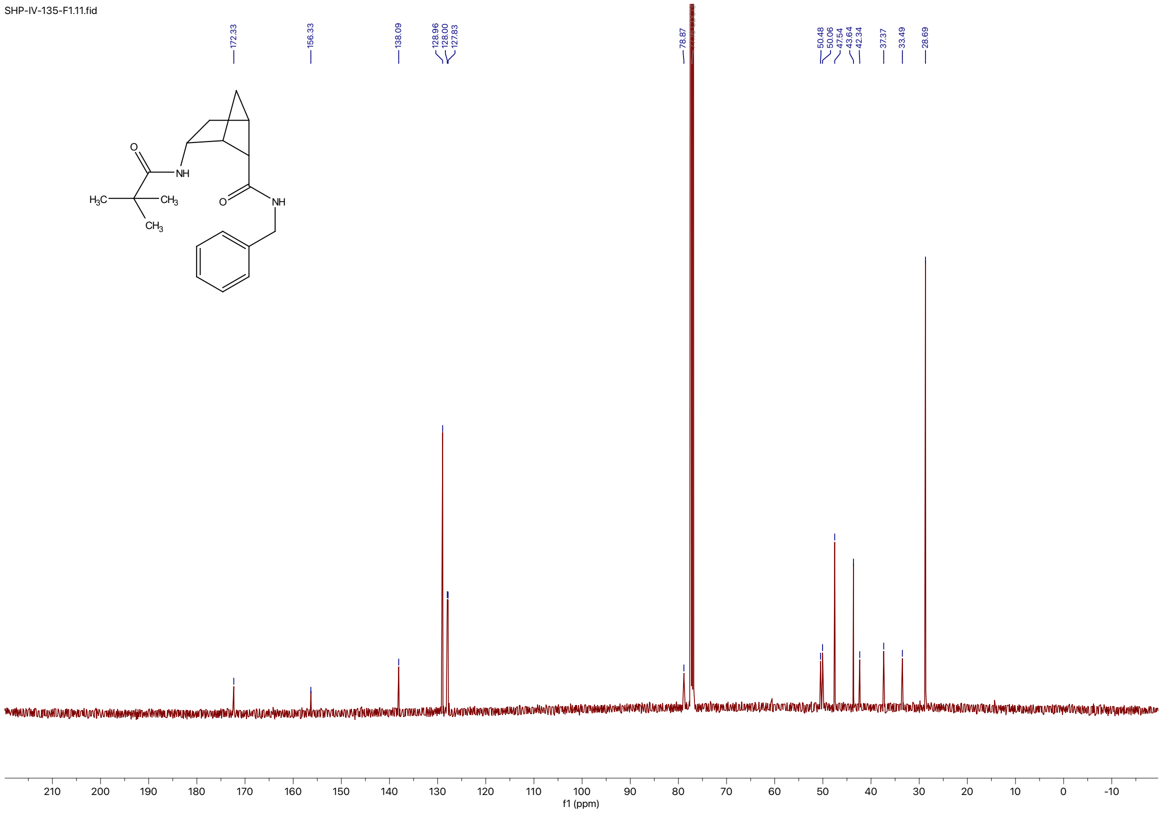
SHP-IV-135-F1.11.fid
172.33 156.33 138.09 128.96 128.00 127.83 78.87 50.48 50.06 47.54 43.64 42.34 37.37 33.49 28.69
H3C CH3 CH3 O NH O NH
210 200 190 180 170 160 150 140 130 120 110 100 90 80 70 60 50 40 30 20 10 0 -10
f1 (ppm)

SI-11

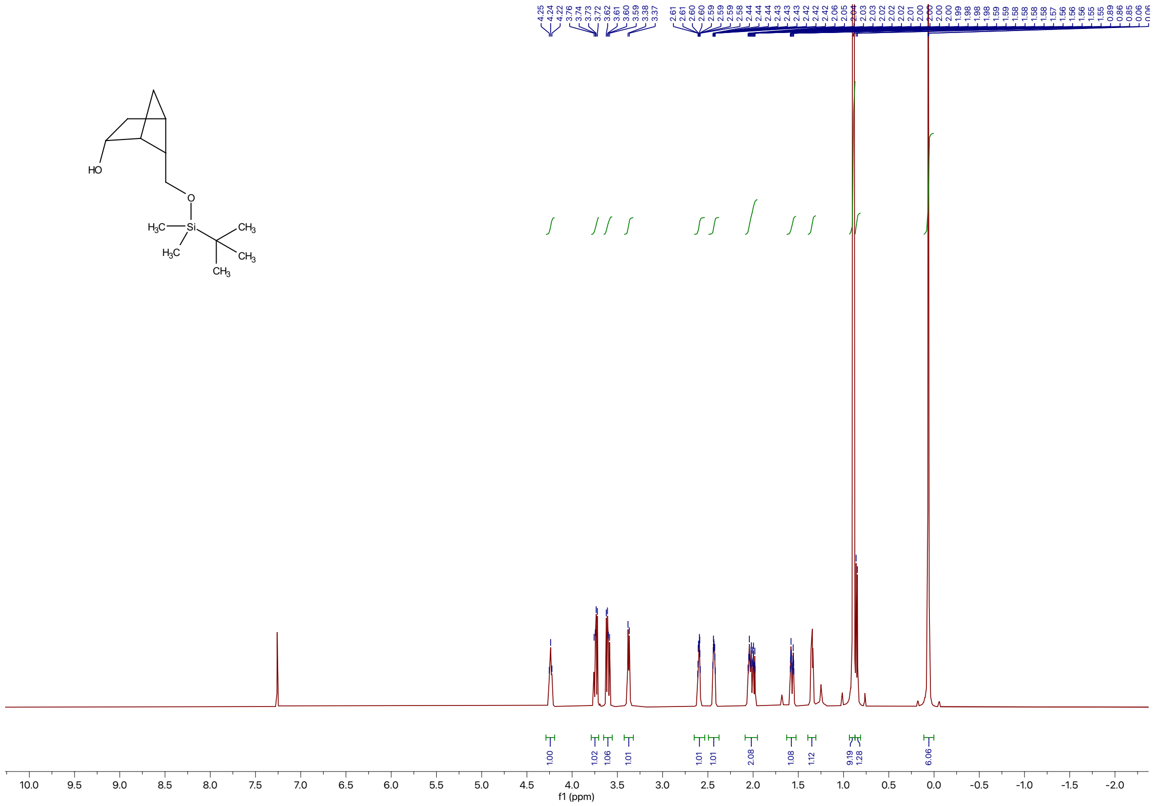

SHP-IV-280-F1.21.fid

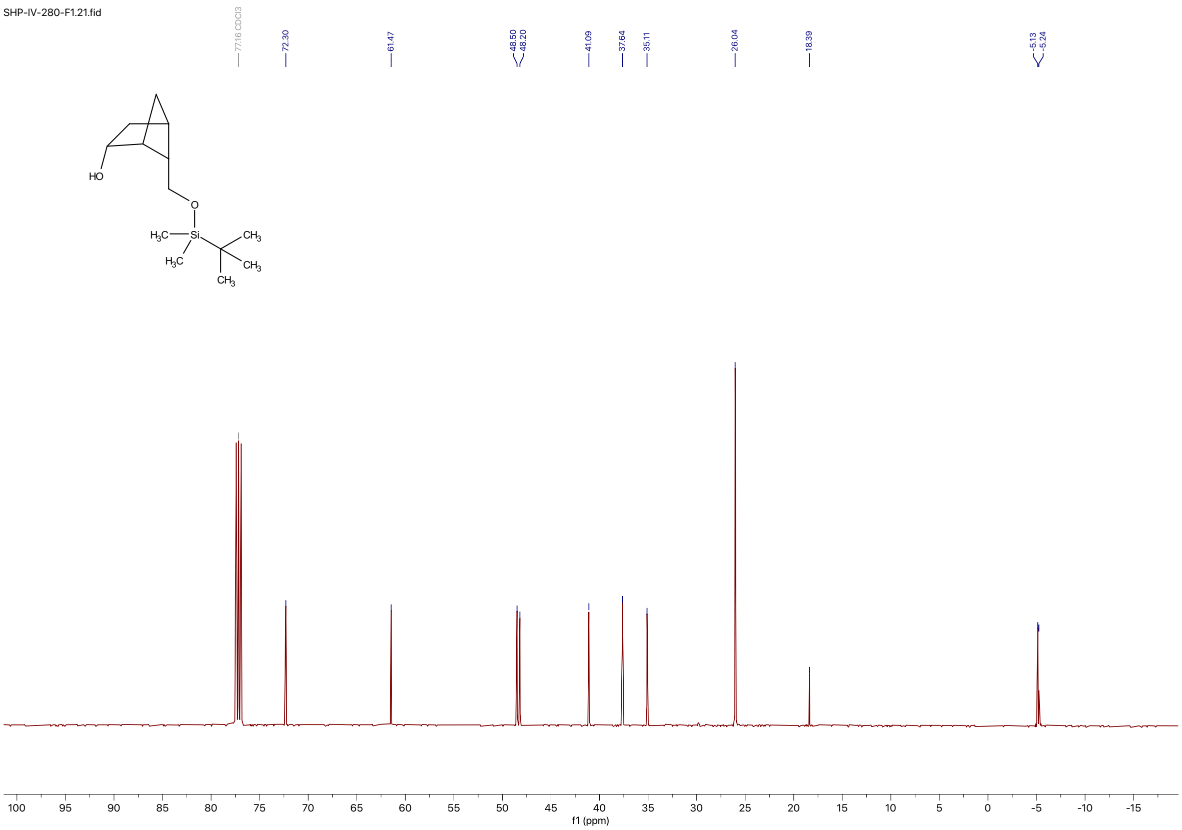

SI-12

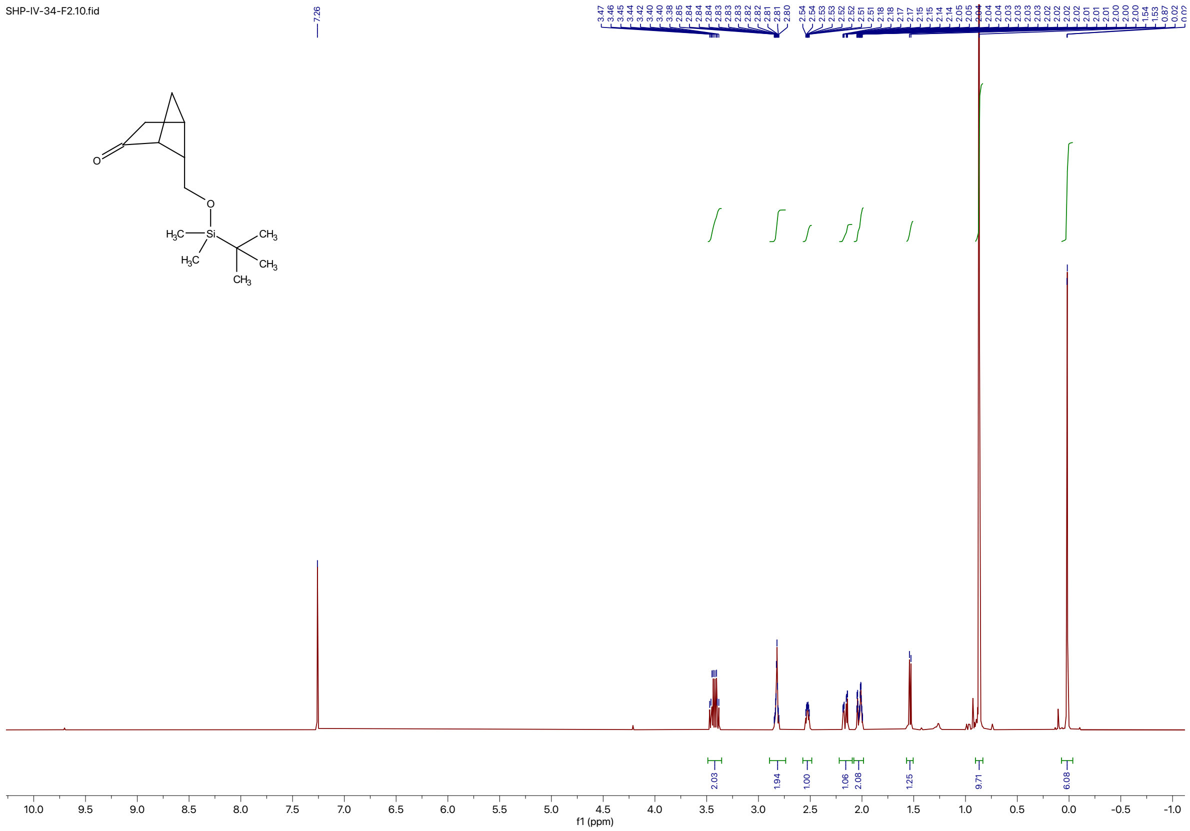
SHP-IV-34-F2.10.fid
7.26
3.47 3.46 3.45 3.44 3.42 3.40 3.40 3.38 2.85 2.84 2.84 2.83 2.83 2.83 2.82 2.82 2.81 2.80 2.54 2.54 2.53 2.53 2.52 2.52 2.51 2.51 2.18 2.18 2.17 2.17 2.15 2.15 2.14 2.14 2.05 2.05 2.04 2.04 2.03 2.03 2.03 2.02 2.02 2.02 2.01 2.01 2.00 2.00 2.00 1.54 1.53 0.87 0.02
O
H_3C
Si
CH_3
H_3C
CH_3
CH_3
2.03
1.94
1.00
1.06
2.08
1.25
9.71
6.08
10.0 9.5 9.0 8.5 8.0 7.5 7.0 6.5 6.0 5.5 5.0 4.5 4.0 3.5 3.0 2.5 2.0 1.5 1.0 0.5 0.0 -0.5 -1.0
f1 (ppm)

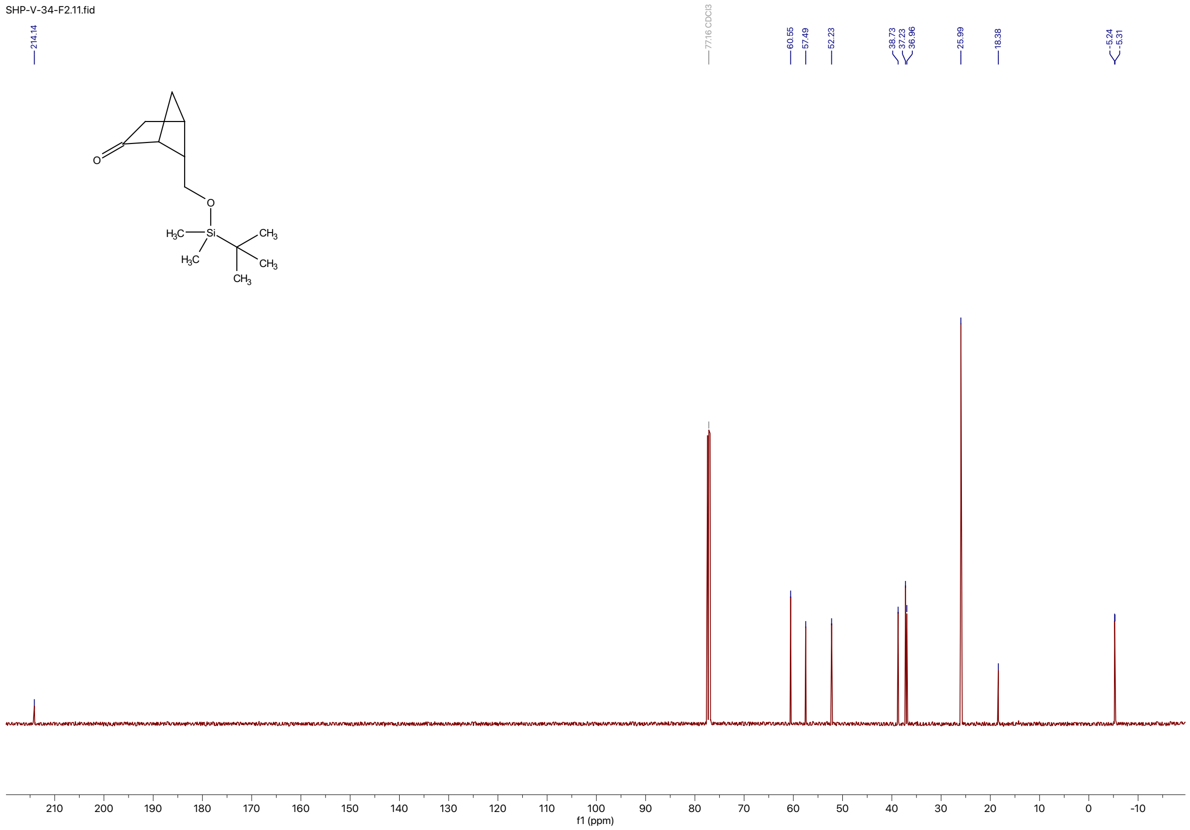
SHP-V-34-F2.11.fid
214.14
77.16 CDCl3
60.55
57.49
52.23
38.73
37.23
36.96
25.99
18.38
-5.24
-5.31
O
H_3C
Si
CH_3
H_3C
CH_3
CH_3
210 200 190 180 170 160 150 140 130 120 110 100 90 80 70 60 50 40 30 20 10 0 -10
f1 (ppm)

28

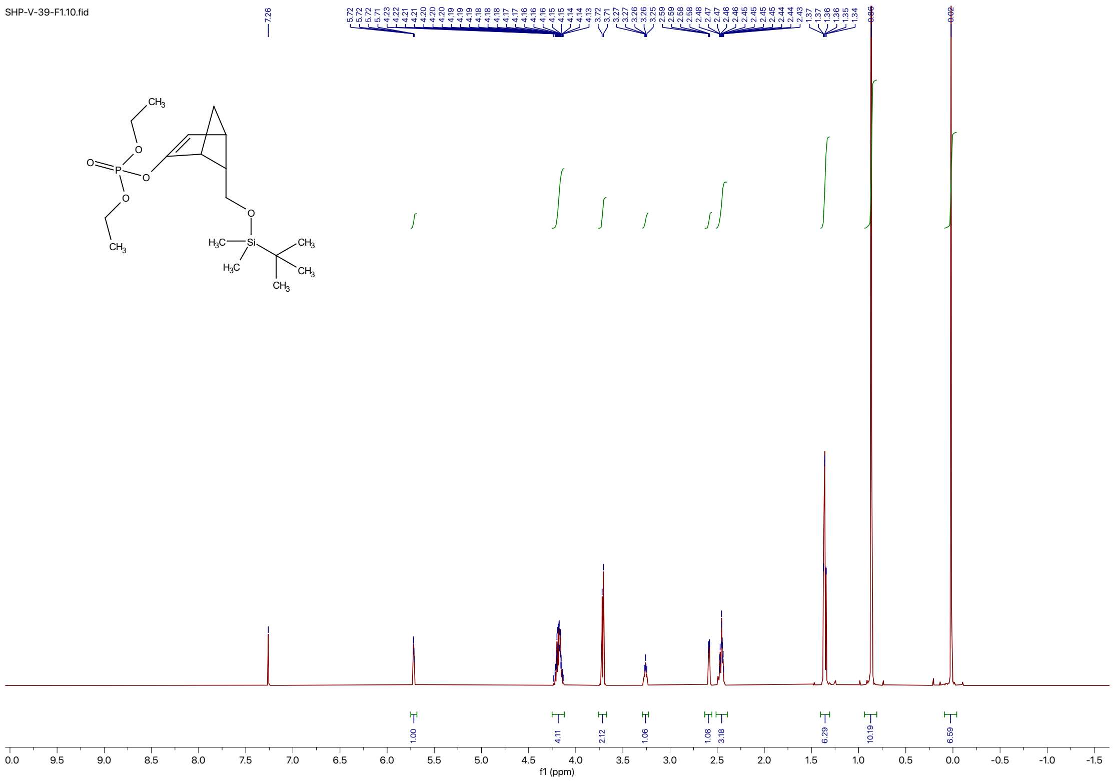

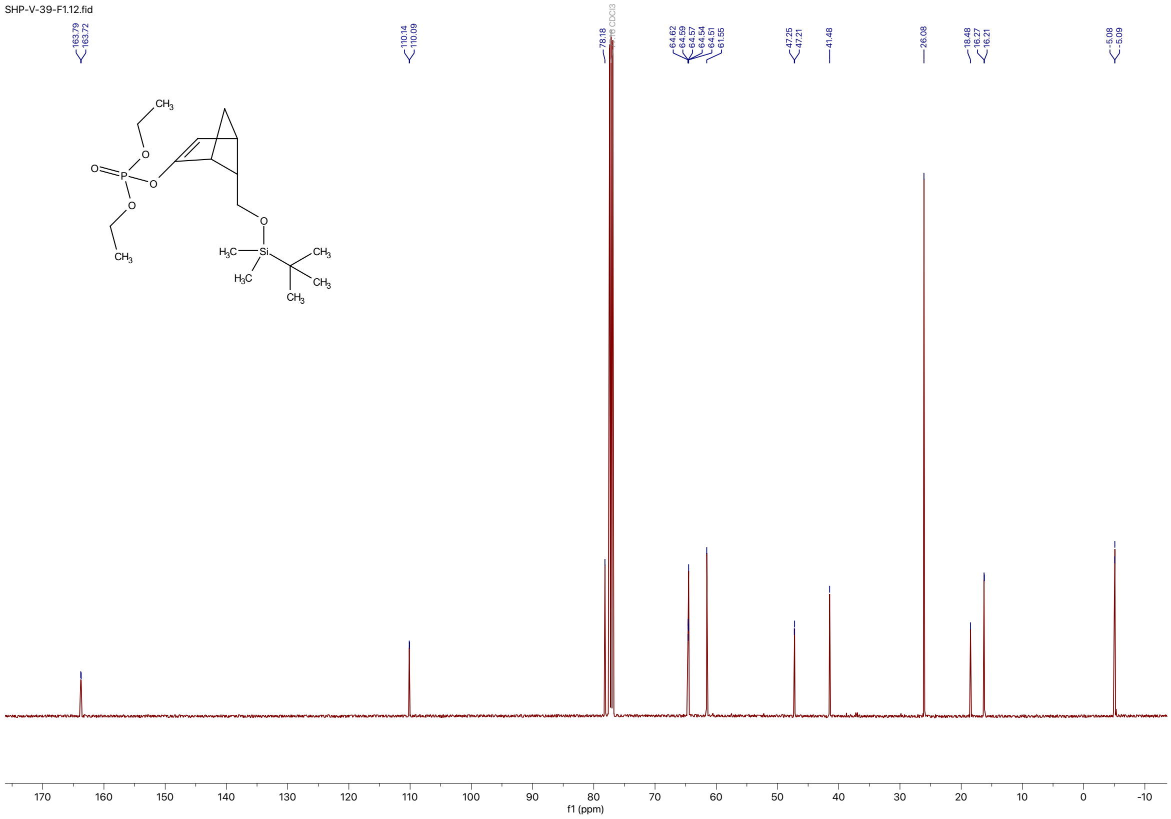

29

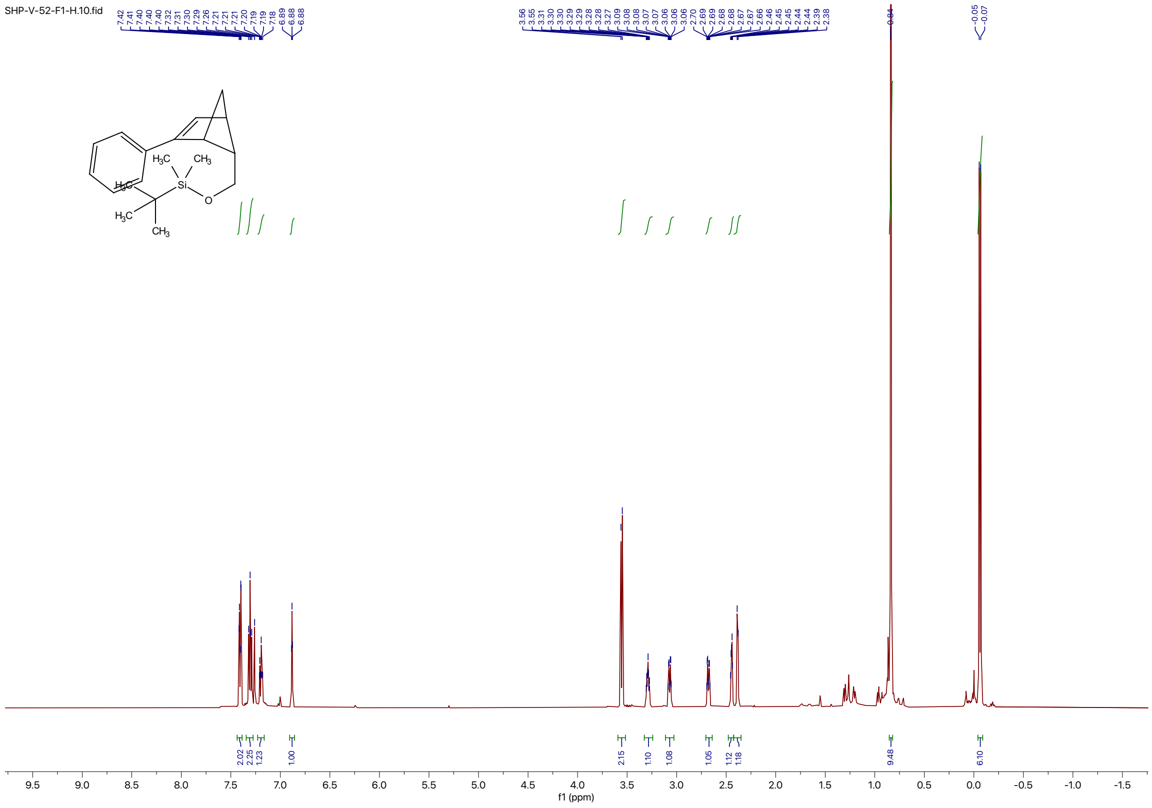

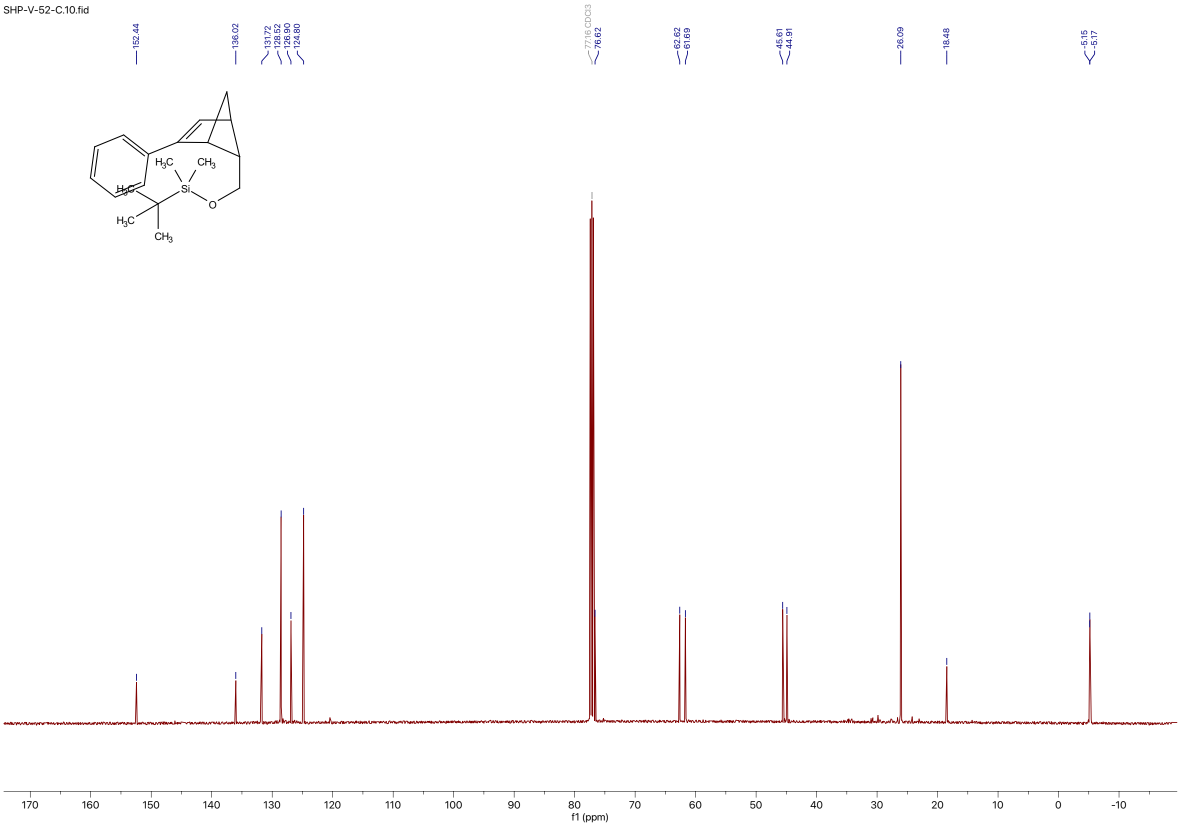

30

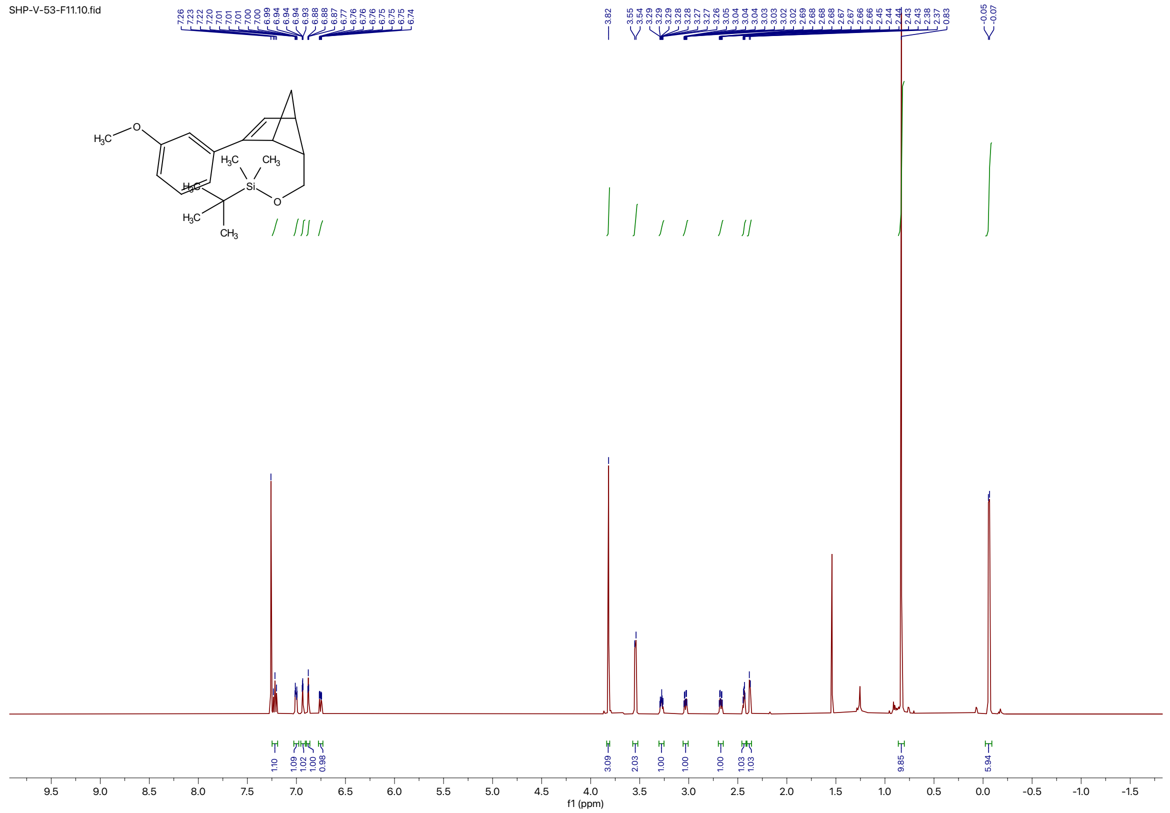

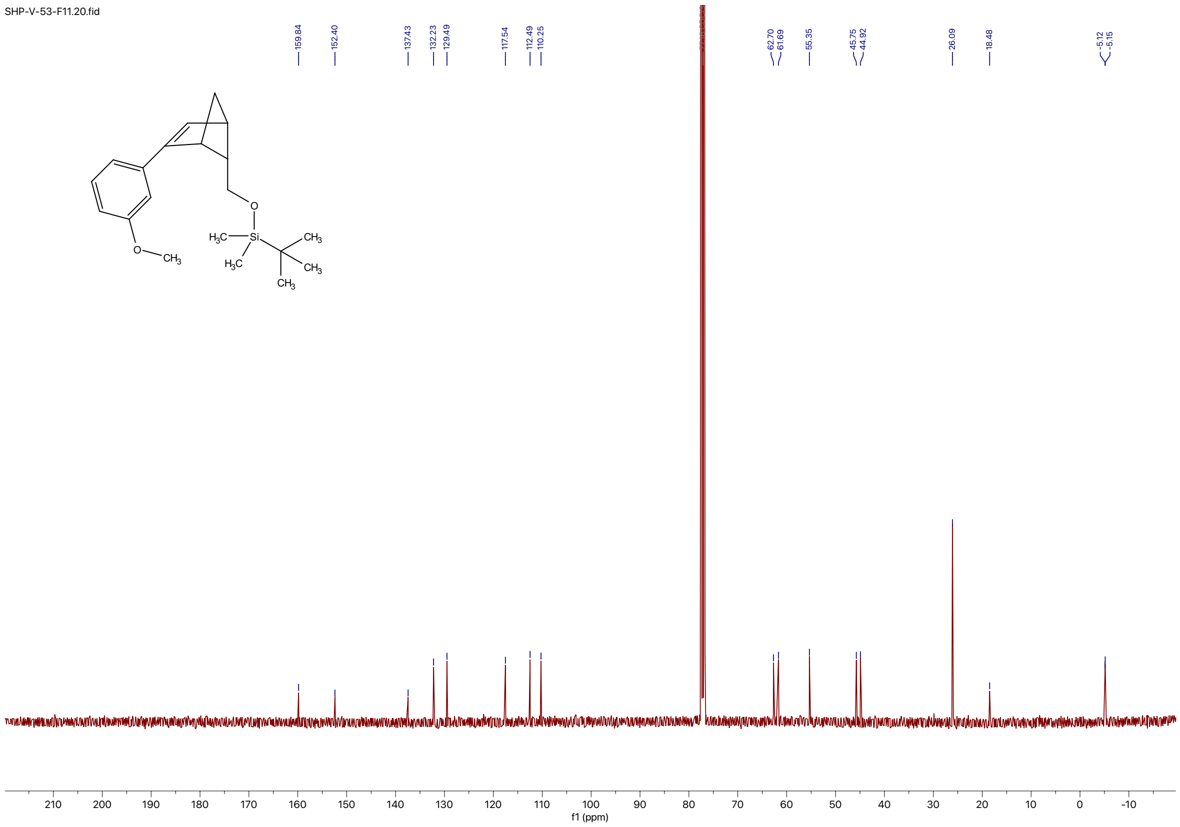

31

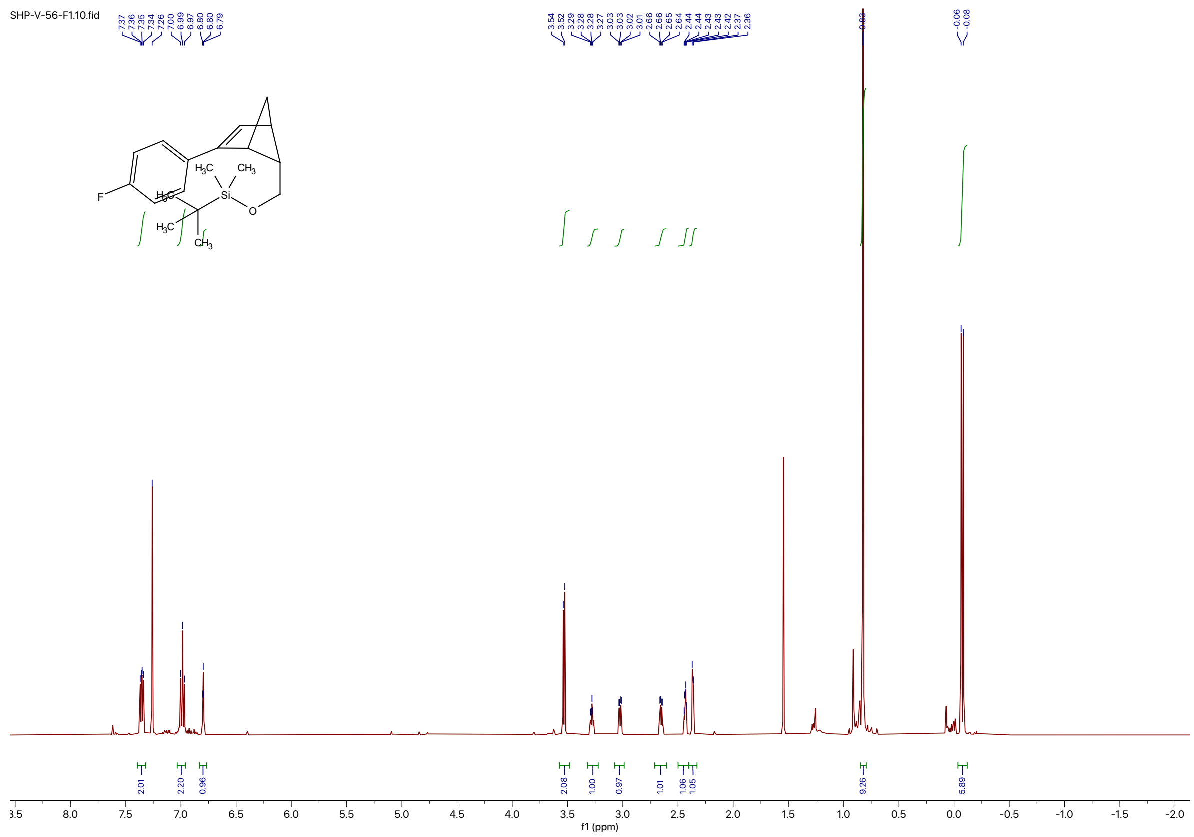
SHP-V-56-F1.10.fid
7.37 7.36 7.35 7.34 7.26 7.00 6.99 6.97 6.80 6.80 6.79
3.54 3.52 3.29 3.28 3.27 3.03 3.03 3.02 3.01 2.66 2.66 2.65 2.64 2.44 2.44 2.43 2.43 2.42 2.37 2.36
0.83
-0.06 -0.08
F
H3C
CH3
Si
O
2.01 2.20 0.96 2.08 1.00 0.97 1.01 1.06 1.05 9.26 5.89
f1 (ppm)

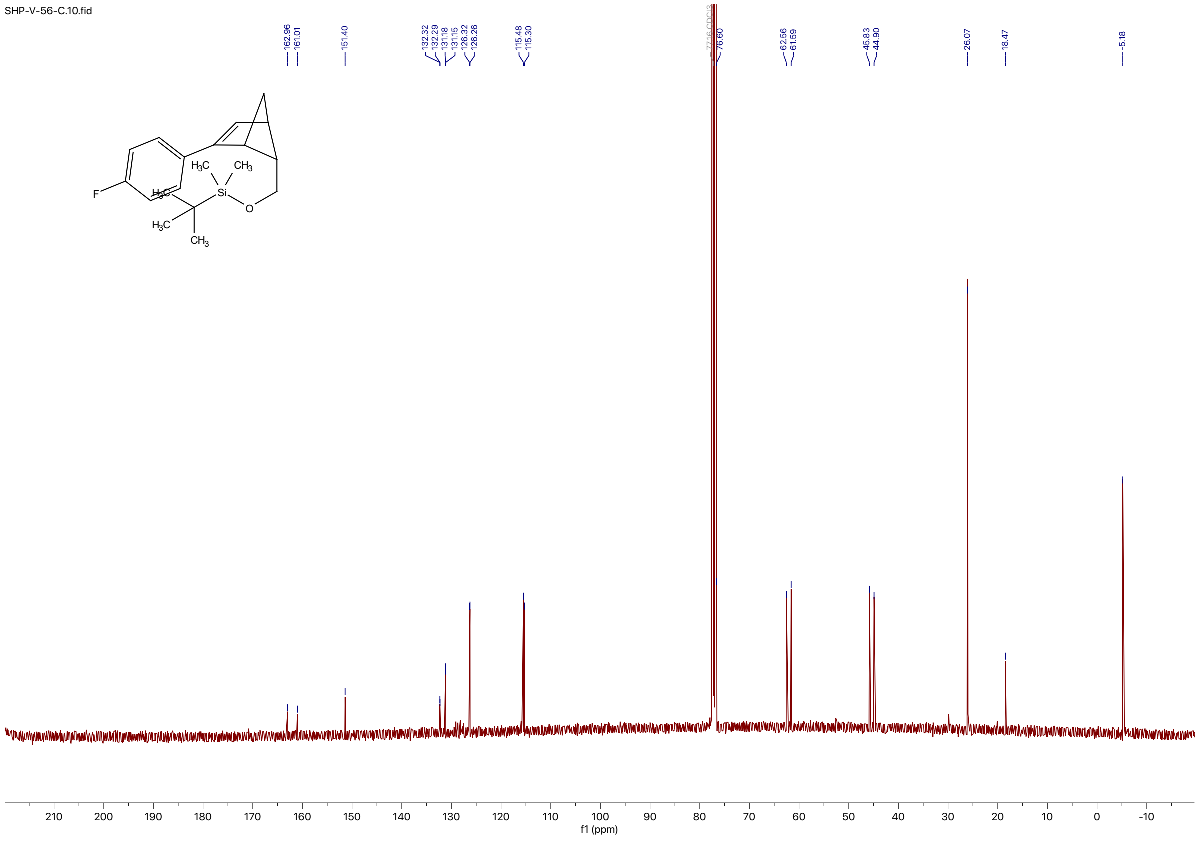
SHP-V-56-C.10.fid
162.96 161.01
151.40
132.32 132.29 131.18 131.15 126.32 126.26
115.48 115.30
77.16 CDCl3 76.60
62.56 61.59
45.83 44.90
26.07
18.47
-5.18
F
H3C
CH3
Si
O
f1 (ppm)

SI-14

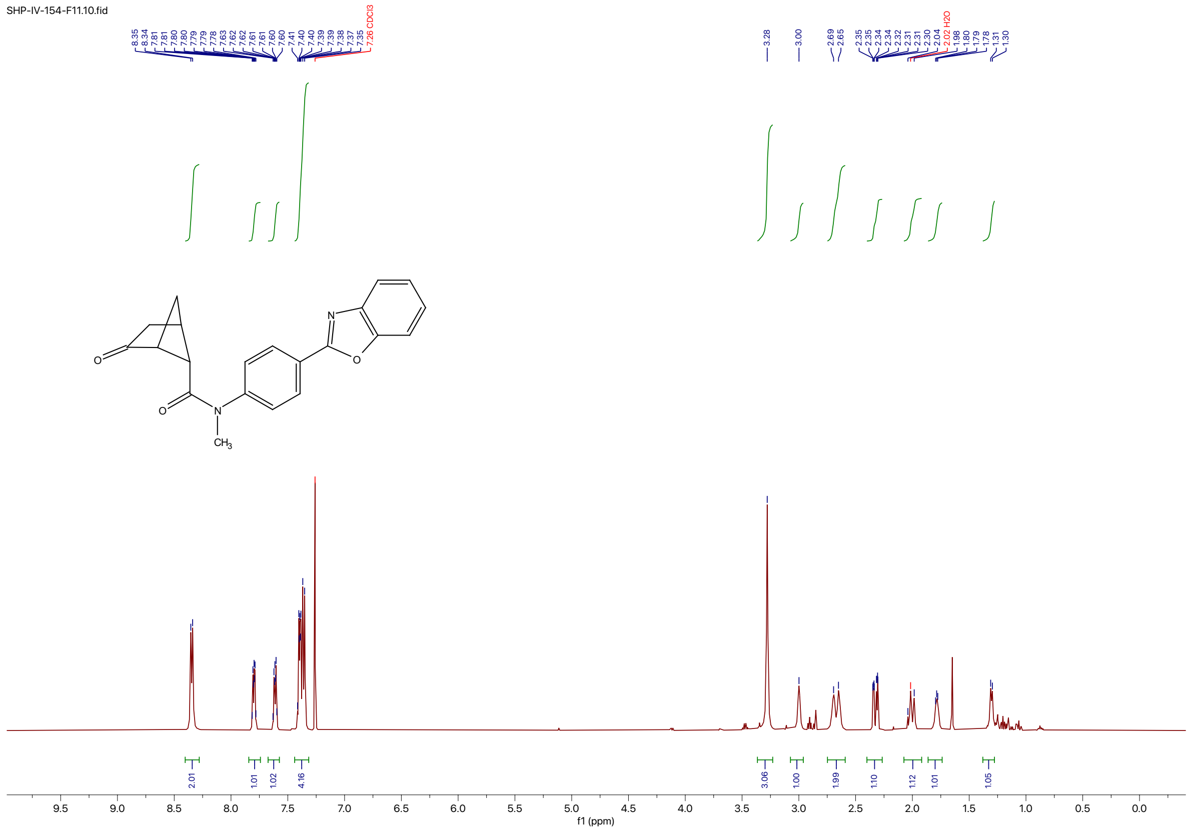

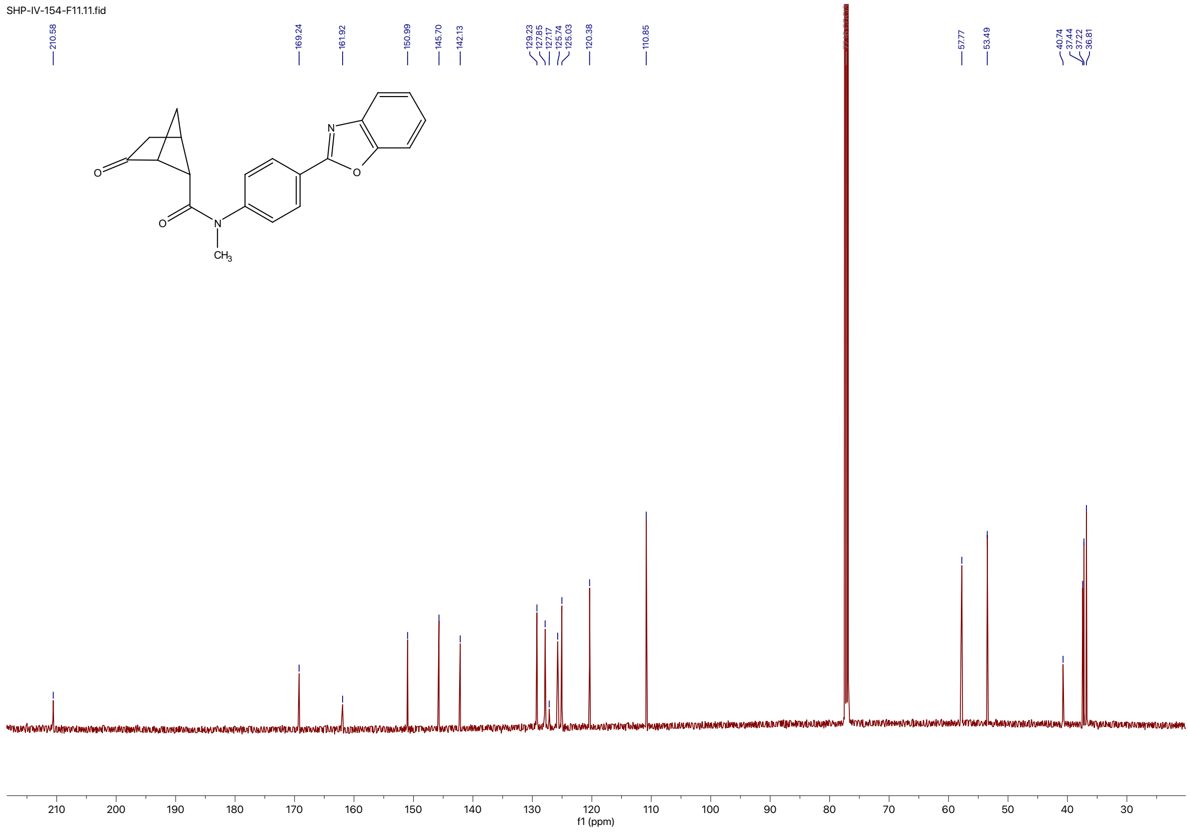

32

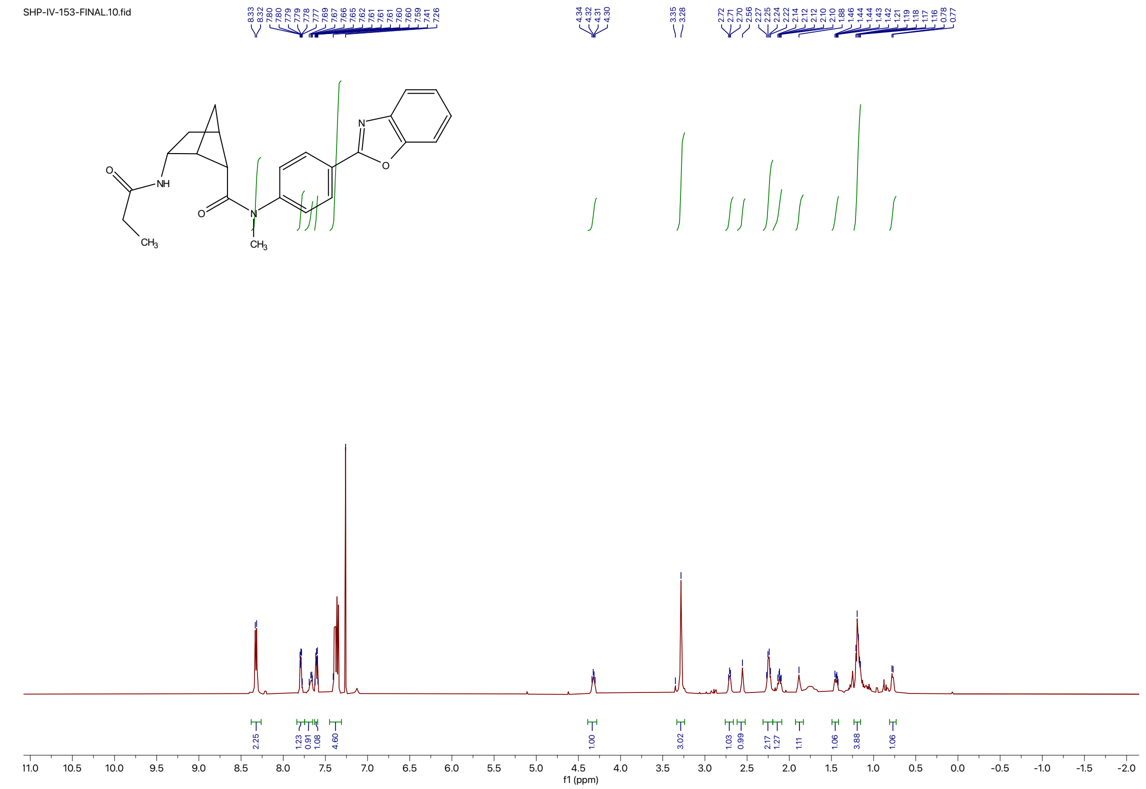

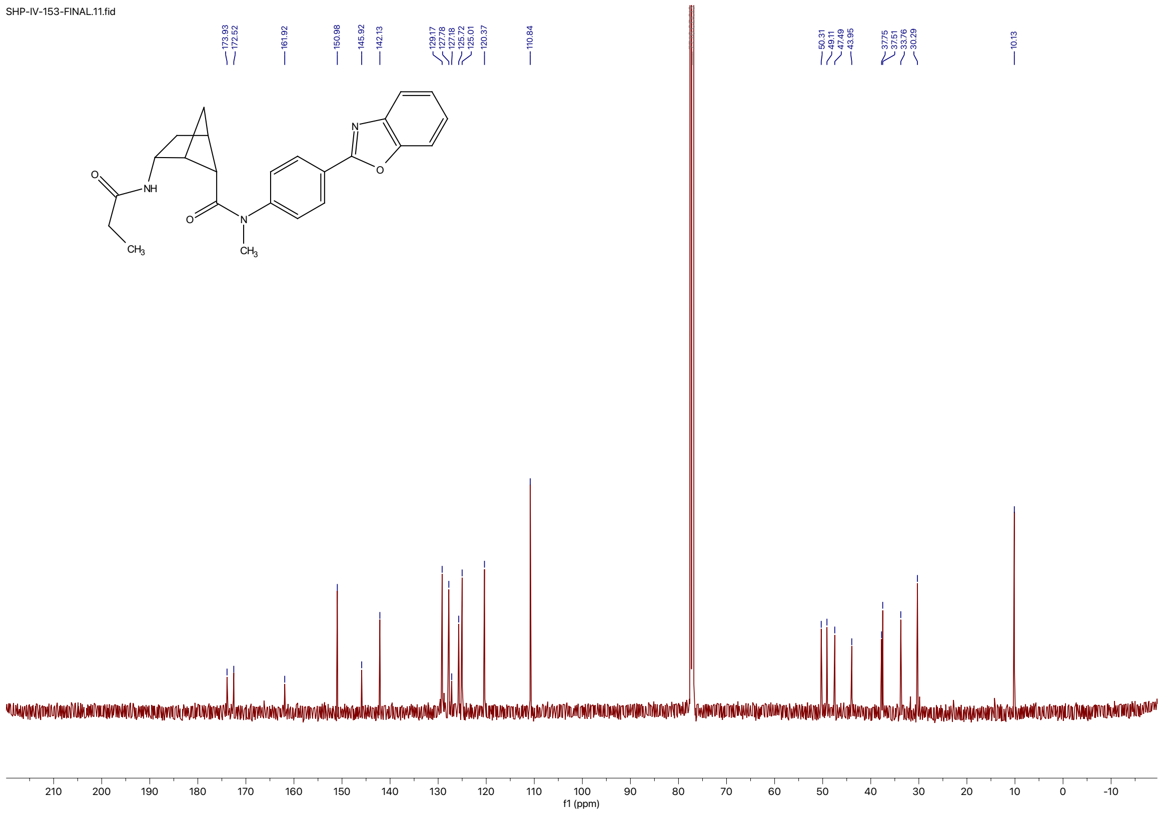

34

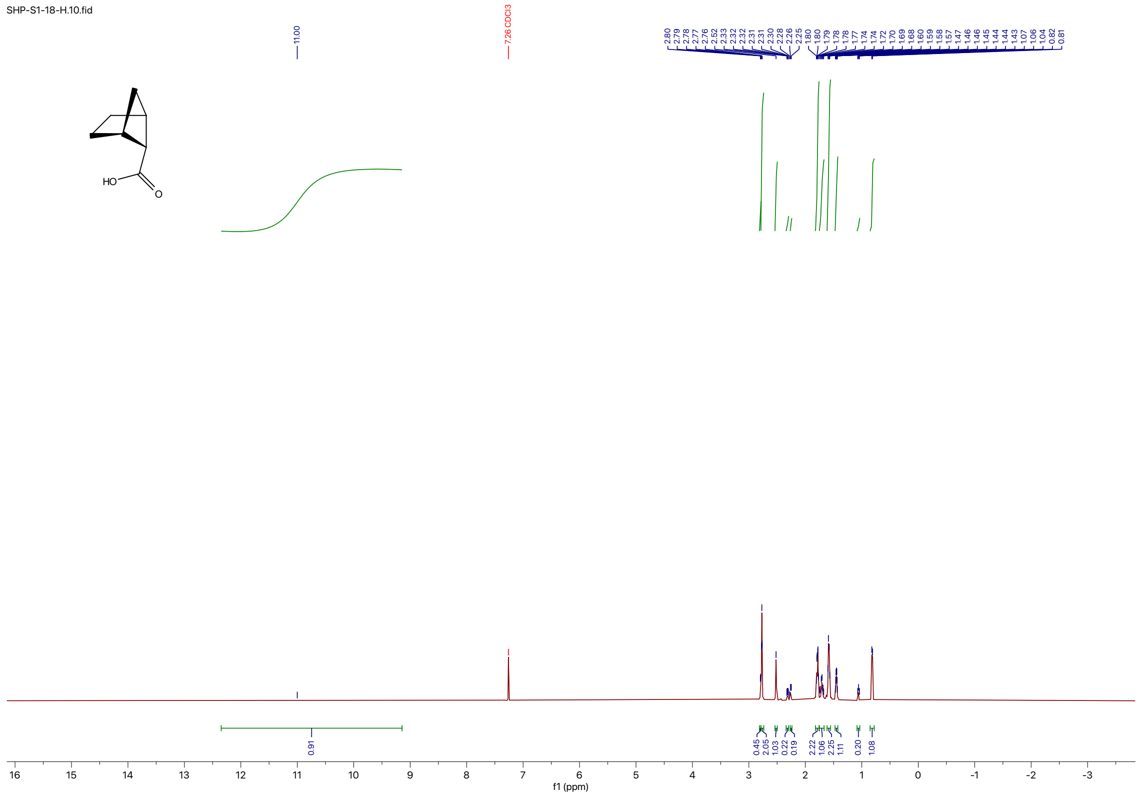
SHP-S1-18-H.10.fid
11.00
7.26 CDCl3
2.80 2.79 2.78 2.77 2.76 2.52 2.33 2.32 2.32 2.31 2.30 2.28 2.26 2.25 1.80 1.80 1.79 1.78 1.78 1.77 1.74 1.74 1.72 1.70 1.69 1.68 1.60 1.59 1.58 1.57 1.47 1.46 1.46 1.45 1.44 1.44 1.43 1.07 1.06 1.04 0.82 0.81
HO
O
0.91
0.45 2.05 1.03 0.22 0.19 2.22 1.06 2.25 1.11 0.20 1.08
16 15 14 13 12 11 10 9 8 7 6 5 4 3 2 1 0 -1 -2 -3
f1 (ppm)

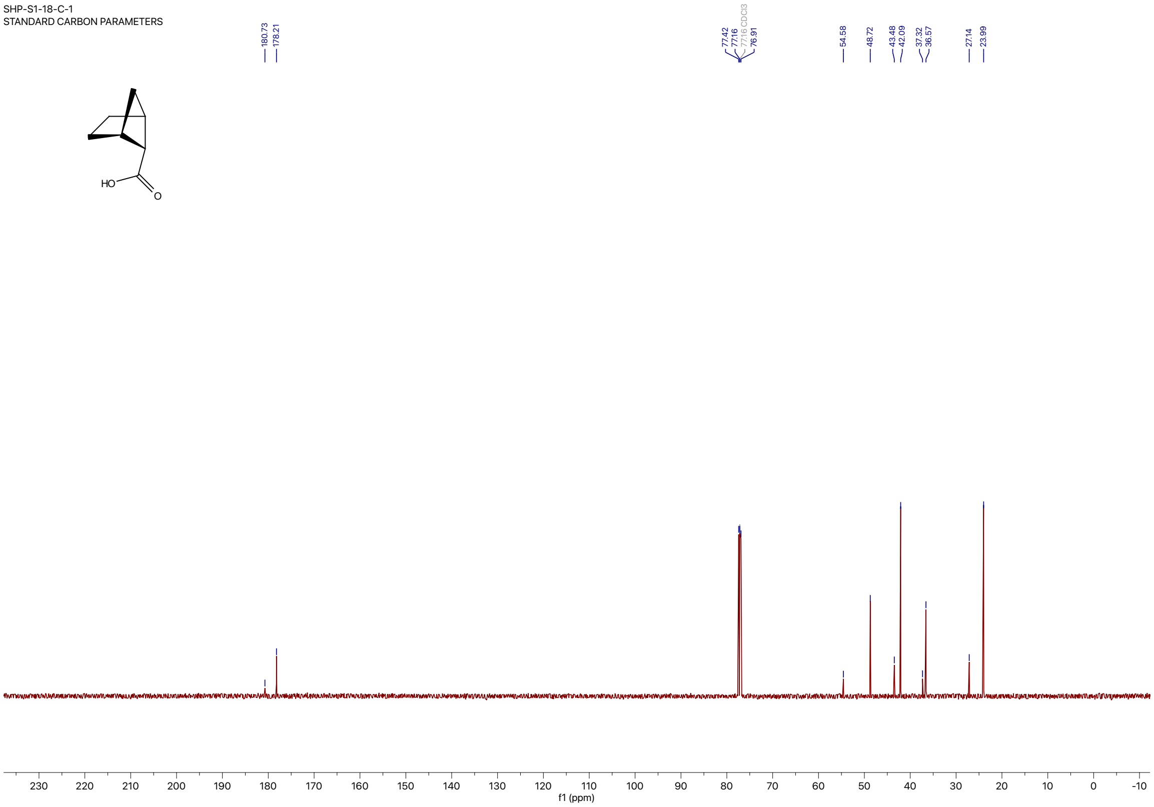
SHP-S1-18-C-1
STANDARD CARBON PARAMETERS
180.73
178.21
77.42 77.16 77.16 CDCl3 76.91
54.58
48.72
43.48 42.09
37.32 36.57
27.14
23.99
HO
O
230 220 210 200 190 180 170 160 150 140 130 120 110 100 90 80 70 60 50 40 30 20 10 0 -10
f1 (ppm)

35

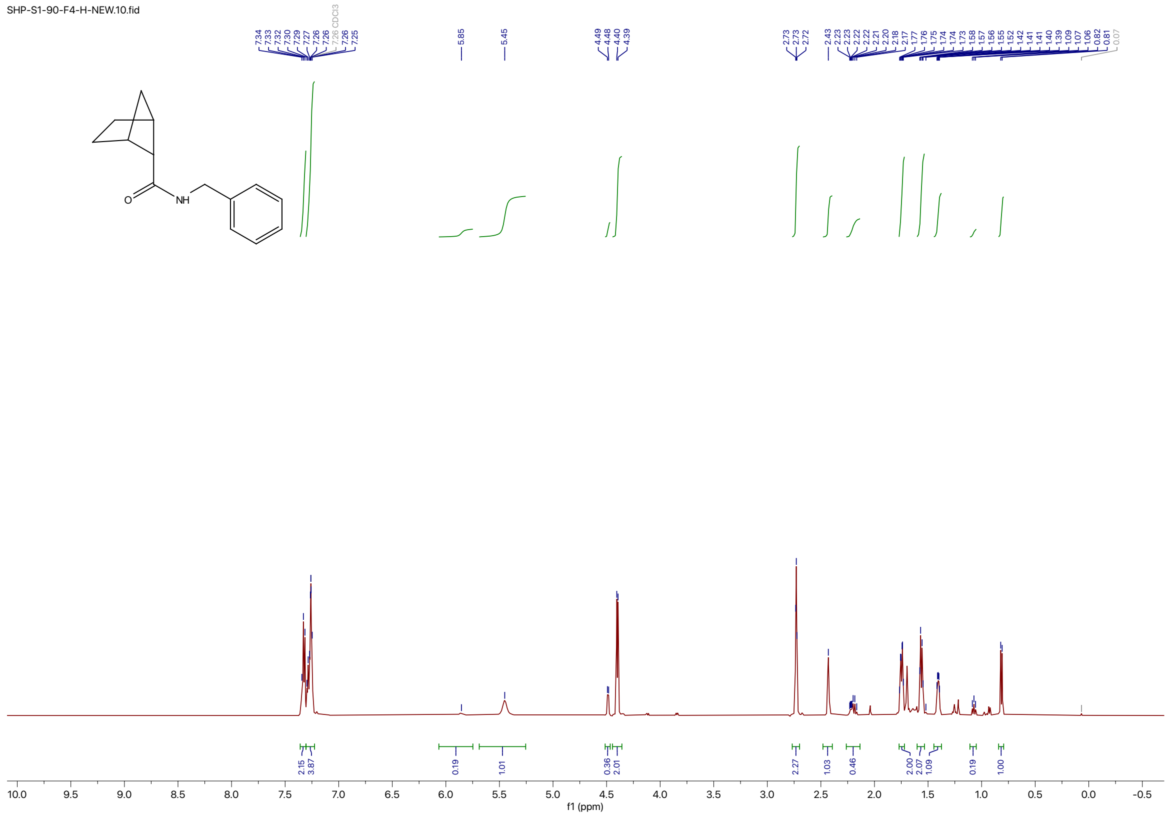
SHP-S1-90-F4-H-NEW.10.fid
O
NH
f1 (ppm)

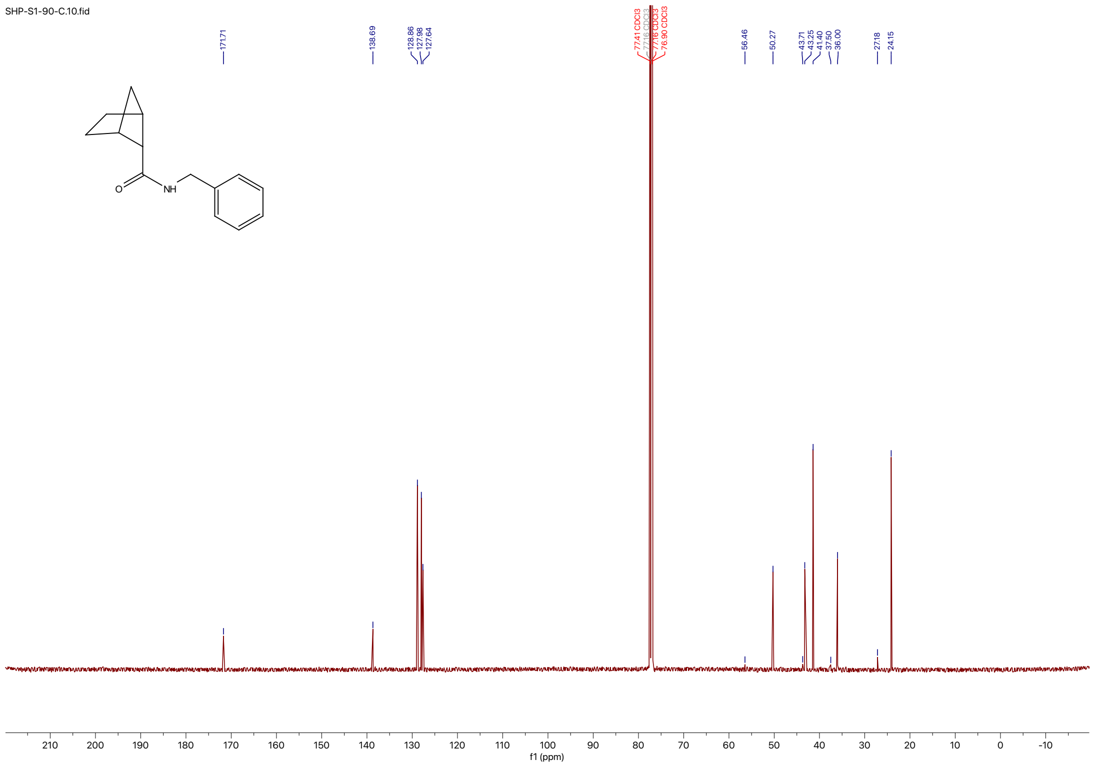
SHP-S1-90-C.10.fid
171.71
138.69
128.86
127.86
127.64
77.41 CDCl3
77.16 CDCl3
76.90 CDCl3
56.46
50.27
43.71
43.25
41.40
37.50
36.00
27.18
24.15
O
NH
f1 (ppm)

44

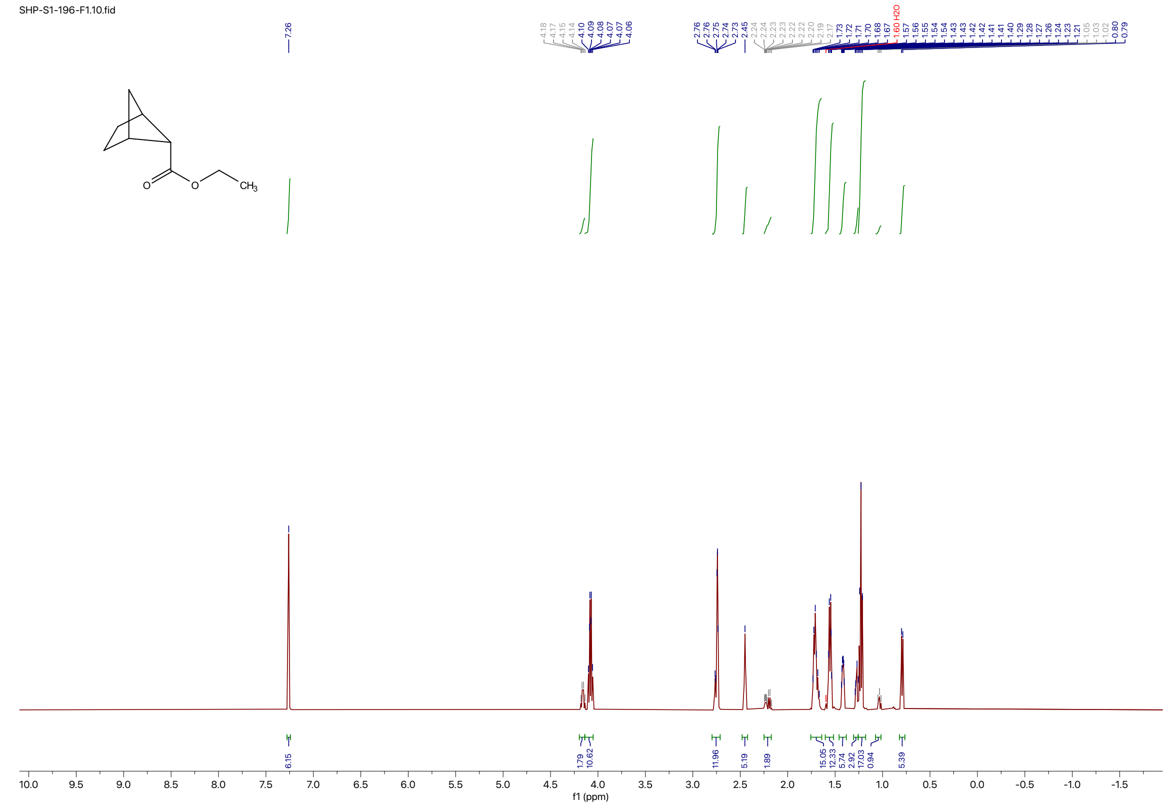
SHP-S1-196-F1.10.fid
CH3
O
O
f1 (ppm)

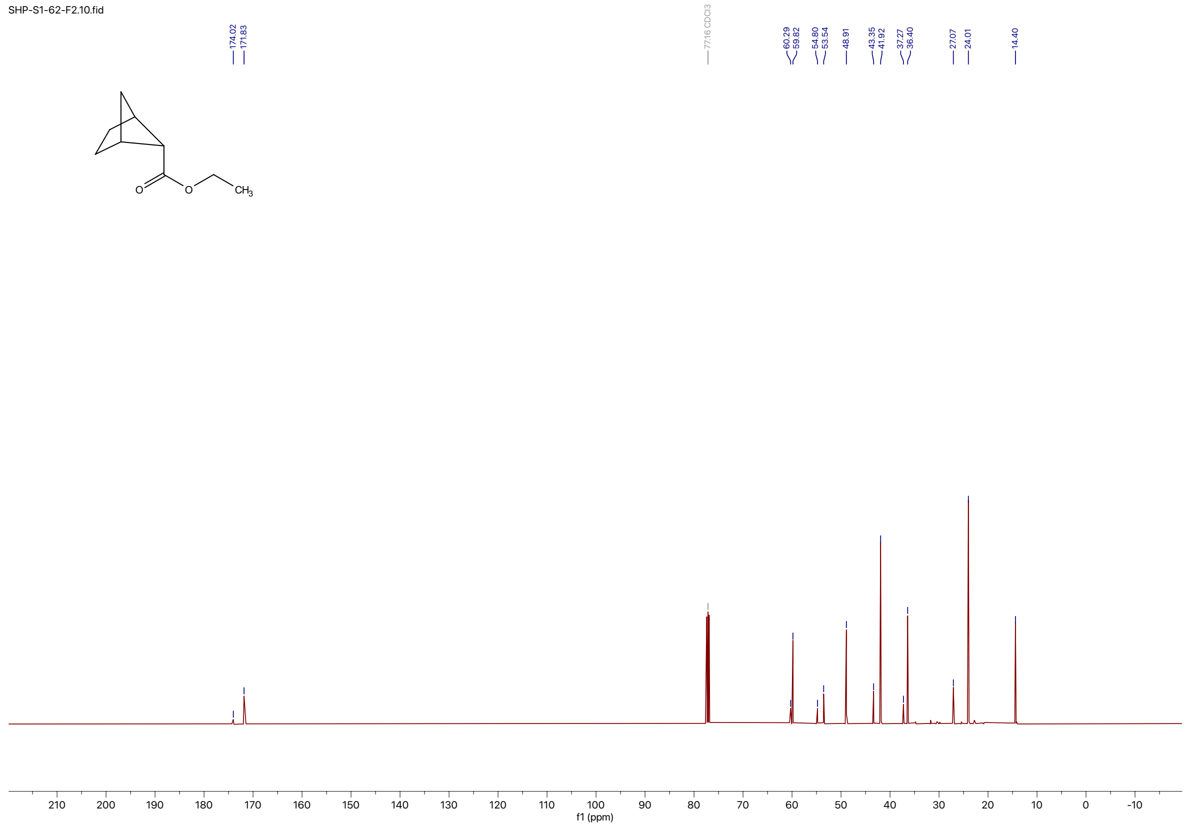
SHP-S1-62-F2.10.fid
174.02
171.83
77.16 CDCl3
60.29
59.82
54.80
53.54
48.91
43.35
41.92
37.27
36.40
27.07
24.01
14.40
CH3
O
O
f1 (ppm)

37

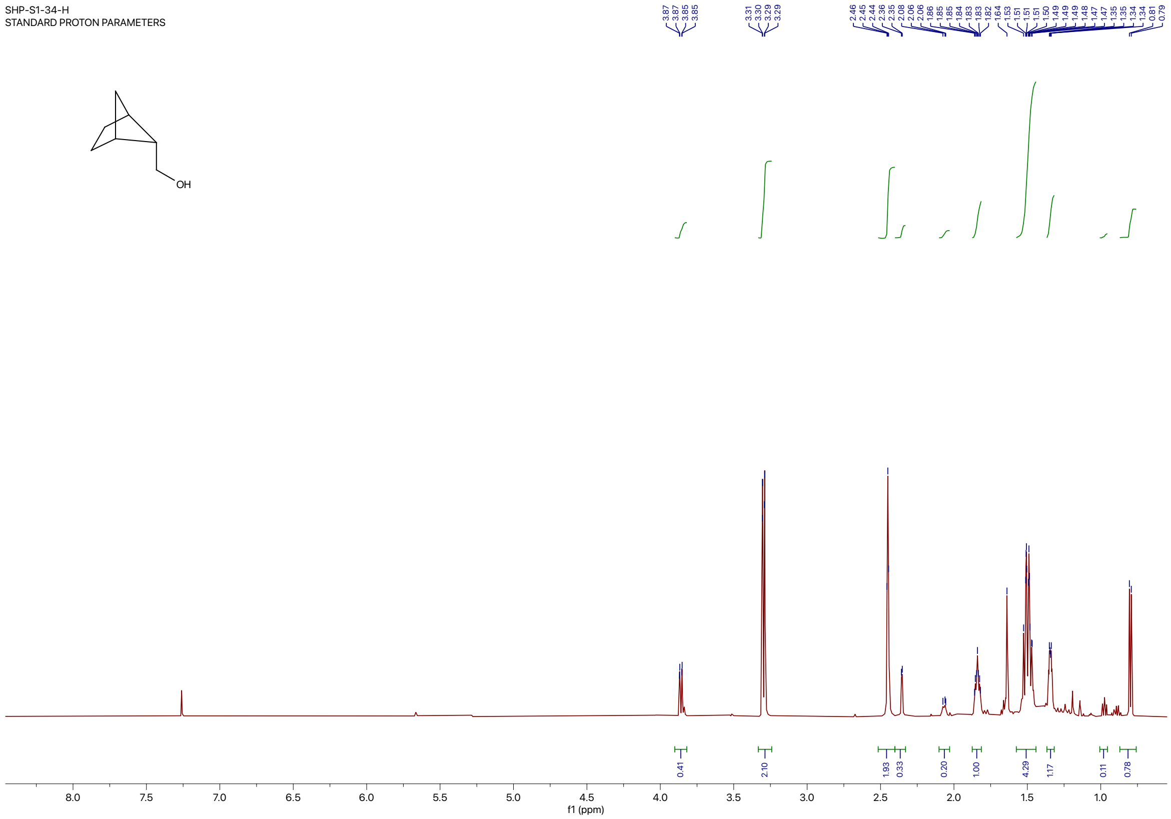

SHP-S1-34-H
STANDARD PROTON PARAMETERS
OH
f1 (ppm)

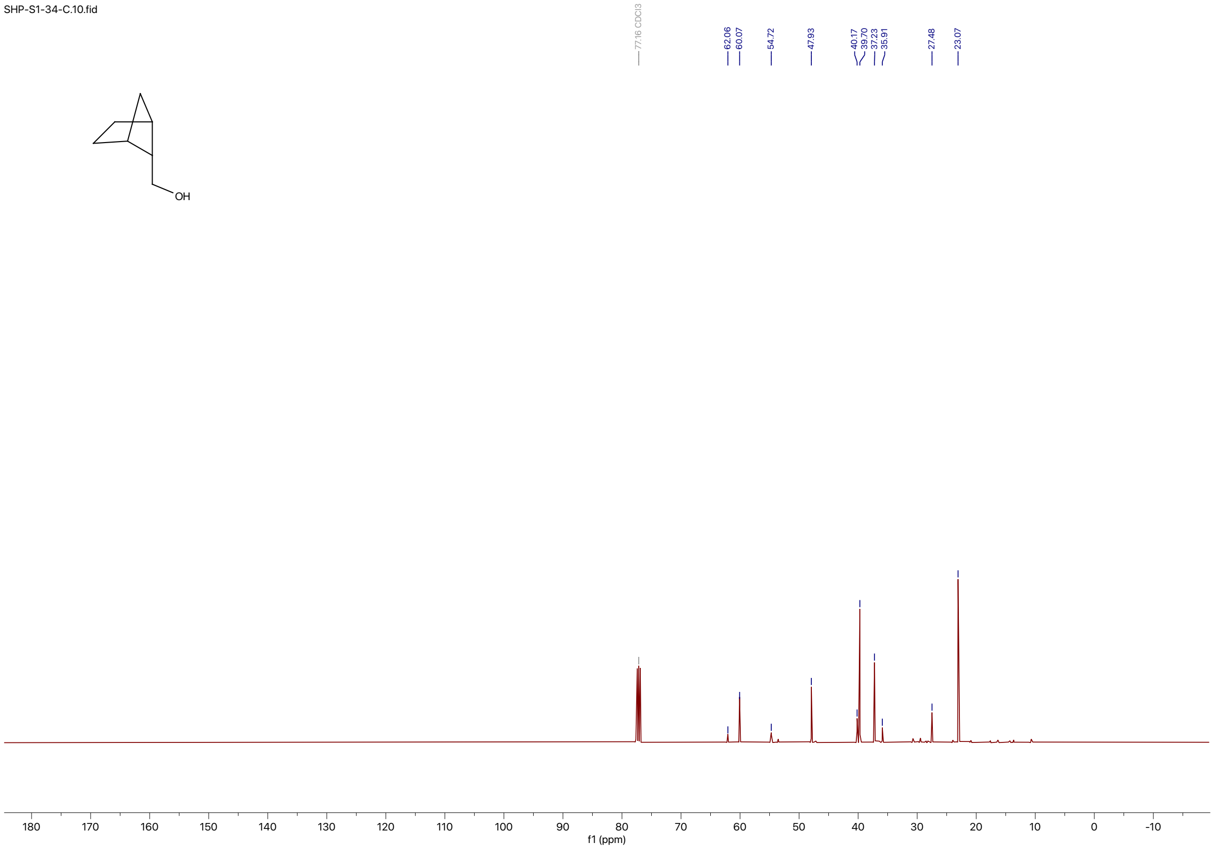

SHP-S1-34-C.10.fid
77.16 CDCl3
62.06
60.07
54.72
47.93
40.17
39.70
37.23
35.91
27.48
23.07
OH
f1 (ppm)

36

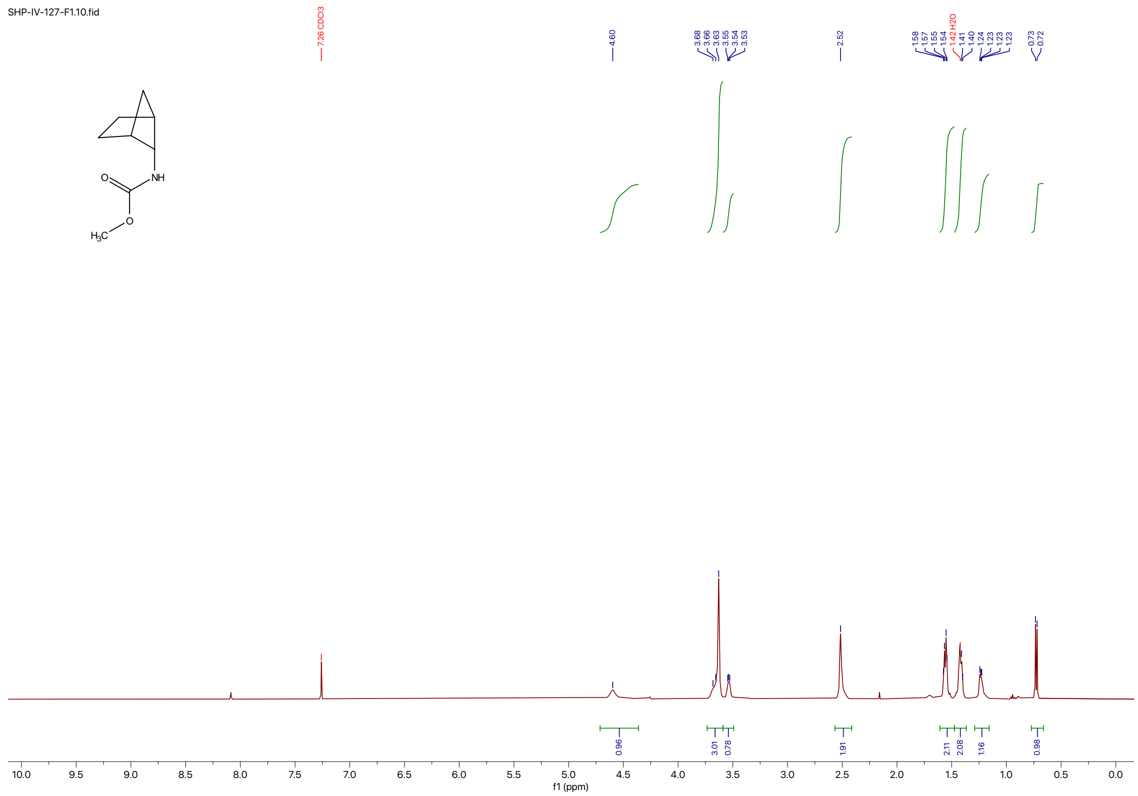
SHP-IV-127-F1.10.fid
7.26 CDCl3
4.60
3.68
3.66
3.63
3.55
3.54
3.53
2.52
1.58
1.57
1.55
1.54
1.42 H2O
1.41
1.40
1.24
1.23
1.23
1.23
0.73
0.72
NH
O
O
H3C
0.96
3.01
0.78
1.91
2.11
2.08
1.16
0.98
f1 (ppm)

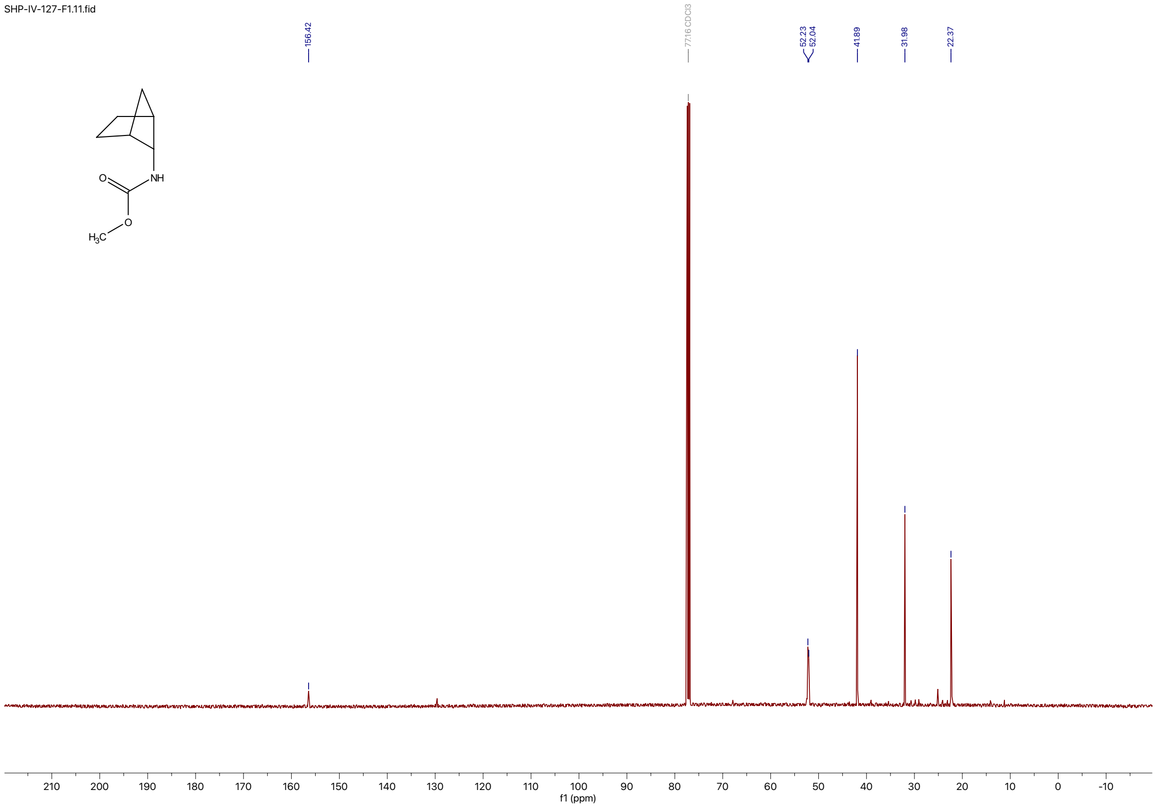
SHP-IV-127-F1.11.fid
156.42
77.16 CDCl3
52.23
52.04
41.89
31.98
22.37
NH
O
O
H3C
f1 (ppm)

38

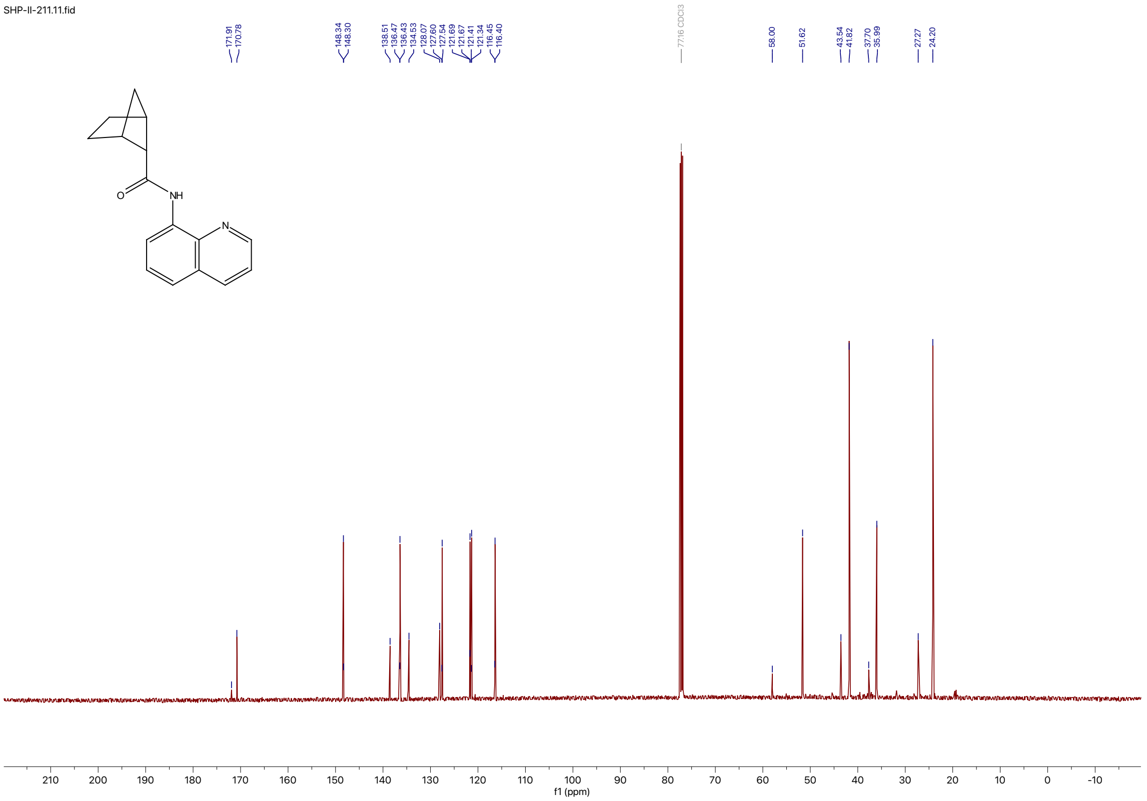
SHP-II-211.11.fid
171.91
170.78
148.34
148.30
138.51
136.47
136.43
134.53
128.07
127.60
127.54
121.69
121.67
121.41
121.34
116.45
116.40
77.16 CDCl3
58.00
51.62
43.54
41.82
37.70
35.99
27.27
24.20
O
NH
N
f1 (ppm)

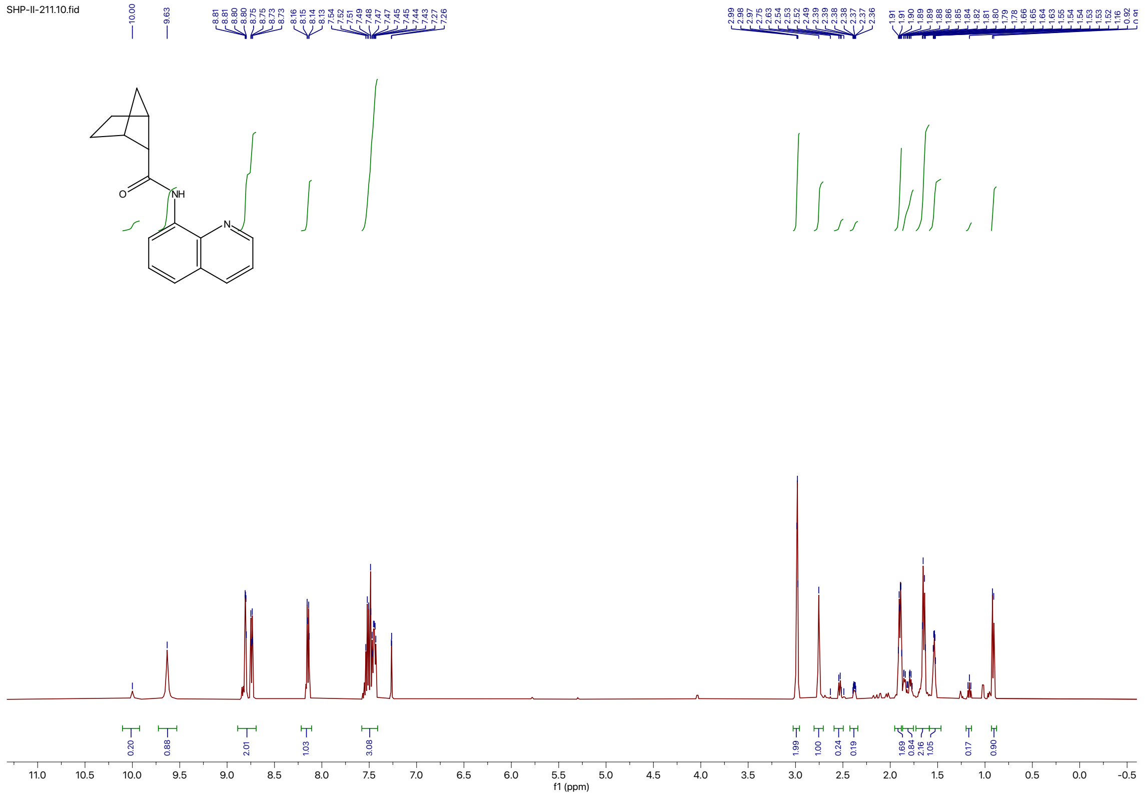
SHP-II-211.10.fid
10.00
9.63
O
NH
N
0.20
0.88
2.01
1.03
3.08
1.99
1.00
0.24
0.19
1.69
0.84
2.16
1.05
0.17
0.90
f1 (ppm)

SI-15

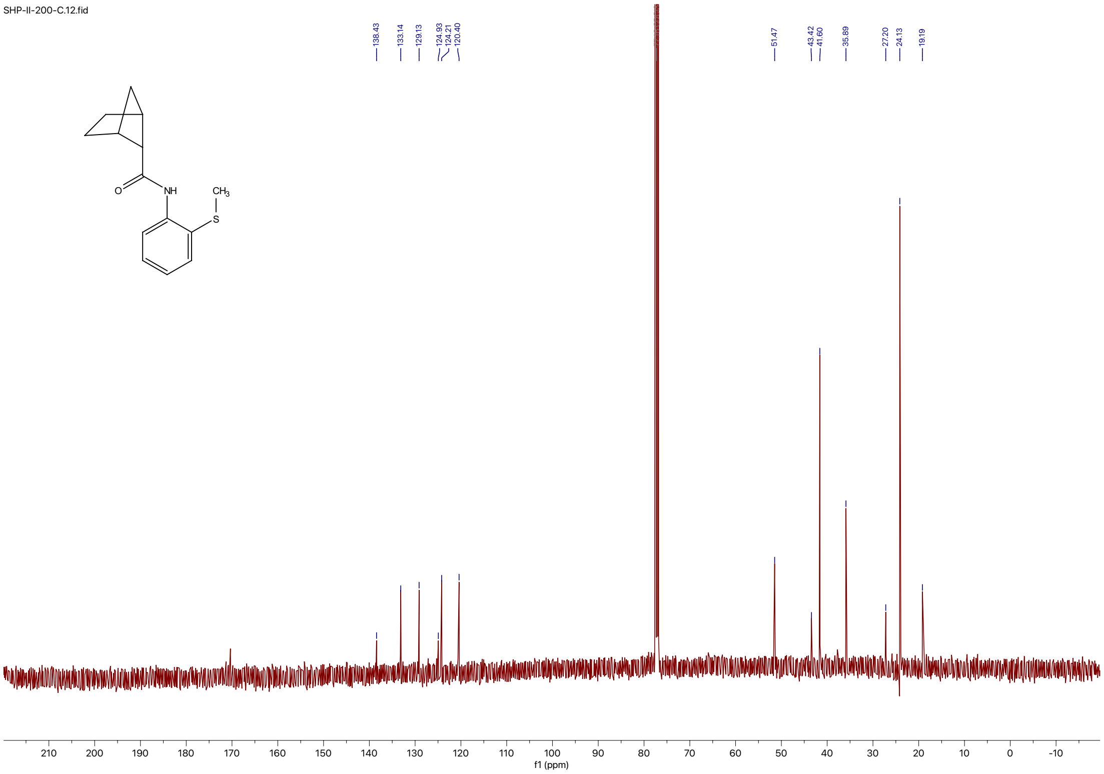

SHP-II-200-C.12.fid
138.43
133.14
129.13
124.93
124.21
120.40
51.47
43.42
41.60
35.89
27.20
24.13
19.19
O
NH
CH3
S
210 200 190 180 170 160 150 140 130 120 110 100 90 80 70 60 50 40 30 20 10 0 -10
f1 (ppm)

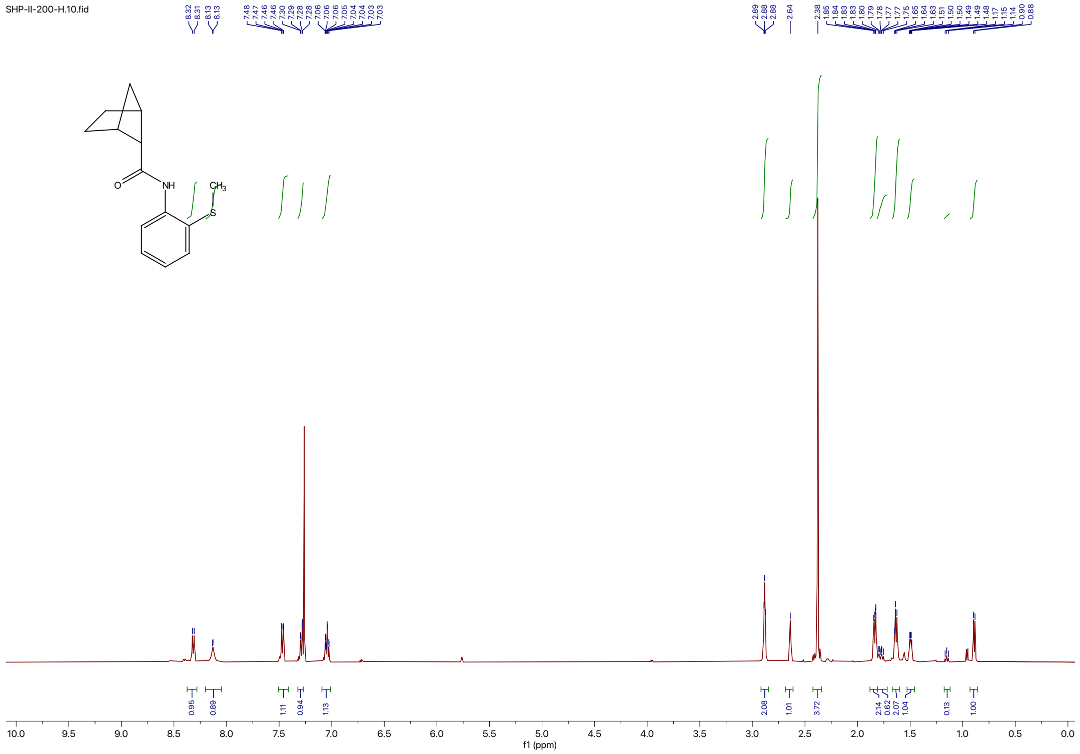

SHP-II-200-H.10.fid
8.32 8.31 8.13 8.13
7.48 7.47 7.46 7.46 7.30 7.29 7.28 7.28 7.06 7.06 7.05 7.04 7.04 7.03 7.03
2.89 2.88 2.88 2.64 2.38 1.85 1.84 1.83 1.83 1.80 1.79 1.78 1.77 1.77 1.75 1.65 1.64 1.63 1.51 1.50 1.50 1.49 1.48 1.17 1.15 1.14 0.90 0.88
O
NH
CH3
S
0.95 0.89 1.11 0.94 1.13 2.08 1.01 3.72 2.14 0.62 2.07 1.04 0.13 1.00
10.0 9.5 9.0 8.5 8.0 7.5 7.0 6.5 6.0 5.5 5.0 4.5 4.0 3.5 3.0 2.5 2.0 1.5 1.0 0.5 0.0
f1 (ppm)

39a

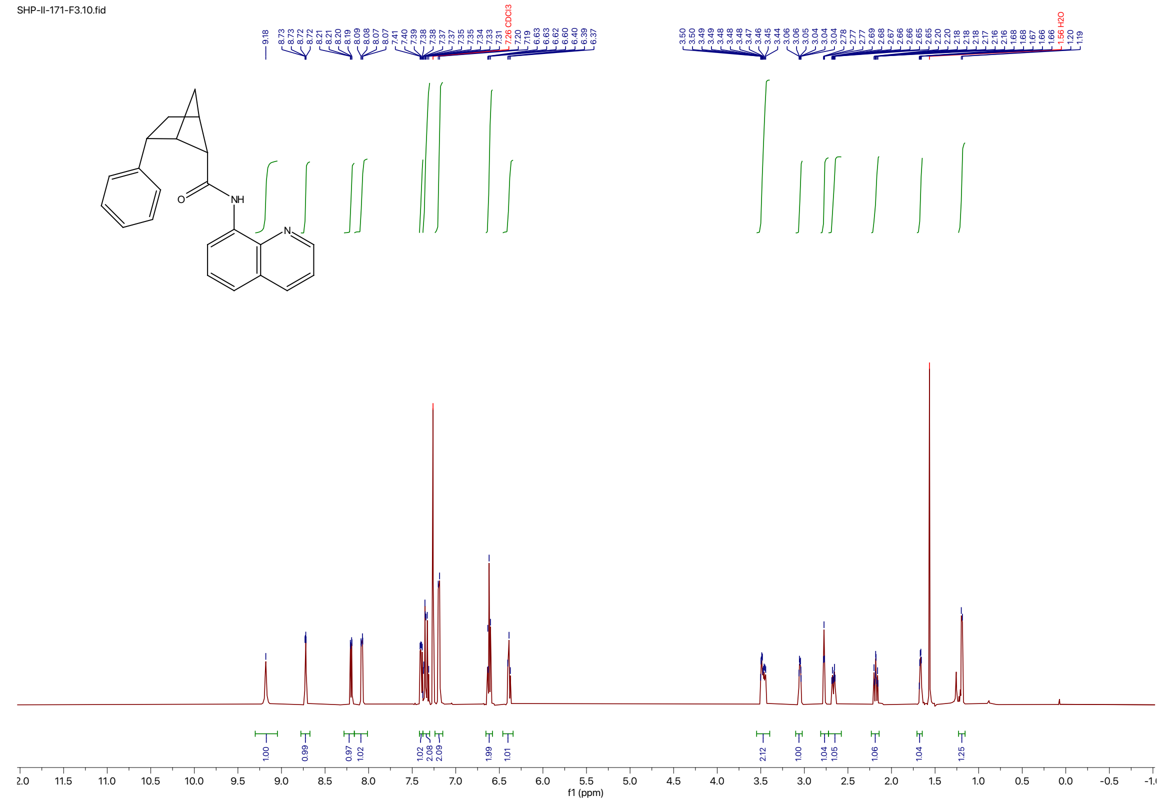
SHP-II-171-F3.10.fid
f1 (ppm)

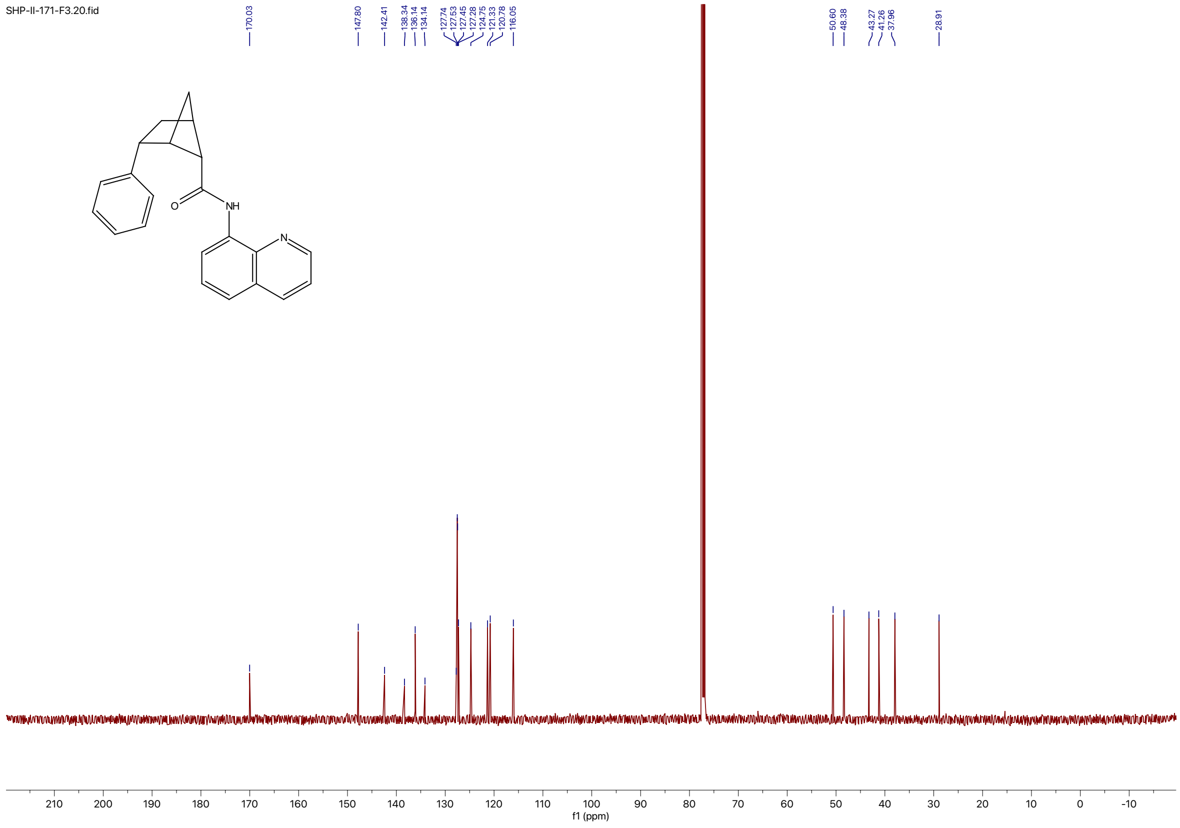
SHP-II-171-F3.20.fid
170.03
147.80
142.41
138.34
136.14
134.14
127.74
127.53
127.45
127.28
124.75
121.33
120.78
116.05
50.60
48.38
43.27
41.26
37.96
28.91
f1 (ppm)

COSY of 39a

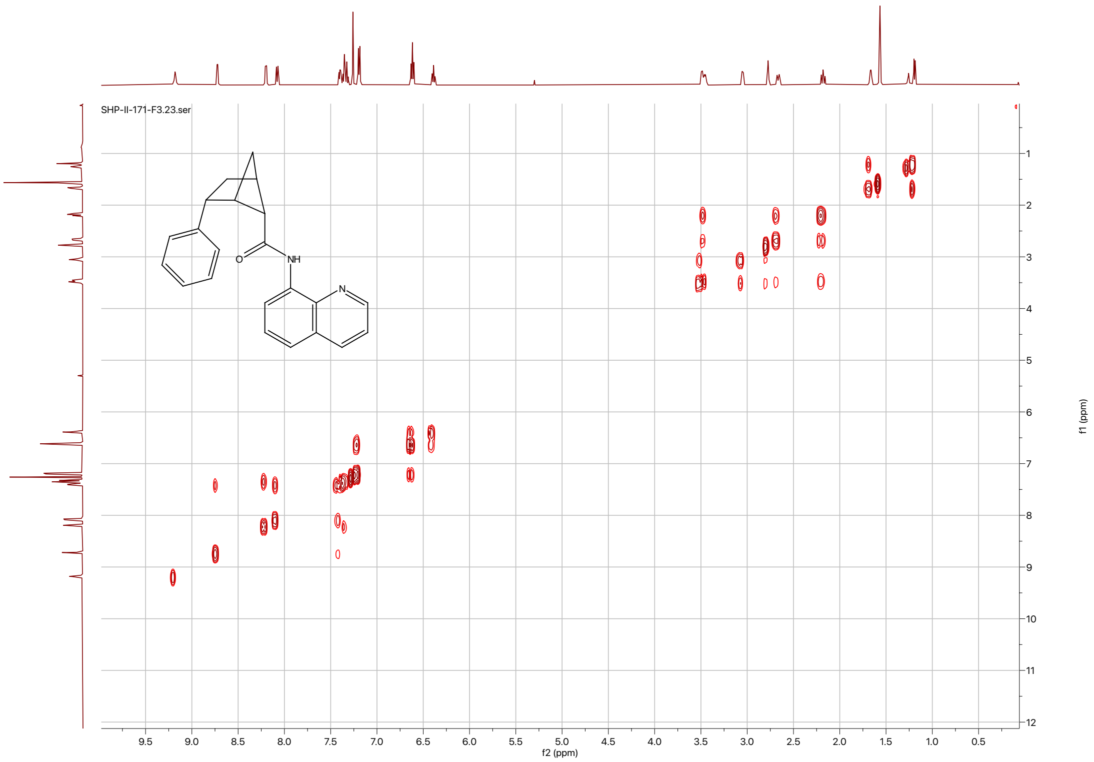

NOSEY of 39a

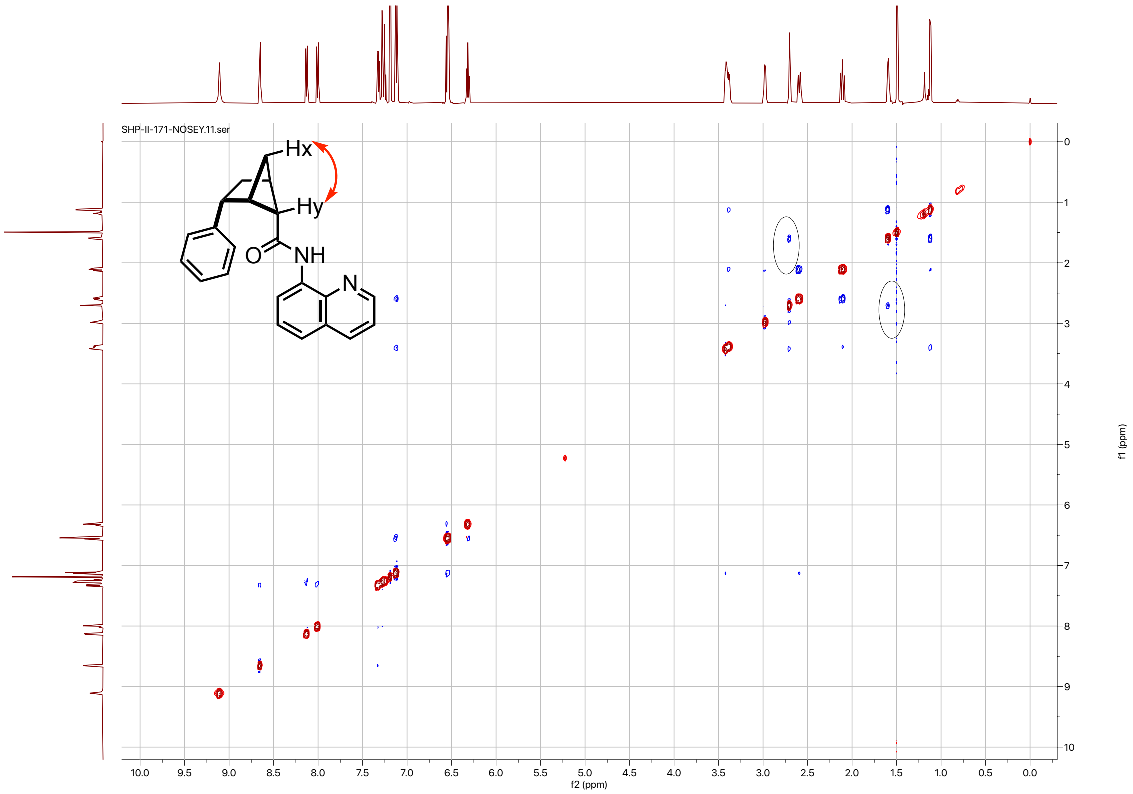

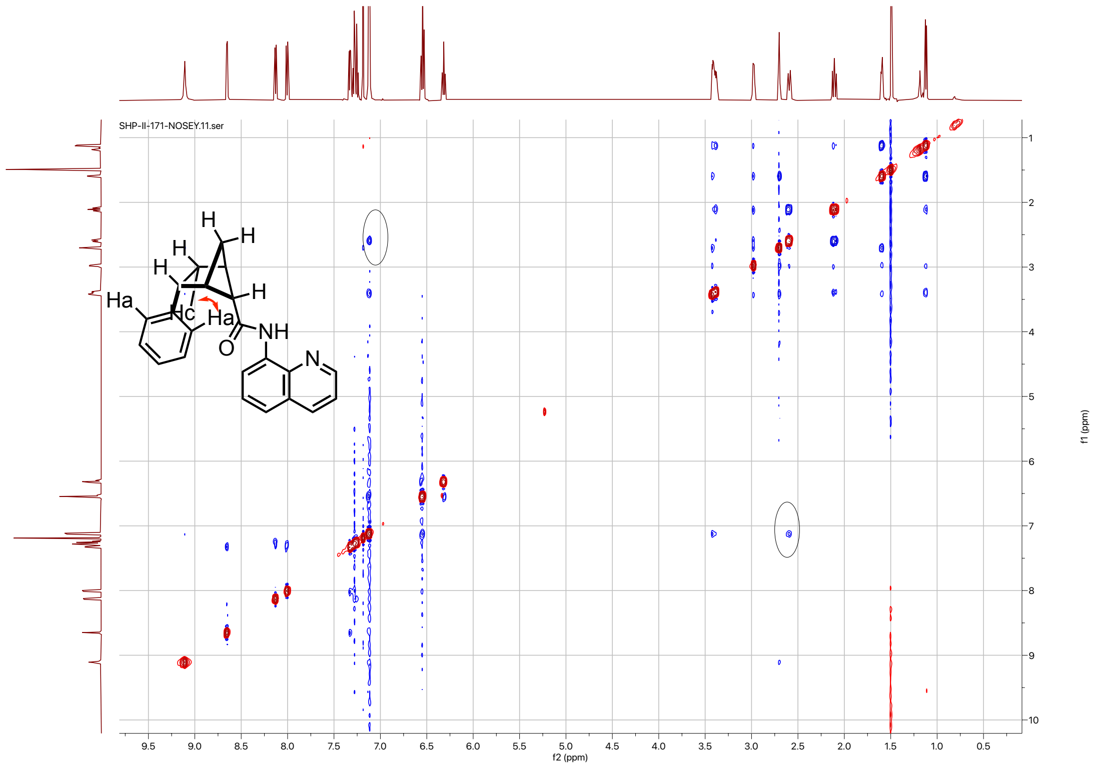
SHP-II-171-NOSEY.11.ser
Ha
Hc
Ha
NH
O
N
f2 (ppm)
f1 (ppm)

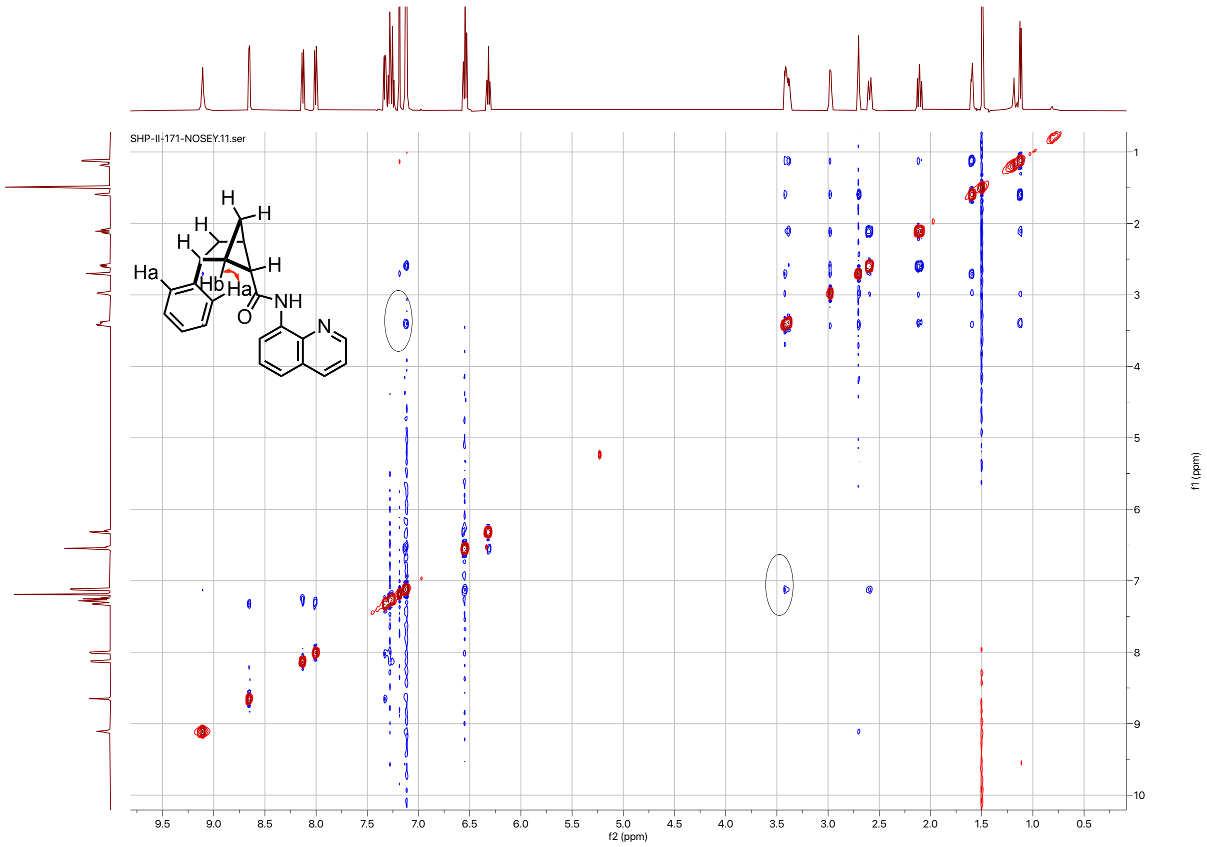
SHP-II-171-NOSEY.11.ser
Ha
Hb
Ha
NH
O
N
f2 (ppm)
f1 (ppm)

39b

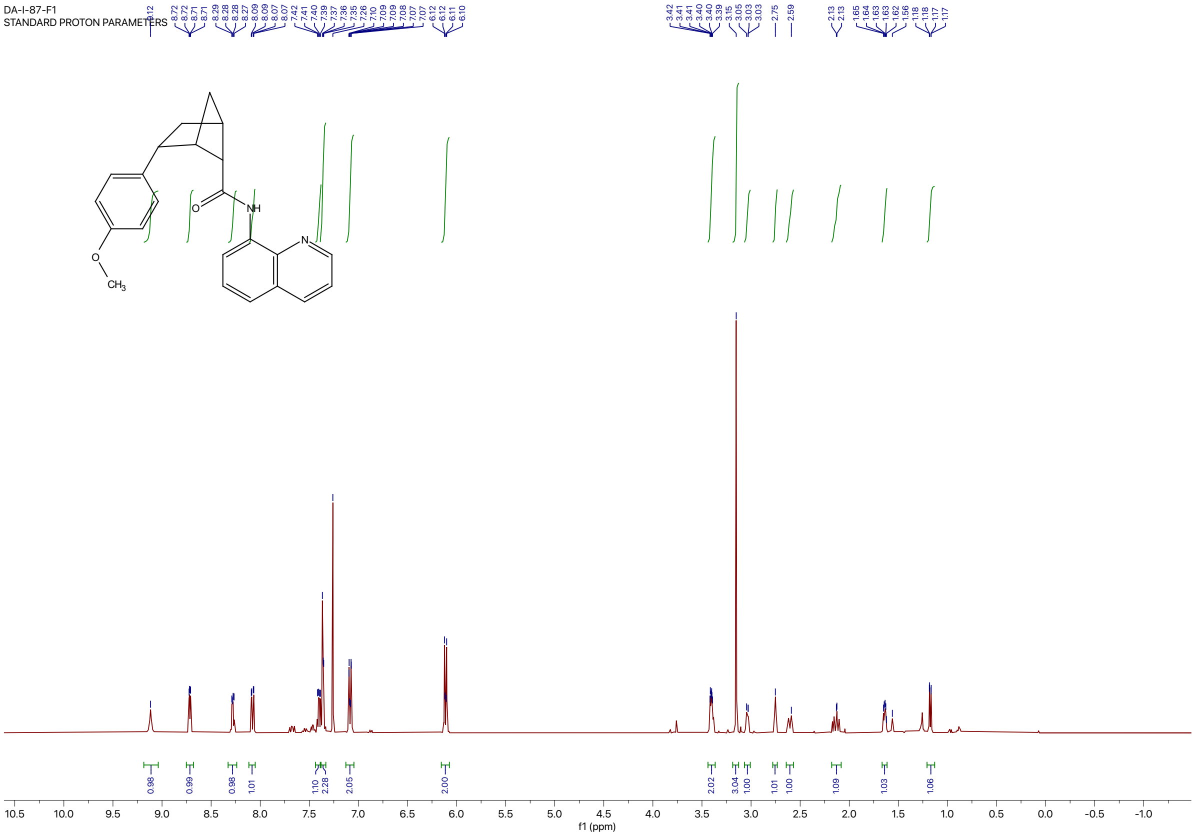
DA-I-87-F1
STANDARD PROTON PARAMETERS
9.12
8.72 8.72 8.71 8.71 8.29 8.28 8.28 8.27 8.09 8.09 8.07 8.07 7.42 7.41 7.40 7.39 7.37 7.36 7.35 7.26 7.10 7.09 7.09 7.08 7.07 7.07 6.12 6.12 6.11 6.10
3.42 3.41 3.41 3.40 3.40 3.39 3.15 3.05 3.03 3.03 2.75 2.59
2.13 2.13 1.65 1.64 1.63 1.63 1.62 1.56 1.18 1.18 1.17 1.17
O
NH
N
CH3
0.98 0.99 0.98 1.01 1.10 2.28 2.05 2.00 2.02 3.04 1.00 1.01 1.00 1.09 1.03 1.06
10.5 10.0 9.5 9.0 8.5 8.0 7.5 7.0 6.5 6.0 5.5 5.0 4.5 4.0 3.5 3.0 2.5 2.0 1.5 1.0 0.5 0.0 -0.5 -1.0
f1 (ppm)

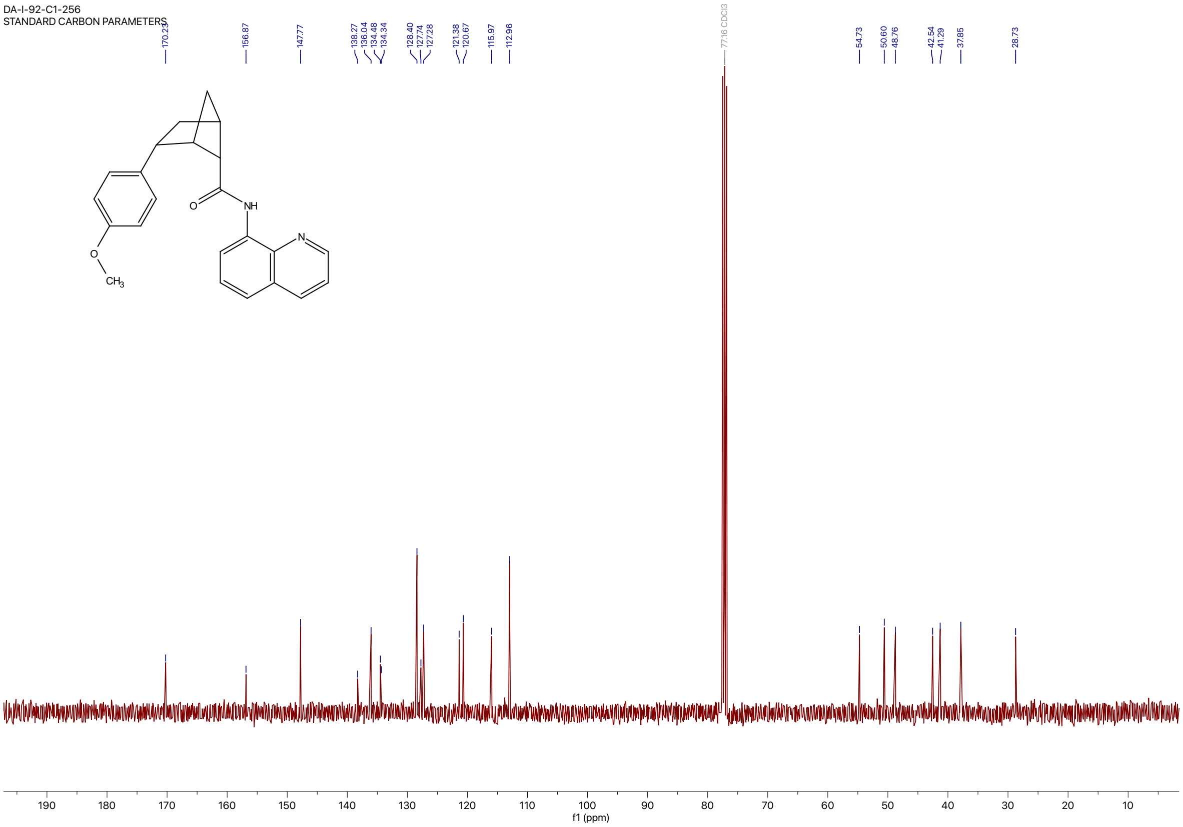
DA-I-92-C1-256
STANDARD CARBON PARAMETERS
170.23
156.87
147.77
138.27 136.04 134.48 134.34
128.40 127.74 127.28
121.38 120.67
115.97
112.96
77.16 CDCl3
54.73
50.60 48.76
42.54 41.29
37.85
28.73
O
NH
N
CH3
190 180 170 160 150 140 130 120 110 100 90 80 70 60 50 40 30 20 10
f1 (ppm)

39c

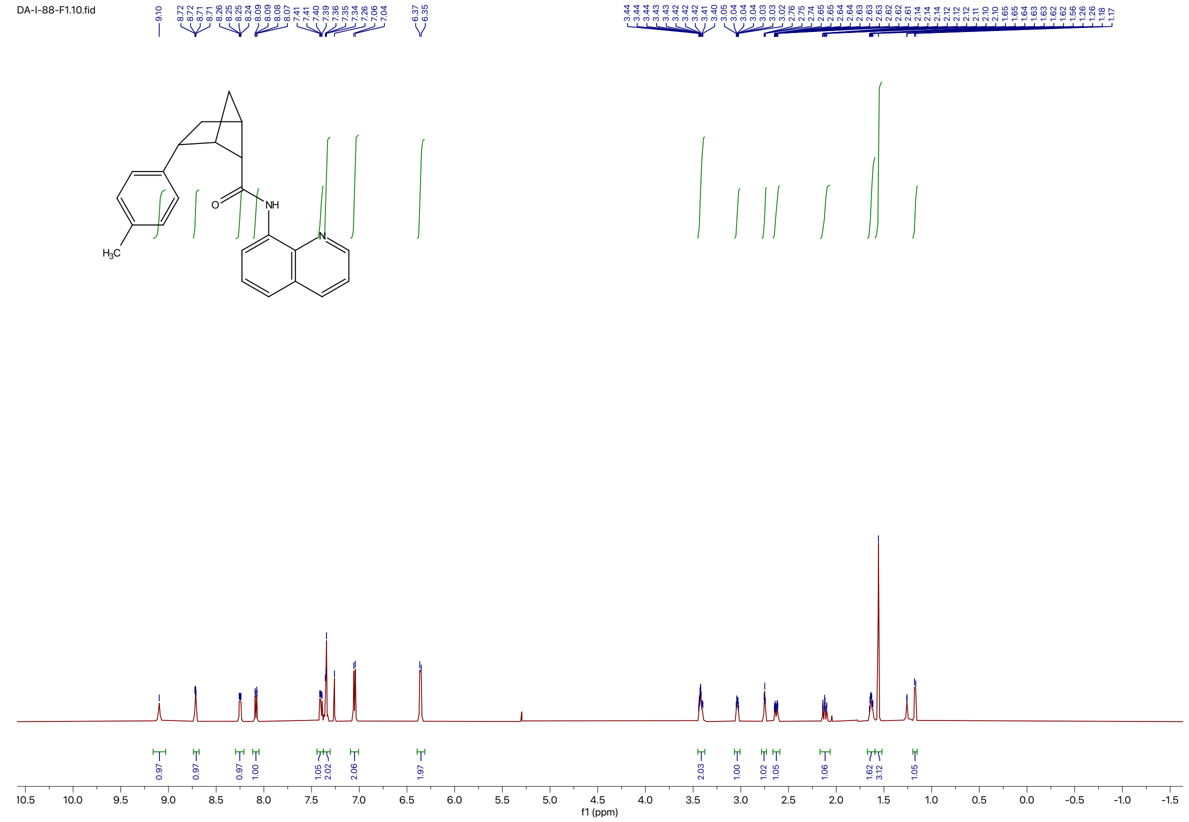
DA-I-88-F1.10.fid
O
NH
N
H3C
f1 (ppm)

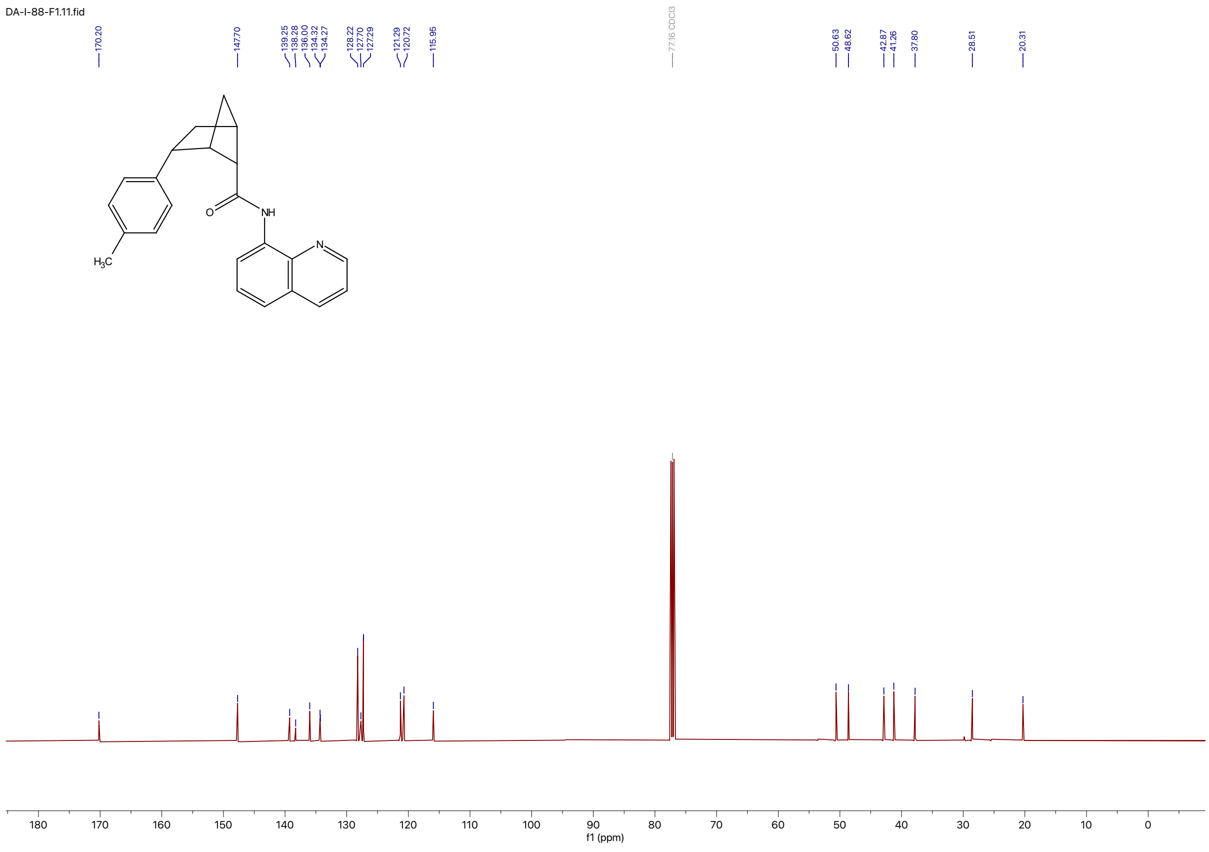
DA-I-88-F1.11.fid
170.20
147.70
139.25
138.28
136.00
134.32
134.27
128.22
127.70
127.29
121.29
120.72
115.95
77.16 CDCl3
50.63
48.62
42.87
41.26
37.80
28.51
20.31
O
NH
N
H3C
f1 (ppm)

39d

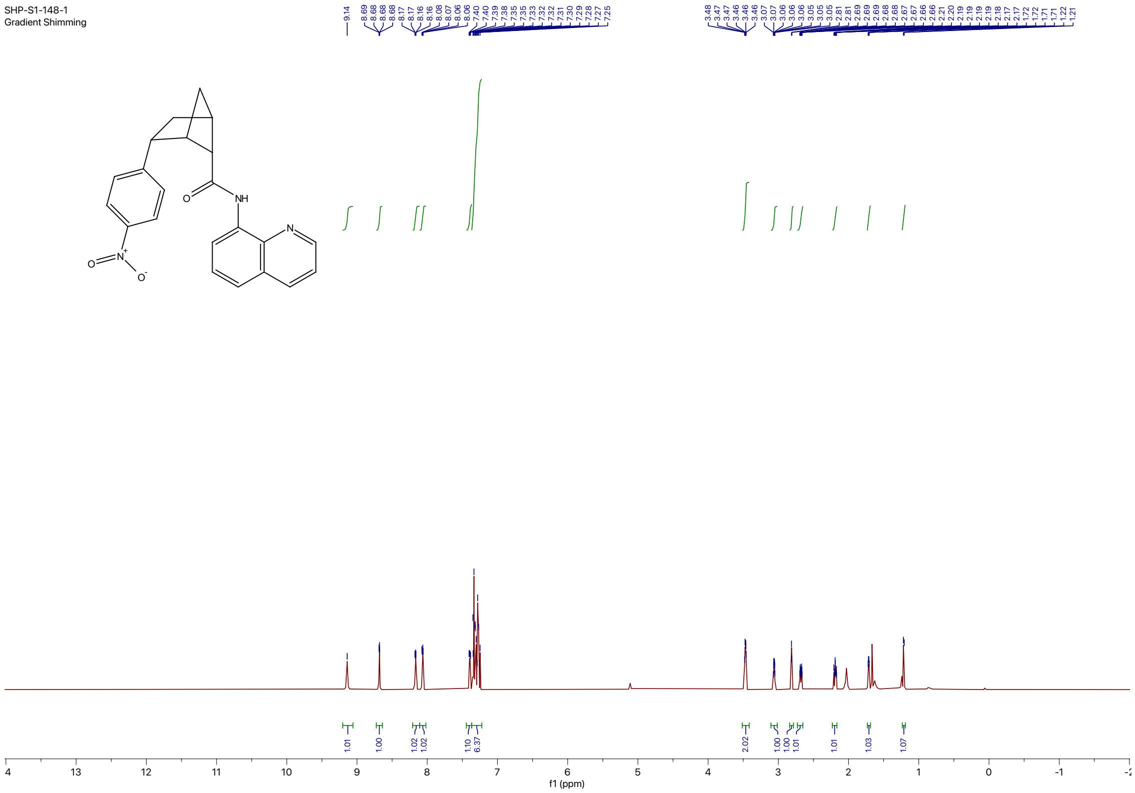
SHP-S1-148-1
Gradient Shimming
9.14 8.69 8.68 8.68 8.68 8.17 8.17 8.16 8.16 8.08 8.07 8.06 8.06 7.40 7.40 7.39 7.38 7.35 7.35 7.33 7.32 7.32 7.31 7.30 7.29 7.28 7.27 7.25
3.48 3.47 3.46 3.46 3.46 3.07 3.06 3.06 3.06 3.05 3.05 3.05 2.81 2.81 2.69 2.69 2.68 2.68 2.67 2.67 2.66 2.66 2.21 2.20 2.19 2.19 2.19 2.19 2.18 2.17 2.17 1.72 1.72 1.71 1.71 1.22 1.21
O
NH
N
O
N
O
1.01 1.00 1.02 1.02 1.10 6.37 2.02 1.00 1.00 1.01 1.01 1.03 1.07
f1 (ppm)

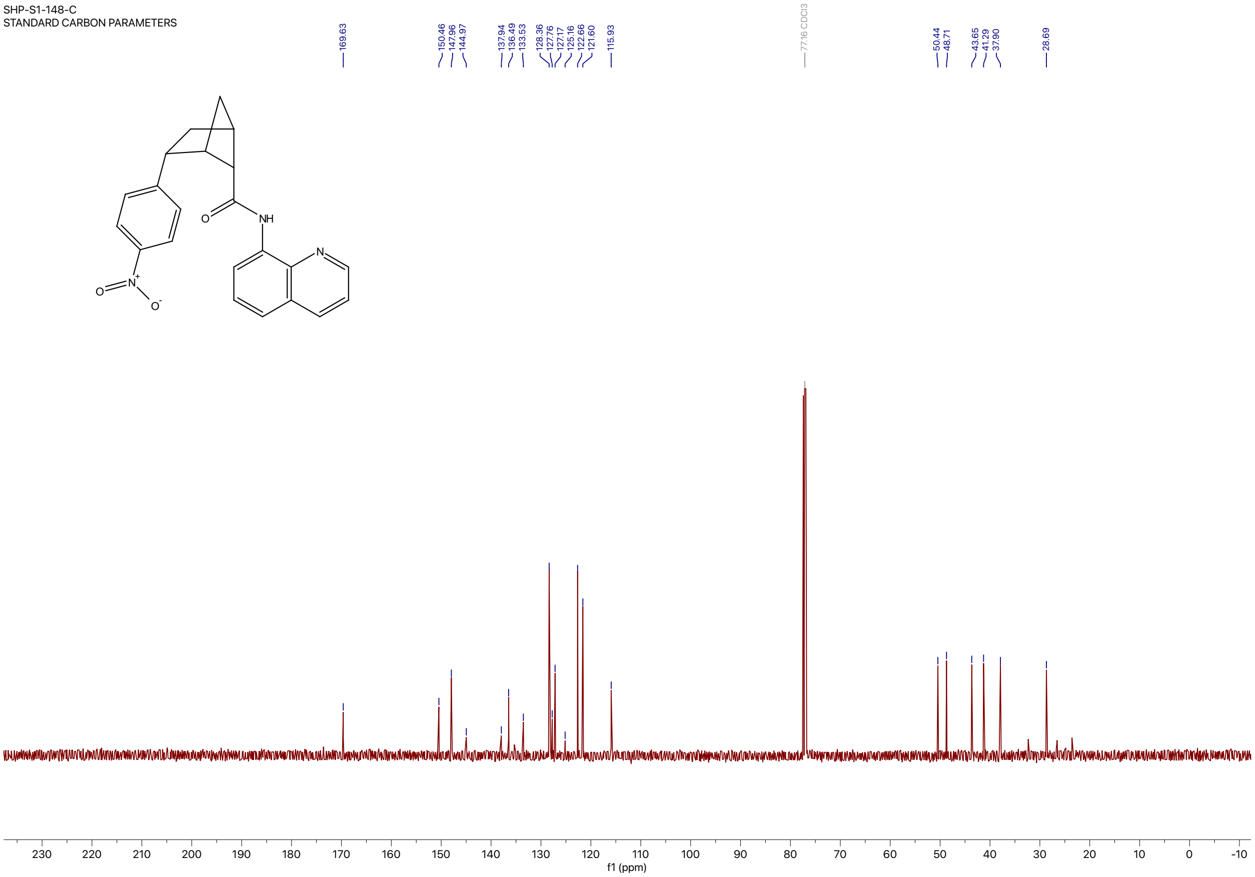
SHP-S1-148-C
STANDARD CARBON PARAMETERS
169.63
150.46 147.96 144.97
137.94 136.49 133.53 128.36 127.76 127.17 125.16 122.66 121.60 115.93
77.16 CDCl3
50.44 48.71 43.65 41.29 37.90 28.69
O
NH
N
O
N
O
f1 (ppm)

42

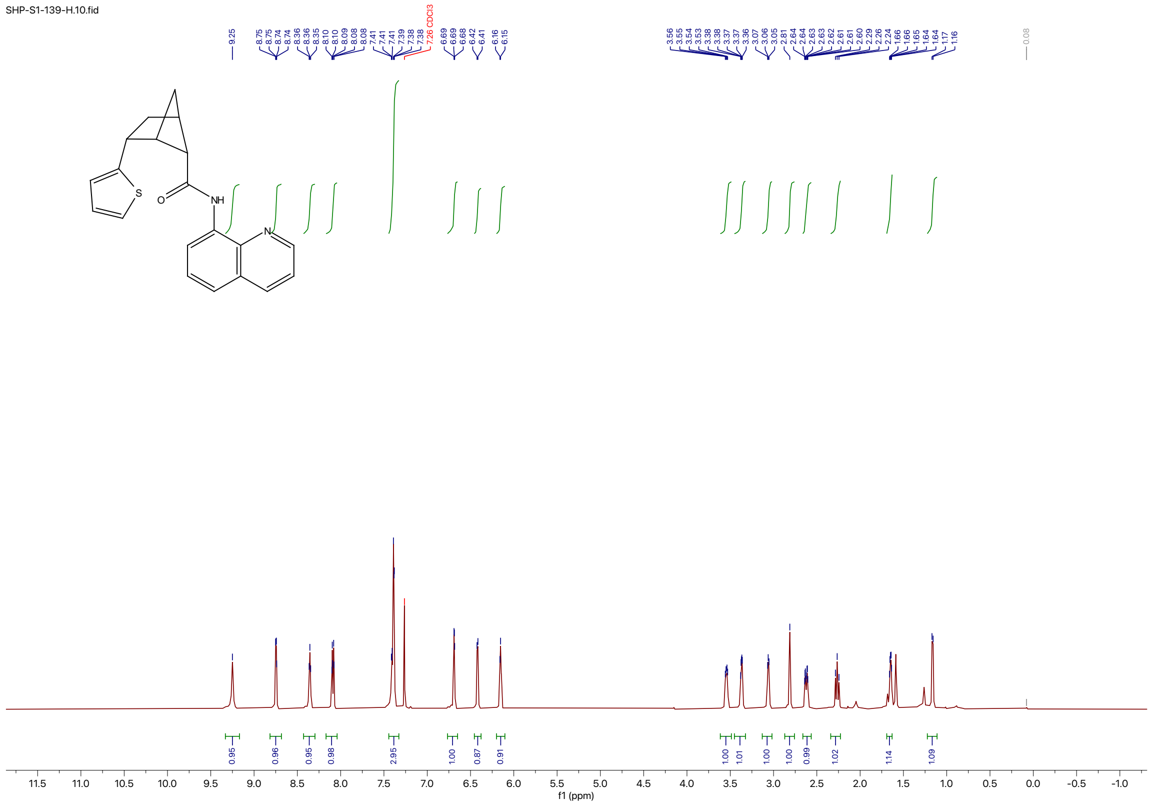
SHP-S1-139-H.10.fid
9.25
8.75
8.75
8.74
8.74
8.36
8.36
8.35
8.10
8.10
8.09
8.08
8.08
7.41
7.41
7.41
7.39
7.38
7.38
7.26 CDCl3
6.69
6.69
6.68
6.42
6.41
6.16
6.15
3.56
3.55
3.54
3.53
3.38
3.38
3.37
3.37
3.36
3.07
3.06
3.05
2.81
2.64
2.64
2.63
2.63
2.62
2.61
2.61
2.60
2.29
2.26
2.24
1.66
1.66
1.65
1.64
1.64
1.17
1.16
0.08
S
O
NH
N
0.95
0.96
0.95
0.98
2.95
1.00
0.87
0.91
1.00
1.01
1.00
1.00
0.99
1.02
1.14
1.09
f1 (ppm)

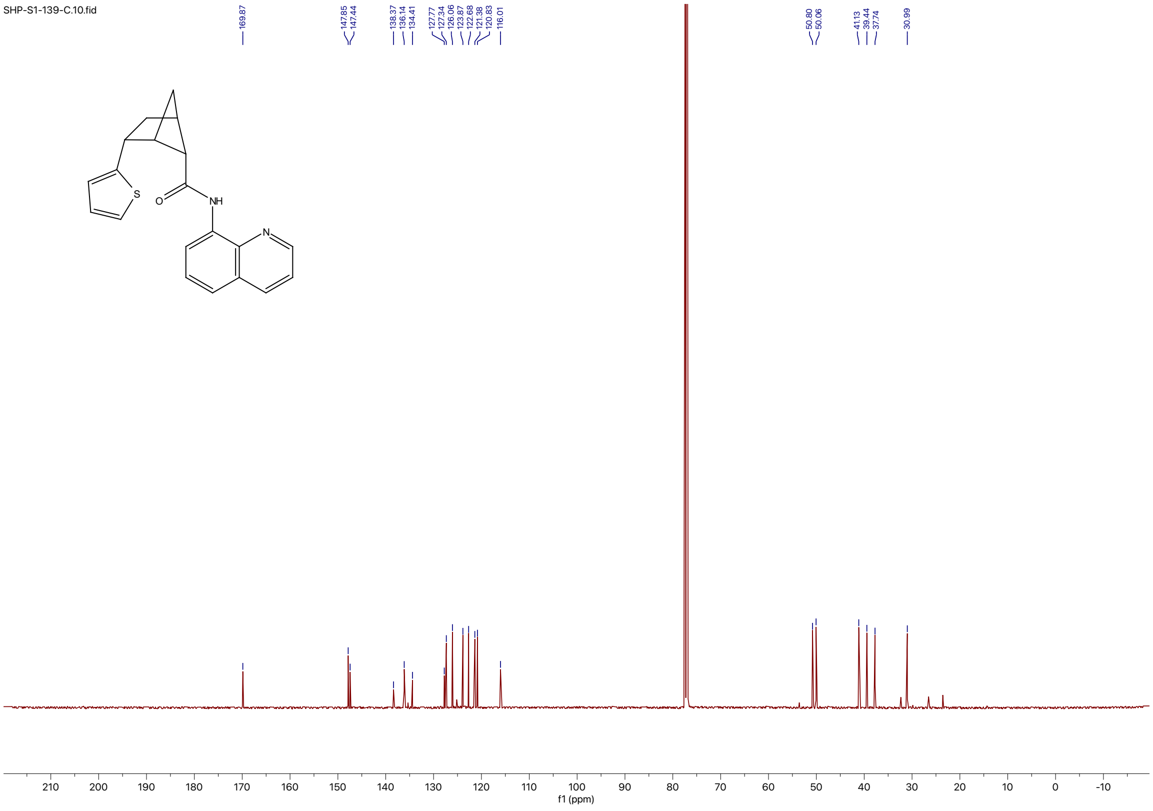
SHP-S1-139-C.10.fid
169.87
147.85
147.44
138.37
136.14
134.41
127.77
127.34
126.06
123.87
122.68
121.38
120.83
116.01
50.80
50.06
41.13
39.44
37.74
30.99
S
O
NH
N
f1 (ppm)

43

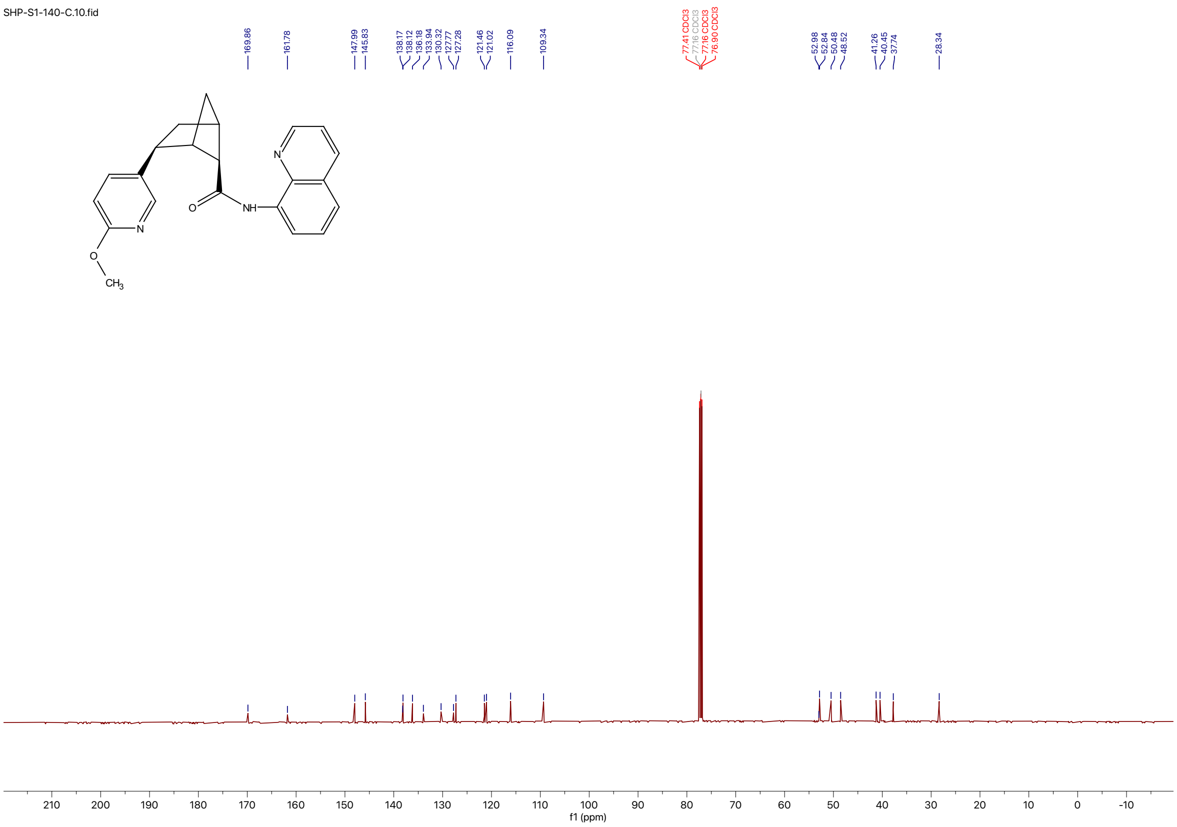
SHP-S1-140-C.10.fid
N
O
NH
O
N
CH3
f1 (ppm)

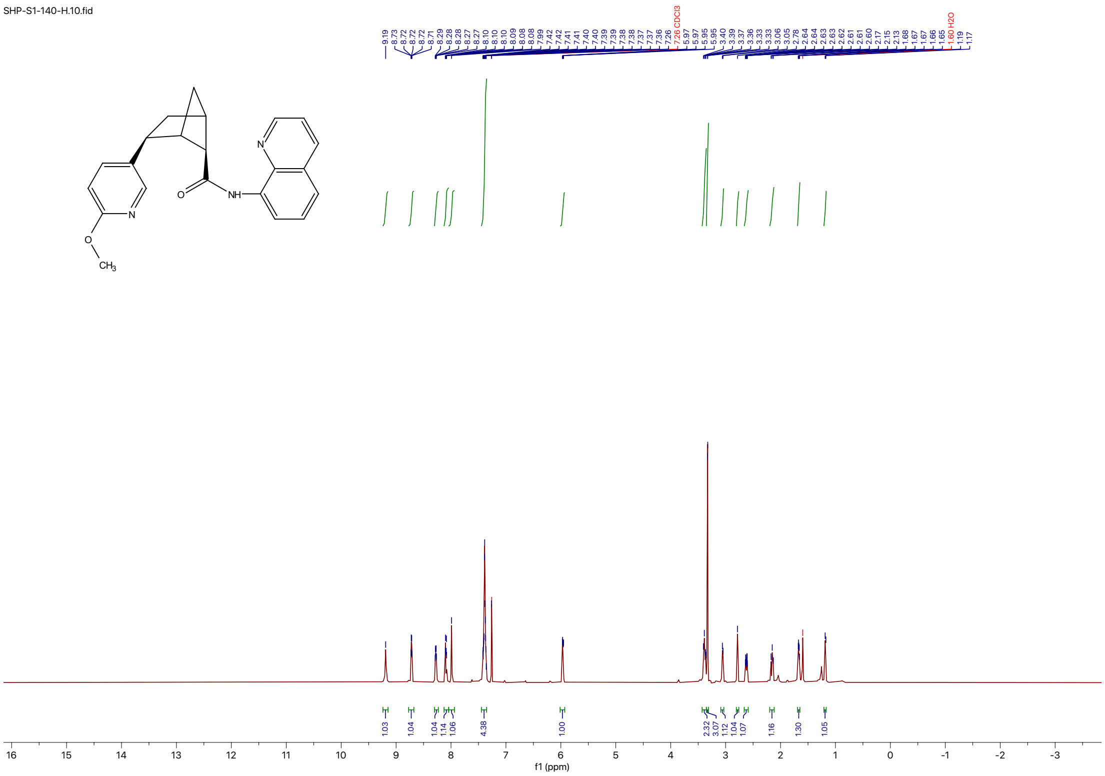
SHP-S1-140-H.10.fid
N
O
NH
O
N
CH3
f1 (ppm)

41

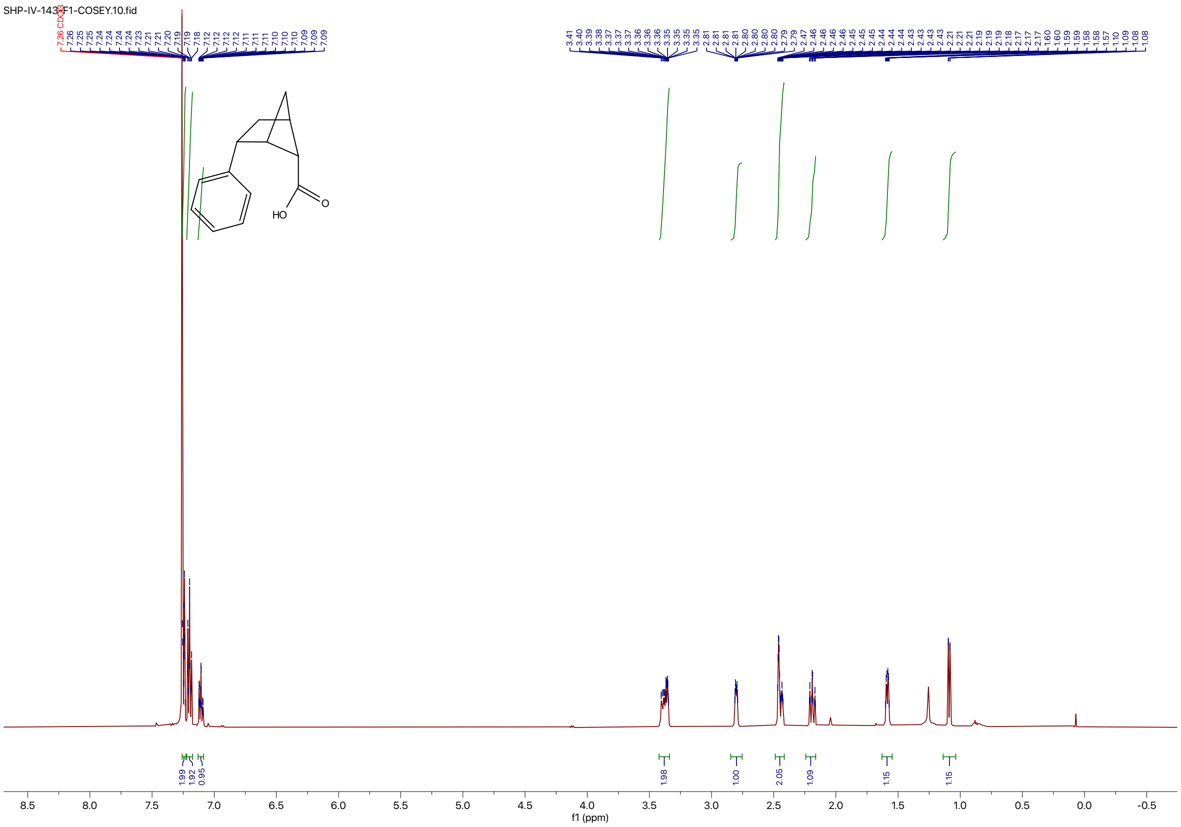
SHP-IV-143-F1-COSEY.10.fid
7.26 CDCl3
HO
O
1.99
1.92
0.95
1.98
1.00
2.05
1.09
1.15
1.15
f1 (ppm)

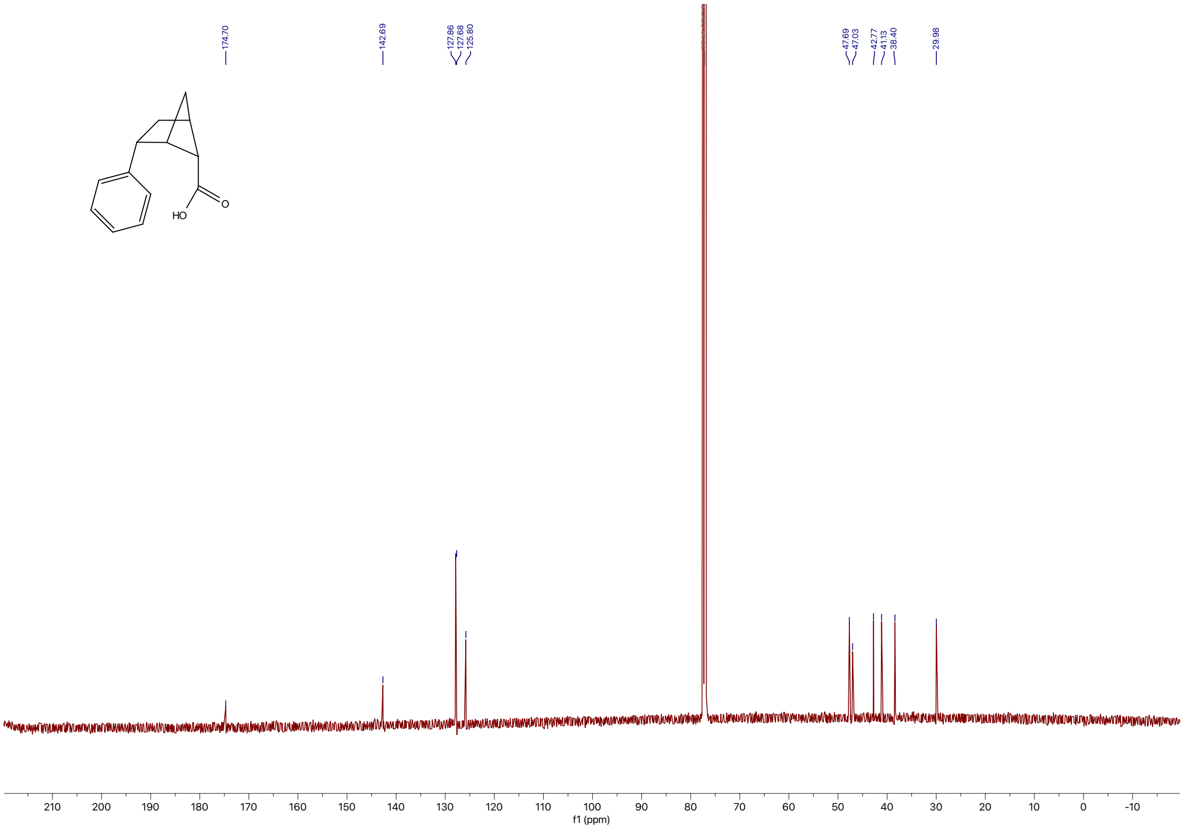
174.70
142.69
127.86
127.68
125.80
47.69
47.03
42.77
41.13
38.40
29.98
HO
O
f1 (ppm)

COSY analysis of 41

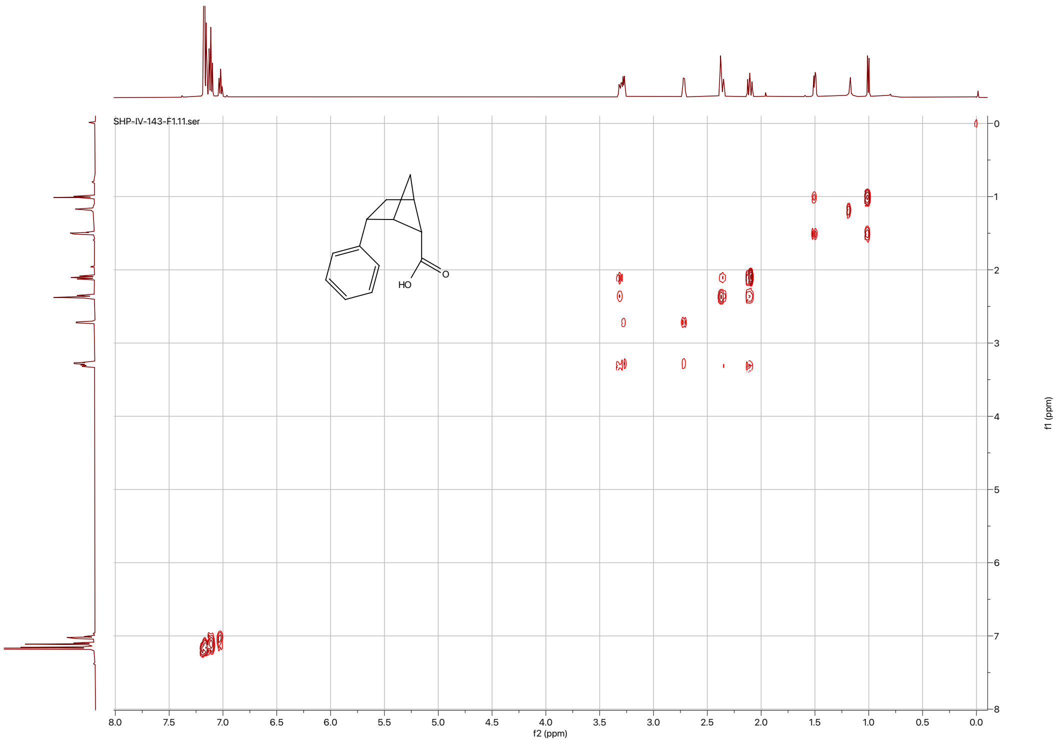

HSQC analysis of 41

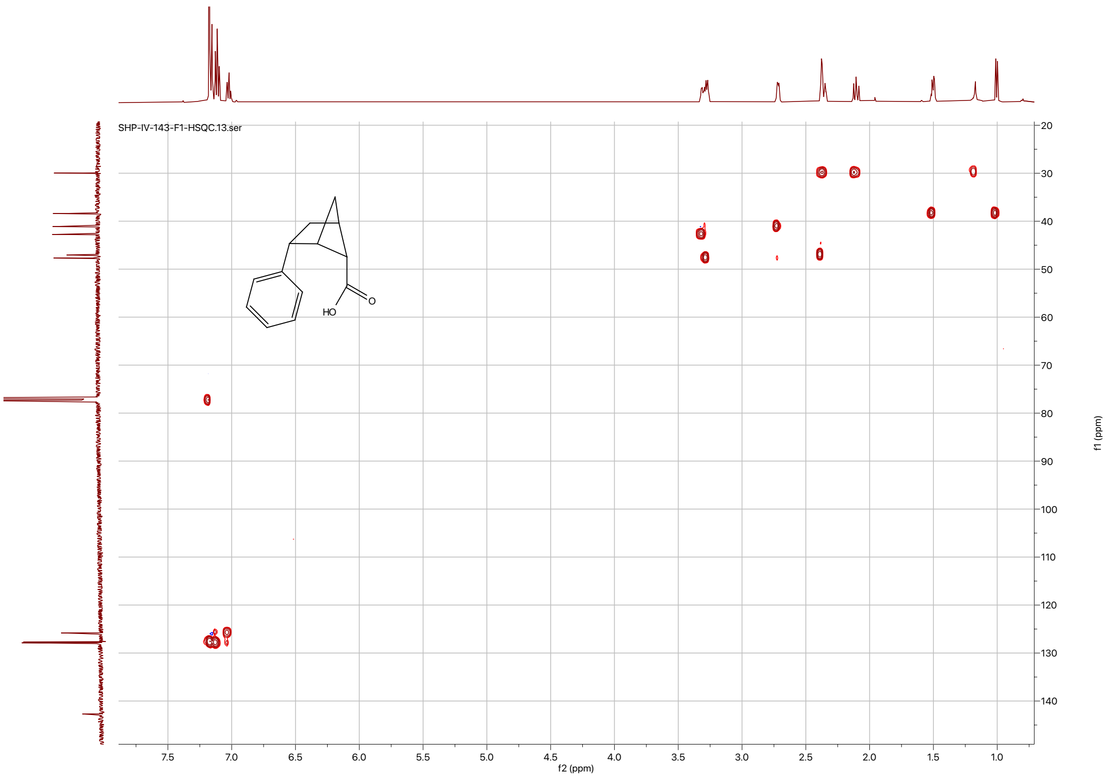

NOSEY analysis of 41

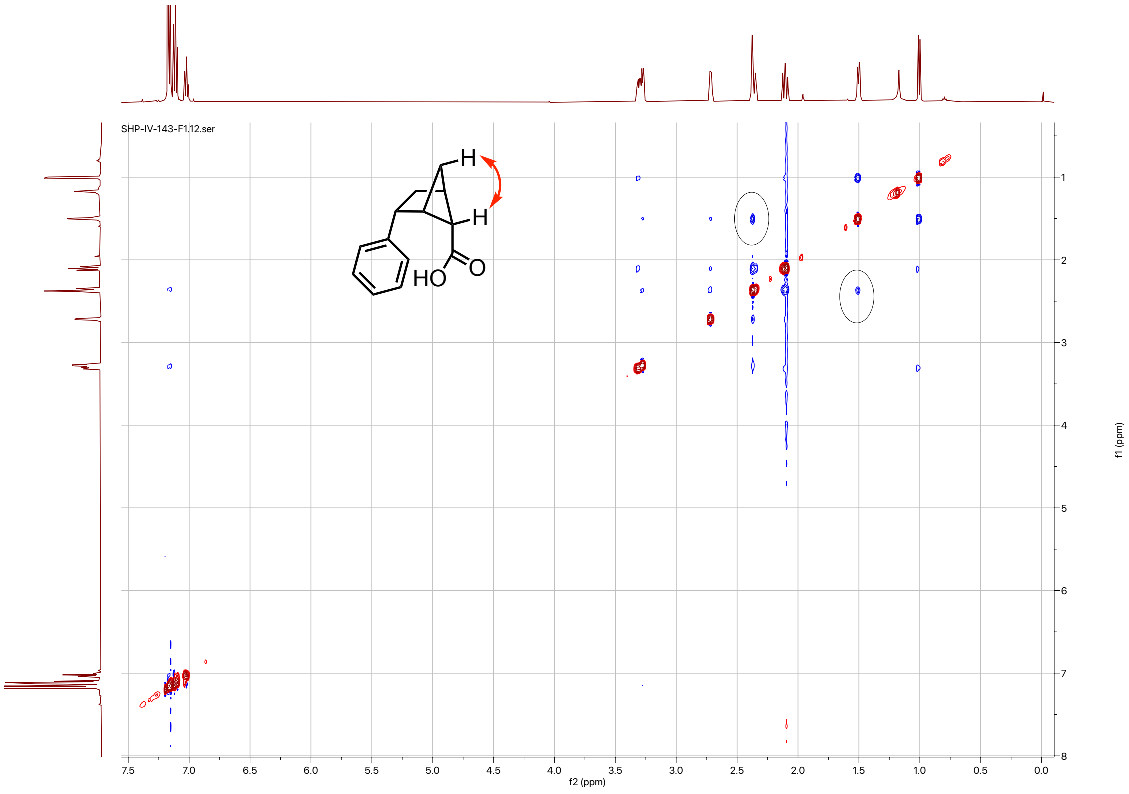

40

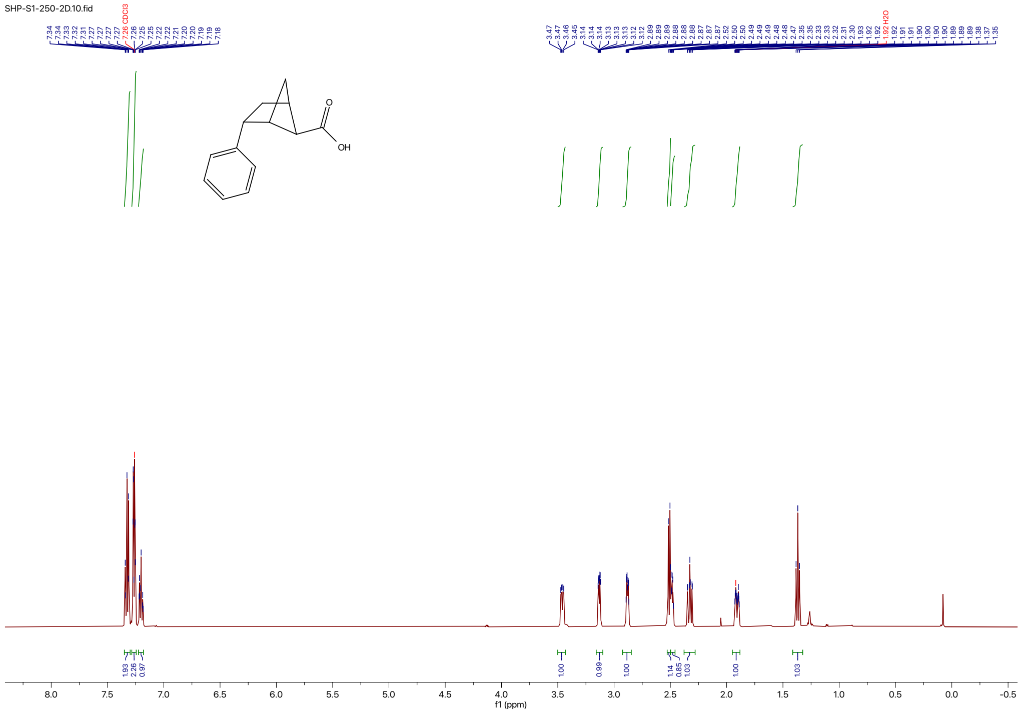

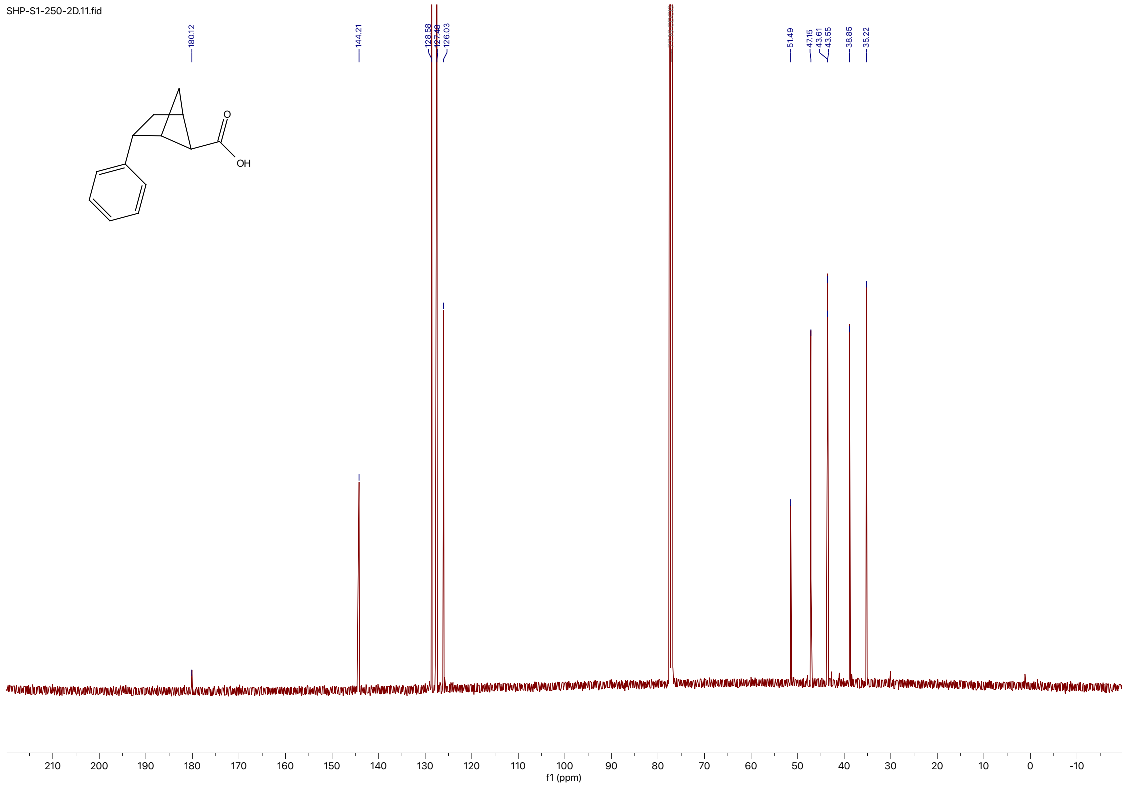

COSY analysis of 40

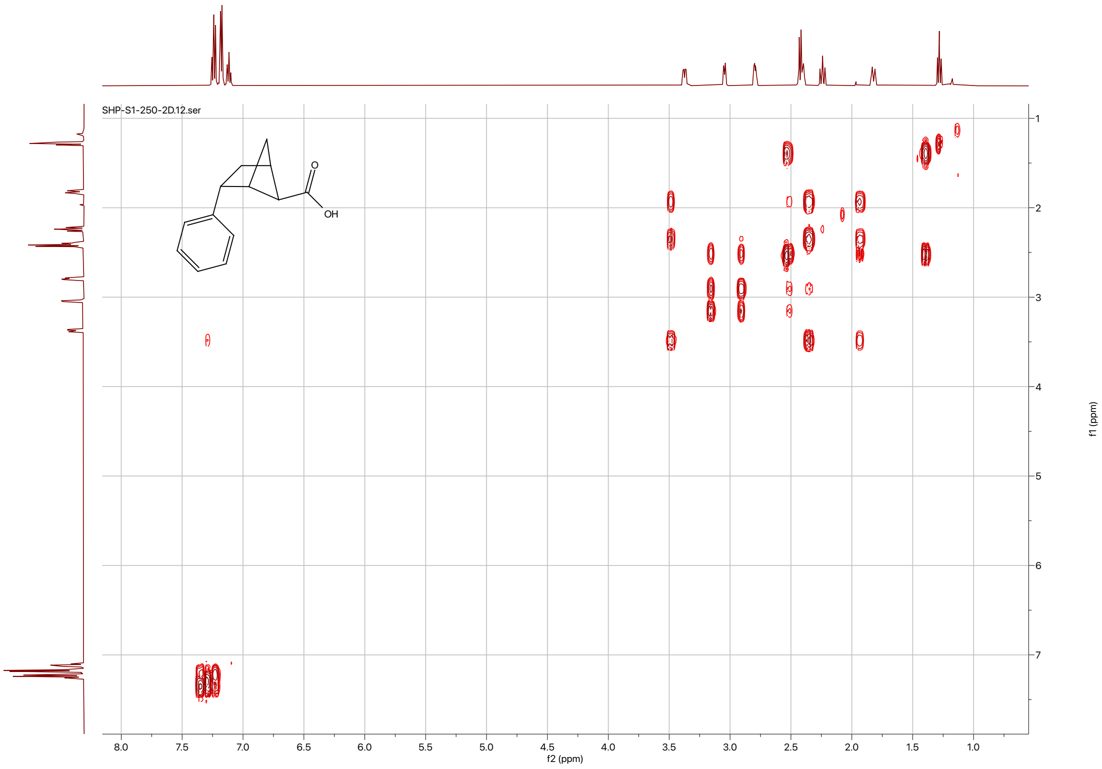

HSQC analysis of 40

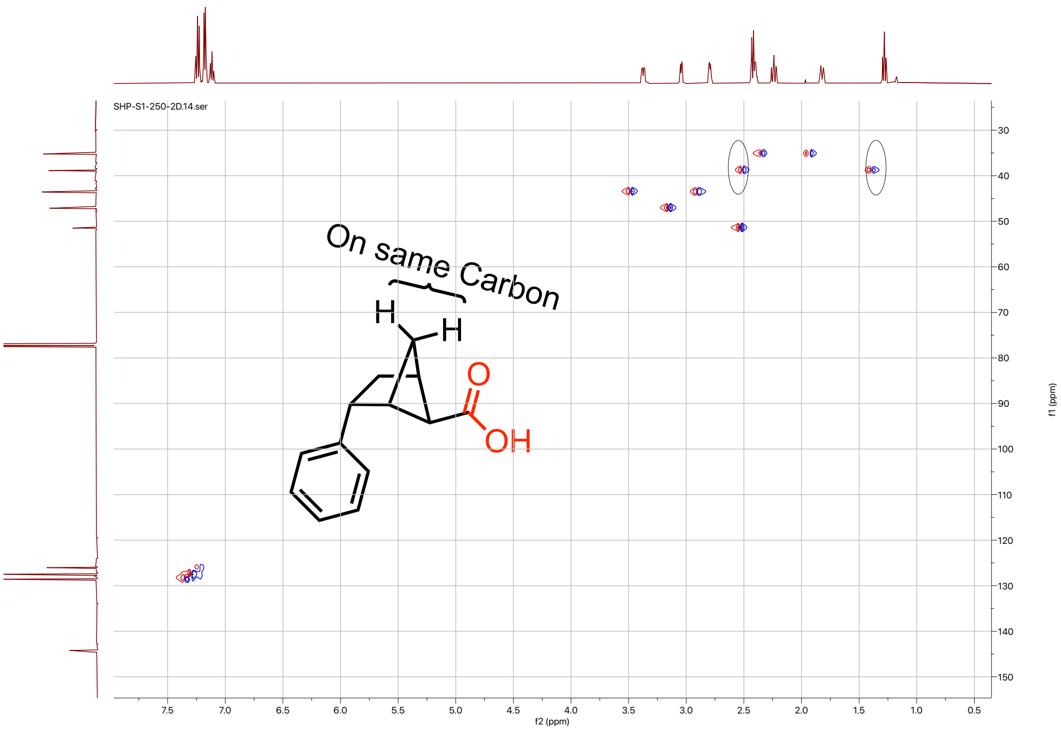

NOSEY analysis of 40

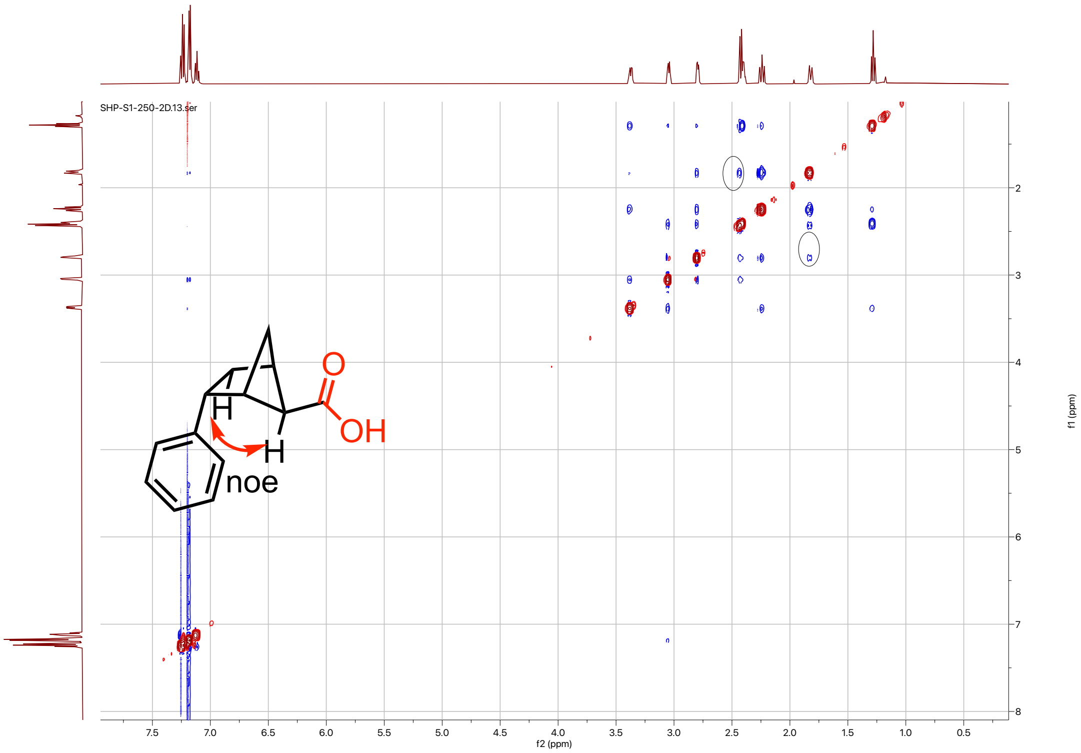

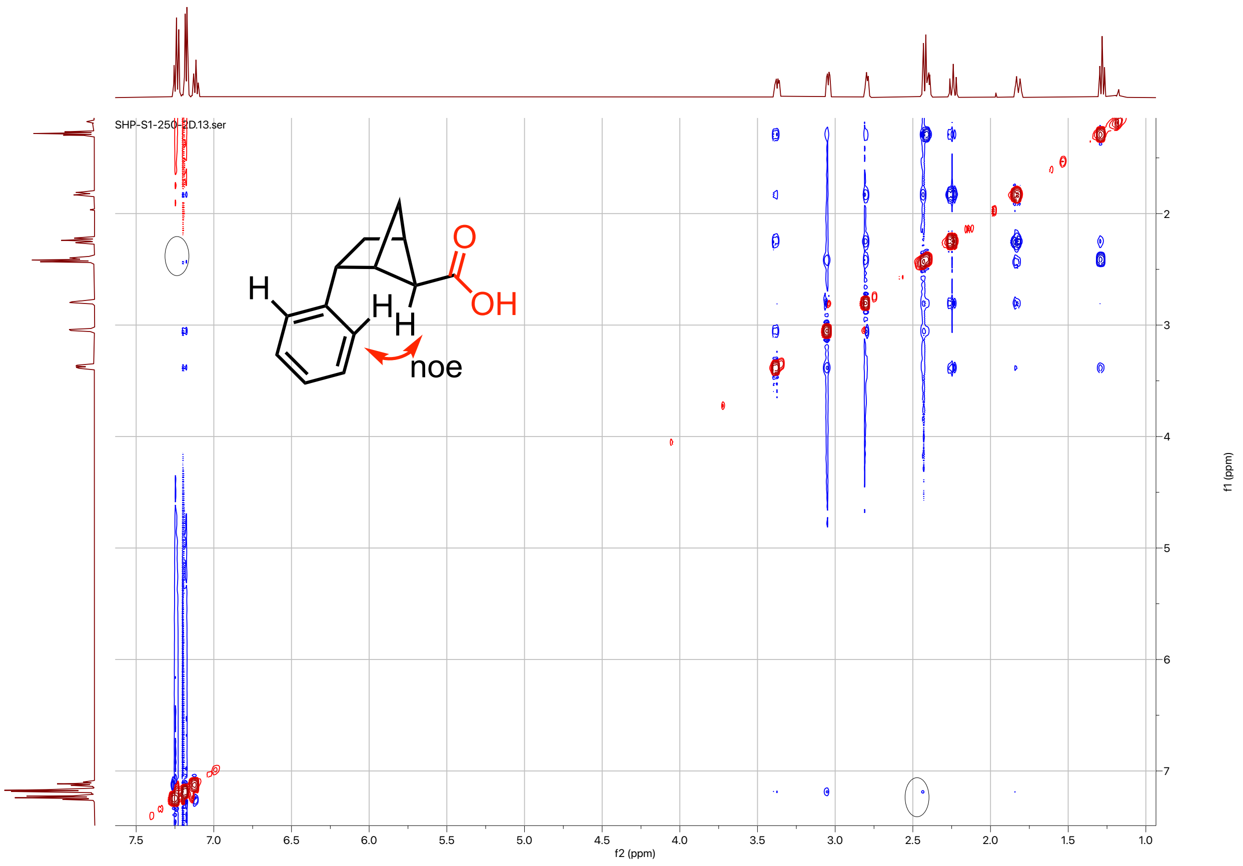
SHP-S1-250-2D.13.ser
H
H
H
O
OH
noe
f2 (ppm)
f1 (ppm)

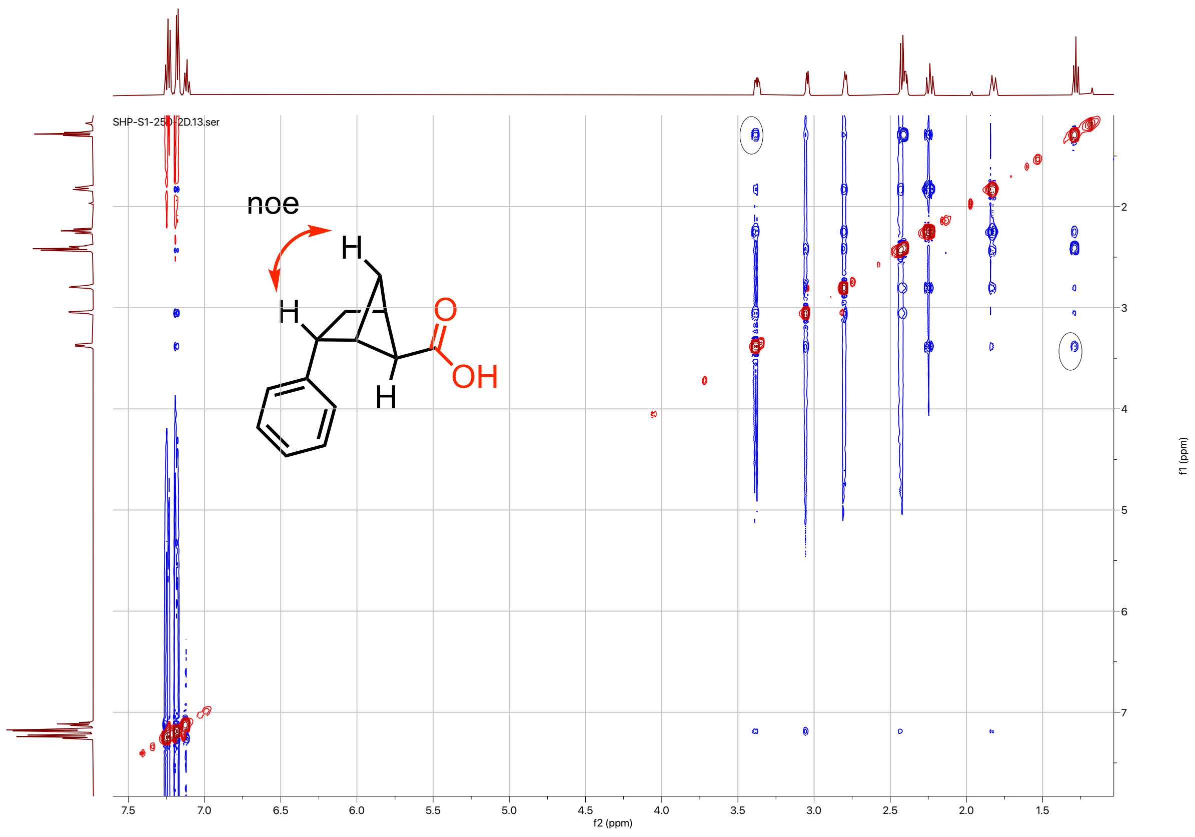
SHP-S1-250-2D.13.ser
noe
H
H
H
O
OH
f2 (ppm)
f1 (ppm)

46a

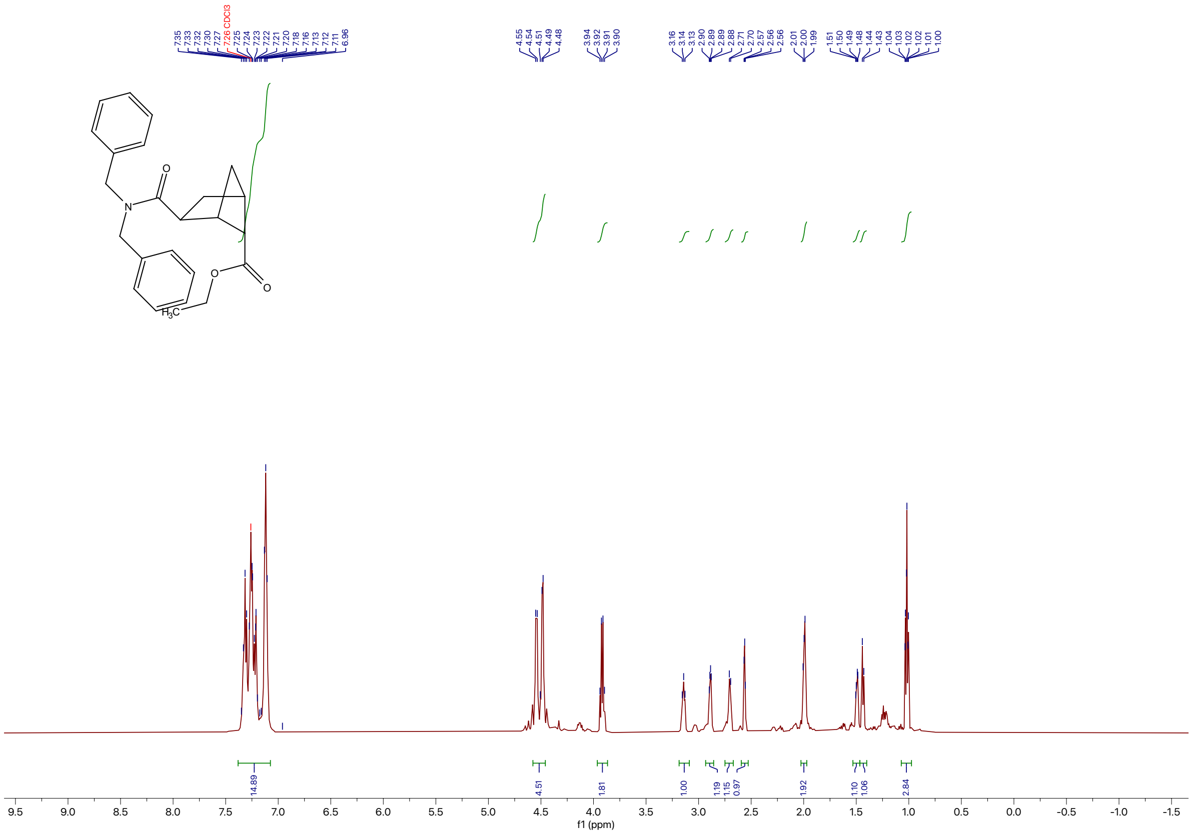

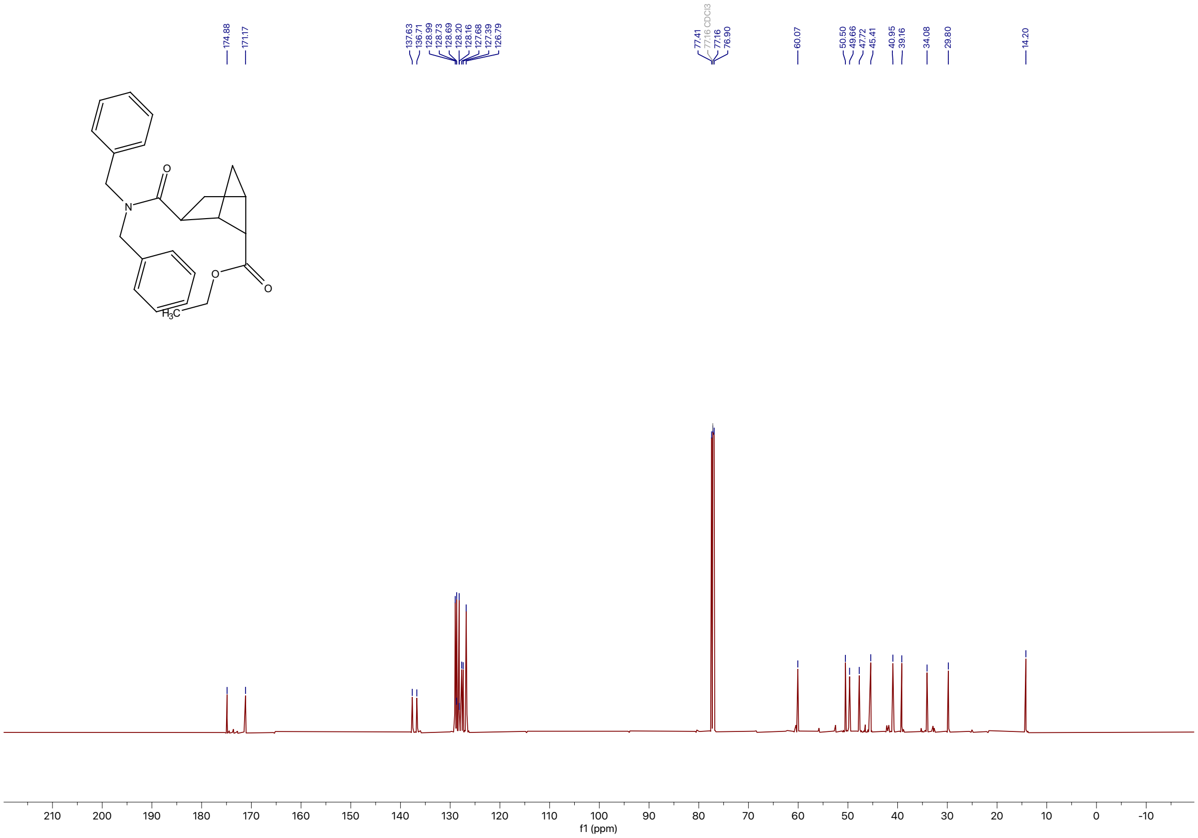

46b

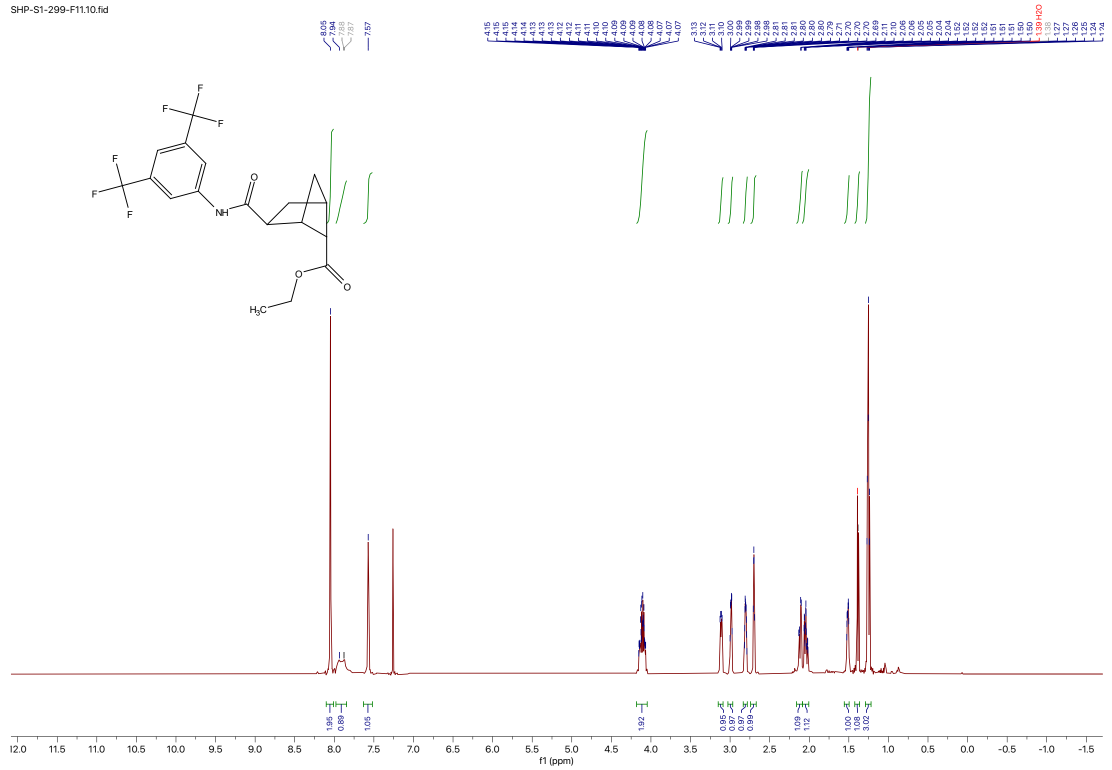

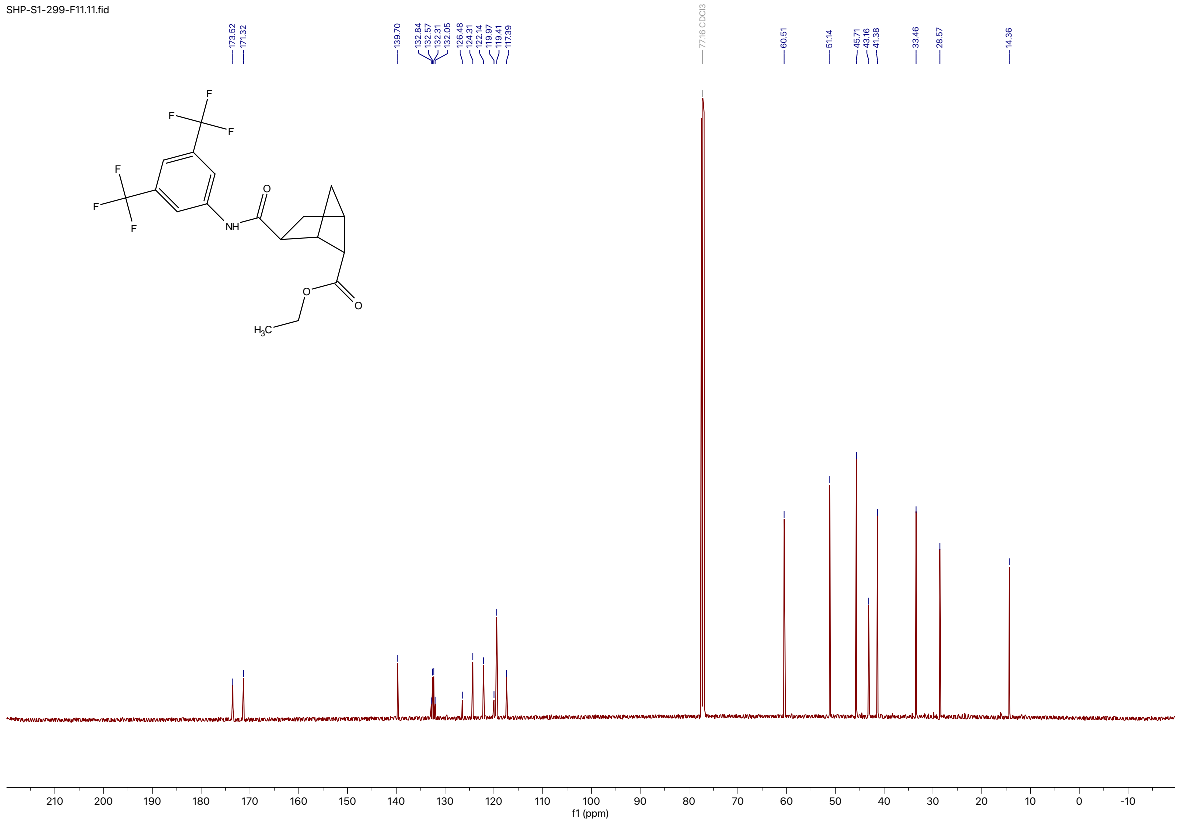

COSY NMR analysis of 46b

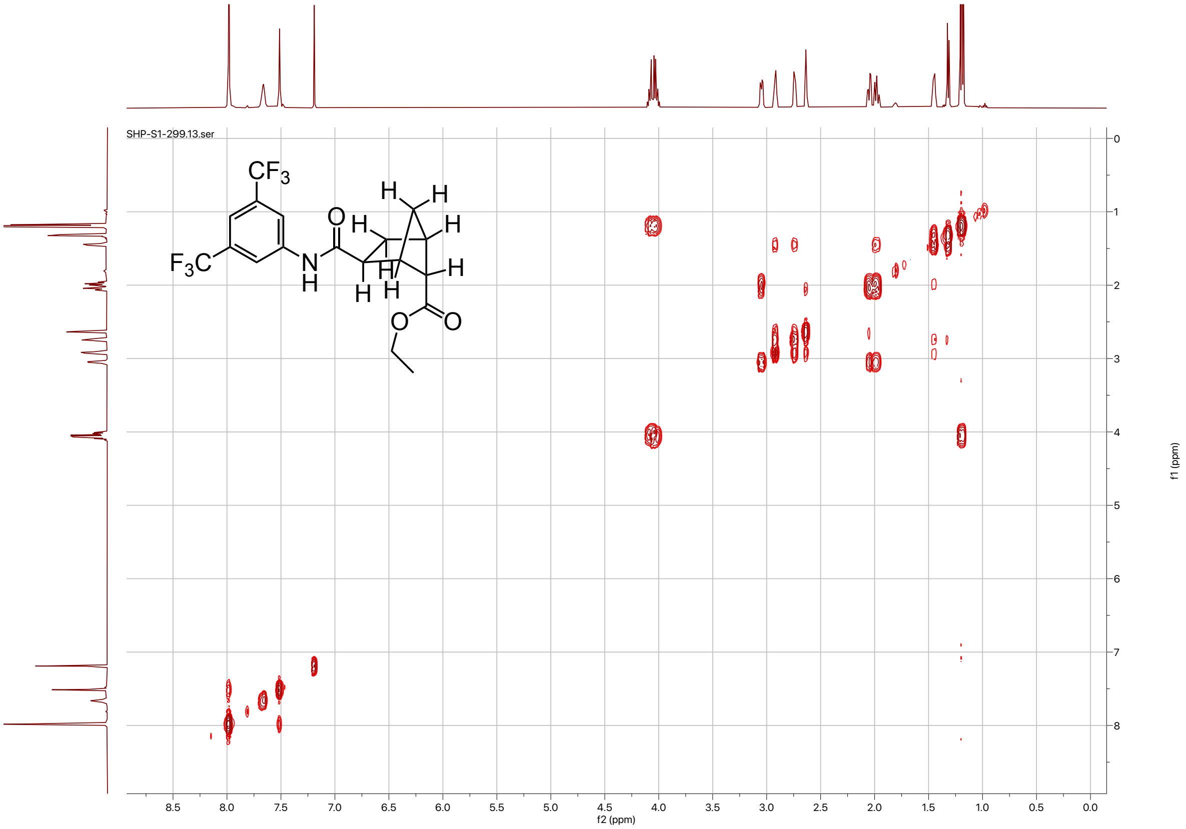

HMBC analysis of 46b

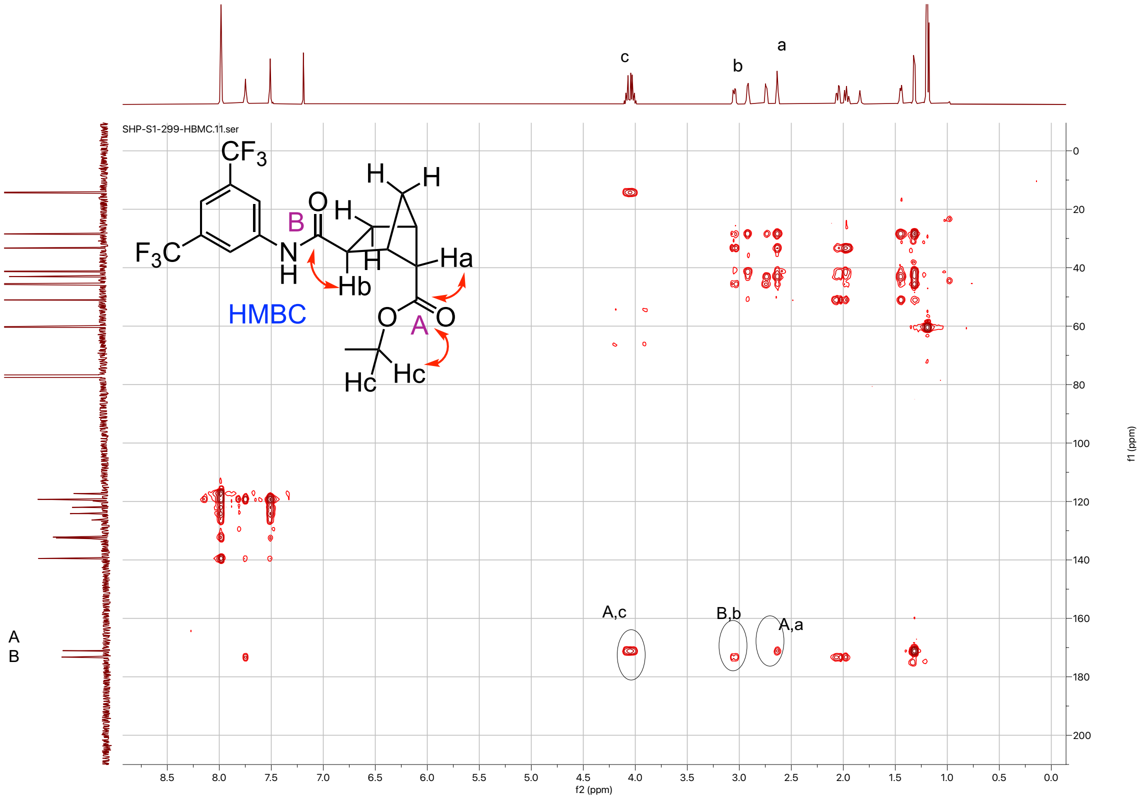

NOSEY NMR analysis of 46b

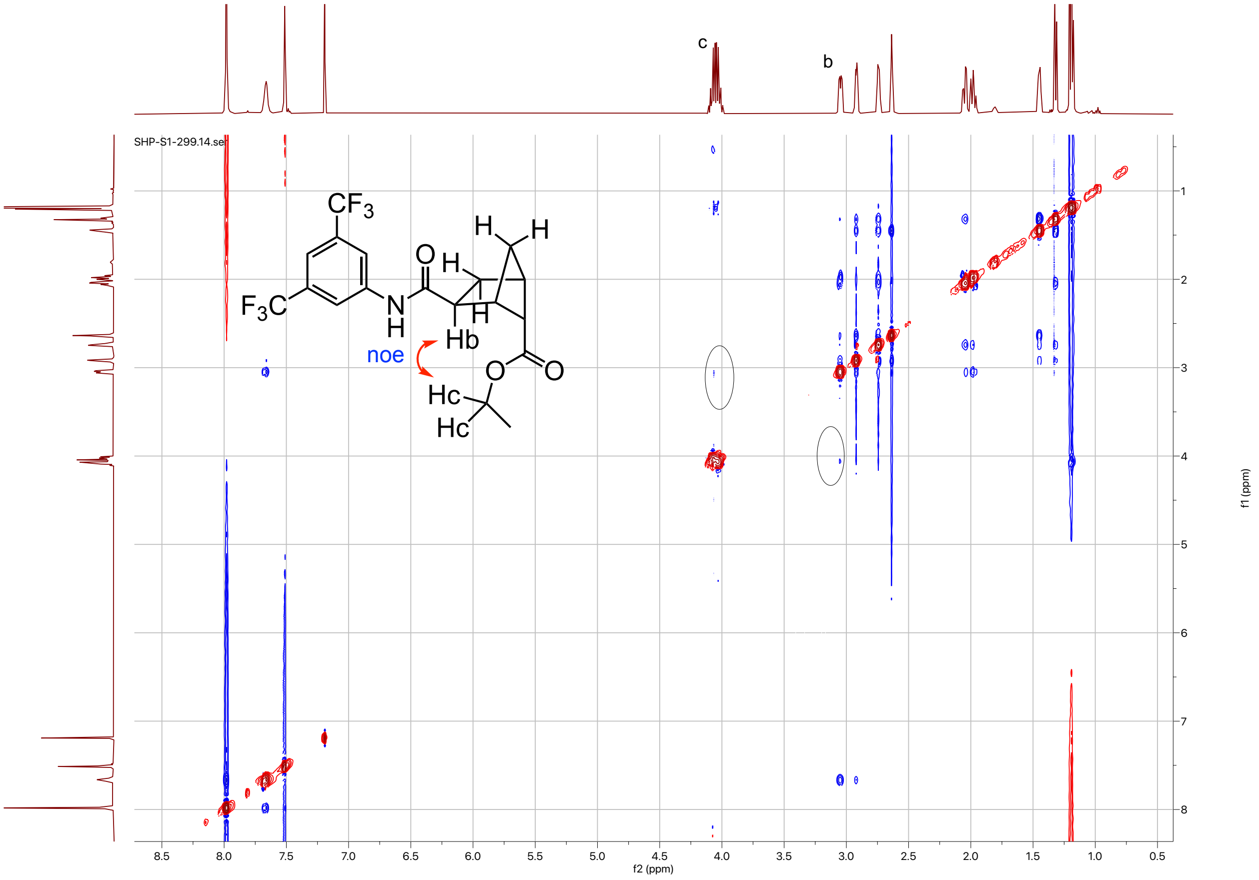

46c

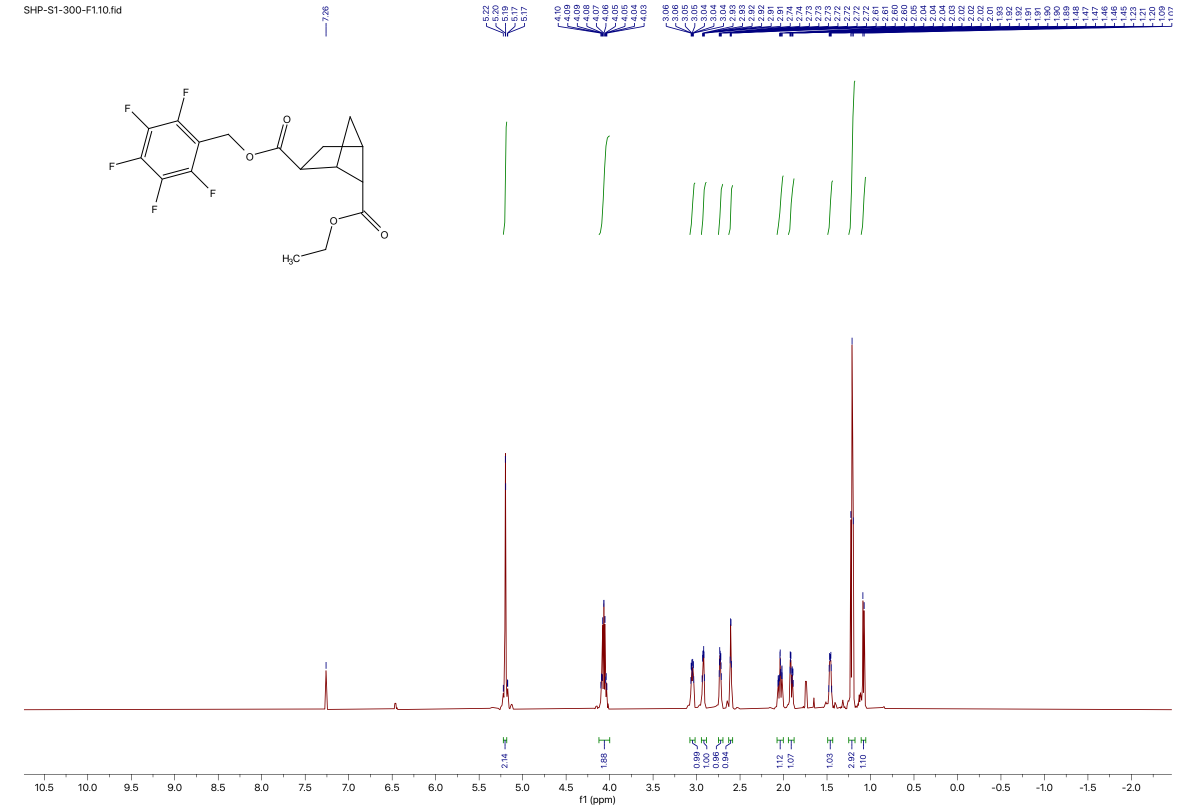

SHP-S1-300-F1.10.fid
7.26
5.22 5.20 5.19 5.17 5.17
4.10 4.09 4.09 4.08 4.07 4.06 4.05 4.05 4.04 4.03
3.06 3.06 3.05 3.05 3.04 3.04 3.04 2.93 2.93 2.92 2.92 2.91 2.91 2.74 2.74 2.73 2.73 2.73 2.72 2.72 2.72 2.72 2.61 2.61 2.60 2.60 2.05 2.04 2.04 2.04 2.03 2.02 2.02 2.02 2.01 1.93 1.92 1.92 1.91 1.91 1.90 1.90 1.89 1.48 1.47 1.47 1.46 1.46 1.45 1.23 1.21 1.20 1.09 1.07
F
O
H3C
2.14
1.88
0.99 1.00 0.96 0.94
1.12 1.07
1.03
2.92 1.10
10.5 10.0 9.5 9.0 8.5 8.0 7.5 7.0 6.5 6.0 5.5 5.0 4.5 4.0 3.5 3.0 2.5 2.0 1.5 1.0 0.5 0.0 -0.5 -1.0 -1.5 -2.0
f1 (ppm)

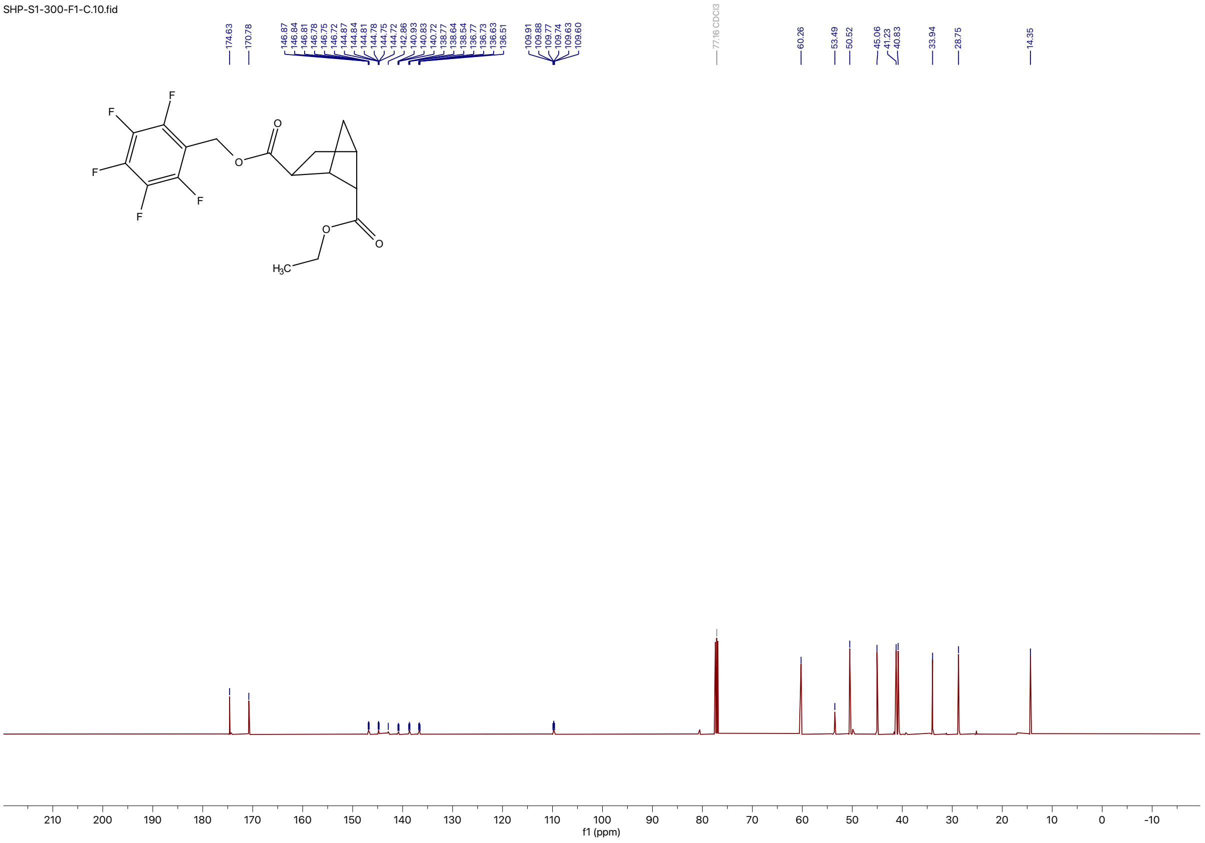

SHP-S1-300-F1-C.10.fid
174.63
170.78
146.87 146.84 146.81 146.78 146.75 146.72 144.87 144.84 144.81 144.78 144.75 144.72 142.86 140.93 140.83 140.72 138.77 138.64 138.54 136.77 136.73 136.63 136.51
109.91 109.88 109.77 109.74 109.63 109.60
77.16 CDCl3
60.26
53.49
50.52
45.06 41.23 40.83
33.94
28.75
14.35
F
O
H3C
210 200 190 180 170 160 150 140 130 120 110 100 90 80 70 60 50 40 30 20 10 0 -10
f1 (ppm)

46d

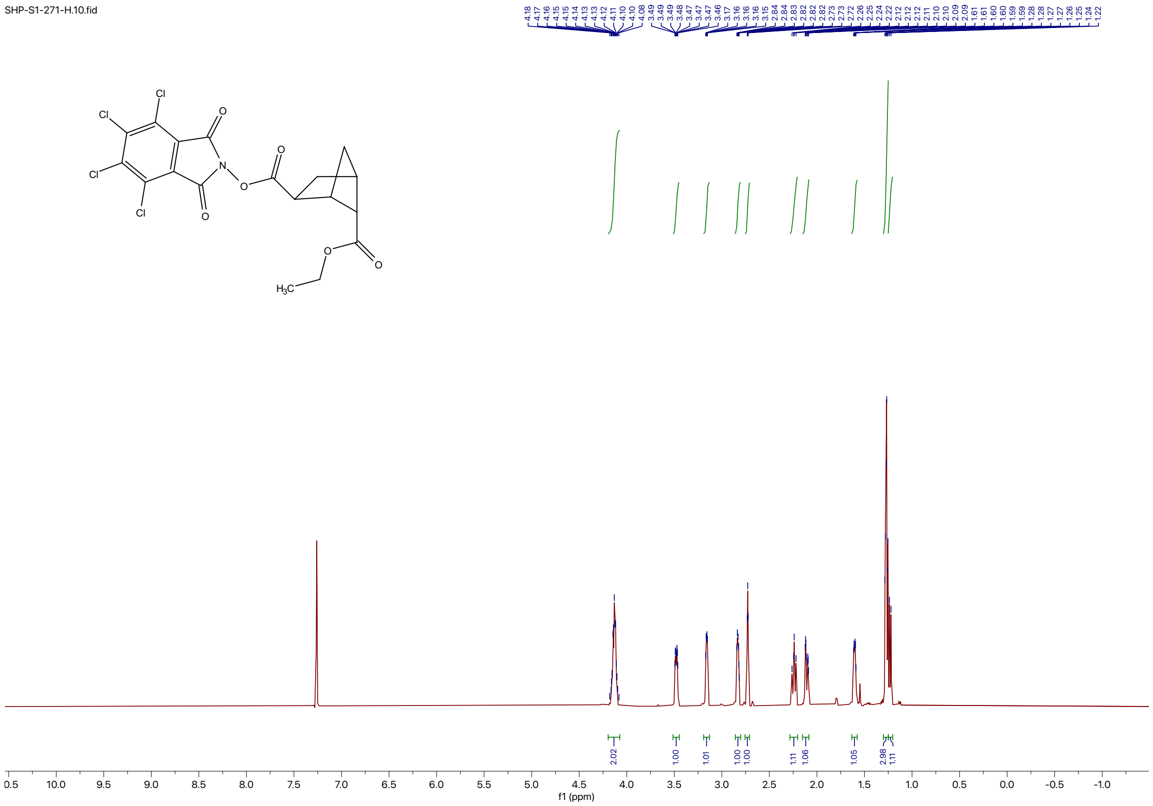
SHP-S1-271-H.10.fid
Cl
Cl
Cl
Cl
O
N
O
O
O
O
H3C
2.02
1.00
1.01
1.00
1.00
1.11
1.06
1.05
2.98
1.11
f1 (ppm)

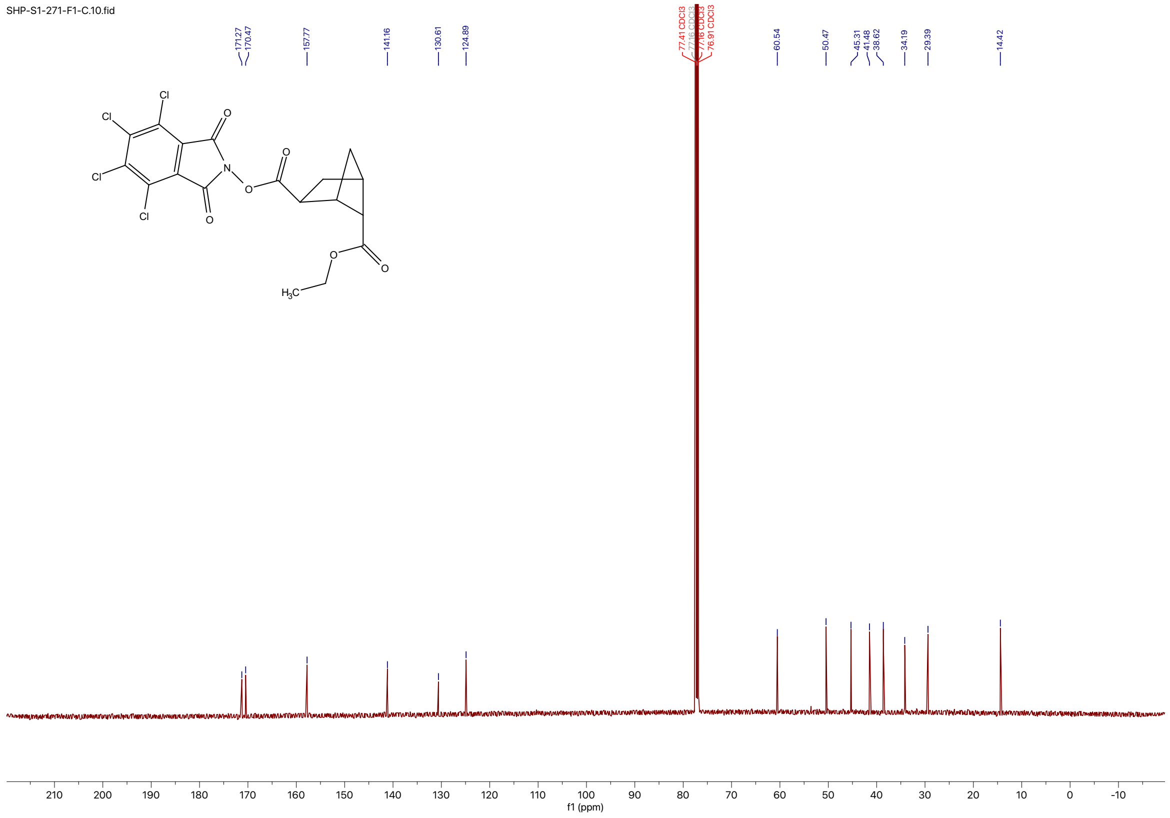
SHP-S1-271-F1-C.10.fid
171.27
170.47
157.77
141.16
130.61
124.89
77.41 CDCl3
77.16 CDCl3
76.91 CDCl3
60.54
50.47
45.31
41.48
38.62
34.19
29.39
14.42
Cl
Cl
Cl
Cl
O
N
O
O
O
O
H3C
f1 (ppm)

47

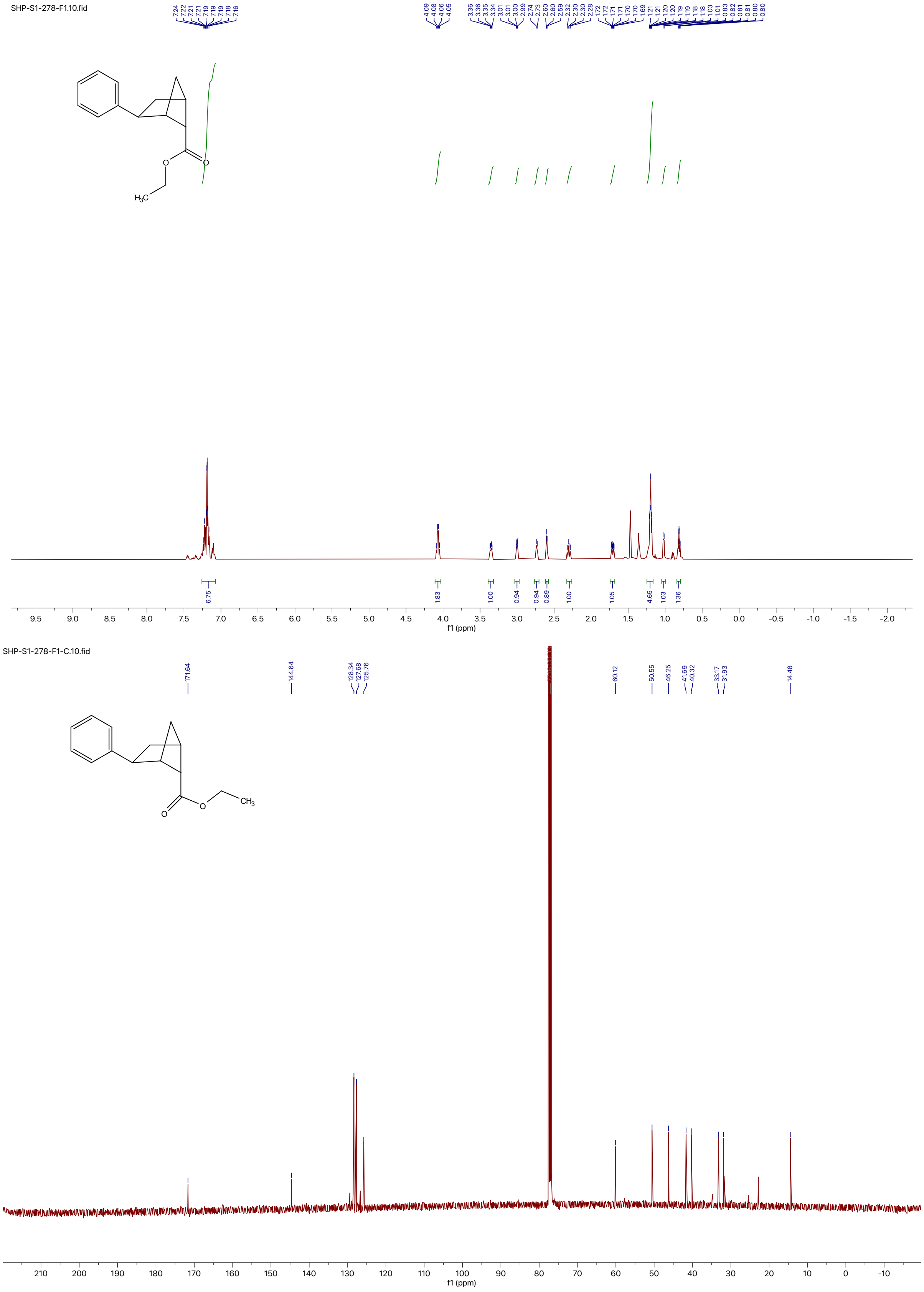
SHP-S1-278-F1.10.fid
7.24
7.22
7.21
7.21
7.19
7.19
7.19
7.19
7.18
7.16
4.09
4.08
4.06
4.05
3.36
3.36
3.35
3.34
3.01
3.01
3.00
2.99
2.74
2.73
2.60
2.60
2.59
2.32
2.30
2.30
2.28
1.72
1.72
1.71
1.71
1.70
1.70
1.69
1.21
1.21
1.20
1.20
1.19
1.19
1.18
1.18
1.03
1.01
0.83
0.82
0.81
0.81
0.80
0.80
H3C
O
O
6.75
1.83
1.00
0.94
0.94
0.89
1.00
1.05
4.65
1.03
1.36
f1 (ppm)
SHP-S1-278-F1-C.10.fid
171.64
144.64
128.34
127.68
125.76
60.12
50.55
46.25
41.69
40.32
33.17
31.93
14.48
O
O
CH3
f1 (ppm)

X-Ray data:

Single crystals suitable for X-ray diffraction were grown by Slow evaporation of a mixture of ethyl acetate and pentane. A colourless crystal (block, approximate dimensions 0.19 × 0.14 × 0.13 mm3) was placed onto the tip of a MiTeGen pin and mounted on a Bruker Venture D8 diffractometer equipped with a PhotonIII detector at 143.0 K.

Data collection

The data collection was carried out using Mo Kα _radiation (λ _= 0.71073 Å, graphite monochromator) with a frame time of 1 second and a detector distance of 40 mm. A collection strategy was calculated and complete data to a resolution of 0.77 Å with a redundancy of 5.4 were collected. The frames were integrated with the Bruker SAINT1 software package using a narrow-frame algorithm. The integration of the data using a triclinic unit cell yielded a total of 57033 reflections to a maximum θ angle of 27.48° (0.77 Å resolution), of which 3879 were independent (average redundancy 14.703, completeness = 100.0%, Rint = 6.76%, Rsig = 2.67%) and 3014 (77.70%) were greater than 2σ(F2). The final cell constants of a = 7.2901(2) Å, b = 10.5132(3) Å, c = 11.8680(4) Å, α = 71.2030(10)°, β = 79.0020(10)°, γ = 83.4110(10)°, volume = 843.87(4) Å3, are based upon the refinement of the XYZ-centroids of 9920 reflections above 20 σ(I) with 5.702° < 2θ < 54.71°. Data were corrected for absorption effects using the Multi-Scan method (SADABS).2 The ratio of minimum to maximum apparent transmission was 0.963. The calculated minimum and maximum transmission coefficients (based on crystal size) are 0.9850 and 0.9900. Please refer to Table 1 for additional crystal and refinement information.

Structure solution and refinement

The space group P-1 was determined based on intensity statistics and systematic absences. The structure was solved using the SHELX suite of programs3 and refined using full-matrix least-squares on F2 within the OLEX2 suite.4 An intrinsic phasing solution was calculated, which provided most non-hydrogen atoms from the E-map. Full-matrix least squares / difference Fourier cycles were performed, which located the remaining non-hydrogen atoms. All non-hydrogen atoms were refined with anisotropic displacement parameters. The hydrogen atoms were placed in ideal positions and refined as riding atoms with relative isotropic displacement parameters. The

final full matrix least squares refinement converged to R1 = 0.0466 and wR2 = 0.1246 (F2, all data). The goodness-of-fit was 1.024. On the basis of the final model, the calculated density was 1.292 g/cm3 and F(000), 348 e-. Disorder was modelled on the phenyl ring.

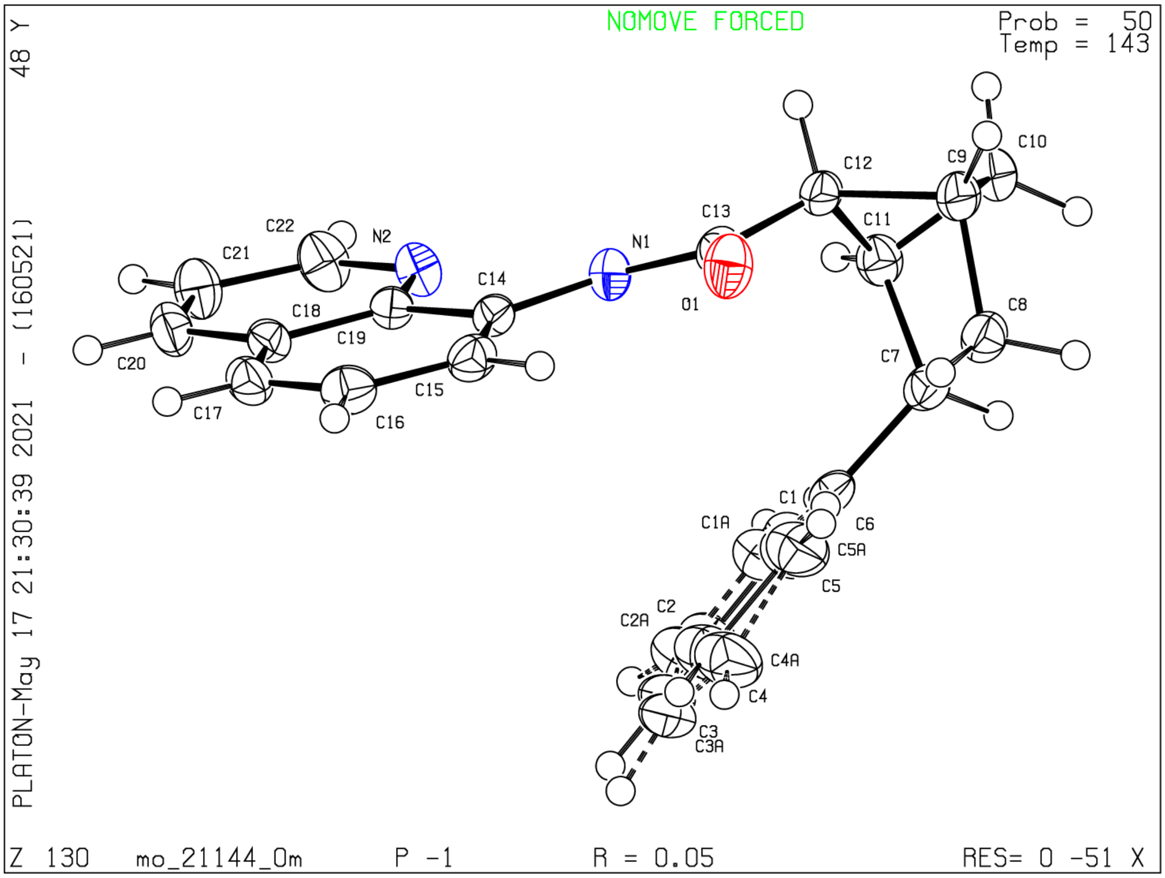

Table 1. Crystal data and structure refinement for mo_21144_0m.

Empirical formula	C22 H20 N2 O	
Formula weight	328.40	
Crystal color, shape, size	colourless block, 0.19 × 0.14 × 0.13 mm^3	
Temperature	143.0 K	
Wavelength	0.71073 Å	
Crystal system, space group	Triclinic, P-1	
Unit cell dimensions	a = 7.2901(2) Å	α = 71.2030(10)°.
	b = 10.5132(3) Å	β = 79.0020(10)°.
	c = 11.8680(4) Å	γ = 83.4110(10)°.
Volume	843.87(4) $Å^3$	
Z	2	
Density (calculated)	1.292 g/cm^3	
Absorption coefficient	0.080 mm^{-1}	
F(000)	348	
**Data collection**		
Diffractometer	Bruker VENTURE D8	
Theta range for data collection	2.050 to 27.484°.	
Index ranges	-9<=h<=9, -13<=k<=13, -15<=l<=15	
Reflections collected	57033	
Independent reflections	3879 [R_{int} = 0.0676]	
Observed Reflections	3014	
Completeness to theta = 25.242°	100.0 %	
**Solution and Refinement**		
Absorption correction	Semi-empirical from equivalents	
Max. and min. transmission	0.7456 and 0.7177	
Solution	Intrinsic methods	
Refinement method	Full-matrix least-squares on F^2	
Weighting scheme	$w = [\sigma^2Fo^2+ AP^2+ BP]^{-1}$, with	
	$P = (Fo^2+ 2 Fc^2)/3$, A = 0.0579, B = 0.3404	
Data / restraints / parameters	3879 / 11 / 242	
Goodness-of-fit on F^2	1.024	
Final R indices [$I>2\sigma(I)$]	R1 = 0.0466, wR2 = 0.1149	
R indices (all data)	R1 = 0.0645, wR2 = 0.1246	
Extinction coefficient	n/a	
Largest diff. peak and hole	0.342 and -0.285 e.$Å^{-3}$	

8. Reference:

[i] E. J. Enholm, H. Satici, G. Prasad. *J. Org. Chem*. **1990**, *55*, 324–329.
[ii] J. L. Alterman, D. X. Vang, M. R. Stroud, L. J. Halverson, G. A. Kraus. *Org. Lett.* **2020**, *22*, 7424–7426.
[iii] L. D. Elliott, S. Kayal., M. W. George, K. Booker-Milburn. *J. Am. Chem. Soc.* **2020**, *142*, 14947–14956.
[iv] J. T. Kley, J. Mack, B. Hamilton, S. Scheuerer, N. Redemann. *Bioorg. Med. Chem. Lett*. **2011**, *21*, 5924-592.
[v] O. E. Edwards, J. Dixon, J. W. Elder, R.J. Kolt, M. Lesage. *Canadian Journal of Chemistry*. **1981**, *59,* 2096–2115.
